# ﻿Morphological investigation of genital organs and first insights into the phylogeny of the genus *Siciliaria* Vest, 1867 as a basis for a taxonomic revision (Mollusca, Gastropoda, Clausiliidae)

**DOI:** 10.3897/zookeys.1077.67081

**Published:** 2021-12-14

**Authors:** Willy De Mattia, Susanne Reier, Elisabeth Haring

**Affiliations:** 1 Central Research Laboratories, of Natural History Museum Vienna, Burgring 7, 1010 Vienna, Austria Central Research Laboratories, of Natural History Museum Vienna Vienna Austria; 2 Department of Evolutionary Biology, University of Vienna, Djerassiplatz 1, 1030 Vienna, Austria University of Vienna Vienna Austria

**Keywords:** The taxonomy and systematics of the door snail genus *Siciliaria* was revised based on an integrative approach including a comprehensive genital anatomical investigation, which was combined with shell morphology and DNA sequence data (mitochondrial COI, nuclear ITS2 sequences). The genital morphology of 120 specimens of 22 taxa from 44 populations was investigated, and a new general description of the genital morphology of the genus is provided. Additionally, 26 specimens of 14 taxa of five genera (MolluscaBase 2021) of Alopiinae (*Mauritanica*, *Charpentieria*, *Stigmatica*, *Gibbularia*, *Papillifera*) were included in the genetic analyses. New anatomical structures are described: the parapseudopapilla for *Charpentieriadyodon* and the hemipapilla for *Charpentieriastenzii*. In the phylogenetic tree based on COI sequences, the species of the genus *Siciliaria* s. l. from northwestern Sicily were found within two separate highly supported main clades. In the tree based on the nuclear ITS2 marker sequence, resolution was considerably lower but it partially confirmed the mitochondrial tree. The genus *Sicania* Tomlin, 1929 (corresponding to main clade II in the trees) is re-introduced. *Siciliariascarificata* did not appear in one of the two main clades but clustered together with *Mauritanicaperinnipolygyra*. Concerning monophyly of species, only the widely distributed *S.calcarae* was paraphyletic in the COI tree, a finding that has to be investigated further with multiple marker sequences. For the other genera (*Mauritanica*, *Charpentieria*, *Stigmatica*, *Gibbularia*, and *Papillifera*) detailed descriptions of the anatomy of the genital organs of 46 taxa for a total amount of 133 dissected specimens are also provided here. Some of these taxa could be included in the phylogenetic analysis. Although the taxon sampling of these taxa was far from being complete, the comprehensive data provided here (concerning morphology, genetics, and distribution) provide first insights into the phylogenetic relationships of this diverse group of clausiliid taxa. The following six taxa new to science are described: *Siciliariagrohmannianaaddaurae***ssp. nov.**, *Siciliariacalcaraeborgettensis***ssp. nov.**, *Siciliariacalcaraejatinensis***ssp. nov.**, *Siciliariacalcaraeparajatinensis***ssp. nov.**, *Siciliariacalcaraecruenta* **ssp. nov.**, and *Siciliariatiberiiarmettensis***ssp. nov.** Anatomy of genital organs, Clausiliidae, phylogeny, *Siciliaria*, systematics, taxonomy

## ﻿Introduction

*Siciliaria* Vest, 1867 is a genus of the family Clausiliidae that is distributed along the calcareous ranges of northwestern Sicily (Italy). It is distributed from Egadi Islands and Trapani in the west to western Madonie in the east. The southern boundaries are represented by the line (west to east) Trapani, Calatafimi, Bosco Ficuzza and Monte San Calogero.

Vest (1867: 166) introduced the genus name *Siciliaria* (type species *Clausiliagrohmanniana* Rossmässler 1836) for a group of Sicilian rock-dwelling clausiliids species also including *Clausiliasyracusana* Philippi, 1836 [currently *Muticariasyracusana* (Philippi, 1836)] from the Iblean Peninsula. Schmidt (1868: 40–43) proposed a first rearrangement, creating the “Formenkreis” of *Clausiliaseptemplicata*, that included all the northwestern Sicilian species, oddly neglecting the genus name *Siciliaria*Vest (1867).

Later, Boettger (1877: 33) split the species into two groups based on the shape of the clausilium, shell sculpture and the development of the plicae and lamellae (Nordsieck 2013b). The “Gruppe der *septemplicata* Phil.” for the *Siciliaria* s. s. species [namely. *Siciliariagrohmanniana*, Siciliaria (Siciliaria) septemplicata, Siciliaria (Siciliaria) calcarae, *Siciliariaadelina* (currently a junior synonym of *Siciliariacalcarae*), Siciliaria (Siciliaria) confinata (currently *Mauritanicascarificata* comb. nov.) and Siciliaria (Siciliaria) tiberii], which he characterised by “Clausilium vorn am Aussenrand stark umgeschlagen”. The “Gruppe der *crassicostata* Ben”, for which he introduced the name *Trinacria* [namely Siciliaria (Trinacria) crassicostata, Siciliaria (Trinacria) crassicostata var. eminens (currently *Sicaniaeminens* comb. nov.), Siciliaria (Trinacria) leucophryna (currently *Siciliarialeucophryna*) and Siciliaria (Trinacria) nobilis] and which he described with “Clausilium tief rinnenförmig, vorn dick und schräg abgestutzt, schwach ausgerandet”.

**Figure 1.**
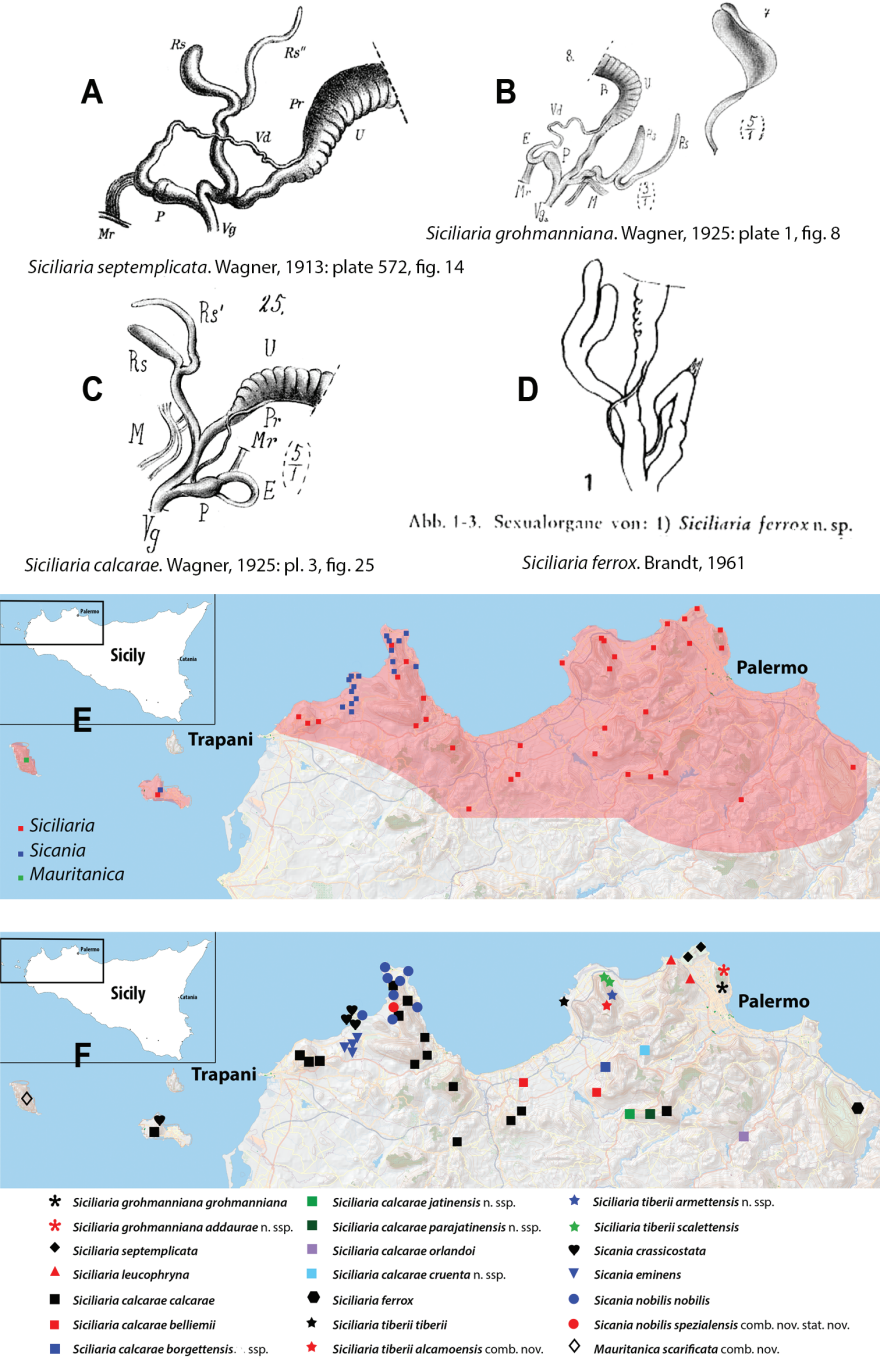
Wagner’s (1913, 1925) and Brandt’s (1961)*Siciliaria* genital drawings **A***Siciliariaseptemplicata*, Wagner, 1913: plate 572, fig. 14 **B***Siciliariagrohmannianagrohmanniana*, Wagner, 1925: plate 1, fig. 8 **C***Siciliariacalcaraecalcarae*, 1925: plate 3, fig. 25 **D***Siciliariaferrox*, Brandt, 1961: fig. 1 **E** overall *Siciliaria/Sicania* and Sicilian *Mauritanica* distribution **F***Siciliaria/Sicania* and Sicilian *Mauritanica* distribution of taxa.

The name *Sicania* had been introduced in order to replace the pre-occupied name *Trinacria*Boettger 1877 (the senior homonym is *Trinacria* Mayer, 1868; Bivalvia: Glycymerididae). Few additional contributions dealt with northwestern Sicilian *Siciliaria*, namely Benoit (1881: 101–120) with a synonym list and some information about distribution of species and Westerlund (1892: 47–48) who simply reported a species list. The genital organs of representatives of *Siciliaria* were treated for the first time by Wagner (1913, 1925), who depicted three northwestern Sicilian species [as Delima (Siciliaria)]: *S.septemplicata*: Wagner 1913: pl. 572, fig. 14; *S.grohmanniana*: Wagner 1925: pl. 1, fig. 8, *S.calcarae*: Wagner 1925: pl. 3, fig. 25. Wagner’s genital anatomical drawings are reproduced in Fig. 1A–C.

**Figure 2.**
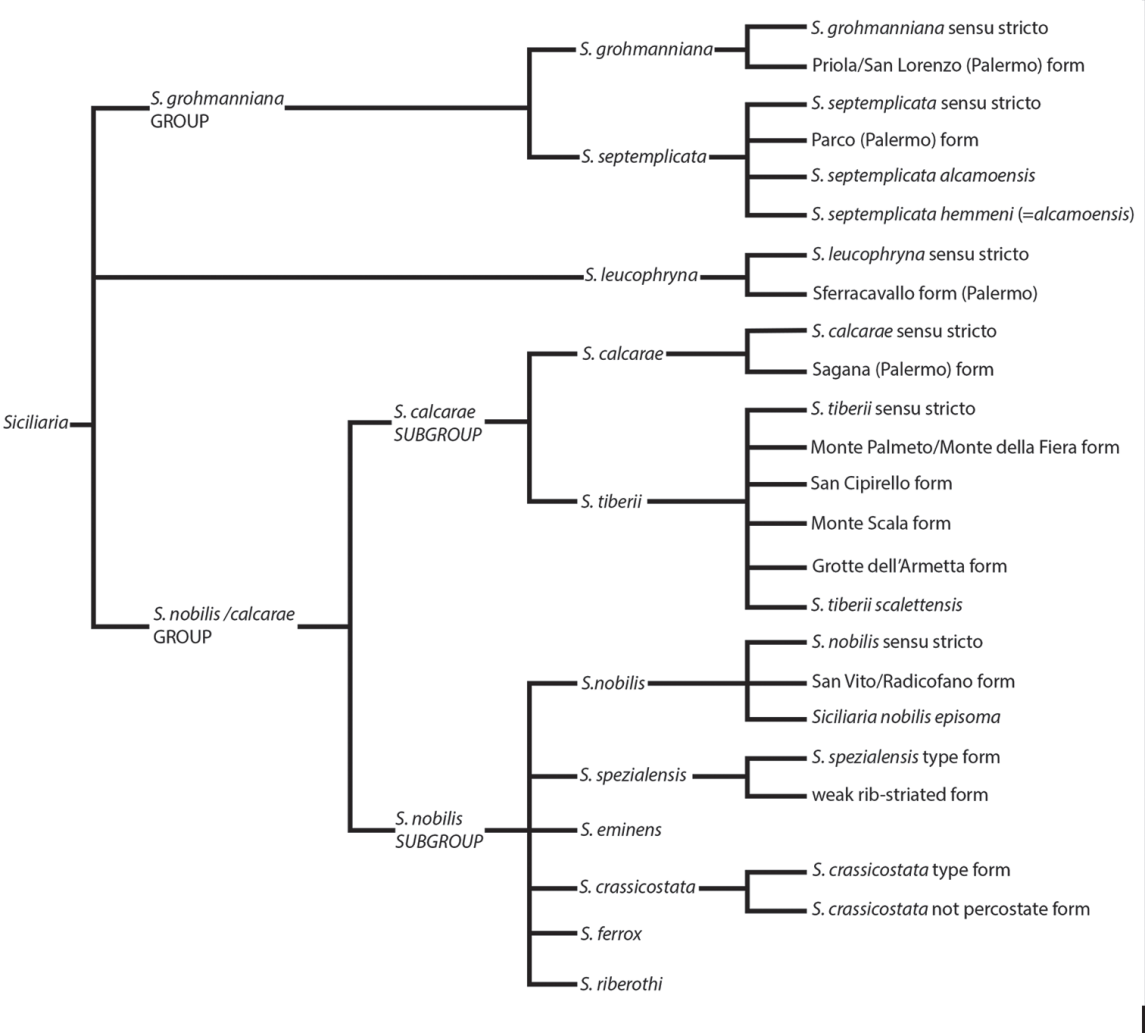
Qualitative shell-based tree depicting Nordsieck´s (2013b)*Siciliaria* system.

More recently, Brandt (1961: 13) depicted the distal genital organs of *Siciliariaferrox* Brandt, 1961 (Fig. 1D). Brandt (1961) introduced five new taxa and made a few systematic arrangements, but his results are inconclusive due to unidentified or wrong localities reported on the material’s labels housed at SMF (mainly provided by Monterosato). Alzona (1971: 90–92) did not list Brandt’s new taxa, although his paper was cited. Nordsieck (1979: 259–260) united Boettger’s groups, because he considered *S.calcarae* (Philippi, 1844) conchologically as an intermediate form. Nordsieck (1984: 202) described *Siciliariaspezialensis*. Later, Nordsieck (2002: 29) considered *Siciliaria* as a subgenus of *Charpentieria* Stabile, 1864 and listed 12 species. Within the genus *Charpentiera*, he grouped many other genera as subgenera, viz. *Stigmatica* Boettger, 1877, *Gibbularia* Monterosato, 1908 and *Mauritanica* Boettger, 1879, creating a new system based on shell characters (Fig. 2). Beckmann (2004) introduced two new subspecies (*Siciliariatiberiiscalettensis*, *Siciliariaseptemplicatahemmeni*), both considered to be conchologically intermediate “hybrid forms”. Nordsieck (2007) kept *Siciliaria* (and all the above-mentioned genera) as subgenus of *Charpentieria*. In the last two decades, contributions to the knowledge of the distribution of species were provided by Reitano et al. (2007: 326) [*Siciliariaferrox* (Brandt, 1961)]; Reitano et al. (2012) [*Siciliarialeucophryna* (Pfeiffer, 1862)] and Liberto et al. (2015) [*Siciliariascarificata* (L. Pfeiffer, 1856)] and two new subspecies were recently described: *Siciliariacalcaraeorlandoi* Liberto, Reitano, Giglio, Colomba & Sparacio, 2016 and *Siciliarialeucophrynamicroinsularis* Sparacio, Surdo, Viviano, Liberto & Reitano, 2021. For a comprehensive recap about the systematics and the nomenclatural history of *Siciliaria* see Nordsieck (2013b: 1–2). This system changed in the following Nordsieck’s papers (2013a; 2013b) where *Charpentieria* and *Siciliaria* were treated again as separated genera and *Stigmatica*, *Gibbularia* and *Mauritanica* were considered as subgenera of *Siciliaria*. Nordsieck (2013b: 2) stated that the genital morphology of all *Siciliaria* species was similar. While that analysis concentrated on the external characters of genital organs (shape, proportions among anatomical parts) and, to some extent, to the pseudopapilla, the internal morphology was not investigated.

Nordsieck (2013a: 4) again separated *Siciliaria* from *Charpentieria* and listed the presence/absence of the thickening as a delimitation of the proximal part of the penis, being distinct or not as the main differences in the morphology of genital organs among *Charpentieria*, *Siciliaria*, *Stigmatica*, and *Mauritanica.* In detail, he stated that in *Charpentieria* the ”epiphallar thickening at the transition epiphallus-penis was missing” and “the delimitation of the proximal part of the penis was distinct”. In *Siciliaria* s. l. the epiphallar thickening should be present [except for S. (Gibbularia)] and the delimitation of the proximal part of the penis was described to be indistinct. *Mauritanica* was deemed as belonging to *Siciliaria* because it exhibits the same characters. Moreover, he stated that *Siciliaria* could be morphologically distinguished from *Charpentieria* only “by the defined characters”. Yet, Nordsieck (2013a: 4) introduced the term “epiphallar thickening” without providing a clear explanation for this term. Given that it concerns the transition between penis and epiphallus, it is not clear whether the epiphallar thickening was an external or internal character of the genital organs. Most likely, it concerns the external appearance of the transition penis-epiphallus, where the epiphallar thickening reveals a clear distinction between penis and epiphallus; it was already depicted by Wagner (1913, 1925) (Fig. 1). In his paper dealing with southern Alpine *Charpentieria* (Nordsieck 1963b), which includes a good number of genital drawings, the concept of epiphallar thickening was not mentioned nor depicted. Our genital anatomical investigations of the internal arrangement of the distal genital organs revealed an intriguing, diversified, and complex situation that may not be summarised by the character states dichotomy proposed by Nordsieck (1963a: 95, figs 9–10; 97, fig. 11; 1963b: 185, figs 4751; 189: figs 52–54; 193: figs 55–56; 1979, 2013).

According to the currently accepted system (MolluscaBase 2021), Siciliaria is deemed as a subgenus of the all-inclusive genus Charpentieria, with 12 species and 17 subspecies. It currently comprises six subgenera with 29 taxa and ~ 70 subspecific taxa. The phylogenetic relationships among *Siciliaria*, *Charpentieria*, *Stigmatica*, *Gibbularia* and *Mauritanica* are still unclear. Scheel and Hausdorf (2012), Uit de Weerd and Gittenberger (2013) and Xu and Hausdorf (2021) included Delimini species in their phylogentic analyses, but those articles concentrated on other aims (south Alpine *Charpentieria*, general Clausiliidae phylogeny) and thus the phylogeny of Delimini is still largely incomplete. Nordsieck (2020) summarised these results by proposing that *Siciliaria* and *Charpentieria* should be separate genera and by recognising a geographic split within *Siciliaria*. He thus introduced again the name *Sicania* as a subgenus of *Siciliaria*, but stressing that there is no morphological evidence supporting such a dichotomy. Moreover, *Mauritanica* was deemed as a subgenus of *Siciliaria*, whereas for *Stigmatica* the need for further research was emphasised.

### ﻿Aims of the study

In the present study a comprehensive investigation of the genus *Siciliaria* Vest, 1867 (type species: *Clausiliagrohamnniana* Rossmässler, 1836) was performed, including morphology of the genital organs and DNA sequence analyses, which were interpreted in the light of the current taxonomic system (Nordsieck 2007, 2013a; MolluscaBase 2021). In addition to *Siciliaria* (*sensu* MolluscaBase 2021), 14 taxa belonging to genera related to *Siciliaria* (namely *Charpentieria*, *Stigmatica*, *Gibbularia*, *Papillifera* and *Mauritanica*) were included in the molecular genetic study as a first attempt to elucidate their relationships using *Papillifera* as outgroup. Yet, we were well aware that the low number of samples would provide only a preliminary insight. We systematically introduced new genital anatomical characters for the above listed genera. This study follows the strategy and methods of the recently published integrative approach to the taxonomy and systematics within *Montenegrina* Boettger, 1877 (De Mattia et al. 2020; Mason et al. 2020a), where DNA-based phylogenetic trees together with morphological characters (both shell and morphology of genital organs) were the basis for a taxonomic revision.

## ﻿Materials and methods

Most of the Sicilian material (mainly *Siciliaria* and *Papillifera*), unless differently stated, was collected during four field collecting trips by the first author (WDM) and currently kept in the Willy De Mattia collection (Syracuse, Italy). The type material of the newly described taxa was deposited in the Natural History Museum in Vienna. Part of the material was made available by other private collections. In order to increase the sample (n) for shell measurements, also dead material was extensively collected. In this study we investigated the anatomy of the genital organs of 120 specimens from 44 populations representing 16 of the 17 taxa presently listed in MolluscaBase eds. (2021). The identification of taxa was based following Nordsieck (2013b) and personal communications with H. Nordsieck.

The non-Sicilian specimens were also collected mostly by the first author (WDM) during collecting trips throughout the central and eastern southern Alps, Italian peninsula, and western Balkans from 1992 to date, though interesting specimens belonging to *Stigmatica* and *Papillifera* were provided by other collectors. As regards the other genera (*Mauritanica*, *Charpentieria*, *Stigmatica*, *Gibbularia*, and *Papillifera*), 133 specimens of 46 taxa were anatomically investigated.

Ninety-four specimens comprising *Siciliaria*, *Charpentieria*, *Gibbularia*, *Stigmatica*, *Mauritanica*, and *Papillifera* were genetically analyzed (see Table 1). The remaining DNA is stored in the DNA/Tissue collection of the Natural History Museum Vienna.

**Table 1.** Specimens used in the molecular genetic analyses. Collection numbers, laboratory IDs, GenBank accession numbers, and geographical coordinates are listed. More than one ITS2 sequence was obtained from some individuals.

Taxon	Collection No.	Lab ID	GenBank Accession No.		Coordinates
			COI	ITS2	
*Sicaniacrassicostata* (L. Pfeiffer, 1856)	WDM Siciliaria 1	1_1	MW758912	MW757114, MW757113	38°05'40.91"N, 12°40'10.98"E
	WDM Siciliaria 1	1_4	MW758924	no	38°05'40.91"N, 12°40'10.98"E
*Mauritanicascarificata* (L. Pfeiffer 1856)	WDM Siciliaria 2	2_1	MW758915	MW757112, MW757111	37°58'11.08"N, 12°03'59.36"E
	WDM Siciliaria 2	2_2	MW758916	no	37°58'11.08"N, 12°03'59.36"E
*Siciliariacalcaraecalcarae* (Philippi 1844)	WDM Siciliaria 5	5_1	MW758925	MW757125, MW757126, MW757127	37°57'29.40"N, 12°57'33.64"E
*Sicanianobilisnobilis* (L. Pfeiffer, 1848)	WDM Siciliaria 14	14_1	MW758875	MW757128, MW757129, MW757130	38°10'51.85"N, 12°43'37.70"E
*Siciliariatiberiiscalettensis* Beckmann, 2004	WDM Siciliaria 17	17_2	MW758923	MW757110, MW757109, MW757108	38°10'35.53"N, 13°07'27.22"E
*Sicanianobilisnobilis* (L. Pfeiffer, 1848)	WDM Siciliaria 18	18_1	MW758918	MW757107, MW757106	38°10'30.07"N, 12°46'14.09"E
*Siciliariatiberiitiberii* (A. Schmidt, 1868)	WDM Siciliaria 19	19_1	MW758919	MW757117, MW757116	38°08'19.06"N, 13°03'14.01"E
	WDM Siciliaria 19	19_2	MW758920	no	38°08'19.06"N, 13°03'14.01"E
*Siciliariaferrox* (Brandt, 1961)	WDM Siciliaria 21	Sf_1	MW758922	MW757115	37°59'17.62"N, 13°36'51.11"E
	WDM Siciliaria 21	Sf_2	MW758917	no	37°59'17.62"N, 13°36'51.11"E
*Sicaniacrassicostata* (L. Pfeiffer, 1856)	WDM Siciliaria 33	33_1	MW758876	no	38°06'44.89"N, 12°40'34.27"E
	WDM Siciliaria 33	33_2	MW758877	MW757105, MW757104, MW757131, MW757103	38°06'44.89"N, 12°40'34.27"E
*Siciliariacalcaraejatinensis* ssp. nov.	WDM Siciliaria 34	34_1	MW758878	no	37°58'15.07"N, 13°12'2.23"E
	WDM Siciliaria 34	34_2	MW758879	MW757118, MW757120, MW757119, MW757121	37°58'15.07"N, 13°12'2.23"E
	WDM Siciliaria 34	34_3	MW758926	no	37°58'15.07"N, 13°12'2.23"E
	WDM Siciliaria 34	34_4	MW758927	no	37°58'15.07"N, 13°12'2.23"E
*Siciliariacalcaraeorlandoi* Liberto et al., 2016	WDM Siciliaria 36	36_1	MW758921	MW757132, MW757133	37°55'46.42"N, 13°23'5.22"E
*Siciliariatiberiialcamoensis* Brandt, 1961	WDM Siciliaria 37	37_1	MW758928	MW757102, MW757101	38°07'48.60"N, 13°08'26.94"E
*Siciliariatiberiiarmettensis* ssp. nov.	WDM Siciliaria 38	38_1	MW758880	MW757100, MW757099, MW757098	38°08'58.56"N, 13°09'25.83"E
	WDM Siciliaria 38	38_2	MW758881	no	38°08'58.56"N, 13°09'25.83"E
*Siciliariatiberiiscalettensis* Beckmann, 2004	WDM Siciliaria 39	39_1	MW758882	MW757097, MW757096, MW757095	38°10'42.45"N, 13°07'34.50"E
	WDM Siciliaria 40	40_1	no	no	38°10'35.53"N, 13°07'27.22"E
*Siciliariagrohmannianaaddaurae* ssp. nov.	WDM Siciiiaria 41	41_1	MW758883	MW757094, MW757093	38°11'13.01"N, 13°21'6.91"E
	WDM Siciiiaria 41	41_2	MW758884	no	38°11'13.01"N, 13°21'6.91"E
	WDM Siciiiaria 41	41_3	MW758929	no	38°11'13.01"N, 13°21'6.91"E
*Siciliariagrohmannianagrohmanniana* (Rossmässler, 1836)	WDM Siciliaria 42	42_1	MW758886	no	38°10'4.41"N, 13°21'2.60"E
	WDM Siciliaria 42	42_2	MW758887	MW757092, MW757091	38°10'4.41"N, 13°21'2.60"E
	WDM Siciliaria 42	42_3	MW758885	no	38°10'4.41"N, 13°21'2.60"E
*Siciliarialeucophryna* (L. Pfeiffer, 1862)	WDM Siciliaria 43	43_1	MW758888	no	38°12'1.32"N, 13°16'3.05"E
*Siciliariacalcaraebelliemii* (Brandt, 1961)	WDM Siciliaria 44	44_1	MW758889	MW757122, MW757123	38°00'54.80"N, 12°59'13.35"E
	WDM Siciliaria 44	44_2	MW758913	no	38°00'54.80"N, 12°59'13.35"E
	WDM Siciliaria 44	44_3	MW758930	no	38°00'54.80"N, 12°59'13.35"E
	WDM Siciliaria 44	44_4	MW758931	no	38°00'54.80"N, 12°59'13.35"E
*Siciliariacalcaraeborgettensis* ssp. nov.	WDM Siciliaria 46	46_1	MW758890	MW757090, MW757089, MW757080	38°02'59.53"N, 13°08'58.55"E
	WDM Siciliaria 46	46_2	MW758891	no	38°02'59.53"N, 13°08'58.55"E
	WDM Siciliaria 46	46_3	MW758932	no	38°02'59.53"N, 13°08'58.55"E
*Siciliariacalcaraecruenta* ssp. nov.	WDM Siciliaria 47	47_1	MW758933	MW757079, MW757078	38°03'37.03"N, 13°12'38.53"E
	WDM Siciliaria 47	47_2	MW758934	MW757088, MW757087, MW757077	38°03'37.03"N, 13°12'38.53"E
	WDM Siciliaria 47	47_3	MW758935	no	38°03'37.03"N, 13°12'38.53"E
	WDM Siciliaria 47	47_4	MW758936	no	38°03'37.03"N, 13°12'38.53"E
*Sicaniaeminens* (A. Schmidt, 1868)	WDM Siciliaria 49	49_1	MW758892	no	38°04'0.52"N, 12°41'1.34"E
	WDM Siciliaria 49	49_2	MW758893	no	38°04'0.52"N, 12°41'1.34"E
	WDM Siciliaria 50	50_1	MW758894	MW757076, MW757075, MW757074, MW757073	38°04'6.38"N, 12°41'48.68"E
	WDM Siciliaria 50	50_2	MW758895	no	38°04'6.38"N, 12°41'48.68"E
	WDM Siciliaria 51	51_1	MW758896	MW757072	38°04'55.97"N, 12°40'9.91"E
	WDM Siciliaria 51	51_2	MW758897	no	38°04'55.97"N, 12°40'9.91"E
	WDM Siciliaria 52	52_1	MW758898	no	38°05'18.10"N, 12°40'17.18"E
	WDM Siciliaria 52	52_2	MW758899	MW757086, MW757071, MW757085	38°05'18.10"N, 12°40'17.18"E
*Siciliariacalcaraebelliemii* (Brandt, 1961)	WDM Siciliaria 54	54_1	MW758900	MW757134, MW757135, MW757136	38°00'22.47"N, 13°06'37.79"E
	WDM Siciliaria 54	54_2	MW758901	no	38°00'22.47"N, 13°06'37.79"E
*Siciliarialeucophryna* (L. Pfeiffer, 1862)	WDM Siciliaria 55	55_1	MW758902	MW757124	38°11'13.61"N, 13°16'44.68"E
	WDM Siciliaria 55	55_2	MW758903	no	38°11'13.61"N, 13°16'44.68"E
*Siciliariaseptemplicata* (Philippi, 1836)	WDM Siciliaria 56	56_1	MW758904	MW757137, MW757138	38°12'39.27"N, 13°17'21.26"E
	WDM Siciliaria 57	57_1	MW758937	MW757070	38°13'5.61"N, 13°18'4.76"E
*Siciliariacalcaraecalcarae* (Philippi 1844)	WDM Siciliaria 59	59_1	MW758905	MW757084, MW757069, MW757068	38°02'54.18"N, 12°48'26.61"E
*Sicanianobilisnobilis* (L. Pfeiffer, 1848)	WDM Siciliaria 60	60_1	MW758906	MW757067, MW757066, MW757139, MW757065	38°06'25.71"N, 12°44'33.37"E
	WDM Siciliaria 60	60_2	MW758907	no	38°06'25.71"N, 12°44'33.37"E
	WDM Siciliaria 60	60_4	MW758938	no	38°06'25.71"N, 12°44'33.37"E
	WDM Siciliaria 60	60_5	MW758939	no	38°06'25.71"N, 12°44'33.37"E
*Siciliariacalcaraecalcarae* (Philippi 1844)	WDM Siciliaria 61	61_1	MW758908	MW757140, MW757141	38°06'25.71"N, 12°44'33.37"E
	WDM Siciliaria 61	61_2	MW758909	MW757142, MW757143, MW757144	38°06'25.71"N, 12°44'33.37"E
*Sicanianobilisnobilis* (L. Pfeiffer, 1848)	WDM Siciliaria 62	62_2	MW758914	no	38°08'48.81"N, 12°44'6.61"E
	WDM Siciliaria 62	62_3	MW758940	no	38°08'48.81"N, 12°44'6.61"E
*Sicanianobilisspezialensis* (Nordsieck, 1984)	WDM Siciliaria 63	63_1	MW758910	MW757040, MW757039	38°07'41.78"N, 12°44'9.24"E
	WDM Siciliaria 63	63_2	MW758911	no	38°07'41.78"N, 12°44'9.24"E
*Charpentieriaitalalorinae* (Gredler, 1869)	WDM Charpentieria 3	C3_2	MW758954	no	45°50'23.18"N, 10°36'49.06"E
*Charpentieriastenziicincta* (Brumati, 1838)	WDM Charpentieria 2	C2_2	MW758953	MW757050, MW757049	46°11'40.51"N, 12°38'14.26"E
*Charpentieriaitalaitala* (G. von Martens, 1824)	WDM Charpentieria 1	C1_1	MW758952	no	45°24'22.30"N, 11°31'11.93"E
*Charpentieriastenziiletochana* (Gredler, 1874)	WDM Charpentieria 19	C19_1	MW758957	no	46°36'27.42"N, 12°12'20.01"E
*Charpentieriaitalabaldensis* (Strobel, 1851)	WDM Charpentieria 20	C20_1	MW758955	no	45°44'25.95"N, 10°51'13.69"E
*Charpentieriadyodondyodon* (S. Studer, 1820)	WDM Charpentieria 44	C44_1	MW758956	MW757052, MW757051	46°12'29.53"N, 8°12'46.18"E
	WDM Charpentieria 44	C44_3	MW758951	no	46°12'29.53"N, 8°12'46.18"E
*Gibbulariagibbulagibbula* (Rossmässler, 1836)	Nordsieck 12167	12167_1	MW758947	no	41°47'1.22"N, 15°26'39.92"E
	Nordsieck 12167	12167_2	MW758948	MW757057, MW757056, MW757055	41°47'1.22"N, 15°26'39.92"E
	WDM Gibbularia 64	G64_1	MW758958	MW757048, MW757083, MW757082	45°36'8.91"N, 13°46'0.72"E
	WDM Gibbularia 64	G64_2	MW758959	no	45°36'8.91"N, 13°46'0.72"E
*Mauritanicaperinnipolygyra* (O. Boettger, 1879	SMF335011/1	SMF 335011_1	MW758966	MW757045, MW757044	36°22'2.45"N, 10°07'11.81"E
*Papilliferapapillarispapillaris* (O.F. Müller, 1774)	Nordsieck 11786	11786_1	MW758941	MW757063, MW757062, MW757064	42°01'4.58"N, 12°54'19.30"E
	Nordsieck 11786	11786_2	MW758942	no	42°01'4.58"N, 12°54'19.30"E
*Papilliferasolidasolida* (Draparnaud, 1805)	Nordsieck 12100	12100_1	MW758944	no	40°40'1.62"N, 16°37'16.08"E
*Papilliferapapillaristransitans* (Paulucci, 1878)	Nordsieck 12147	12147_1	MW758945	no	38°28'45.91"N, 16°27'48.05"E
	Nordsieck 12147	12147_2	MW758946	MW757059, MW757058	38°28'45.91"N, 16°27'48.05"E
*Papilliferapapillarisaffinis* (Philippi, 1836)	WDM Papillifera 2	P2_1	MW758960	no	37°58'2.36"N, 13°11'45.19"E
	WDM Papillifera 2	P2_2	MW758961	no	37°58'2.36"N, 13°11'45.19"E
	WDM Papillifera 3	P3_1	MW758962	MW757081, MW757047, MW757046	37°57'16.92"N, 12°58'9.06"E
	WDM Papillifera 4	P4_1	MW758963	no	37°57'58.18"N, 13°13'14.57"E
*Stigmaticastigmaticasturmii* (L. Pfeiffer, 1848)	Nordsieck 12194	12194_1	MW758949	MW757054, MW757053	40°40'31.60"N, 17°56'40.44"E
	Nordsieck 12194	12194_2	MW758950	no	40°40'31.60"N, 17°56'40.44"E
*Stigmaticavulcanicavulcanica* (Benoit, 1860)	Nordsieck 12068	12068_2	MW758943	MW757061, MW757060	39°17'2.08"N, 16°15'30.22"E
*Stigmaticakobeltiana* (Küster, 1876)	WDM Stigmatica 11	St11_1	MW758965	no	39°07'58.01"N, 16°15'01.92"E
*Stigmaticapantocratorispantocratoris* (O. Boettger, 1889)	WDM Stigmatica 9	St9_2	MW758964	MW757042, MW757043, MW757041	39°46'18.23"N, 19°50'47.23"E

The DNA extraction was performed in a sterile room using the QIAmp DNeasy Blood and Tissue Kit (QIAGEN, Hilden, Germany) following the protocol of the manufacturer. A 655 bp long fragment of the mitochondrial (mt) cytochrome c oxidase subunit 1 gene (COI) was amplified using the primers LCO1490_ABOL_Moll_1 (5'-TCAACAAAYCATAARGAYATTGG-3') and HCO2198_ABOL_Moll_1 (5'-TAAACTTCTGGRTGACCAAAAAAYCA-3') (Duda et al. 2017). As nuclear (nc) marker sequence we analyzed the internal transcribed spacer 2 (ITS2) between the two nuclear rRNA genes (5.8S rRNA gene and 28S rRNA gene).

Using the primers 5.8S_LSU-1fw (5'-CTAGCTGCGAGAATTAATGTGA-3') and 28S_LSU-3rv (5'-ACTTTCCCTCACGGTACTTG-3') (Wade and Mordan 2000), which bind in the flanking rRNA genes, fragments with varying lengths (911–957 bp) were amplified (this fragment, hereafter named ITS2, comprised besides ITS2 also short partial sequences of the two rRNA genes). PCR reactions were performed in a final volume of 25 µl containing, 2.5 µl 10× PCR buffer, 1.5 mM MgCl2, 0.2 mM of each dNTP, 0.5 µM of each primer, 0.5 units TopTaq Polymerase and 1 µl template DNA. Each PCR had the following conditions: 94 °C for 3 min, 35 cycles of (94 °C for 30 s, 50 °C for 30 s and 72 °C for 60 s) and 72 °C for 7 min.

The ITS2 PCR fragments proved to be length-variable due to insertions/deletion, and most individuals were heterozygous. To avoid unreadable sequences, PCR products were cloned. For this task, additional PCRs were performed using the proofreading Platinum® Taq DNA Polymerase High Fidelity (Invitrogen, Carlsbad, CA, USA). These PCRs were conducted with different PCR conditions: 95 °C for 2 min, 5 cycles (94 °C for 15 s, 60 °C for 30 s, 72 °C for 1 min), 35 cycles (94 °C for 15 s, 50 °C for 30 s, 72 °C for 1 min) and without final elongation. PCR fragments were purified with the QIAquick Gel Extraction Kit (QIAGEN, Hilden, Germany) and cloned with the TOPO-TA© cloning kit (Invitrogen, Carlsbad, CA, USA). Three to five clones per individual were sequenced.

Sequencing was performed at Microsynth (Balgach, Switzerland) in both directions using the original PCR primers or, for cloned PCR products, using M13 universal primers. Sequences were processed in Geneious 2.10.3 (https://www.geneious.com). COI sequences were unambiguously aligned (605 sites, no missing data), while the alignments of ITS2 sequences was performed in Geneious 2.10.3 using the MAFFT algorithm (Katoh and Standley 2013), resulting in a 985 bp alignment (no missing data). Alignments of both markers were concatenated using Geneious 2.10.3. Neighbour joining (NJ) trees were calculated in MEGA 10.0.5 (Kumar et al. 2018). In addition, Bayesian inference (BI) trees were calculated using MrBayes v.3.2.1 (Huelsenbeck and Ronquist 2001; Ronquist and Huelsenbeck 2003). The best-fit models using Bayesian Information Criterion (BIC) were evaluated for each gene using ModelFinder (Kalyaanamoorthy et al. 2017), which were GTR+F+I+G4 (COI) and K2P+G4 (ITS2) respectively. BI analyses were performed with three runs for 30 × 10^6^ generations and four chains each. Trees and parameters were sampled every 1000^th^ generation. The first 25% of trees were discarded as burn-in and a 50% majority rule consensus tree was built from the remaining trees. Inter- and intraspecific uncorrected p-distances were calculated using MEGA 10.0.5 (Kumar et al. 2018).

The shells of all dissected specimens were photographed in multiple views (front, back, lateral and internal mouth), including the clausilium distal part, with a Canon EOS camera equipped with 60 mm macro lenses mounted on a Kaiser microslide frame for multi-image stacking. Maximum shell height was measured with a digital caliper (accuracy ± 0.05 mm). For each population at least two specimens were dissected (except for *Mauritanicaperinnipolygyra*) in order to reduce the possibility of being misled by abnormities or single freak specimens (Nordsieck 2007).

Anatomical examinations and dissections were performed under a Zeiss stereoscope with a ring LED illumination apparatus, connected to a digital, high-resolution camera and a camera lucida. The genital organs were separated from the rest of the body after a careful crushing of the shell. Dissections were made using a pair of very fine and pointed micro-tweezers (Dumostar Biology 55) and a micro-scissor (FST 150000, Aesculap OC series) in a Petri dish with black paraffin on the bottom and filled with 70% ethanol. The genital organs were fixed with very fine steel micro-pins (commonly used for the preparation of microlepidopteran specimens in entomology). Internal characters of the genital organs (based on cross and longitudinal sections) were examined after dissection with micro-tweezers or a pair of micro-scissors. Measurements were taken using a millimetric measurement scale. The genital organs were photographed in different positions (40–50 high-resolution images) to create an image database. Drawings were prepared by tracing the most representative digital images after contrast enhancement with picture editing software.

**Figure 3.**
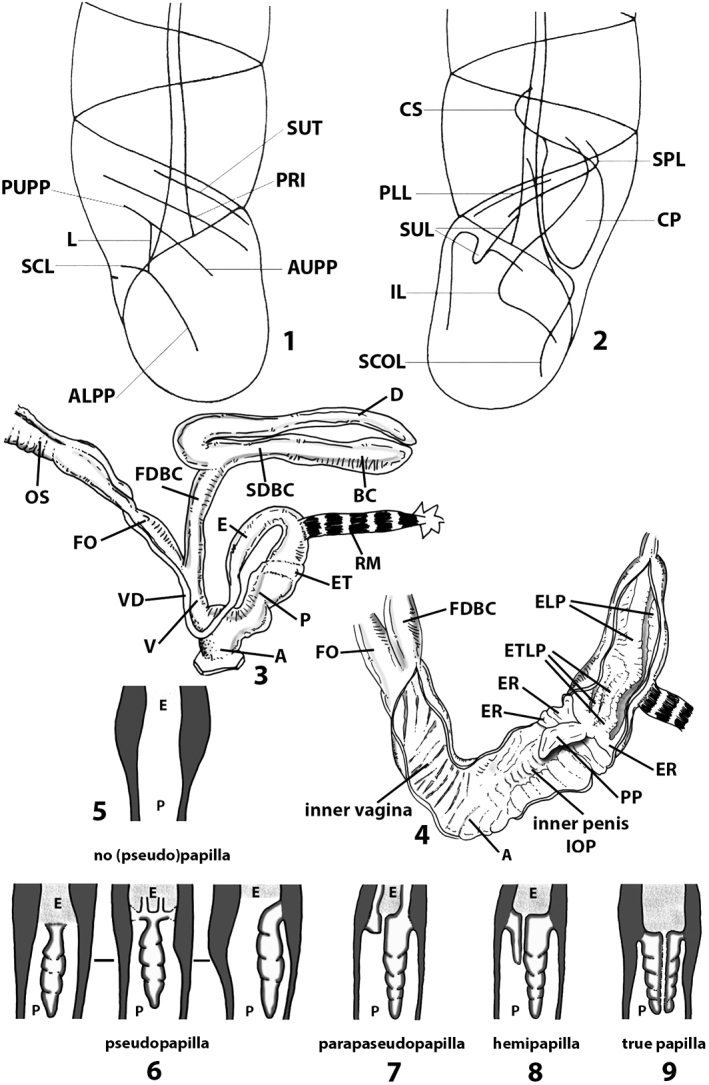
**3.1, 2***Siciliaria*/*Sicania* clausilial apparatus (from hnords.de, modified): **3.1** plicae **3.2** lamellae **3.3, 4***Siciliaria*/*Sicania* anatomy of the genital organs general scheme and (**3.5–9**) variability of the pseudopapilla within genera *Siciliaria*, *Sicania*, *Mauritanica*, *Charpentieria*, *Stigmatica*, *Gibbularia* and *Papillifera***3.5** no (pseudo)papilla **3.6** pseudopapilla **3.7** parapseudopapilla **3.8** hemipapilla **3.9** eupapilla (true penial papilla).

Measurements of anatomical features (e.g., length of P, V, BC+SDBC) were taken using image editing software directly on the high-resolution photographs. The anatomical nomenclature partially follows Nordsieck (1969a, 1972, 2009) and Giusti et al. (1995). The anatomical partition and acronyms are depicted in Fig. 3. Gonads, albumen glands, the first hermaphroditic duct, and most ovispermiducts are neither described nor depicted in drawings as they are deemed as not carrying any reliable taxonomical characters. The VR was found in the same position in all the investigated specimens: thus, in order to render the anatomical drawings clearer and focus on key taxonomic features, it is not depicted.

In order to highlight the proportions among the genital parts, the measurements were converted into relative values (ratios). All the ratios are based on measures of at least two specimens. The ratios are FO/V, D/BC+SDBC, BC+SDBC/V, D/BC+SDBC, P+E/V, and E/P. The measurements of both shell and genital organs are always given in mm. The proximal/distal indications refer as seen from the gonads. The subdivision of the genital apparatus in anatomical parts follows De Mattia et al. (2020).

### ﻿Acronyms

**A** atrium;

**ALPP** anterior lower palatal plica;

**AUPP** anterior upper palatal plica;

**BC** actual bursa copulatrix;

**CP** clausiliar plate;

**CS** clausiliar stalk;

**CSP** cross section of the penial pseudopapilla;

**CSE** cross section of the epiphallus;

**D** diverticulum of the bursa copulatrix;

**E** epiphallus;

**EF** epiphallar funnel;

**ELP** epiphallar longitudinal pleats;

**ER** epiphallar ring;

**ETLP** epiphallar transitional longitudinal pleats;

**EO** epiphallar outlet of the hemipapilla;

**ET** epiphallar thickening;

**FDBC** first duct of the bursa copulatrix;

**FELP** free epiphallar longitudinal plate;

**FO** free oviduct;

**H _a_** aperture height;

**H _s_** shell height;

**HG** hermaphroditic gland;

**H _s_** shell height;

**IOP** internal sculpturing of penis;

**IL** columellaris or inferior lamella;

**L** lunella;

**OELP** occupied epiphallar longitudinal plate;

**OS** ovispermiduct (second hermaphroditic duct);

**P** penis;

**PC** penial complex (P+E);

**PCL** penial complex length;

**PG** prostatic gland;

**PLL** parallelis;

**PP** penial pseudopapilla;

**PPP** parapseudopapilla;

**PR** penial retractor muscle;

**PRI** principalis;

**PUPP** posterior upper palatal plica;

**SCL** subclaustralis;

**SCOL** subcolumellatis;

**SDBC** second duct of the bursa copulatrix;

**SG** sperm groove;

**SPL** spiralis;

**spm** specimen(s);

**SUL** sulcalis or parietalis;

**SUT** suturalis;

**TP** transition passage;

**V** vagina;

**VD** vas deferens;

**VR** vaginal retractor;

**W _a_** aperture width;

**W _s_** shell last whorl width (body whorl).

**CWDM** malacological collection of Willy De Mattia;

**NHMW**Natural History Museum Vienna (Naturhistorisches Museum Wien);

**SMF**Senkenberg Museum Frankfurt.

### ﻿Structure and rationale of *Siciliaria/Sicania* taxonomic analysis

In the first part of the Results we describe the molecular genetic data. For clarity, and in view of the various taxonomic changes in the past, a comprehensive table is presented at the end of the taxonomic part including the new full checklist of the taxa so far considered as *Siciliaria* (Table 12). In the following, we refer to these taxa as belonging to “*Siciliaria*/*Sicania*“. We decided to apply the new taxonomic system already in the phylogenetic trees and their discussion, as well as in the morphological descriptions of the taxonomic part. In the general discussion we interpret the molecular and anatomical results and give an overview on the, so far, unidentified or enigmatic taxa mentioned in the literature.

The taxonomic part of the results comprises the descriptions of the taxa with a detailed list of examined specimens and the principal systematic and/or faunistic publications. For the specimens investigated genetically, the identifier is listed together with the GenBank accession numbers (in square brackets) for sequences of the mt cytochrome c oxidase subunit 1 gene (COI) and where available for the internal transcribed spacer 2 (ITS2). The descriptions of the shells and the clausiliar apparatus of those taxa that are currently valid (MolluscaBase 2021) were already provided and discussed by Nordsieck (2013b). We report here only the descriptions provided by Nordsieck (2013b). For the newly described taxa, the shell and clausiliar apparatus are described with full details.

## ﻿Results

### ﻿Phylogenetic trees

**Figure 4.**
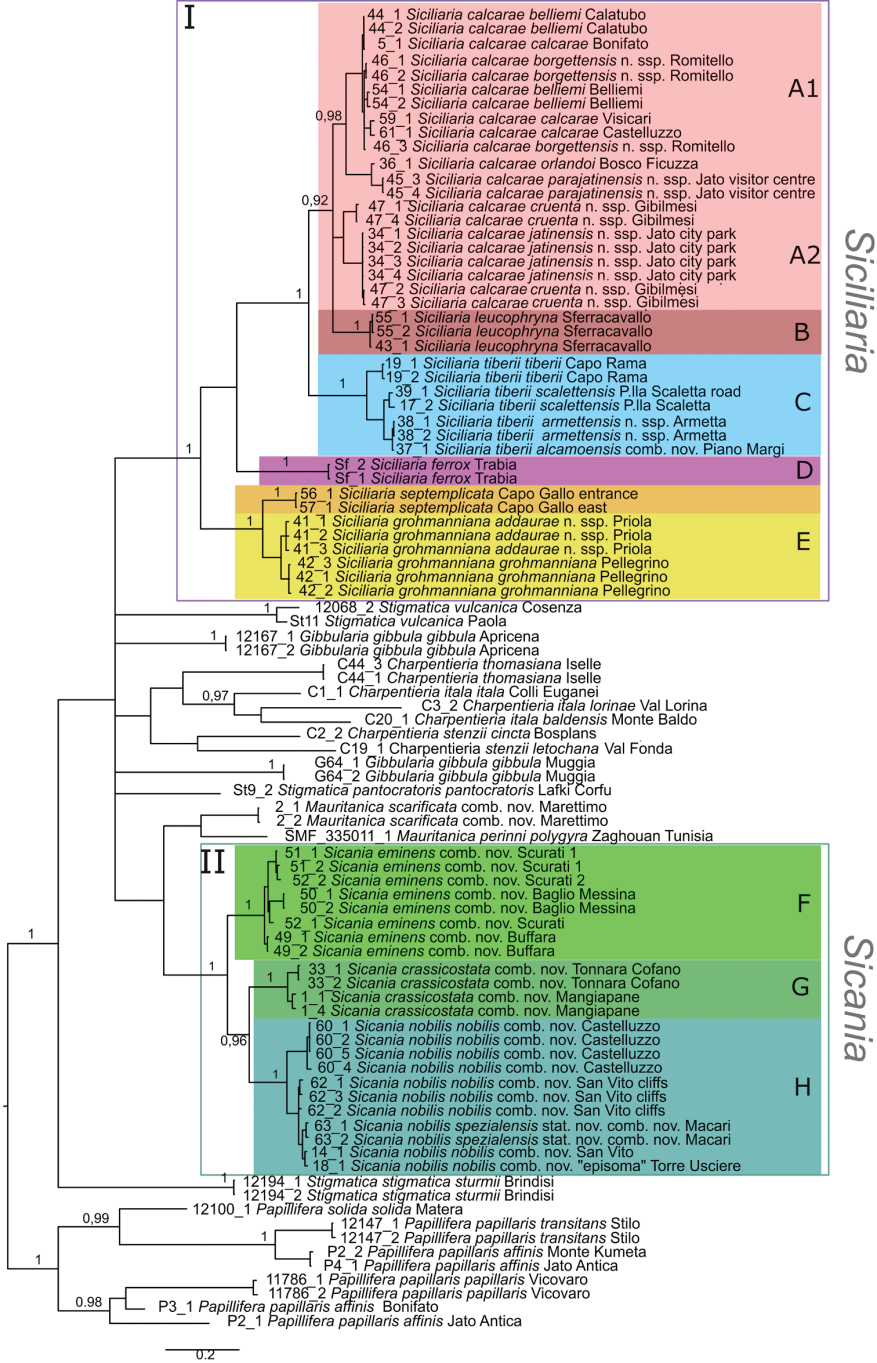
BI tree of based on the COI data set of *Siciliaria*, *Sicania* and related genera. BI posterior probability values (> 0.90) of major nodes are indicated.

The collection numbers, voucher numbers, GenBank accession numbers and geographical coordinates for the specimens used in the molecular genetic analyses are listed in Table 1. *Papillifera* was used as outgroup for the phylogenetic trees calculated from the three data sets (COI, ITS2, combined). BI and NJ analyses yielded the same overall topologies (not shown). The BI tree based on COI sequences comprising 92 individuals is shown in Figure 4. In this tree, the species from northwestern Sicily so far comprising the genus *Siciliaria* were found within two separate highly supported main clades. Albeit the two clades (I and II) emerge as sister groups in the combined tree, this grouping is not well supported, and it remains unclear whether *Siciliaria* and *Sicania* together form a monophylum. There is only one exception, *Siciliariascarificata* (in the tree designated as *Mauritanicascarificata* comb. nov.), which forms the sister group of *Mauritanicaperinnipolygyra*. This branch is the sister clade of clade II, although this relationship is very weakly supported. The mean p distance between clades I and II was 19.6% (range 18.9–21.6%). The two clades correspond to the division into the two genera *Siciliaria* and *Sicania* as proposed by Boettger (1877).

The taxa comprising the two main clades I and II cluster in a geographic pattern (maps in Fig. 1E, F). Clade I includes species that are found in limited areas in the eastern part of the distribution area, plus the widely distributed *Siciliariacalcarae*. The latter is found in several occurrences in the southern parts of the distribution range from west to east. Along the eastern part of its distribution area, the nominate subspecies of *S.calcarae* is sometimes replaced by local costate forms. Clade II comprises the western species distributed in a comparatively small area from San Vito Lo Capo Peninsula, Monte Cofano to Cornino-Custonaci area. Within the main clades, the various (generally highly supported) subclades are mostly consistent with taxa and reflect their distribution. Specimens from the same locality delivered very similar or identical sequences. Intraspecific distances (summarised in Table 3) within both *Siciliaria* and *Sicania* were moderately low mean distances up to 7.0% with the highest values found in *S.calcarae* (7.0%) and *S.grohmanniana* (5.1%). Besides the main clades I and II, the tree contains several quite distinct lineages representing other taxa [belonging to S*tigmatica*, *Charpentieria*, as well as *Gibbularia*, which are separated by high p distances from each other (mean distances ranging from 18.6–23.8%), as well as from clades I and II (18.3–23.1%). In this tree, the genera S*tigmatica* and *Gibbularia* are not monophyletic. *Charpentieria* forms one clade but the support values are very low. The relationships between the two main clades and the other genera remain unresolved and appear as a polytomy in the tree. The low resolution in the deeper nodes is not surprising given the high distances, as substitutional saturation might blur the relationships among lineages. The average p distance between the outgroup *Papillifera* and the ingroup sequences was 24.6% (range of means between groups 23.8–25.6%). Even within some genera remarkably high distances were found (up to 25.8% *Papillifera*, 22.6% *Charpentieriaitala*, 18% *Charpentieriastenzii*), while distances within *Siciliaria* and *Sicania* were lower (mean p distance 13.4 and 11.4%, respectively) (see Table 2).

**Table 2.** Mean genetic distances (p-distances in %) for the COI dataset between the genera and/or lineages analysed. Mean genetic distances within the genera *Siciliaria* and *Sicania* are in bold. *Gibbularia* 1 = *Gibbulariagibbulagibbula* from Apricena; *Gibbularia* 2 = *Gibbulariagibbulagibbula* from Muggia.

		**1**	**2**	**3**	**4**	**5**	**6**	**7**	**8**	**9**	**10**
**1**	*Siciliaria*	13.4									
**2**	*Sicania*	19.6	11.4								
**3**	*Mauritanica*	20.5	17.0								
**4**	*Stigmaticapantocratis*	21.5	18.3	17.1							
**5**	*Stigmaticavulcanica*	21.3	21.3	20.7	18.6						
**6**	*Stigmaticasturmii*	22.1	19.9	19.8	19.5	22.4					
**7**	*Charpentieriaitala*	21.7	21.4	20.8	20.7	22.5	21.9				
**8**	*Charpentieriastenzi*	21.3	21.8	20.2	18.6	21.0	21.9	20.7			
**9**	*Gibbularia* 1	21.0	21.4	19.4	19.4	20.7	22.8	22.3	20.1		
**10**	*Gibbularia* 2	23.1	23.1	22.2	20.7	22.4	23.8	22.3	21.9	20.5	
**11**	*Papillifera*	24.5	24. 5	24.0	24.1	25.7	23.9	25.6	24.4	23.8	25.3

**Table 3.** Intra- and interspecific mean genetic distances (p-distances in %) among the main clades of *Siciliaria* and *Sicania* calculated from the COI sequences. Intraspecific mean distances are in bold.

		**1**	**2**	**3**	**4**	**5**	**6**	**7**	**8**
**1**	*Siciliariacalcarae*	**7.0**							
**2**	*Siciliarialeucophryna*	11.3	**0.4**						
**3**	*Siciliariatiberii*	14.3	14.4	**0.5**					
**4**	*Siciliariaferrox*	17.0	16.6	17.8	**0.4**				
**5**	*Siciliariaseptemplicata / grohmanniana*	16.9	17.3	17.3	16.4	**5.1**			
**6**	*Sicaniaeminens*	20.8	21.3	20.8	20.8	19.7	**2.8**		
**7**	*Sicaniacrassicostata*	21.4	20.3	21.3	20.2	20.2	12.2	**2.5**	
**8**	*Sicanianobilis*	20.9	20.5	21.6	19.7	18.9	13.3	12.5	**4.3**

**Figure 5.**
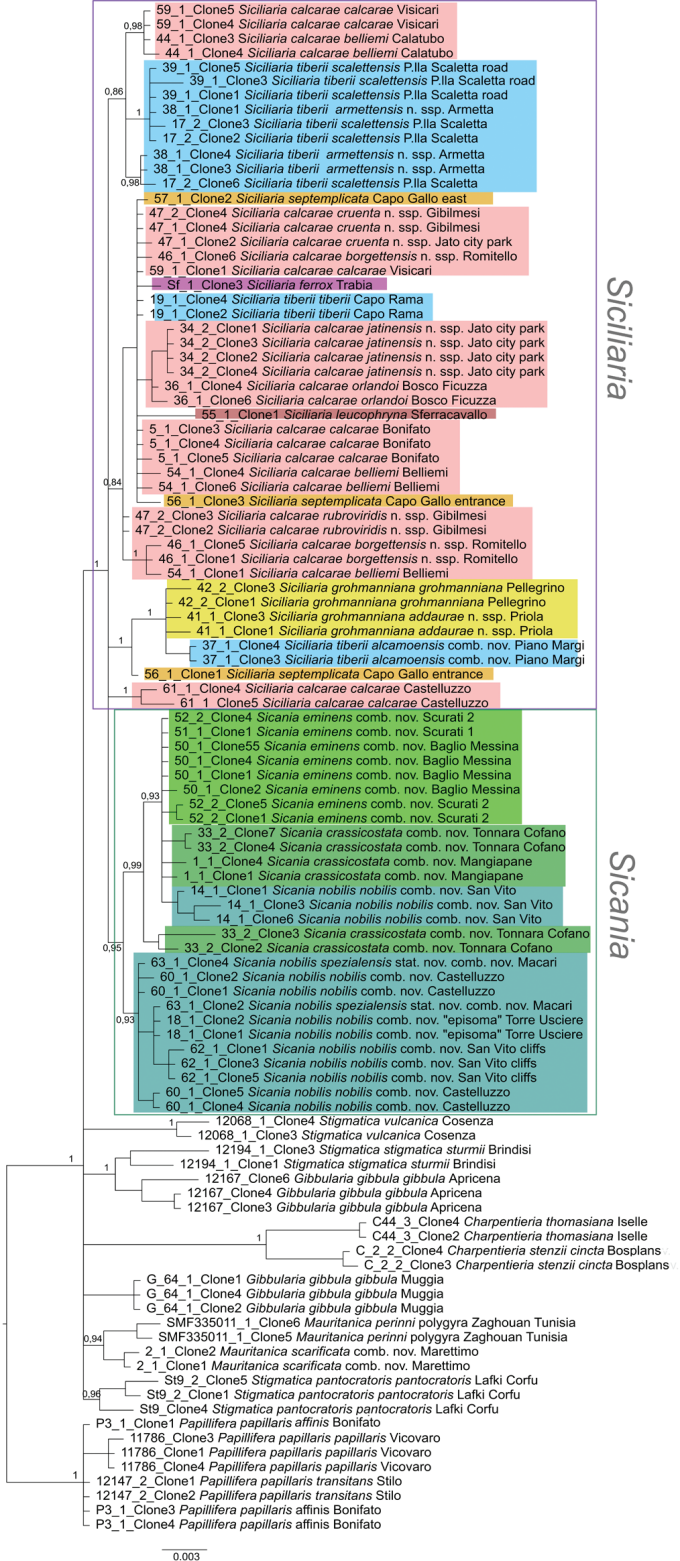
BI tree of based on the ITS2 data set of *Siciliaria*, *Sicania* and related genera. Colours of *Siciliaria* and *Sicania* clades correspond to those in the distribution maps (Figs 1E, F). BI posterior probability values (> 0.90) of major nodes are indicated.

The ITS2 sequences were obtained from 42 individuals (Table 1) and comprised altogether 106 sequences (due to up to five cloned sequences per individual; see Materials and methods). The BI tree (Fig. 5) shows all sequence variants isolated from those individuals. The variation among ITS2 copies is due to the fact that for some individuals, more than one clone was sequenced (due to the genomic arrangement of the rRNA genes in repeats of clustered units, more than two alleles can be expected). Generally, small distances were found among ITS2 sequences, resulting in a low resolution in the ITS2 tree. Most branches did not obtain sufficient support, but the tree reflects to some extent the COI tree. The western taxa (corresponding to main clade II, *Sicania*) appear as separated group including no representatives of main clade I (*Siciliaria*). One subgroup contains only *S.nobilis* and the other subgroup contains *S.crassicostata*, *S.eminens*, and a few sequences of S. *nobilis* (from San Vito). Various clones of *S.crassicostata* differed slightly and for the specimen 33, clones 2 and 3 appeared in a subclade separate from the remaining sequences of *S.crassicostata* (including those from the same individual). The taxa corresponding to main clade I are found within several mixed clades/haplogroups with no apparent taxonomic or geographic pattern. Sequences from specimens of the same population cluster together with low distances. Sequences of the other genera appear as various separate branches. *Stigmatica* and *Gibbularia* are not monophyletic.

**Figure 6.**
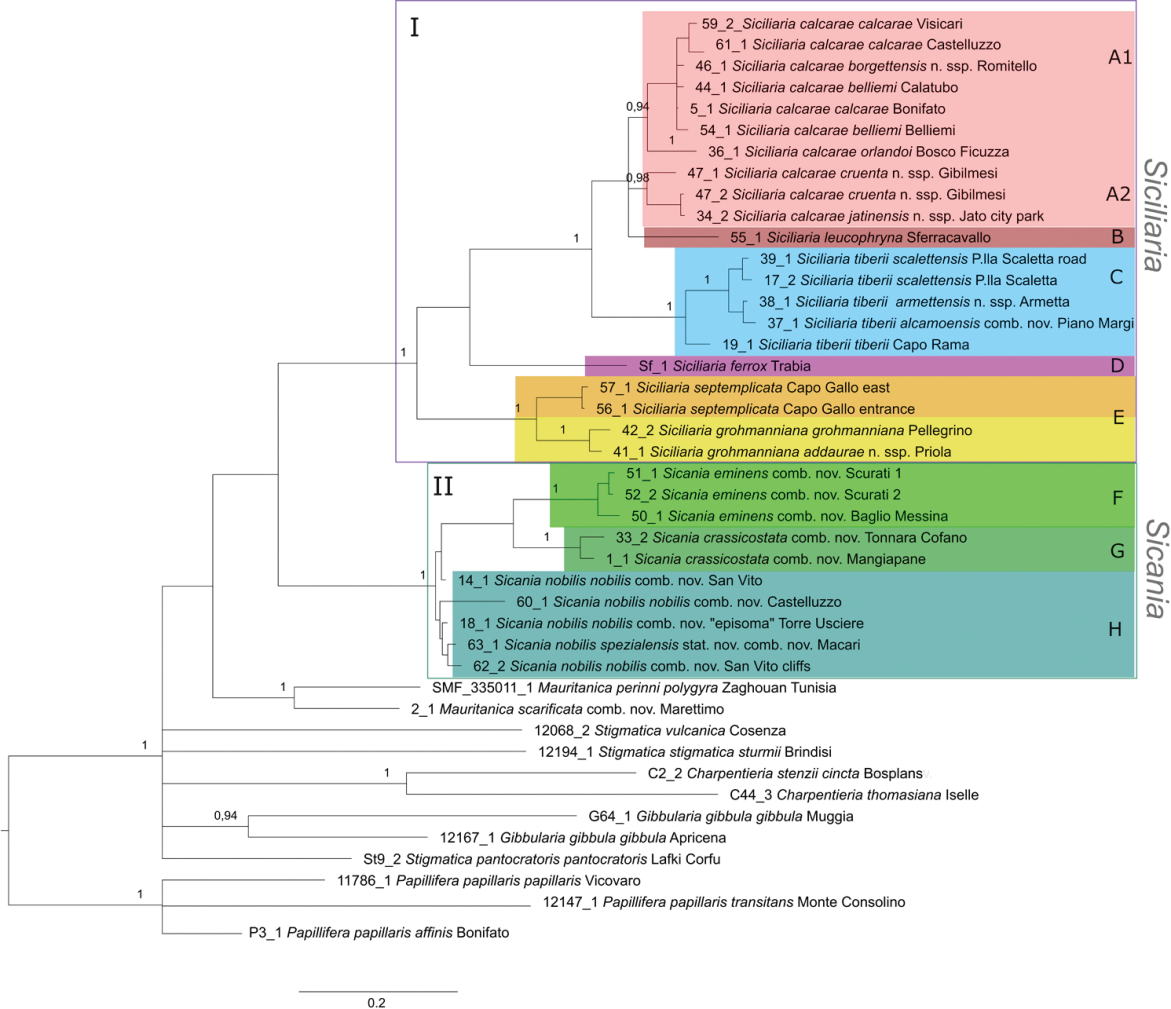
BI tree of based on the concatenated COI and ITS2 data set of *Siciliaria*, *Sicania* and related genera. Colours of *Siciliaria* and *Sicania* clades correspond to those in the distribution maps (Fig. 1E, F). BI posterior probability values (> 0.90) of major nodes are indicated.

The tree based on the combined data set (COI plus ITS2; 45 individuals) is shown in Fig. 6. It has the same overall topology as the COI tree, but with high support of several nodes, but fewer individuals represented. The apparent sister group relationship between clades I and II received no considerable support and thus, the relationship between the two clades remains open. The species are monophyletic and most of them well supported. Concerning the other genera, it should be mentioned that this tree provides less information as the number of individuals is lower than in the COI tree. As in the COI tree, *C.stenzii* and *C.itala/dyodon* cluster, highly supported, but in the combined tree of this clade only *C.stenzi* and *C.dyodon* were included. *Mauritanica* (including the former *S.scarificata*) is highly supported as well.

Specimens from the same locality delivered very similar or identical sequences. Intraspecific distances (summarised in Table 3) within both clades I and II (i.e., *Siciliaria* and *Sicania*) show moderately low mean distances, up to 7.0% with the highest values found in *S.calcarae* (7.0%) and *S.grohmanniana* (5.1%). With one exception, all species were highly supported in the COI tree and in the combined tree. Within *Siciliaria*/*Sicania* only *S.calcarae* was found in two subclades, which formed together with *Siciliarialeucophryna* an unresolved trichotomy within one clade. In some cases, subspecies formed slightly separated lineages (e.g., *Siciliariatiberiitiberii*), while generally this was not the case.

### ﻿Phylogeography: distribution and phylogeny of *Siciliaria*/*Sicania* with analysis of the sympatries among taxa

The taxa comprising the two main clades I (*Siciliaria*) and II (*Sicania*) cluster in a geographic pattern (maps in Fig. 1E, F). Most taxa show well defined and delimited distributions, with some exceptions (of both *Siciliaria* and *Sicania*). The widely distributed *Siciliariacalcarae* has a partially overlapping distribution with either *Sicanian.nobilis* or *Sicaniaeminens*. Moreover, along the eastern part of the distribution area, the nominate subspecies of *Siciliariacalcarae* is locally replaced by other subspecies that differ by virtue of the costate shells or details of the clausiliar apparatus. The distribution records of *Sicaniacrassicostata* and *Sicaniaeminens* overlap in a small area near Contrada Scurati/Grotta Mangiapane, where the two species are, hardly distinguishable by their shell but show differences in the morphology of the genital organs and the genetic results (see *Sicaniaeminens* comb. nov.).

*Siciliarialeucophryna* shows a narrow distribution, limited to Isola delle Femmine, Pizzo Mollica, and Pizzo Manolfo. *Siciliariatiberii* is restricted to the calcareous massif of Monte Palmeto, Pizzo di Mezzo, Monte Pecoraro and Montagna Longa and its northwestern coastline. *Siciliariaferrox* occurs in the Trabia-S. Onofrio area. The closely related *Siciliariagrohmanniana* and *Siciliariaseptemplicata* are indeed geographically close, the former occurring exclusively along the southern slopes of Monte Gallo and on Monte Pellegrino and the latter found along the northern slopes of Monte Gallo and scattered localities south of Palermo (Nordsieck 2013b). Syntopy seems to be very rare among *Siciliaria*/*Sicania* taxa. To our current knowledge, *Siciliariacalcarae* is sympatric with *Siciliariaseptemplicata* (south and west of Palermo area), with *Siciliariagrohmanniana* (Monte Pellegrino), with *Sicaniaeminens* (Custonaci and Castellammare del Golfo Area) and with *Sicanianobilis* (San Vito lo Capo area). The sympatry of *Siciliariacalcarae* and *Sicaniacrassicostata* from Favignana Island seems to be due to antropochorous dispersal of the latter taxon. The southern part of the *Siciliaria*/*Sicania* distribution area so far seems to be occupied only by *Siciliariacalcarae* as also supported by the molecular genetic results. In one locality only (Castelluzzo) *Siciliariacalcarae* and *Sicanianobilis* were found together at the same spot: shady limestone walls in a *Quercusilex* shrub. Nonetheless, a clear ecological niche separation of these two taxa syntopic at this locality was detected. The actual and detailed distribution ranges of the *Siciliaria* taxa still have to be clarified. Moreover, further field research must still verify undetermined or uncertain old literature citations, and thoroughly investigate a diverse and heterogenous territory with an amazing endemism rate.

### ﻿Autecology: *Siciliaria* and *Sicania* taxa are not obligate rock-dwelling snails

*Siciliaria*/*Sicania* taxa show a remarkable adaptation and capability to colonise not only dry, open and exposed limestone walls and cliffs but also a huge variety of habitats and ecological niches. The most stenoecious species are, in order, *Sicanianobilis*, *Siciliariaferrox*, and *Siciliariaseptemplicata*, which are found exclusively on limestone walls or limestone boulders. *Sicaniacrassicostata* is found on limestone walls or under rocks. More euryoecious habits were recorded for *Siciliariagrohmannianagrohmanniana*, which is found on exposed limestone walls hiding in cracks and under crags. It is also found in *Pinus* sp. forest on decaying woods and tree trunks, including shady and humid spots on mosses and ferns. Interestingly, its subspecies *Siciliariagrohmannianaaddaurae* ssp. nov. is found exclusively on limestone walls. *Siciliarialeucophryna* and *Sicaniaeminens* colonise limestone cliffs hiding in cracks or under stones. They are very common on isolated boulders and tree trunks and barks in the xerophilic scrub. The most euryoecious species is *Siciliariacalcarae*, which colonises all the habitats until now described but also is commonly found in anthropogenic environments as stone walls, road walls, ruins and disturbed habitats.

### ﻿External morphology of the genital organs

The high number of *Siciliaria*/*Sicania* specimens dissected during this study (n = 120) provided an accurate overall description of the genital organs of these genera.

No seasonal variations were detected concerning shape or internal sculpturing of genital organs. The only clear distinction was between adult specimens with full-developed genital organs and immature, subadult specimens with extremely reduced, vestigial genital organs.

The general external morphology of the genital organs is the same in all taxa of *Siciliaria*/*Sicania*. The relative dimensions (ratio between the shell height and the length of the penial complex H_s_/PCL×100) do not substantially differ among taxa, ranging from 25% to 35%, revealing a great dimension stability of genital organs in relationship to the shell dimensions. The genus *Montenegrina* (De Mattia et al. 2020) showed a higher variability, ranging from 13.9% to 87.6%. The overall appearance of the genital organs of *Siciliaria*/*Sicania* is always balanced [“mesomorphic” as defined in De Mattia et al. (2020)], without showing the extreme variability found in *Montenegrina* where extremely elongated and thin or short and bulky genital organs were found.

The hermaphroditic gland (HG) has many consolidated acini and it is found along the two apical whorls of the animal’s body. The first hermaphroditic duct (FHD) is very thin along its proximal portion but abruptly turns into a highly coiled, skin-like structure. The diameter of the FHD is much larger along the coiled part, and the number of whorls ranges from six to eleven. The distal uncoiled part of the FHD is very thin, straight and ends directly in the fertilisation chamber-spermatheca complex. The albumen gland (AG) is slender. The general scheme of the FHD is stable among all the *Siciliaria* taxa. The AG is very variable in size compared to the ovispermiduct (OS). In full-grown adult specimens, it can be larger or as long as the OS or barely reaching ^1^/_4_ of its length. The OS consists of a female and a male part including the seminal groove, which is not visible from outside. Externally, it is irregularly spaced by smooth constrictions and it is whitish to cream in colour. The male, prostatic part consists of the visible prostatic gland (PG) and the sperm groove (SG), light to dark grey in colour and clearly distinguishable from the lighter female part. The overall size of the OS is very stable among the *Siciliaria/Sicania* taxa. The free oviduct (FO) is usually thin and long and its length is stable in relation to the length of the vagina (V), and its average ratio (FO/V) is ~ 1. The V is simple, usually cylindrical, without any appendix or caecum. The vaginal retractor (VR) is strong; it is always present and attached to the second duct of the bursa copulatrix (SDBC). It was not depicted in the anatomical drawing due to its low taxonomic value. The V is stable in length in relation both to shell height and penis length. The ratio between the length of the penial complex (PC) and the length of the V (PC/V) is also stable, ranging from two to three. The BC+SDBC complex (CBC) consists of the FDBC, a second duct (SDBC), a well-developed D (diverticulum) and the actual BC+SDBC. The D is always longer than the SDBC+BC. The transitional section between the BC and the SDBC can range from clearly visible to completely missing (with all degrees of transitional states). The PC comprises the penis (P) and the epiphallus (E). The transition between P and E does not always show the epiphallar thickening (ET), as stated in Nordsieck (2013b). More than 50% of the dissected specimens did not present the ET. The presence of the ET is not species-specific as it is randomly present or missing also in specimens of the same population of one given taxon. The flagellum is absent. The retractor muscle (RM) is single and undivided and usually strong. The P is usually uniformly cylindrical or slightly swollen proximally, without caecum or penial appendix. The E is usually as long as the P. It can be cylindrical to thin and spindly. The transitional area between the E and the distal portion of the vas deferens (VD) is usually smooth or barely visible. The atrium (A) is usually large and short.

### ﻿Internal morphology of the genital organs

In contrast to the external morphology of the genital organs, the internal sculpturing differs between the various taxa of *Siciliaria* and *Sicania*, but is stable within the taxa, with only minor population/individual variability. The penis shows the highest variability, which involves the combination (or the absence) of pleats arranged in a longitudinal, transversal, or oblique direction or creating irregular patterns. The epiphallus shows different types of sculpturing, with longitudinal pleats or without any structure. The internal transition between the epiphallus and the vas deferens is very clear due to the clear change of the sculpturing. The structure and the variability of the penial pseudopapilla and the morphology of the internal transition between penis and epiphallus will be discussed in detail in the following. The atrium can exhibit different configurations, from completely smooth to variable pleats. In contrast to what was found in *Montenegrina* (De Mattia et al. 2020), a large atrial fold has never been detected. The internal V shows few different configurations. Similar to the penis, its variability lies in the combination (or absence) of pleats arranged either in a longitudinal, transversal or oblique direction.

Two specimens were found with the spermatophore intact and embedded into the diverticulum of the bursa copulatrix complex or in the vagina and partially inside the first duct of the complex of the bursa copulatrix. The spermatophore of *Siciliariacalcaraecalcarae* (Philippi, 1844) and *Mauritanicaperinnipolygyra* (O. Boettger, 1879) are described for the first time. The spermatophores are slender and slightly longer than the diverticulum. The overall shape is slightly to markedly bent. These preliminary observations revealed a difference between *Siciliaria* with both upper and lower keel and *Mauritanica* with only the upper keel.

All the genera *Siciliaria*, *Sicania*, *Mauritanica*, *Stigmatica*, *Gibbularia*, and *Papillifera* exhibit a pseudopapilla. The pseudopapilla (PP) is a “false” penial papilla, found at the transition between the penis and the epiphallus, slightly distally to the insertion of the retractor muscle. The pseudopapilla, conversely to the actual penial papilla [as in the genus *Montenegrina*, depicted in De Mattia et al. (2020)], originates from one side of the penial wall and totally lacks a penial channel. Its lumen is filled with loose spongy tissue (Fig. 3.6). The shape and dimensions of the pseudopapilla are extremely variable, from conical and blunt to elongated and cylindrical. Sometimes it is extremely reduced or totally missing. Presumably the absence of the pseudopapilla represents the apomorphic state. The base of the pseudopapilla can be simple, thus it directly originates from the penial wall. Otherwise, the pseudopapilla can originate from a fleshy structure emerging from the penial wall. This structure is circular, fleshy, ring-like, usually smooth and more or less robust. We name this genital structure “epiphallar ring” (ER). The ER can be missing or up to three. When detectable from outside, the transition between penis and epiphallus presents a more or less sudden narrowing of the proximal penis, distally to the base of the retractor muscle and corresponding to the area where the pseudopapilla originates. The genital portion immediately distal to the narrowing could be seen as a thickening and could represent what Nordsieck (2013a) introduced as the ”epiphallar thickening“ (ET).

In *Siciliaria*/*Sicania* one to three strong epiphallar rings are always present (only one being the origin of the pseudopapilla). *Charpentieria* and *Stigmatica* present only one epiphallar ring which is the origin of the pseudopapilla. In *Gibbularia* the epiphallar ring was found only in Gibbulariagibbulacf.sanctangeli (Fig. 56.6). *Papillifera* never shows an epiphallar ring and the pseudopapilla is either isolated (thus directly originating from the wall) or directly originating from the distal end of one epiphallar longitudinal pleat.

In the Southern Alpine *Charpentieria* the genital investigations revealed two undescribed structures of the transition between P and E: in *C.dyodon* s. l. it is named “parapseudopapilla” (PPP) and in *C.stenzii* s. l. it is named “hemipapilla” (HP) and are described in the following sections. In order to describe and classify the huge variety of PP-ER-ELP combinations found in all the taxa, we created a pseudopapillar formula. This formula will be provided for each taxon and population dissected, in order to highlight the extreme variable situation of this part of the genital organs. In this formula the parentheses () mean direct connection (continuity/contiguity of anatomical traits), whereas the plus + means interruption of the anatomical continuity. For example, the formula 1ER(PP)+2ER(ELP) means that PP originates from the distal (first) ER and the proximal ER (second ER) is directly connected with the ELP.

### ﻿Diagnoses of the shell and distal genital organs of the genera *Siciliaria* and *Sicania*

**Shell.** Shell elongated, fusiform, sinistral, can be either decollate or non-decollate, with or without well-developed white surface layer. Apex subacute when present, whitish to dark brick red in colour. Protoconch smooth. Spire slowly and regularly growing with 9.5–11.5 whorls. Umbilicus closed, sutures shallow. Aperture subovoid to subquadrangular. Peristome continues to be interrupted. Average shell height 19.9 ± 2.1 mm (n = 630, decollate and not decollate shells), average last whorl width 4.5 ± 0.4 mm (n = 630), average aperture height 4.6 ± 0.5 mm (n = 630), average aperture width 2.9 ± 0.4 mm (n = 630). Whorls weakly rib-striated, rib-striated, ribbed to percostate without or with evident to barely recognisable sutural papillae. Dorsal keel missing, mostly indistinct to prominent. Lunella always present, dorsal to dorso-lateral, more or less developed. Inferior lamella low, moderately high, high to very high. Missing, one or two anterior upper palatal plica; when two are present: upper one mostly separated from upper palatal plica; when one is present: separated from or connected with upper posterior palatal plica. Rarely, lower anterior upper palatal plica is present, separated from or connected with upper palatal plica. ALPP tending to be shortened to almost missing. Spiralis overlapping or not overlapping parietalis. Posterior lower palatal plica missing more or less reduced. Palatal edge of clausilium plate distally receding, slightly or not receding. Clausilium palatal edge against distal end not bent upwards, or bent upwards to strongly bent upwards.

**External distal part of the genital organs**. Free oviduct slightly shorter to remarkably longer than the vagina. Vas deferens thin. First duct of the bursa copulatrix slightly shorter to much longer than second duct of bursa copulatrix + the actual bursa. Distinction between the actual bursa and second duct of bursa copulatrix clear to missing. Diverticulum of the bursa copulatrix longer to much longer than the vagina and second duct of bursa copulatrix + the actual bursa. The vagina is long or short and the atrium usually large. Penial complex always longer to much longer than the vagina. Retractor muscle short to long, usually robust. Epiphallar thickening present or missing. Epiphallus slightly shorter to much longer than the penis. Transition between epiphallus and vas deferens clearly visible to totally indistinguishable.

**Internal distal part of the genital organs**. Vagina and penis with internal sculpturing comprising a combination (or also absence) of pleats of different size and texture, arranged in a longitudinal, transversal or oblique direction or creating particular net-like pattern. Atrium simple or pleated. Pseudopapilla originating from epiphallar wall or epiphallar ring, elongated to roundish, with smooth or coarse surface. Epiphallar rings up to three to completely missing. Proximal epiphallar ring connected or not with the epiphallar pleats. Epiphallus with or without longitudinal pleats, both smooth or fringed. Epiphallar walls almost smooth, finely granulated of with small transverse papillae.

## ﻿General discussion

The alpha-taxonomy of *Siciliaria*/*Sicania* was based on shell morphology. However, the variability of the shell characters reflects the complicate nomenclatural history (Nordsieck 2013b). After a brief general discussion, the Taxonomic part will provide in-depth discussion of the results regarding taxonomy and taxonomic decisions.

The external shape of the genital organs was found to be of low taxonomic value, because no particular configuration is exclusive and distinctive of one taxon or a group of taxa. On the other side, the internal sculpturing is highly variable, but stable at the population level and the morphology of the genital organs delimits (sub)species, similar to what was found in the genus *Montenegrina* (De Mattia et al. 2020). Different (sub)species can share a similar morphology of one or more sections of the genital organs (atrium, penis, epiphallus, penial papilla, distal vagina, proximal vagina), but no taxon, both at specific and subspecific level, was found to show an identical overall anatomical arrangement with another one. Even closely related taxa differ in their anatomy of the genital organs (e.g., *Siciliariacalcarae* complex). Isolation plus time (i.e., drift + microenvironmental conditions) may be the “engine” of morphological diversification (anatomy of the genital organs, shell) in *Siciliaria* and *Sicania* (and also in related genera, e.g., *Stigmatica* and *Charpentieria*). Furthermore, preliminary results indicated that even in *Papillifera*, the morphology of the genital organs, which was thought to be “stable”, shows a much higher diversity (see Fig. 65). Nevertheless, homoplasies in parts of the anatomy of the genital organs may be expected. It is worth stressing that despite the high anatomical variability found, no diagnostic traits of the external or internal genital organs were found distinguishing *Siciliaria* from *Sicania*. Furthermore, our anatomical investigation proved that morphological characters proposed by Nordsieck (2013a: 4), to differentiate *Siciliaria* (sensu MolluscaBase 2021) from other genus-groups (*Charpentieria*, *Stigmatica*, *Gibbularia*, and *Papillifera*), namely the presence/absence of the ET and the delimitation of the proximal part of the penis (may it be ”distinct” or ”indistinct”) revealed to be not valid. Character combinations, both external and internal, were not found exclusively in any given (sub)genus.

Nordsieck (2013b) proposed *Mauritanica* as a subgenus of *Siciliaria* Vest, 1867 based on the preliminary molecular data of Scheel and Hausdorf (2012) and Uit de Weerd and Gittenberger (2013) and on some common morphological characters of the genital organs, such as the presence of a thickening in the passage (ET) from the penis to the epiphallus (missing in *Charpentieria*) and for an indistinct delimitation between proximal and distal penis (distinct in *Charpentieria*). Our genital investigations did not confirm the observations of Nordsieck, namely that the epiphallar thickening (ET) is always missing in *Charpentieria* and always present in *Siciliaria*. The external shape of the transition between penis and epiphallus is highly variable in *Siciliaria*/*Sicania* and *Charpentieria*, in taxa of both genera the ET may be present or missing. The characters presented by Nordsieck as the ”indistinct delimitation between proximal and distal penis (distinct in *Charpentieria*)” was also not verified in the present genital investigations, which is probably due to the lower sample size analysed by Nordsieck (1963a; 1963b).

### ﻿Species delimitation in *Siciliaria* and *Sicania*

The principal rationale of our integrative strategy was whether phylogenetic relationships confirm morphogroups found in a specific area and whether or not they correspond with current taxonomy. We deliberately refrained from applying automated species delimitation methods, for which larger sample sizes would have been necessary. Furthermore, in various land snail species as it was shown such methods may deliver discordant results (e.g., Prevot et al. 2013; Modica et al. 2016; Batomalaque et al. 2019; Mason et al. 2020a), there may be a lot of factors hampering straightforward delimitation solely based on genetic clades and the concept of the DNA barcoding gap (Meier et. al. 2008). First, intra specific distances were found to vary considerably among various land snail species and are sometimes higher than commonly reported interspecific distances (Davison et al. 2009). Further examples for high intraspecific distances are the clausiliid genera *Montenegrina* (Mason et al. 2020a) and *Clausilia* (Mason et al. 2020b), the genus *Schileykula* (Harl et al. 2019) and the genetically highly diverse hairy snail *Trochulushispidus* where even budding speciation was observed (Kruckenhauser et al. 2011).

So far, mostly mitochondrial marker sequences were used successfully in phylogenetic studies of land snails. The quite frequently used nuclear encoded histone genes H3 and H4 (including the spacer region in-between) often did not add much information or even resulted in confusing trees, probably due to multiple paralogous copies of these genes that escaped homogenisation and/or recombination events after hybridisation (e.g., Harl et al. 2019; Mason et al. 2020a). Indication for interspecific hybridisation, even (at least sporadically) over wide phylogenetic distances were reported for several species, e.g., in the clausiliid genus *Montenegrina* (Mason et al. 2020a). Further examples were found e.g., in the bradybaenid land snail genera *Ainohelix* and *Ezohelix* (Morii et al. 2015) or the orculid genus *Schileykula* (Harl et al. 2019). Likewise, the internal transcribed spacer ITS2 used in the present study did not yield a well resolved tree and showed too low variation to unambiguously elucidate the phylogenetic relationships within *Siciliaria* and *Sicania*. Anyhow, several mt lineages were confirmed in the ITS2 tree. The ITS2 marker sequence showed some problems caused by paralogous copies as indicated by the various positions the sequences from single individuals took in the tree. Based on the presently available data it cannot be decided whether this is solely due to incomplete lineage sorting. Intraspecific hybridisation would result in similar patterns and there are some cases where this explanation appears likely (e.g., *alcamoensis*; see Taxonomic part).

In summary, in the COI tree the species were monophyletic except the widely distributed *S.calcarae*. Clustering of subspecies is mentioned in the taxonomic part, albeit this aspect is of minor relevance, (1) because subspecies would not necessarily be expected to be monophyletic and (2) because they were represented by too small samples.

### ﻿Unidentified taxa, enigmatic species and the hybridisation concept within *Siciliaria* and *Sicania*

Potiez and Michaud (1838: 181–182) introduced *Clausiliadecollata* and *Clausiliadeshayesii* with a broad distribution indication: ”Hab. La Sicilie“. These names probably belong to *Siciliaria*/*Sicania*, but their specific status was not yet clarified. Benoit (1876: 151; 1881: 106) mentioned *Clausiliadeshayesii*, providing a precise distribution (“vive nelle campagne di Palermo; l’abbiamo pure ricevuta dai dintorni di Prizzi”). As synonyms, Benoit (1876) listed *Clausiliacalcarae*, *Clausiliaassimilis* (= ?) and *Clausiliacastanea* (= ?) and Benoit (1881) listed *Clausiliacalcarae*, *Clausiliaseptemplicata* and *Clausiliacastanea* (= ?) thus, as already suggested by Nordsieck (2020: 6) this name could be regarded as a junior synonym of *Siciliariacalcarae* or *Siciliariaseptemplicata*. As a matter of fact, both taxa are known to occur in the surroundings of Palermo. On the other hand, Prizzi is currently considered remarkably outside of the known distribution of *Siciliaria*/*Sicania*, and further research in this area is needed in order to clearly assess the identity of this name. Its synonymy with *Clausiliacalcarae* and *Clausiliaseptemplicata* can only be speculated until additional data are available. Benoit (1876, 1881) introduced three taxa that currently are unidentified. The first is *Clausiliatrinacrina* (Benoit 1876: 151) or also cited as *Clausiliatrinaclina* (Benoit 1881: 105), erroneously cited by Nordsieck (2013b: 4) as *C.trinacliana* that he arbitrarily considered as a misprint of *trinacriana*. The distribution of this taxon (Benoit 1881: 106) is the area surrounding Palermo and Calatafimi. De Stefani (1895: 186) cited the species with its proper name, which is *Clausiliatrinacrina*, as he received the correct name spelling directly from Benoit: “nel detto catalogo (Benoit 1881) Benoit la chiama *trinaclina*, ciò per errore di stampa come risulta dalla correzione a penna fatta da lui stessa nella copia donatami”. Moreover, De Stefani (1895: 187) provided a precise locality for the species: Monte Cuccio near Palermo. The differential diagnosis provided by Benoit and Boettger’s opinion (Boettger 1881: 106) makes the synonymy with *Siciliariacalcare* very plausible. Benoit (1876: 151) cited *Clausiliapanormitana* from Monte Cuccio, east of Palermo as a synonym of *Siciliariagrohmanniana*. Our field research confirmed that *Siciliariagrohmanniana* is not present on Monte Cuccio and its surrounding peaks. For *Clausiliatrinacrina*, the synonymy with *Siciliariacalcare* is very plausible.

Finally, Benoit (1876: 152) cited *Clausilialaudabilis* from San Fratello. This locality is probably and old toponym from the surroundings of Bagheria (Palermo) as San Fratello near Messina is totally out of the distribution range of *Siciliaria*/*Sicania*. *Clausilialaudabilis* is considered by Nordsieck (2013b: 7) as a synonym of *Siciliarialeucophryna* despite the locality provided by Benoit doesn’t match with the current known distribution of this species. Thus, both the exact position of the locality and the identity of *Clausilialaudabilis* remain to be assessed.

Benoit (1881: 117) introduced also *Clausiliarubra* from San Fratello, and San Fratello near Messina cannot be excluded in this case. *Clausiliarubra* Benoit, 1881 could be referred to *Stigmaticaincerta* (Küster, 1861), which is known to occur from the area (WDM personal data). Despite Boettger’s opinion that considered *Clausiliarubra* as a form of *Siciliariaseptemplicata*, Benoit (1881: 117) disagreed with his opinion, by virtue of a differential diagnosis that highlights important differences. The given locality is found remarkably outside of the currently known distribution of both, *Siciliaria* and *Sicania* (Nordsieck 2013b: 2). More field and taxonomical research are needed in order to properly assess the status of this species and, at the moment, no synonymy is conceivable.

*Clausiliasubdiaphana* was introduced by Benoit (1881: 118), who stated that this taxon is conchologically similar to *Siciliariagrohmanniana* but differs by a different arrangement of the lamelle and plicae and by a very fragile, not decollate shell. The collecting locality is ”montagna Buon Riposo presso Palermo”. This old toponym is currently almost forgotten, but Scinà (1818: 51, 57) identified this hill at the western foot of Monte Cuccio, east of Palermo. Yet, the lithological substratum of that hill and its surroundings is composed by quartzite, gres, and sandstones, thus an unusual habitat for *Siciliaria/Sicania*. The fragile shell could represent an adaptation to a soil extremely poor in limestone contents. Monterosato (1894: 170) assumed the snails from Monte Pellegrino to be ”Var. ex forma: *subdiaphana*, Ben.”, a variety of *Siciliariagrohmanniana*. Monterosato neither justified the synonymy, nor stated that he actually found Benoit’s taxon at Monte Pellegrino. At the moment, no synonymy is conceivable.

The status of *Siciliariariberothi*, introduced by Brandt (1961: 8 as *Siciliarialeucophrynariberothi*) was thoroughly discussed by Nordsieck (2013b: 14). The taxon is based on a specimen provided by Monterosato labelled as *Clausiliariberothi*, collected from a valley north of Ribera (Agrigento) (currently outside of the known distribution of *Siciliaria* and *Sicania*), housed at the Senckenberg Natural History Museum (SMF 163947). This specimen was depicted by Brandt (1961: 20–21). Brandt (1961: 8), as additional material, listed a ”Paratypoide“ from his own collection (Cl. 2518/5) but the origin of this sample(s) is not specified. Probably, the collecting data provided by Monterosato on the original label was wrong as was also stated by Beckmann (2004: 188) after a field survey.

Nordsieck (2013b: 5) depicted another specimen from Monterosato’s collection, labelled as *Clausiliaremota* (SMF 232084) collected by Monterosato from an unidentified locality, namely Monte della “Terrazza” or “Torrazza”. As stated by Beckmann (2004: 188), it is unlikely that this locality is Torrazza, east of San Vito lo Capo as from this locality only *Siciliariacalcarae* and *Siciliarianobilis* were found. Considering the overall shell shape, the prominent dorsal keel and the arrangement of the plicae and lamellae (AUPP and ALPP developed and visible from the frontal view of the mouth), both *Siciliariariberothi* and Monterosato’s samples from Monte della “Terrazza” or “Torrazza” could be provisionally referred to *Siciliariatiberii* ssp.

The concept of interspecies hybridisation (or geographic forms) introduced by Nordsieck (Nordsieck 2007: 99–101; 2013b: 10) refers to populations that share certain shell characters and have to some extent overlapping distribution [such as *calcarae*-*septemplicata* (e.g., Parco, south of Palermo; Sagana near Borghetto), *septemplicata*-*grohmanniana* (Punta Priola), *tiberii*-*calcarae* (Monte della Fiera), *leucophryna*-*calcarae* (Sferracavallo), *crassicostata*-*eminens* (Monte Cofano) etc]. When available, such populations were analysed anatomically and genetically in the present study. Hybridisation and gene flow proved to provide no suitable explanation for sharing common shell characters. The results did not support close relationships or hybridisation between these taxon pairs. The “hybridisation” cases will be discussed taxon by taxon in the taxonomic part.

## ﻿Taxonomic part

The list of the examined specimens of *Siciliaria* and *Sicania* is depicted in Table 4.

We isolated and depicted the clausiliar plates in order to verify the morphology described by Boettger (1877) for the two clausiliar types. The results are displayed in Table 5. Unexpectedly and in contrast to Boettger (1877), the ”deep gutter-shaped“ clausiliar plate, deemed by Boettger as being exclusive to the ”Gruppe of *crassicostata*”, was found to be present only in few taxa of the “Gruppe der *septemplicata*“, namely *Siciliariagrohammniana* s. l., *Siciliariaseptemplicata* and *Siciliariacalcaraeorlandoi* (see Table 5) but not present in any representative of the “Gruppe of *crassicostata*”. Despite this inconsistency, the “Gruppe of *crassicostata*” showed a stable structure of the clausiliar plate, which is, for all the taxa, distally not receding, not gutter-like and distally not bent upwards. The “Gruppe der *septemplicata*” presents a variable situation, with the taxa that present a combination of the three characters states. The genera *Siciliaria* and *Sicania* cannot be distinguished by any shell or genital character. No morphological character of the shell (surface, shape, decollation or dimensions), of the clausiliar apparatus and of the external/internal shape and sculpturing of the genital organs was found that is exclusively present in one genus. The main differences in shell and genital organs between *Siciliaria* and *Sicania* are listed in Table 6.

**Table 4.** *Siciliaria/Sicania* taxa examined (anatomy and/or molecular genetic data) with description of the variable penis-epiphallus transition: epiphallar formula. DNA = molecular genetic data available (Yes/No).

Taxon	Locality	DNA	Epiphallar formula
*Siciliariagrohmannianagrohmanniana* (Rossmässler, 1836)	Italy, Sicily, Palermo, Monte Pellegrino, N side of the top plateau, 380 m asl, 38°10'55.26"N, 13°21'1.66"E, leg. W. De Mattia and J. Macor leg.	Y	1ER(PP+ELP)
	Italy, Sicily, Palermo, Monte Pellegrino, Santuario Santa Rosalia, 420 m asl, 38°10'4.41"N, 13°21'2.60"E, leg. W. De Mattia and J. Macor leg.	Y	1ER(PP+ELP)
*Siciliariagrohmannianaaddaurae* ssp. nov. De Mattia, Reier & Haring	Italy, Sicily, Palermo, N side of Monte Pellegrino, Punta Priola, Grotte dell’Addaura, 115 m asl, 38°11'13.01"N, 13°21'6.91"E, W. De Mattia and J. Macor leg.	Y	1ER(PP)+ELP
*Siciliariaseptemplicata* (Philippi, 1836)	Italy, Sicily, Palermo, N side of Monte Gallo near Sferracavallo, 40 m asl, 38°12'39.27"N, 13°17'21.26"E, W. De Mattia and J. Macor leg.	Y	1ER+2ER(PP+ELP)
	Italy, Sicily, Palermo, E side of Monte Gallo, 140 m asl, 38°13'5.61"N, 13°18'4.76"E, W. De Mattia and J. Macor leg.	Y	1ER+2ER(PP+ELP)
*Siciliarialeucophryna* (L. Pfeiffer, 1862)	Italy, Sicily, Palermo, Grotta Conza, 150 m asl, 38°11'13.61"N, 13°16'44.68"E, W. De Mattia and J. Macor leg.	Y	1ER+2ER(PP+ELP)
	Italy, Sicily, Palermo, Sferracavallo, via Plauto, 50 m asl, 38°11'13.01"N, 13°21'6.91"E, W. De Mattia and J. Macor leg.	Y	1ER(PP)+ELP
*Siciliariacalcaraecalcarae* (Philippi, 1844)	Italy, Sicily, Alcamo, Monte Bonifato, top of the mountain, 640 m asl, 37°57'29.40"N, 12°57'33.64"E, A. Margelli leg.	N	1ER(PP)+ELP
	Italy, Sicily, Alcamo, Monte Bonifato, top of the mountain, 640 m asl, 37°57'29.40"N, 12°57'33.64"E, I. Niero leg.	Y	1ER(PP+ELP)
	Italy, Sicily, Alcamo, Monte Bonifato, west side of the mountain, over the quarry, 550 m asl, 37°57'16.92"N, 12°58'9.06"E, W. De Mattia and J. Macor leg.	N	1ER(PP+ELP)
	Italy, Sicily, Piana degli Albanesi, 500 m south of Portella Ginestra, northern cliffs of Monte Kumeta, 970 m asl, 37°58'13.35"N, 13°15'22.06"E, W. De Mattia and J. Macor leg.	N	1ER(PP)+2ER(ELP)
	Italy, Sicily, Castellammare del Golfo, Castello di Baida, W of the town along the road to Visicari, 300 m asl, 38° 02'41.64"N, 12°48'14.34"E, W. De Mattia and J. Macor leg.	N	1ER(PP)+2ER(ELP)
	Italy, Sicily, San Vito lo Capo, Castelluzzo, west cliffs E of town, 120 m asl, 38°06'25.71"N, 12°44'33.37"E, W. De Mattia and J. Macor leg.	Y	1ER(PP)+ELP
	Italy, Sicily, Castellammare del Golfo, Visicari, 395 m asl, 38°02'54.18"N, 12°48'26.61"E, W. De Mattia and J. Macor leg.	Y	1ER(PP+ELP)
*Siciliariacalcaraecalcarae* (Philippi, 1844) (= *adelina* Küster, 1847)	Italy, Sicily, Calatafimi, Castello Eufemio, 395 m asl, 37°57'45.67"N, 12°51'21.13"E, W. De Mattia and J. Macor leg.	N	1ER+2ER(PP)+ELP
*Siciliariacalcaraebelliemii* (Brandt, 1961)	Italy, Sicily, Partinico, W side of the Mount Belliemi, 440 m asl, 38°00'22.47"N, 13°06'37.79"E, W. De Mattia and J. Macor leg.	Y	1ER+2ER(PP)+ELP
	Italy, Sicily, Alcamo, E side of the Calatubo Castle, 75 m asl, 38°00'54.80"N, 12°59'13.35"E, W. De Mattia and J. Macor leg.	Y	1ER+2ER(PP+ELP)
*Siciliariacalcaraeborgettensis* ssp. nov. De Mattia, Reier & Haring	Italy, Sicily, Municipality of Borghetto, road to Romitello, 400 m asl, 38°02'59.53"N, 13°08'58.55"E, W. De Mattia and J. Macor leg.	Y	1ER(PP)+ELP
	Italy, Sicily, San Giuseppe Jato, quarry E of the town, 630 m asl, 37°58'15.07"N, 13°12'2.23"E, W. De Mattia and J. Macor leg.	Y	1ER+2ER(PP+ELP)
*Siciliariacalcaraeparajatinensis* ssp. nov. De Mattia, Reier & Haring	Italy, Sicily, Monreale, W part of Monte Kumeta toward Jato Antica, 630 m asl, 37°57'8.18"N, 13°13'14.57"E, W. De Mattia and J. Macor leg.,	Y	1ER+2ER(PP+ELP)
*Siciliariacalcaraeorlandoi* Liberto, Reitano, Giglio, Colomba & Sparacio, 2016	Italy, Sicily, Monreale, Bosco Ficuzza, Ponte Arcere, 470 m asl, 37°55'46.42"N, 13°23'5.22"E, W. De Mattia and J. Macor leg.	Y	1ER+2ER(PP+ELP)
*Siciliariacalcaraecruenta* ssp. nov. De Mattia, Reier & Haring	Italy, Sicily, Monreale, N side of Monte Gibilmesi, 890 m asl, 38°03'37.03"N, 13°12'38.53"E, W. De Mattia and J. Macor leg.	Y	1ER+2ER(PP)+ELP
*Siciliariaferrox* Brandt, 1961	Italy, Sicily, Trabia, Contrada Sant’Onofrio, 170 m asl, 37°59'17.62"N, 13°36'51.11"E, W. De Mattia and J. Macor leg.	Y	?ER+PP(ELP)
*Siliciariatiberitiberi* (A. Schmidt, 1868)	Italy, Sicily, Terrasini, Capo Rama, 30 m asl, 38°08'19.06"N, 13°03'14.01"E, W. De Mattia and J. Macor leg.	Y	ER+PP(ELP)
*Siciliariatiberiialcamoensis* Brandt, 1961 comb. nov.	Italy, Sicily, Cinisi, Piano Margi, 670 m asl, 38°08'58.56"N, 13°09'25.83"E, W. De Mattia and J. Macor leg.	Y	1ER(PP+ELP)
*Siciliariatiberiiarmettensis* ssp. nov. De Mattia, Reier and Haring	Italy, Sicily, Carini, Grotta dei Puntali o dell’Armetta, 90 m asl, 38°08'58.56"N, 13°09'25.83"E, W. De Mattia and J. Macor leg.	Y	1ER+2ER+3ER(PP+ELP)
*Siciliariatiberiiscalettensis* Beckmann, 2004	Italy, Sicily, Cinisi, Portella Scaletta [locus typicus], 90 m asl, 38°10'35.53"N, 13°07'27.22"E, W. De Mattia and J. Macor leg.	Y	1ER+PP(ELP)
	Italy, Sicily, Cinisi, along the SS113 road from Villagrazia di Carini to Cinisi, 70 m asl, 38°10'42.45"N, 13°07'34.50"E, W. De Mattia and J. Macor leg.	Y	1ER+PP(ELP)
*Sicaniacrassicostata* (Pfeiffer, 1856)	Italy, Sicily, Castelluzzo, cliffs W of the Tonnara di Monte Cofano, 50 m asl, 38°6'44.89"N, 12°40'34.27"E, W. De Mattia and J. Macor leg.	Y	1ER+PP(ELP)
	Italy, Sicily, Custonaci, cliffs N of the Mangiapane cave, 63 m asl, 38°05'40.91"N, 12°40'10.98"E, W. De Mattia and J. Macor leg.	Y	1ER(PP)+ELP
*Sicaniaeminens* (A. Schmidt, 1868), comb. nov.	Italy, Sicily, Custonaci, Baglio Messina, 340 m asl, 38° 4'06.38"N, 12°41'48.68"E, W. De Mattia and J. Macor leg.,	Y	1ER+2ER(PP+ELP)
	Italy, Sicily, Custonaci, Buffara, NE side of the hollow, 150 m asl, 38°04'0.52"N, 12°41'1.34"E, W. De Mattia and J. Macor leg.	Y	1ER(PP+ELP)
	Italy, Sicily, Custonaci, contrada Scurati, crossing with Strada Provinciale 18, 65 m asl, 38°04'56.04"N, 12°40'9.86"E, W. De Mattia and J. Macor leg.	Y	1ER(PP)+2ER(ELP)
	Italy, Sicily, Custonaci, contrada Scurati, 60 m asl, 38° 05'18.10"N, 12°40'17.18"E, W. De Mattia and J. Macor leg.	Y	1ER(PP)+2ER(ELP)
	Italy, Sicily, Custonaci, contrada Scurati, limestone cliffs S of the village, 60 m asl, 38°06'44.89"N, 12°40'34.27"E, W. De Mattia and J. Macor leg.	Y	1ER(PP)+2ER(ELP)
*Sicanianobilisnobilis* (L. Pfeiffer, 1848), comb. nov.	Italy, Sicily, Castelluzzo, Monte Cofano E of Tonnara di Monte Cofano, 80 m asl, 38°06'22.04"N, 12°40'59.77"E, W. De Mattia and J. Macor leg.	N	1ER+2ER+3ER(PP+ELP)
	Italy, Sicily, San Vito lo Capo, boulders W of the town, 50 m asl, 38°10'51.85"N12°43'37.70"E, W. De Mattia and J. Macor leg.	Y	1ER+2ER+3ER(PP+ELP)
	Italy, Sicily, San Vito lo Capo, Castelluzzo, west cliffs E of town, 120 m asl, 38°06'25.71"N, 12°44'33.37"E, W. De Mattia and J. Macor leg.	Y	1ER+2ER(PP)+ELP
	Italy, Sicily, San Vito lo Capo, cliffs S of “El-Bahira” camping, 60 m asl, 38°08'48.81"N12°44'06.61"E, W. De Mattia and J. Macor leg.	Y	1ER+2ER(PP)+ELP
*Sicanianobilisnobilis* (L. Pfeiffer, 1848), comb. nov. (= *episoma* Brandt, 1961)	Italy, Sicily, San Vito lo Capo, Torre delle Usciere, 5 m asl, 38°10'30.07"N, 12°46'14.09"E, W. De Mattia and J. Macor leg.	Y	1ER+2ER+3ER(PP+ELP)
*Sicanianobilisspezialensis* (Nordsieck 1984), stat. nov., comb. nov.	Italy, Sicily, San Vito Lo Capo, Macari, E side of Monte Speziale, 75 m asl, 38°07'41.78"N, 12°44'09.24"E, W. De Mattia and J. Macor leg.	Y	1ER+2ER(PP+ELP)

**Table 5.** Distribution of the clausilium character states within the *Siciliaria/Sicania* taxa. n = number of specimens analysed.

Taxon	n	Boettger’s groups: Gruppe der *septemplicata* / Gruppe der *crassicostata*	Clausiliar plate distally receding/not receding	Clausiliar plate gutter-like/not gutter like	Clausiliar plate bent upwards/not bent upwards
*Siciliariagrohmannianagrohmanniana* (Rossmässler, 1836)	22	*septemplicata*	receding	gutter-like	bent
*Siciliariagrohmannianaaddaurae* ssp. nov.	18	*septemplicata*	receding	gutter-like	bent
*Siciliariaseptemplicata* (Philippi, 1836)	15	*septemplicata*	receding	gutter-like	bent
*Siciliarialeucophryna* (L. Pfeiffer, 1862)	20	*septemplicata*	not receding	not gutter-like	not bent
*Siciliariacalcaraecalcarae* (Philippi 1844)	36	*septemplicata*	not receding	not gutter-like	bent
*Siciliariacalcaraebelliemii* (Brandt, 1961)	25	*septemplicata*	not receding	not gutter-like	bent
*Siciliariacalcaraeborgettensis* ssp. nov.	12	*septemplicata*	not receding	not gutter-like	bent
*Siciliariacalcaraejatinensis* ssp. nov.	21	*septemplicata*	not receding	not gutter-like	bent
*Siciliariacalcaraeparajatinensis* ssp. nov.	15	*septemplicata*	not receding	not gutter-like	bent
*Siciliariacalcaraeorlandoi* Liberto, Reitano, Giglio, Colomba & Sparacio, 2016	9	*septemplicata*	partially receding	gutter-like	bent
*Siciliariacalcaraecruenta* ssp. nov.	7	*septemplicata*	not receding	not gutter-like	bent
*Siciliariaferrox* (Brandt, 1961)	11	*septemplicata*	partially receding	partially gutter-like	not bent
*Siciliariatiberiitiberii* (A. Schmidt, 1868)	31	*septemplicata*	not receding	not gutter-like	bent
*Siciliariatiberiiarmettensis* ssp. nov.	33	*septemplicata*	not receding	not gutter-like	bent
*Siciliariatiberiihemmeni* (Beckmann, 2004), comb. nov.	6	*septemplicata*	not receding	not gutter-like	not bent
*Siciliariatiberiiscalettensis* Beckmann, 2004	27	*septemplicata*	not receding	not gutter-like	not bent
*Sicaniacrassicostata* (L. Pfeiffer, 1856), comb. nov.	19	*crassicostata*	not receding	not gutter-like	not bent
*Sicaniaeminens* (A. Schmidt, 1868), comb. nov.	29	*crassicostata*	not receding	not gutter-like	not bent
*Sicanianobilisnobilis* (L. Pfeiffer, 1848), comb. nov.	35	*crassicostata*	not receding	not gutter-like	not bent
*Sicanianobilisspezialensis* (Nordsieck, 1984), comb. nov., stat. nov.	21	*crassicostata*	not receding	not gutter-like	not bent

**Table 6.** Main morphological differences between *Siciliaria* and *Sicania*.

**Character**	***Siciliaria***	***Sicania***
Shell – waxy surface	not present	only in *Sicanianobilisnobilis*
Shell – percostate	not present	only in *Sicaniacrassicostata*
Shell – clausiliar plate gutter-like	only in *S.grohmannianas. l.* and *S.septemplicata*	not present
Shell – clausiliar plate receding	only in *S.grohmannianas. l.* and *S.septemplicata*	not present
Ganitalia – penial internal papillose sculpturing	not present	only in *Sicanianobilisnobilis*

### Order Stylommatophora

**Infraordo Clausilioidei Gray, 1855**

**Superfamilia Clausilioidea Gray, 1855**

**Familia Clausiliidae Gray, 1855**

**Subfamilia Alopiinae A.J. Wagner, 1913**

### Tribus Delimini R.A. Brandt, 1956

#### Genus *Siciliaria* Vest, 1867

##### ﻿ Siciliaria grohmanniana

Taxon classificationAnimaliaStylommatophoraClausiliidae

(Rossmässler, 1836) s. l.

6DBBAB53-D1A5-52FA-B9D1-E9432D6C9680

###### Remarks.

*Siciliariagrohmanniana* forms a subclade with its sister group *Siciliariaseptemplicata* (mean p distance 9.0%). The two subspecies of *S.grohmanniana* appear monophyletic in the mt tree (Fig. 4), albeit each represented by only a small number of samples: *Siciliariagrohmannianagrohmanniana* (one population with three samples), *Siciliariagrohmannianaaddaurae* ssp. nov. (one population with three samples). Mean distance between the two subspecies was found to be 3.3%.

The shell of *Siciliariagrohmannianaaddaurae* ssp. nov. was at first considered by Nordsieck (2013b: 6), as an ”intermediate geographic form“ of *S.grohmanninana* s. s. with ”shell not decollate and whorls more weakly ribbed“ and (2013b: 10) as: ”a *S.grohmanniana* with characters of *S.septemplicata*“ but without stating what these characters are. He introduced it as the: ”Priola form from Priola (= Punta Priola) and San Lorenzo near Palermo“ and depicted a shell (Nordsieck 2013b: 12, fig. 8). The internal penial sculpturing of *Siciliariagrohmannianagrohmanniana* is similar to *Siciliariaseptemplicata* (internal penial sculpturing showing a more or less fringed longitudinal pleats) although the sculpturing of epiphallus and vagina shows greater variability.

##### ﻿ Siciliaria grohmanniana grohmanniana

Taxon classificationAnimaliaStylommatophoraClausiliidae

(Rossmässler, 1836)

389B2207-9F0A-5AE4-A23B-9CE1D26A3029

Figs 1.F, 7.1–7.3, 8.1–8.4, 13.1–13.4

Clausilia grohmanniana Rossmässler 1836: 7.
Clausilia grohmanniana var. minor A. Schmidt 1868: 40 [non Rossmässler].
Clausilia grohmanuiana [sic!] – Benoit 1876: 151.
Clausilia grohmaniana [sic!] – Benoit 1881: 108.
Clausilia (Siciliaria) grohmanniana – Monterosato 1894: 170.
Delima (Siciliaria) grohmanniana – Wagner 1925: 67.
Siciliaria grohmanniana – Schileyko 2000: 665.
Charpentieria grohmanniana – Beckmann 2004: 188.
Siciliaria grohmanniana – Nordsieck 2007: 53.
Siciliaria grohmanniana – Welter-Schultes 2012: 340.
Siciliaria grohmanniana – Nordsieck 2013b: 6.
Charpentieria grohmanniana – De Mattia 2017a.

###### Specimens examined.

Italy, Sicily, Palermo, Monte Pellegrino, N side of the top plateau, 380 m asl, 38°10'55.26"N, 13°21'1.66"E, W. De Mattia and J. Macor leg., 20.xii.2003. 15 live spm, 3 dissected spm. Italy, Sicily, Palermo, Monte Pellegrino, Santuario Santa Rosalia, 420 m asl, 38°10'4.41"N, 13°21'2.60"E, [Lab ID 42_1, COI: MW758886; Lab ID 42_2, COI: MW758887, ITS2: MW757091MW757092; Lab ID 42_3, COI: MW758885], W. De Mattia and J. Macor leg., 15.iv.2017. 12 live spm, 3 dissected spm.

###### Shell (Figs 8.1–8.4, 13.1–13.4).

Shell mostly decollate; whorls ribbed; dorsal keel indistinct or missing; inferior lamella very high; two anterior upper palatal plicae present, upper one mostly separated from upper palatal plica; palatal edge of clausilium plate distally receding, plate gutter-like narrowed, palatal edge against distal end bent upwards and more or less pointed (as in Nordsieck 2013b).

###### Measurements

(n = 25, decollate): shell height 19.4 ± 0.8, whorl width 5.1 ± 0.2, aperture height 3.9 ± 0.2, aperture width 2.7 ± 0.2.

###### External morphology of the genital organs (Fig. 7.1).

The FO is longer than the V (FO/V range from 1.6 to 1.8). The VD is thin along its whole course. The FDBC is shorter than the BC+SDBC (FDBC/BC+SDBC range from 0.7 to 0.8). The BC+SDBC is cylindrical or club-like and slightly longer than the V (BC+SDBC/V range 1.3–1.4), with no clear distinction between the SDBC and the BC. The apex is wide and round or pointed. The D is longer than the V (D/V range 2.0–2.1) and longer that the BC+SDBC (D/BC+SDBC range 1.4–1.7), thinner than the BC+SDBC and with a pointed apex. The V is short and cylindrical. The A is large. The PC is longer than the V (P+E/V range 2.4–2.6). The PR is long and robust. There is a clear distinction between P and E as there is a visible ER and a proximal narrowing. The E is thinner but longer than the P (E/P range 1.1–1.3), almost gradually shrinking and turning into the VD.

###### Internal morphology of the genital organs (Figs 7.2, 7.3).

The A is smooth or with weak traces of the distal penial pleats. The P presents 4–6 longitudinal and very irregular pleats. These pleats are very variable in thickness and sculpture, being both smooth or segmented along the same pleat. These pleats often split (proximally, distally or both) into smaller pleats. The fine structure of the wall is smooth. The PP is big, irregular, wrinkly and pointed. It can be smooth or with small tubercles. The P-E transition presents one ER with the PP and ELP originating from the ER. The epiphallar formula is: 1ER(PP+ELP). The E shows 4 to 6 main longitudinal finely fringed pleats. The V is almost completely smooth. The wall is finely granulated.

###### Ecology.

*Siciliariagrohmannianagrohmanniana* is widespread and common throughout its range. It is found to live on exposed limestone walls hiding in cracks and under crags. It is also found in *Pinus* sp. forest on decaying woods and tree trunks, including shady and humid spots on mosses and ferns. According to De Mattia (2017a)*Siciliariagrohmannianagrohmanniana* is considered as Endangered following the IUCN criteria B1ab(i,ii,iii,v)+2ab(i,ii,iii,v). The whole area of distribution is currently included in protected areas.

###### Distribution.

The taxon is limited to the calcareous mountains northern of Palermo: Monte Pellegrino and the southern slopes of Monte Gallo.

##### ﻿ Siciliaria grohmanniana addaurae ssp. nov.

Taxon classificationAnimaliaStylommatophoraClausiliidae

BD00F7E2-77DE-5647-84CB-BD9EEF2B9429

http://zoobank.org/47B512C7-4087-41F2-8781-EB2A56803C95

Figs 1.F, 7.4–7.6, 8.5–8.9, 13.5–13.8

###### Type locality.

Italy, Sicily, Palermo, N side of Monte Pellegrino, Punta Priola, Grotte dell’Addaura, 115 m asl, 38°11'13.01"N, 13°21'6.91"E.

###### Type material.

1 ***Holotype*** (NHMW 113611) [Lab ID 41_1, COI: MW758883, ITS2: MW757093, MW757094] and 8 ***Paratypes*** (NHMW 113612): Italy, Sicily, Palermo, N side of Monte Pellegrino, Punta Priola, Grotte dell’Addaura, 115 m asl, 38°11'13.01"N, 13°21'6.91"E, [Lab ID 41_2, COI: MW758884; Lab ID 41_3 COI: MW758929], W. De Mattia and J. Macor leg., 15.iv.2017. 3 dissected spm. 10 ***Paratypes*** (CWDM 18222): same locality.

###### Shell diagnosis.

Shell decollate; whorls ribbed; dorsal keel weak but distinguishable; inferior lamella very high; two anterior upper palatal plicae present, both of them separated from the lunella; parietalis short ends before the beginning of the spiralis; palatal edge of clausilium plate distally receding, plate gutter-like narrowed, palatal edge against distal end bent upwards and more or less pointed.

###### Shell description (Figs 8.5–8.9, 13.5–13.8).

The shell is elongated, fusiform, sinistral and decollate, rarely not decollate. It is brown to reddish brown in colour. The external surface has small raised ribs almost equally arranged in all whorls of the teleoconch. The spire slowly and regularly grows with eight or nine whorls that are only slightly convex. The sutures are moderately shallow with very rare whitish papillae present towards the apex. The basal and the cervical keels are weak but distinguishable. The umbilicus is closed. The aperture width is ~ ^1^⁄_4_ of shell height and subovoid in shape. The PRI is long and raised, wider along its posterior part and not fused with the L. The PRI is not visible from the aperture. The L is antero-lateral, with a reduced, knob-like PUPP connected to it. There are two AUPP, both of them detached from the L and barely visible from the aperture. The upper AUPP is stronger and longer than the lower one. The ALPP (BAS) starts directly from the L and it is long and strong, clearly visible from the aperture. The SCL is present and robust. It is connected to the L. The IL is high to very high. The SUL is short, tooth-like and ends before the beginning of the SPL that is wider along its posterior part. The SCOL is not emergent. The peristome is continuous, markedly thickened and reflected. It is not superiorly fused to the wall of the first whorl. The palatal edge of the clausilium is distally receding and bent upwards. The plate is narrow and gutter-like.

**Figure 7.**
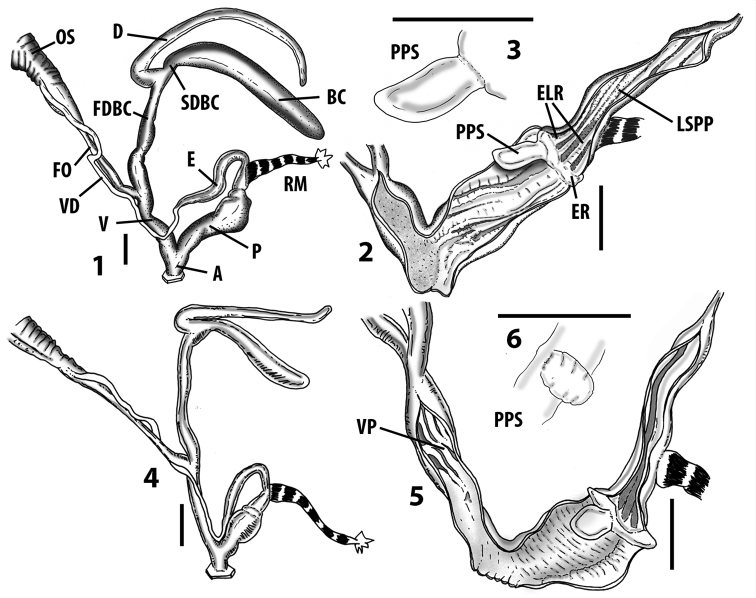
*Siciliariagrohmannianagrohmanniana* (Rossmässler, 1836), Monte Pellegrino, Palermo **7.1** whole distal genital organs **7.2** internal distal part of genital organs **7.3** penial pseudopapilla detail. *Siciliariagrohmannianaaddaurae* ssp. nov. Grotta dell’Addaura, Punta Priola **7.4** whole distal genital organs **7.5** internal distal part of genital organs **7.6** penial pseudopapilla detail.

**Figure 8.**
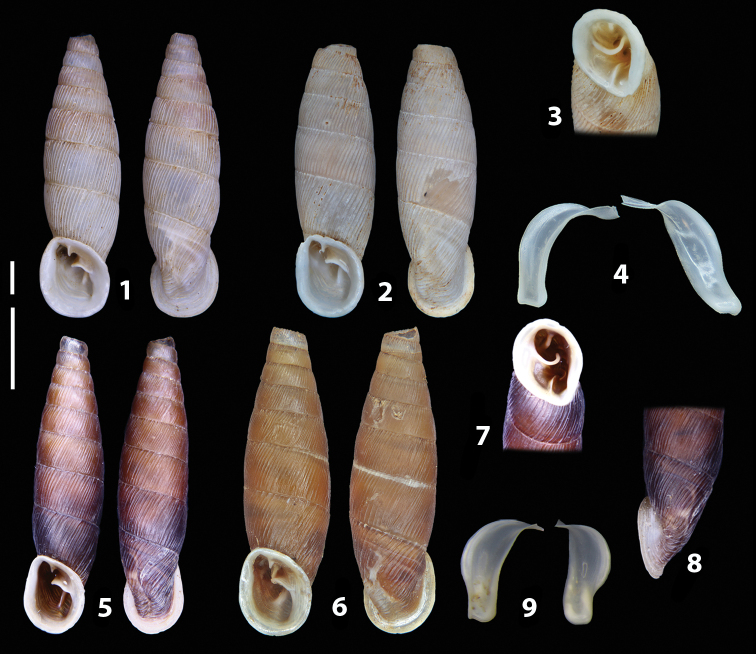
*Siciliariagrohmannianagrohmanniana* (Rossmässler, 1836), Monte Pellegrino, Palermo **8.1** shell **8.2** shell **8.3** detail of the aperture **8.4** clausiliar plate double side. *Siciliariagrohmannianaaddaurae* ssp. nov. Grotta dell’Addaura, Punta Priola **8.5** shell **8.6** shell **8.7** detail of the aperture **8.8** detail of the columellar side of last whorl **8.9** clausiliar plate double side.

###### Measurements

***Holotype***: decollate shell height 17.3, whorl width 4.5, aperture height 4, aperture width 2.5. ***Paratypes*** (n = 20, decollate): shell height 17.2 ± 0.6, whorl width 4.4 ± 0.1, aperture height 3.9 ± 0.3, aperture width 2.9 ± 0.3.

###### External morphology of the genital organs (Fig. 7.4).

The FO is longer than the V (FO/V range 1.5–1.7). The VD is thin along its whole course. The FDBC is longer than the BC+SDBC (FDBC/BC+SDBC range 1.0–1.3). The BC+SDBC is cylindrical or club-like and slightly longer than the V (BC+SDBC/V = 1.1), with no clear distinction between the SDBC and the BC. The apex is wide and round. The D is longer than the V (D/V range 1.3–1.5) and longer that the BC+SDBC (D/BC+SDBC range 1.1–1.3), thinner than the BC+SDBC and with a small apex. The V is long and cylindrical and thin in diameter. The A is small. The PC is longer than the V (P+E/V range 1.6–1.8). The PR is long and thin. There is a clear distinction between P and E as there is a visible ER and a proximal narrowing. The E is much thinner but longer than the P (E/P range 1.2–1.5), gradually shrinking and turning into the vas deferens.

###### Internal morphology of the genital organs (Figs 7.5, 7.6).

The A is smooth. The P presents very smooth and scarcely elevated weak transverse pleats. The fine structure of the wall is smooth. The PP is big, rounded and smooth. It originates from the big epiphallar ring. The P-E transition presents one ER with the PP originating from it. The ELP are not connected with the ER. The epiphallar formula is: 1ER(PP)+ELP. The E shows three or four main smooth longitudinal pleats. The proximal V shows an irregular set of smooth longitudinal pleats that, distally, turn into a smooth fold that goes as far as the atrium.

###### Comparative analysis.

*Siciliariagrohmannianaaddaurae* ssp. nov. differs from *Siciliariagrohmannianagrohmanniana* by its slender shape and darker shell colour and its denser ribbing (Fig. 8.5, 8.6). The two AUPP are longer and stronger. The basal and the cervical keels are more prominent. The internal wall of the P presents different sculpturing: smooth and scarcely elevated weak transverse pleats versus 4 to 6 longitudinal and very irregular pleats (Fig. 7.5). *Siciliariagrohmannianaaddaurae* ssp. nov. differs from *Siciliariaseptemplicata* by its more ribbed surface and the stronger ALPP. The SCL is much longer and more robust. The internal wall of the P presents different sculpturing: smooth and scarcely elevated weak transverse wrinkles versus four to five smooth longitudinal or fringed pleats. (Fig. 7.5).

###### Distribution.

*Siciliariagrohmannianaaddaurae* ssp. nov. is exclusively known from the type locality, along the limestone cliffs around the Addaura caves near Punta Priola, Palermo. It is likely to be present in other spots along the northeastern limestone cliffs of the Monte Pellegrino, but more research is needed to define its distribution.

###### Ecology.

*Siciliariagrohmannianaaddaurae* ssp. nov. is an obligate rock-dweller and inhabits the limestone cliffs, on open walls or hiding in cracks in humid spots, around the Addaura caves. This subspecies has a very limited distribution range with an area of much less than 1 km^2^. Although, the area is included in the Riserva Naturale Orientata Monte Pellegrino and the Addaura caves are fenced, the habitat quality is inferred to be declining due to no access regulation nor restriction, resulting in dumping, littering and murals on the limestone walls (Ferrante 2011).

###### Etymology.

The taxon is named after the Addaura caves, a complex of three natural caverns where wall engravings dated to the Paleolithic and the Mesolithic were discovered.

##### ﻿ Siciliaria septemplicata

Taxon classificationAnimaliaStylommatophoraClausiliidae

(Philippi, 1836)

9FE8C514-3272-5A08-9EAF-1239D18CAB52

Figs 1.F, 9.1–9.2, 10.1–10.7, 13.9–13.11

Clausilia septemplicata Philippi 1836: 139.
Clausilia sericina Rossmässler 1836: 7.
Clausilia septemplicata – Calcara 1840: 15.
Clausilia septemplicata var. prasina A. Schmidt 1868: 41.
Clausilia septemplicata – Benoit 1876: 151.
Clausilia prasina Benoit 1876: 151.
Clausilia septemplicata – Monterosato 1882: 102.
Clausilia (Siciliaria) septemplicata – Monterosato 1894: 170.
Delima (Siciliaria) septemplicata prasina – Wagner 1913: plate 572, fig. 14.
Charpentieria septemplicata – Beckmann 2004: 188.
Siciliaria septemplicata – Welter-Schultes 2012: 343.
Siciliaria septemplicata – Nordsieck 2013b: 6.
Charpentieria septemplicata – De Mattia 2017b.

###### Taxonomical and phylogenetic remarks.

The case of *Siciliariaseptemplicata* and its alleged subspecies (“geographic forms” following Nordsieck 2013b: 7) highlights the weakness of a entirely shell-based taxonomy at the species level.

###### Specimens examined.

Italy, Sicily, Palermo, N side of Monte Gallo near Sferracavallo, 40 m asl, 38°12'39.27"N, 13°17'21.26"E, [Lab ID 56_1, COI: MW758904, ITS2: MW757137, MW757138], W. De Mattia and J. Macor leg., 16.iv.2017. 5 live spm, 2 dissected spm. Italy, Sicily, Palermo, E side of Monte Gallo, 140 m asl, 38°13'5.61"N, 13°18'4.76"E, [Lab ID 57_1, COI: MW758937, ITS2: MW757070], W. De Mattia and J. Macor leg., 16.iv.2017. 4 live spm, 2 dissected spm.

###### Shell

**(Figs 10.1–10.7, 13.9–13.11).** Shell mostly not decollate; whorls rib-striated, with sutural papillae; dorsal keel indistinct or missing; inferior lamella very high; two anterior upper palatal plicae present, upper one mostly separated from upper palatal plica; palatal edge of clausilium plate distally receding, plate gutter-like narrowed, palatal edge against distal end bent upwards and more or less pointed (as in Nordsieck 2013b).

###### Measurements

(n = 18, not decollate): shell height 17.9 ± 0.6, whorl width 4.2 ± 0.2, aperture height 3.9 ± 0.2, aperture width 2.9 ± 0.4.

###### External morphology of the genital organs (Fig. 9.1).

The FO is longer than the V (FO/V range from 1.1 to 3.9). The VD is thin along its whole course. The FDBC is slightly longer than the BC+SDBC (FDBC/BC+SDBC range 1.1–1.9). The BC+SDBC is cylindrical to spindle-like and longer than the V (BC+SDBC/V range 1.1–1.3), with no clear distinction between the SDBC and the actual BC. The apex is big and pointed. The D is longer than the V (D/V = 1.9) and longer that the BC+SDBC (D/BC+SDBC range 1.6–1.7), thinner than the BC+SDBC and with a small and round apex. The V is cylindrical. The A is large. The PC is longer than the V (P+E/V range 2.0–2.4). The PR is long and thin or short and strong. The E is as long as the P (E/P = 1.0), gradually shrinking and turning into the VD.

**Figure 9.**
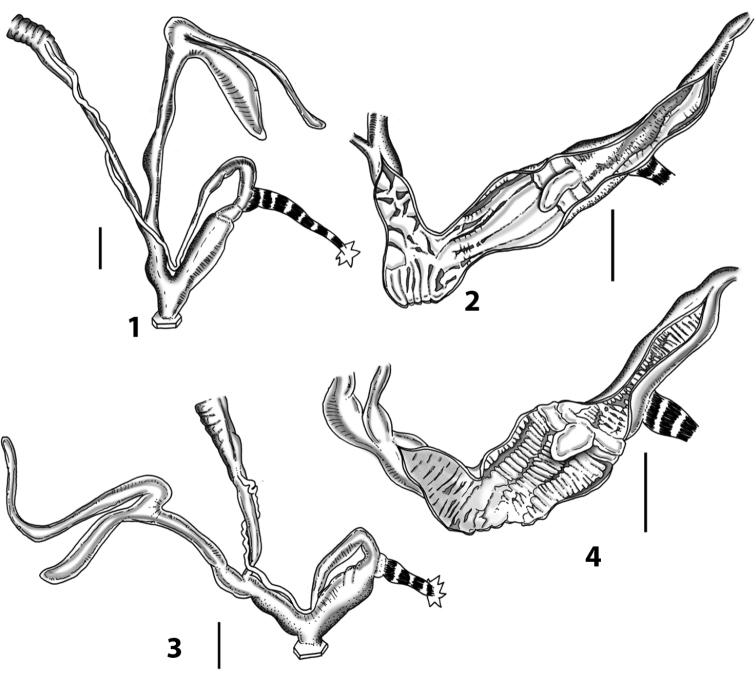
*Siciliariaseptemplicata* (Philippi, 1836), Monte Gallo, Sferracavallo **9.1** whole distal genital organs **9.2** internal distal part of genital organs **9.3** whole distal genital organs **9.4** internal distal part of genital organs.

###### Internal morphology of the genital organs (Fig. 9.2).

The A shows a set of longitudinal irregular pleats. The P presents four to five smooth longitudinal or slightly fringed pleats. The fine structure of the wall is smooth. The PP is small to medium in dimensions and elongated. The P-E transition presents a first distal ER, PP and ELP originate from the second proximal ER. The epiphallar formula is: 1ER+2ER (PP+ELP). The E shows one main longitudinal slightly segmented pleat that fades out before the VD. Few additional small, weak, elevated pleats can also be present. The wall of the E is coarsely granulated. The V presents a set of irregular fleshy folds.

**Figure 10.**
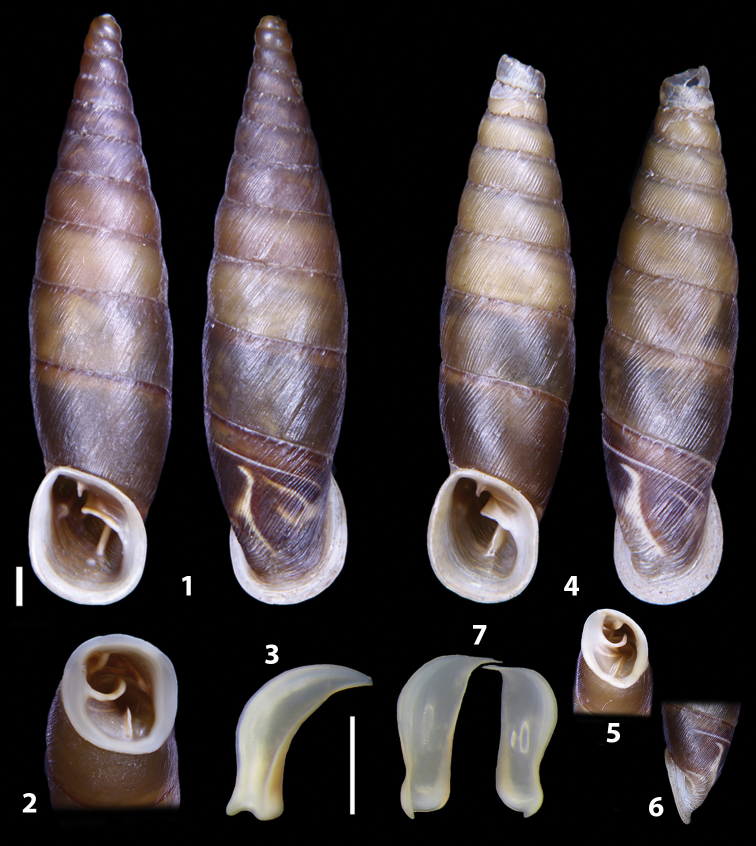
*Siciliariaseptemplicata* (Philippi, 1836), Monte Gallo, Sferracavallo **10.1** shell **10.2** detail of the aperture **10.3** clausiliar plate **10.4** shell **10.5** detail of the aperture **10.6** detail of the columellar side of last whorl **10.7** clausiliar plate double side.

###### Ecology.

*Siciliariaseptemplicata* was found dwelling on limestone cliffs, hiding in cracks in exposed or shady spots. According to De Mattia (2017b) the species is Near Threatened.

###### Distribution.

Following the new taxonomical results, the distribution of *Siciliariaseptemplicata*, must be reconsidered. Beckmann (2004: 188) and Nordsieck (2013b: 7) described a wide area from Cinisi in the west and Monte Gallo in the north to Piana degli Albanesi in the south and Monte Catalfano in the east. At least the area of Cinisi must be removed from the distribution list of *Siciliariaseptemplicata*, it is occupied by *S.tiberiialcamoensis* comb. nov.

##### ﻿ Siciliaria leucophryna

Taxon classificationAnimaliaStylommatophoraClausiliidae

(L. Pfeiffer, 1862)

8D9A0FAF-EA71-520C-A06C-C1BCF4FC7D89

Figs 1.F, 11.1–11.5, 12.1–12.6, 13.12–13.14

Clausilia leucophryna Pfeiffer 1862: 204.
Siciliaria leucophryne [sic!] – Boettger 1877: 34.
Clausilia leucophryne var. laudabilis [sic!] Boettger 1879: 34.
Clausilia lencophryna [sic!] – Benoit 1881: 108.
Clausilia confinata merens Westerlund 1892: 47.
Clausilia leucophryne laudabilis [sic!] – Westerlund 1892: 48.
Charpentieria leucophryna – Beckmann 2004: 188.
Siciliaria leucophryna – Welter-Schultes 2012: 341.
Siciliaria leucophryna – Liberto et al. 2012: 560.
Siciliaria leucophryna – Nordsieck 2013b: 7.
Siciliaria leucophryna – Liberto et al. 2015: 489.
Charpentieria leucophryna – De Mattia 2017c.
Siciliaria leucophryna microinsularis Sparacio et al. 2021: 598.

###### Remarks.

*Siciliarialeucophryna* in the mt tree forms a separate subclade within the big *S.calcarae* clade (mean p distance 11.3%). As depicted in the combined tree (Fig. 6), the close relationship of this species with *S.calcarae* appears clear. *Siciliarialeucophrynamicroinsularis* Sparacio, Surdo, Viviano, Liberto & Reitano, 2021 was recently introduced from the island of Isola delle Femmine (Palermo). In the comparative notes provided by Sparacio et al. (2021: 599), the character states reveal to fall into the variability we detected for the topotypical specimens from Grotta Conza (Palermo), both regarding shell and genital morphology. In the type locality, small specimens (**H_s_** = 14.7 mm) and light brown colour of the shell are also known. The same applies for the connection between the AUPP and PUPP where specimens from the type locality also show a “wide” spacing. Following the pictures provided by Sparacio et al. (2021: figs 43, 44), the PP of *S.leucophrynamicroinsularis* does not appear longer if compared to the PP of the specimens from the type locality (Figs 11.2, 11.3, 11.5). The variability of the sculpturing of the internal walls of the vagina refrains us to consider it a reliable taxonomic character for the whole genus *Siciliaria*. The status of *S.leucophrynamicroinsularis* should be further investigated morphologically as well as genetically. Anyhow, *Siciliarialeucophrynamicroinsularis* Sparacio, Surdo, Viviano, Liberto & Reitano, 2021 is here deemed as a local form, thus a junior synonym of *Siciliarial.leucophryna*.

The population from Via Plauto, concerning the shell, matches with the “Sferracavallo form” described by Nordsieck (2013b: 7), showing a lower inferior lamella and the palatal edge of the clausilium plate more strongly bent upwards.

###### Specimens examined.

Italy, Sicily, Palermo, Grotta Conza, 150 m asl, 38°11'13.61"N, 13°16'44.68"E, [Lab ID 55_1, COI: MW758902, ITS2: MW757124; Lab ID 55_2, COI: MW758903], W. De Mattia and J. Macor leg., 15.iv.2017. 15 live spm, 3 dissected spm. Italy, Sicily, Palermo, Sferracavallo, via Plauto, 50 m asl, 38°12'1.32"N, 13°16'3.05"E, [Lab ID 43_1, COI: MW758888], W. De Mattia and J. Macor leg., 15.iv.2017. 7 live spm, 2 dissected spm.

###### Shell

(**Figs 12.1–12.6, 13.12–13.14).** Shell decollate; whorls ribbed; dorsal keel mostly indistinct or missing; inferior lamella moderately high; anterior upper palatal plica present, widely separated from or connected with upper palatal plica; palatal edge of clausilium plate distally slightly or not receding, palatal edge distally bent upwards (as in Nordsieck 2013b) or not.

###### Measurements

(n = 35, decollate): shell height 18.2 ± 2.1, whorl width 4.3 ± 0.2, aperture height 4.5 ± 0.2, aperture width 3.4 ± 0.2.

###### External morphology of the genital organs (Figs 11.1, 11.4).

The FO is longer than the V (FO/V range 2.1–2.4). The VD is thin along its whole course. The FDBC of the BC+SDBC is longer than the BC+SDBC (D/BC+SDBC = 2.1). The BC+SDBC is club-like to cylindrical and longer than the V (BC+SDBC/V range 1.4–1.6), with no clear distinction between the SDBC and the BC. The apex is wide and rounded. The D is longer than the V (D/V range 2.7–2.8) and longer that the BC+SDBC (D/BC+SDBC range 1.9–2.4), thinner than the BC+SDBC and with a pointed apex. The V is short and cylindrical. The A is very large. The PC is much longer than the V (P+E/V range 2.4–3.9). The PR is long and thin. There is a clear distinction between P and E as there is a visible ER and a proximal narrowing. The E is thinner but longer than the P (E/P range 1.4–1.8), almost gradually shrinking and turning into the VD.

**Figure 11.**
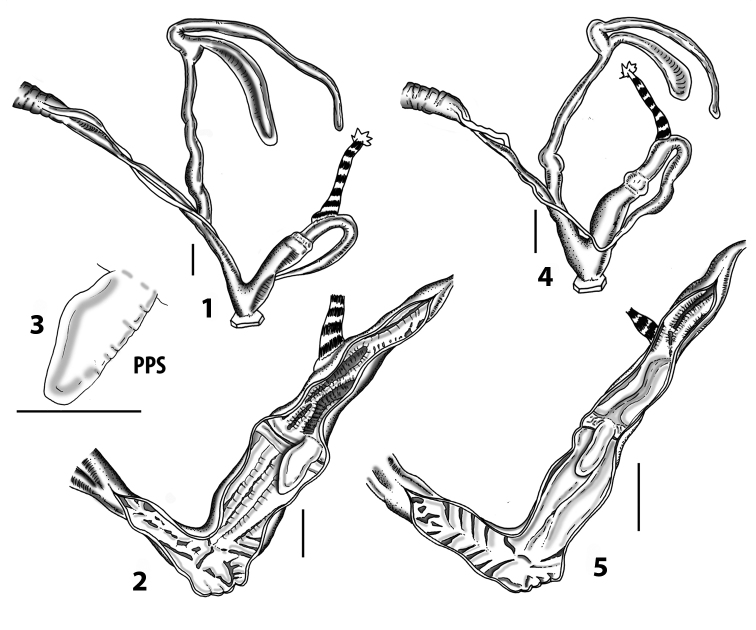
*Siciliarialeucophryna* (L. Pfeiffer, 1862), Grotta Conza, Palermo **11.1** whole distal genital organs **11.2** internal distal part of genital organs **11.3** pseudopapilla detail. Sferracavallo, via Plauto **11.4** whole distal genital organs **11.5** internal distal part of genital organs.

**Figure 12.**
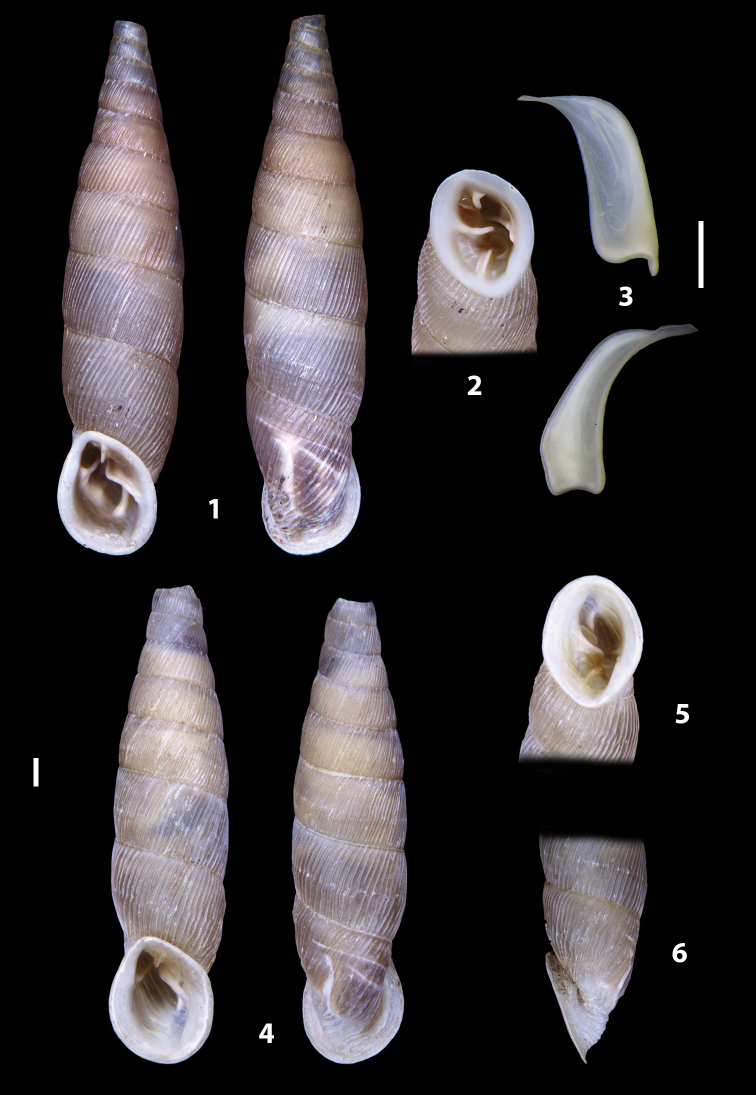
*Siciliarialeucophryna* (L. Pfeiffer, 1862), Grotta Conza, Palermo **12.1** shell **12.2** detail of the aperture **12.3** clausiliar plate double side. Sferracavallo, via Plauto **12.4** shell **12.5** detail of the aperture **12.6** detail of the columellar side of last whorl.

###### Internal morphology of the genital organs (Figs 11.11.2–11.2, 11.4).

The A shows a set of irregular fleshy folds. The P presents two big smooth longitudinal pleats that occupy almost the whole internal penial space. The fine structure of the wall is smooth. The PP is very elongated and smooth with a pointed tip. The P-E transition presents two slightly different structures. The population from the L.T. (Grotta Conza) presents a first distal ER, the PP and ELP originate from the second proximal ER. The epiphallar formula is: 1ER+2ER(PP+ELP). The V presents irregular, transverse to slightly longitudinal pleats. The population from Sferracavallo presents one ER with the PP originating from it. The ELP are not connected with the ER. The epiphallar formula is: 1ER(PP)+ELP. Distally, the E shows two main ELP that, at its mid length abruptly become finely fringed. The V presents a set of transverse-oblique smooth pleats that merge together one into another forming a kind of chevron or irregular pattern.

**Figure 13.**
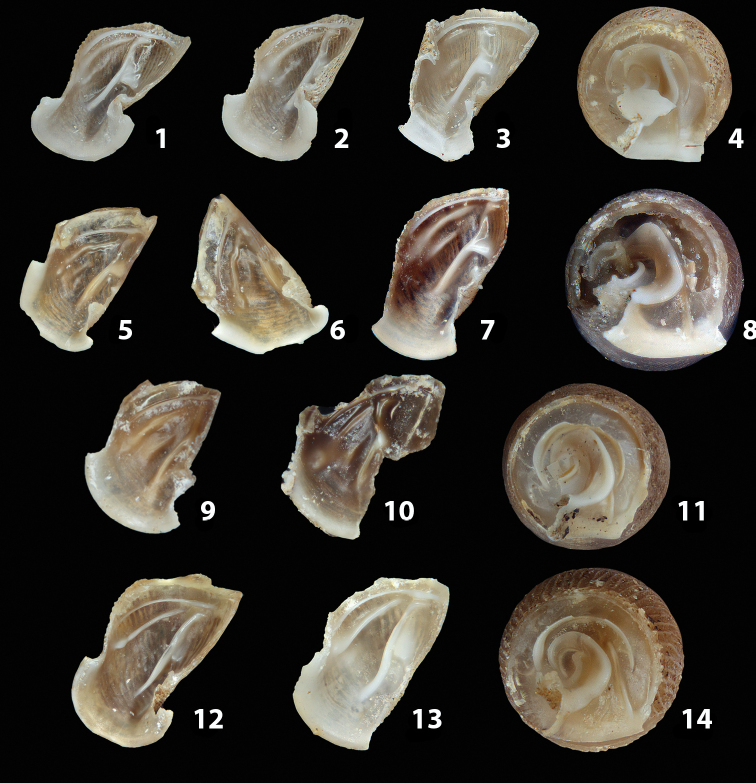
*Siciliariagrohmannianagrohmanniana* (Rossmässler, 1836), Monte Pellegrino, Palermo. **13.1–13.3** palatal plicae **13.4** parietal lamellae *Siciliariagrohmannianaaddaurae* ssp. nov., Grotta dell’Addaura, Punta Priola **13.5–13.7** palatal plicae **13.8** parietal lamellae. *Siciliariaseptemplicata* (Philippi, 1836), Monte Gallo, Sferracavallo **13.9–13.10** palatal plicae **13.11** parietal lamellae. *Siciliarialeucophryna* (L. Pfeiffer, 1862), Grotta Conza, Palermo **13.12–13.13** palatal plicae **13.14** parietal lamellae.

###### Ecology.

*Siciliarialeucophryna* colonises limestone cliffs hiding in cracks or under stones. It is very common on isolated boulders and tree trunks and barks in the xerophilic scrub around Grotta Conza. The species is quite abundant where found. According to De Mattia (2017c) the species is Endangered following the IUCN criteria B1ab(iii,v)+2ab(iii,v). The map provided by De Mattia (2017) partially misses a portion of the actual distribution area, as the western part was left out from the perimeter (Pizzo Mollica and Isola delle Femmine).

###### Distribution.

*Siciliarialeucophryna* is known to occur from a small area from Isola delle Femmine to Sferracavallo, including the northernmost sides of Pizzo Mollica and Pizzo Manolfo (where Grotta Conza is located) [(Sparacio et al. (2021)]. Nordsieck (2013b) reports also Monte Gallo, despite recent field collecting failed to find it there. Further field research throughout the whole Pizzo Manolfo-Pizzo Castellaccio-Cozzo San Rocco massif is required to precisely define the distribution of this species.

##### ﻿ Siciliaria calcarae

Taxon classificationAnimaliaStylommatophoraClausiliidae

(Philippi, 1844) s. l.

2E574A6B-4726-5CBB-B7E2-8CD4BEED44DB

###### Remarks.

In the COI tree two subclades of *Siciliariacalcarae* s. l. appear together with *S.leucophryna* in an unresolved trichotomy. The subspecies of *Siciliariacalcarae* are distributed within two haplogroups, one (haplogroup 1) comprising the nominate subspecies as well as *S.c.belliemii*, *S.c.borgettensis* ssp. nov., *S.c.orlandoi*, *S.c.parajatinensis* ssp. nov. and the other one (haplogroup 2) consisting of *S.c.cruenta* ssp. nov. and *S.c.jatinensis* ssp. nov. Unfortunately, *S.parajatinensis* could not be included into the ITS2 analysis. Yet, given the low distances among all representatives of *S.calcarae* in the ITS2 tree (and the resulting bad resolution), this data set does not tell us much concerning these relationships. Thus, presently the close relationship between *S.c.orlandoi* and *S.c.parajatinensis* hast to rely on mt data only. Concerning the anatomy of the genital organs, shell morphology, distribution, ecology and haplogroup of *Siciliariacalcarae*, two general types can be distinguished for each category.

###### Genital organs.

Two main anatomical types of male (penial) internal sculpturing are found. The first type presents clear longitudinal pleats that run from the origin of penial pseudopapilla down to (or almost to) the atrium. These longitudinal pleats can be either (almost) completely smooth (Monte Belliemi) or more or less markedly fringed (Calatubo, Romitello, Ficuzza, Jato city park, Monte Kumeta, and Castelluzzo). The second type presents transverse and strong smooth pleats (Bonifato, Gibilmesi, and Visicari).

###### Sculpturing of the surface of the teleoconch.

The first type corresponds to *S.calcaraecalcarae* (Philippi, 1844) morphotype, which is more or less striated (Bonifato, Ficuzza, Romitello, Visicari, Gibilmesi, and Castelluzzo), whereas the second type presents a markedly ribbed shell (Calatubo, Jato city park, Monte Kumeta, and Monte Belliemi).

###### Distribution.

Throughout the whole *S.calcarae* distribution, the nominate subspecies *S.calcaraecalcarae* has the widest distribution, whereas all the remaining subspecies have an isolated, punctiform distribution. The populations from Alcamo, Visicari and Castelluzzo belong to the widely distributed nominate subspecies (1)m whereas the remaining populations (Ficuzza, Calatubo, Monte Kumeta, Jato city park, Romitello, Gibilmesi and Monte Belliemi) belong to limited, punctiform populations (2).

###### Ecology.

The populations belonging to this subclade were collected in two main ecological niches. Most of them (type 1) were found on limestone rocks and cliffs (Jato city park, Bonifato, Calatubo, Gibilmesi, Monte Kumeta, Romitello, Monte Belliemi and Visicari), whereas the other populations (type 2), from Ficuzza and Castelluzzo, were exclusively found on tree trunks.

###### Haplotypes.

Concerning the haplogroups of the *calcarae* clade, eight populations fall into subclade 1 (Bonifato, Calatubo, Romitello, Monte Belliemi, Visicari and Catselluzzo, Monte Kumeta, Ficuzza), whereas two populations (Gibilmesi, and Jato city park) fall in to the subclade 2. *Siciliariacalcaraeorlandoi*Liberto et al., 2016 was recently described from a few scattered localities restricted to Bosco Ficuzza (Corleone, Sicily) (Liberto et al. 2016: 371). The arrangement of the internal penial sculpturing of the holotype seems not matching with what was found by our dissection, where, instead of ”two long weak longitudinal furrows” (Liberto et al. 2016: 372) a set of transverse, interrupted fleshy smooth pleats were found instead. Yet, the authors provided besides pictures and description of the external morphology of the genital organs, only very few details on the internal morphology of the genital organs, namely: ”internal walls of penis show two long weak longitudinal furrows; conic penial papilla, with slightly pointed apex and a restriction to the base”. The brief description and the quality of the images (Liberto et al. 2016: 375) do not allow accurate comparison of the internal features with those found in the present analysis. Moreover, the pseudopapilla was erroneously considered as a true penial papilla.

The position of *S.calcaraeorlandoi* in the COI tree within haplogroup 1 is close to sequences of a population of *Siciliaria* from the western slopes of Monte Kumeta. Despite the close phylogentic relationship of this population with *S.calcaraeorlandoi*, the differences in the arrangement of the internal male genital organs (a set of transverse, interrupted fleshy smooth pleats and epiphallus with two main fringed chords vs. smooth longitudinal pleats and smooth proximal epiphallus) as well as the remarkable differences concerning shell morphology (see also Liberto et al. 2016: 372 for a comparative discussion with conspecific taxa) we propose the population from Monte Kumeta as *S.calcaraeparajatinensis* ssp. nov.

Two additional new subspecies of *Siciliariacalcarae* will be described in the following sections: *S.calcaraeborgettensis* ssp. nov. and *S.calcaraecruenta* ssp. nov. Both taxa, despite showing close phylogenetic relationships with the other *Siciliariacalcarae* taxa, present distinctive shell and genital characters strong enough to propose their description as new subspecies.

##### ﻿ Siciliaria calcarae calcarae

Taxon classificationAnimaliaStylommatophoraClausiliidae

(Philippi, 1844)

DAB9B5EC-3029-5FB6-B18E-47D2E9F039A2

Figs 1.F, 14.1–14.10, 15.1–15.17, 16.1–16.6, 24.1, 24.2

Clausilia calcarae Philippi 1844: 107.
Clausilia adelina Küster 1847: 298.
Clausilia adelina – Benoit 1876: 152.
Clausilia brugnoneana Pini 1884: 379.
Clausilia calcarae var. nodosa Westerlund 1892: 48.
Clausilia adelina var. subsolida Monterosato 1892: 28.
Delima (Siciliaria) calcarae – Wagner 1925: pl. 3, fig. 25.
Charpentieria calcarae – Beckmann 2004: 188.
Siciliaria calcarae – Welter-Schultes 2012: 338.
Siciliaria calcarae – Nordsieck 2013b: 7.
Siciliaria (Siciliaria) calcarae calcarae – Liberto et al. 2015: 489.
Siciliaria (Siciliaria) calcarae calcarae – Liberto et al. 2016: 372.
Charpentieria calcarae – De Mattia 2017d.

###### Specimens examined.

Italy, Sicily, San Vito lo Capo, Castelluzzo, west cliffs E of town, 120 m asl, 38°6'25.71"N, 12°44'33.37"E, [Lab ID 61_1, COI: MW758908, ITS2: MW757140MW757141; Lab ID 61_2, COI: MW758909, ITS2: MW757142MW757143MW757144], W. De Mattia and J. Macor leg., 14.iv.2017. 2 dissected spm. Italy, Sicily, Castellammare del Golfo, Visicari, 395 m asl, 38°02'54.18"N, 12°48'26.61"E, [Lab ID 59_1, COI: MW758905, ITS2: MW757068MW757069MW757085], W. De Mattia and J. Macor leg., 15.iv.2017. 2 dissected spm. Italy, Sicily, Alcamo, Monte Bonifato, top of the mountain, 640 m asl, 37°57'29.40"N, 12°57'33.64"E, [Lab ID 5_1, COI: MW758925, ITS2: MW757125, MW757126, MW757127], I. Niero leg., 15.vi.2010. 2 dissected spm. Italy, Sicily, Alcamo, Monte Bonifato, top of the mountain, 640 m asl, 37°57'29.40"N, 12°57'33.64"E, A. Margelli leg., 15.vi.2010. 2 dissected spm. Italy, Sicily, Alcamo, Monte Bonifato, west side of the mountain, over the quarry, 550 m asl, 37°57'16.92"N, 12°58'9.06"E, W. De Mattia and J. Macor leg., 10.iv.2017. 2 dissected spm. Italy, Sicily, Castellammare del Golfo, Castello di Baida, W of the town along the road to Visicari, 300 m asl, 38°2'41.64"N, 12°48'14.34"E, W. De Mattia and J. Macor leg., 15.iv.2017. 2 dissected spm. Italy, Sicily, Piana degli Albanesi, 500 m south of Portella Ginestra, northern cliffs of Monte Kumeta, 970 m asl, 37°58'13.35"N, 13°15'22.06"E, W. De Mattia and J. Macor leg., 18.iv.2017. 2 dissected spm. Italy, Sicily, Calatafimi, Castello Eufemio, 395 m asl, 37°57'45.67"N, 12°51'21.13"E, W. De Mattia and J. Macor leg., 18.vi.2020. 2 dissected spm.

###### Shell

**(Figs 14.1–14.10, 15.1–15.17, 24.1–24.2).** Shell not decollate; whorls striated, with sutural papillae; dorsal keel indistinct or missing; inferior lamella moderately high or low; anterior upper palatal plica present, mostly separated from upper palatal plica, rarely lower anterior upper palatal plica present; palatal edge of clausilium plate distally not receding, palatal edge distally more or less strongly bent upwards (Nordsieck 2013b).

###### Measurements

**(n = 50, not decollate).** shell height 19.7 ± 0.8, whorl width 4.3 ± 0.3, aperture height 4.1 ± 0.2, aperture width 2.6 ± 0.1.

###### External morphology of the genital organs

**(Figs 14.1, 14.3, 14.5, 14.7, 14.9).** The FO is slim and long. The ratio FO/V ranges from 0.9 to 1.8. The VD is very thin along its whole course. The FDBC is shorter or longer than the BC+SDBC (FDBC/BC+SDBC) ranges from 0.9 to 1.8. The BC+SDBC can be either cylindrical or club-like, with either a pointed or a blunt apex. Its ratio with the V (BC+SDBC/V) ranges from 0.9 to 1.8. The transition between the BC and then SDBC can be clearly visible or almost indistinguishable. The D is moderately to extremely long, with a ratio with the V (D/V) that ranges from 1.3 to 4.1. It is thinner than the BC+SDBC, cylindrical in shape, with a rounded thin apex. The ratio D/BC+SDBC ranges from 1.4 to 2.5. The V is thick, cylindrical or hourglass-like in shape and shorter than the PC. The A can be short and narrow or large and long. The PC is longer than the V, with a range (P+E/V) that goes from 2.1 to 4.8. The P is wider than the V, cylindrical or distally swollen. The PR can be both long and thin or short and strong. The distinction between P and E with a visible ER is not always present as it appears randomly among the populations. The E is thinner than the P and can be slightly shorter or longer than the P, with a ratio (E/P) that ranges from 0.9 to 3.0. The actual transition area between E and the VD can be clearly visible or almost indistinguishable and it is clearly visible only from inside.

###### Internal morphology of the genital organs

**(Figs 14.2, 14.4, 14.6, 14.8, 14.10).** The internal wall of the A presents a variety of sculpture. It can be almost smooth, or with the distal part of the penial longitudinal pleats that gradually fade. Otherwise, it presents longitudinal broad and poorly elevated fleshy pleats or an irregular pattern of fleshy lumps. The internal sculpturing of the P can be distinguished in two main arrangements. The first is represented by 3 to 6 longitudinal fleshy smooth or irregularly segmented pleats that reach the A. The second arrangement presents large fleshy transverse pleats that are interrupted in the median part of the internal P. These pleats become smaller and continuous as approaching the A. The PP is big, elongated or nipple-like/roundish in shape, smooth, with a rounded apex and sometimes slightly depressed in the middle. The P-E transition presents three different structures among the examined populations. The populations from the top of Monte Bonifato and Castelluzzo present one ER with the PP originating from it. The ELP are not connected with the ER. The epiphallar formula is: 1ER(PP)+ELP. The populations from Monte Bonifato, from Monte Bonifato near the quarry and Visicari present one ER with both the PP and the ELP originating from it. The epiphallar formula is: 1ER(PP+ELP). Two additional populations from Portella Ginestra and Castello di Baida present a slightly more complex structure, namely a first distal ER with the PP originating from it and the ELP originating from a second proximal ER. The epiphallar formula is: 1ER(PP)+2ER(ELP).

**Figure 14.**
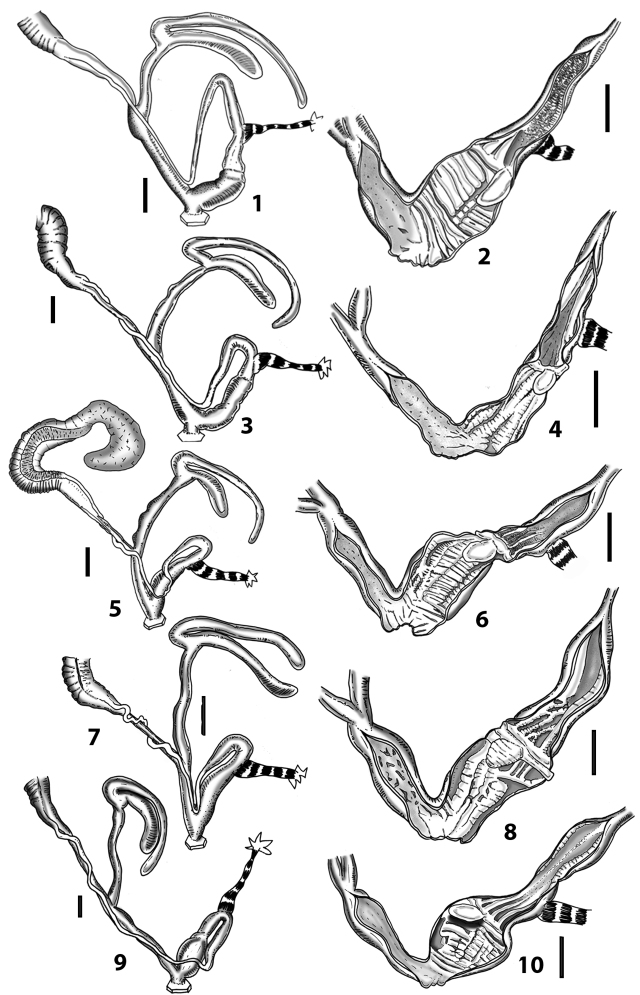
*Siciliariacalcaraecalcarae* (Philippi, 1844), Visicari, Castellammare del Golfo **14.1** whole distal genital organs **14.2** internal distal part of genital organs. Castelluzzo, San Vito Lo Capo **14.3** whole distal genital organs **14.4** internal distal part of genital organs. Castello di Baida, Castellammare del Golfo **14.5** whole distal genital organs **14.6** internal distal part of genital organs. Piana delle Ginestre, Monte Kumeta **14.7** whole distal genital organs **14.8** internal distal part of genital organs. Calatafimi, Castello Eufemio. (= *adelina* Küster, 1847) **14.9** whole distal genital organs **14.10** internal distal part of genital organs.

**Figure 15.**
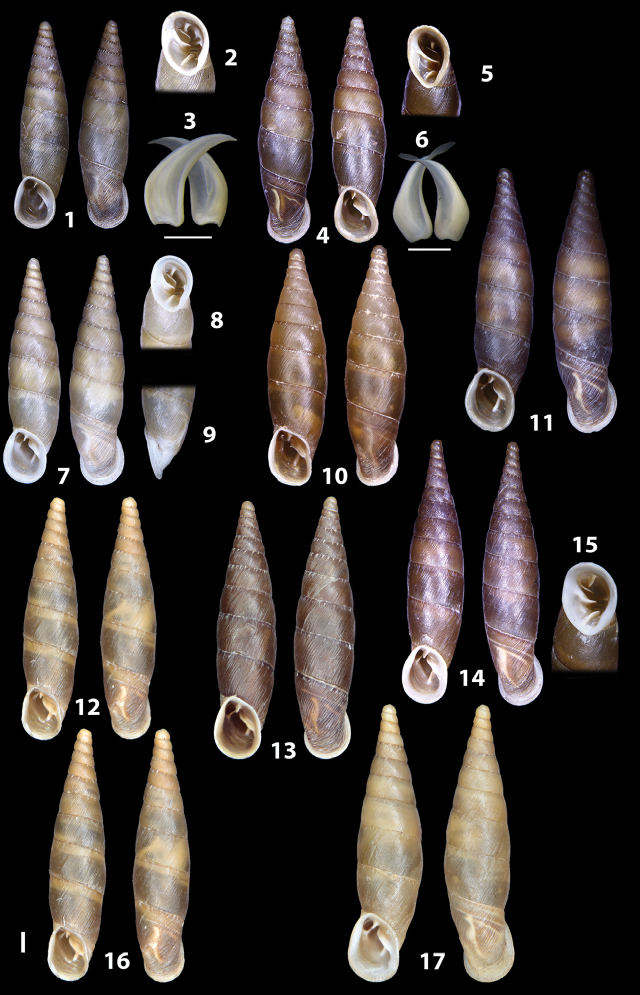
*Siciliariacalcaraecalcarae* (Philippi, 1844), Visicari, Castellammare del Golfo **15.1** shell **15.2** detail of the aperture **15.3** clausiliar plate double side. Castelluzzo, San Vito Lo Capo **15.4** shell **15.5** detail of the aperture **15.6** clausiliar plate double side. Castello di Baida, Castellammare del Golfo **15.7** shell **15.8** detail of the aperture **15.9** detail of the columellar side of last whorl. Monte Bonifato, Alcamo **15.10–15.12** shells. Calatafimi, Castello Eufemio, (= *adelina* Küster, 1847) **15.13** shell. Piana delle Ginestre, Monte Kumeta **15.14** shell **15.15** detail of the aperture. Monte Erice, western slopes **15.16** shell. Scopello **15.17** shell.

The E internal sculpturing presents a variety of arrangements, as: two main large smooth or fringed longitudinal pleats that gradually disappear toward the distal origin of the vas deferens or 4 thin smooth longitudinal pleats that abruptly disappear turning into an irregular texture of small dense papillae. The V can be either smooth and showing a very fine granulation or showing a coarse chevron pattern made of large fleshy pleats, merging together along the median longitudinal axis.

###### Spermatophore

**(Figs 16.3–16.4).** The spermatophore is thin and elongated. It is 6.7 mm long and 0.7 mm wide at its widest point. The tail and the head are missing as probably already digested. The upper and the lower keels are simple and run throughout the known length of the spermatophore. The lower keel is half as tall as the upper one.

###### Ecology.

*Siciliariacalcaraecalcarae* was found on south exposed limestone walls (Alcamo, Monte Bonifato), shady and humid north exposed limestone walls (Piana degli Albanesi, 500 m south of Portella Ginestra), shady habitats on decaying woods and tree trunks of *Quercusilex* (Castelluzzo, west cliffs E of town) or among grass and shrub nearby a cave entrance (Castellammare del Golfo, Visicari). *Siciliariacalcaraecalcarae* appeared to be adapted to many different habitats and niches and it is not an obliged rock-dwelling taxon. According to De Mattia (2017d), *Siciliariacalcarae* is Least Concern. The nominate subspecies is common and widespread all over its range and it is somewhere replaced by local rib/striated forms.

###### Distribution.

*Siciliariacalcaraecalcarae* presents the widest distributional area among the species of the genus *Siciliaria*. It is found from Trapani-Erice in the west to Montagna Grande and Bagheria in the east and Calatafimi-Piana degli Albanesi-Castelvetrano in the south. It is also known from the islands of Favignana and Levanzo (Nordsieck 2013b; Liberto et al. 2016: 382).

###### Remarks.

*Siciliariacalcaraecalcarae* shows minor shell differences among the populations which were described by the introduction of new names, such as a well-developed whole UPP (in Clausiliaadelinavar.subsolida), a well-developed AUPP in *Clausiliaadelina*) or the presence of a knob as a second AUPP (Clausiliacalcaraevar.nodosa) (Liberto et al. 2016: 382).

##### ﻿ Siciliaria calcarae belliemii

Taxon classificationAnimaliaStylommatophoraClausiliidae

(Brandt, 1961)

0FD3497A-9F6A-56FB-806D-CF1C3379BDD4

Figs 1.F, 16.7–16.11, 17.1–17.6, 24.4

Siciliaria (Siciliaria) calcarae belliemii Brandt 1961: 9.
Charpentieria calcarae belliemii – Nordsieck 2007: 53.
Siciliaria calcarae belliemii – Nordsieck 2013b: 8.
Siciliaria (Siciliaria) calcarae belliemii – Liberto et al. 2016: 371.

###### Specimens examined.

Italy, Sicily, Partinico, W side of the Mount Belliemi, 440 m asl, 38°00'22.47"N, 13°6'37.79"E, [Lab ID 54_1, COI: MW758900, ITS2: MW757134, MW757135, MW757136; Lab ID 54_2, COI: MW758901], W. De Mattia and J. Macor leg., 12.iv.2017. 15 live spm, 3 dissected spm. Italy, Sicily, Alcamo, E side of the Calatubo Castle, 75 m asl, 38°00'54.80"N, 12°59'13.35"E, [Lab ID 44_1, COI: MW758889, ITS2: MW757122, MW757123; Lab ID 44_2, COI: MW758913; Lab ID 44_3, COI: MW758930; Lab ID 44_4, COI: MW758931], W. De Mattia and J. Macor leg., 14.iv.2017. 18 live spm, 2 dissected spm.

###### Shell

**(Figs 17.1–17.6, 24.4).** not decollate; whorls ribbed, with sutural papillae; dorsal keel indistinct or missing; inferior lamella moderately high to low; anterior upper palatal plica present, separated from upper palatal plica, sometimes lower anterior upper palatal plica present; palatal edge of clausilium plate distally not receding, palatal edge distally more or less strongly bent upwards.

###### Measurements

(n = 40, not decollate, Monte Belliemii): shell height 17.6 ± 1.2, whorl width 3.1 ± 0.1, aperture height 2.9 ± 0.2, aperture width 2.0 ± 0.1. (n = 22, not decollate, Calatubo): shell height 19.6 ± 1.4, whorl width 3.9 ± 0.2, aperture height 3.4 ± 0.2, aperture width 3.0 ± 0.2.

###### External morphology of the genital organs

**(Figs 16.7, 16.9).** The FO is slim and long. The ratio FO/V ranges from 2.0 to 2.2. The VD is very thin along its whole course. The ratio of the BC+SDBC with the BC+SDBC+the second duct (FDBC/BC+SDBC) ranges from 1.4 to 2.1. The BC+SDBC can be either cylindrical or club-like, with either a pointed or a blunt apex. It is slightly shorter or as long as the V, with a ratio (SDBC+BC/V) that ranges from 0.9 to 1.0. The transition between the BC and then SDBC is little visible or almost indistinguishable. The D is long, with a ratio with the V (D/V) that ranges from 2.9 to 3.1. It is thinner or as thin as the SDBC+BC, cylindrical in shape with a rounded thin apex. The ratio D/BC+SDBC ranges from 2.1 to 2.4. The V is short and cylindrical. The A is short but wide. The PC is much longer than the V, with range (PC/V) 2.8–3.1. The P is wider than the V, cylindrical or distally swollen. The PR is robust. The E is thin and slightly shorter or as long as the P, with the ratio E/P ranging 0.9–1. It gradually shrinks and turns into the VD. The actual transition area between E and the VD can be clearly visible or almost indistinguishable and it is clearly visible only from inside.

###### Internal morphology of the genital organs

**(Figs 16.8, 16.10, 16.11).** The A shows 3–5 large longitudinal pleats oriented towards the P. The P presents 4–6 longitudinal fleshy and smooth pleats, gradually narrowing towards the A. Sometimes the lower pleats gradually become irregularly segmented. The fine structure of the wall is smooth. The PP is big, nipple-like in shape, smooth with a rounded apex. The P-E transition presents two slightly different structures in the two examined populations. The population from Monte Belliemi (type locality) presents a first distal ER, the PP originates from the second proximal ER. The ELP are not connected to the second proximal ER. The epiphallar formula is: 1ER+2ER(PP)+ELP. The population from Calatubo presents a first distal ER, while the PP and ELP originate from the second proximal ER. The epiphallar formula is: 1ER+2ER(PP+ELP). The E shows 4–6 irregular metameric longitudinal and irregular pleats that gradually disappear toward the distal origin of the vas deferens. The V shows a coarse chevron pattern made of large fleshy pleats, merging together along the median longitudinal axis or sometimes presents an irregular pattern made of small irregular fleshy pleats.

**Figure 16.**
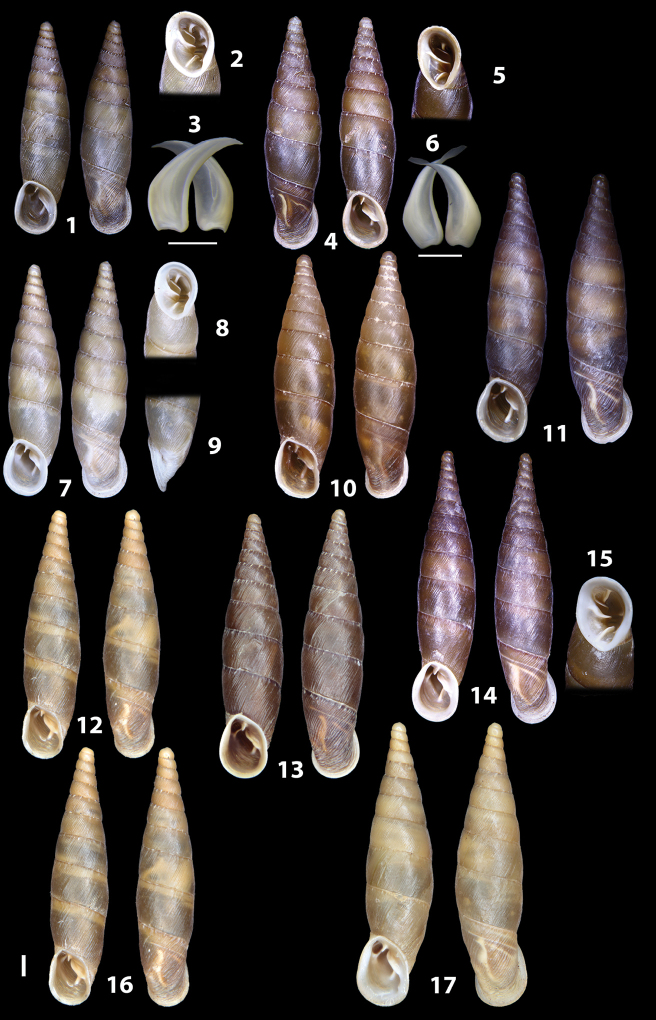
*Siciliariacalcaraecalcarae* (Philippi, 1844), Monte Bonifato **16.1** whole distal genital organs **16.2** internal distal part of genital organs **16.3** cross section of the spermatophore **16.4** spermatophore **16.5** whole distal genital organs **16.6** internal distal part of genital organs. *Siciliariacalcaraebelliemii* (Brandt, 1961), Monte Belliemi, Partinico **16.7** whole distal genital organs **16.8** internal distal part of genital organs. *Siciliariacalcaraebelliemii* (Brandt, 1961), Castello Calatubo, Alcamo **16.9** whole distal genital organs **16.10** internal distal part of genital organs **16.11** penial pseudopapilla.

**Figure 17.**
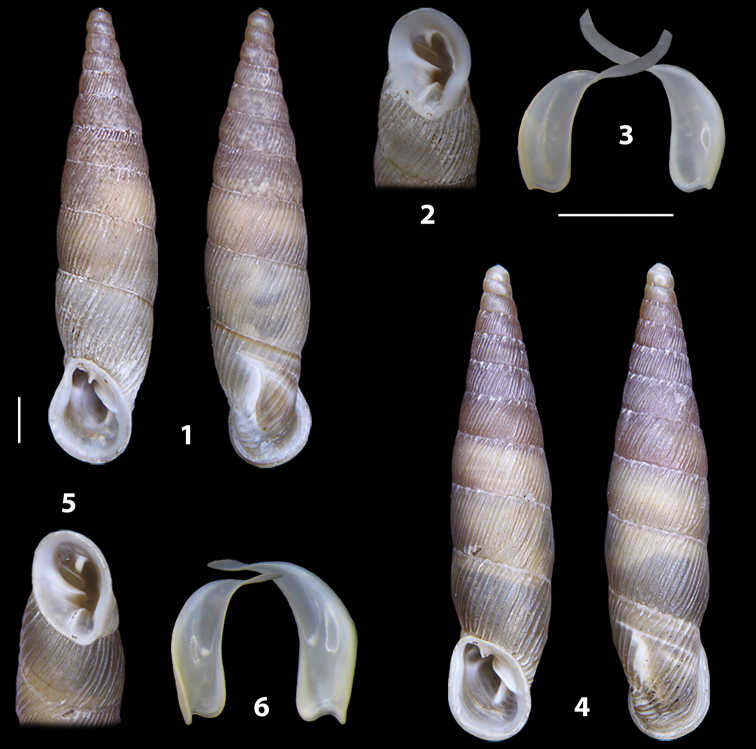
*Siciliariacalcaraebelliemii* (Brandt, 1961), Monte Belliemi, Partinico **17.1** shell **17.2** detail of the aperture **17.3** clausiliar plate double side. Castello di Calatubo, Alcamo **17.4** shell **17.5** detail of the aperture **17.6** clausiliar plate double side.

###### Ecology.

The subspecies inhabits both scattered isolated boulders (Monte Belliemi) or limestone cliffs (Calatubo Castle). This subspecies is known only from two localities with very limited size, although it is abundant. Both localities are not included in protected areas.

###### Distribution.

*Siciliariacalcaraebelliemii* is known from Monte Belliemi near Partinico and from the limestone cliffs of the Calatubo Castle near Alcamo.

###### Remarks.

The Calatubo population is bigger than the population from the type locality (Monte Belliemi). The average shell heights are 19.6 ± 1.4 vs. 17.6 ± 1.2. The two populations occupy remarkably different ecological niches: high cliffs vs isolated small boulders scattered throughout open fields. Could the different ecology of the two populations be the origin or cause of such different dimensions of the shell? This interesting hypothesis could be tested also including other *Siciliariacalcarae* ssp. populations found both on limestone cliffs or different niches as tree’s barks (Visicari, Castelluzzo and Ficuzza).

##### ﻿ Siciliaria calcarae borgettensis ssp. nov.

Taxon classificationAnimaliaStylommatophoraClausiliidae

0CC45FED-6686-573A-B77C-B81B8A3DC2E5

http://zoobank.org/A092D144-BDF7-4F35-A104-5E8AE27B1BD8

Figs 1.F, 18.1–18.2, 19.1–19.5, 24.5, 24.6

###### Type locality.

Italy, Sicily, Municipality of Borgetto, Anime Sante hill, road to Romitello, 400 m asl, 38°02'59.53"N, 13°08'58.55"E.

###### Type material.

1 ***Holotype*** (NHMW 113613) [Lab ID 46_1, COI: MW758890, ITS2: MW757080, MW757089, MW757090] and two ***Paratypes*** (NHMW 113614): Italy, Sicily, Municipality of Borghetto, Anime Sante hill, road to Romitello, 400 m asl, 38°02'59.53"N, 13°08'58.55"E, [Lab ID 46_2, COI: MW758891; Lab ID 46_3, COI: MW758932], W. De Mattia and J. Macor leg., 22.vi.2015. 3 dissected spm. 3 ***Paratypes*** (CWDM 18223): same locality.

###### Shell diagnosis.

Shell not decollate; whorls rib-striated; dorsal keel not distinguishable; inferior lamella very high; anterior upper palatal plicae present detached from the lunella; parietalis short partially overlapping of the spiralis; palatal edge of clausilium plate distally receding, plate gutter-like narrowed, palatal edge against distal end bent upwards and more or less pointed.

###### Shell description

**(Figs 19.1–19.5, 24.5, 24.6).** The shell is elongated, fusiform to slightly pyriform, sinistral and not decollate. It is light reddish-brown in colour. The external surface is irregularly ribbed and striated. The spire is slowly and regularly growing with 9 ½–10 ¼ slightly convex whorls. The sutures are shallow with whitish papillae, mainly present along the 4^th^–8^th^ whorls. The whitish papillae become much less dense along the first four whorls of the teleoconch. The suture is dark red in colour, forming a darker line along it. The basal and the cervical keels are almost indistinguishable or not present. The umbilicus is closed. The aperture is ~ ^1^⁄_4_ of shell height and subovoid in shape. The PRI is long and moderately raised, wider along its posterior part and not fused with the L. The PRI is not visible from a frontal view of the aperture. The L is anterior, with a very reduced, knob-like PUPP connected to it. The AUPP is strong, detached from the L and well visible from the aperture. The BAS starts directly from the L and it is long and strong, clearly visible from the aperture. The SCL is vestigial to absent. The IL is very high. The SUL is tooth-like and partially overlaps with the spiralis. The SCOL is not emergent. The peristome is continuous, markedly thickened and reflected. It is not superiorly fused to the wall of the first whorl. The palatal edge of the clausilium is distally receding and bent upwards. The plate is narrow and gutter-like. The palatal edge against distal end is bent upwards and more or less blunt.

###### Measurements.

***Holotype***: not decollate shell height 18.7, whorl width 4.2, aperture height 3.9, aperture width 2.8. ***Paratypes*** (n = 20, not decollate): shell height 19.0 ± 0.3, whorl width 4.3 ± 0.1, aperture height 3.8 ± 0.1, aperture width 2.9 ± 0.1.

###### External morphology of the genital organs (Fig. 18.1).

The FO is as long as or slightly longer than the V (FO/V range 1.0–1.1). The VD is thin along its whole course. The FDBC is longer than the BC+SDBC (FDBC/BC+SDBC range 1.5–1.8). The BC+SDBC is club-like to cylindrical and as long as the V (BC+SDBC/V = 1.0), with no clear distinction between the SDBC and the BC. The apex is big and rounded. The D is longer than the V (D/V range 2.2–2.7) and longer that the BC+SDBC (D/BC+SDBC range 2.1–2.5), slightly thinner than BC+SDBC and with a small and round apex. The V is cylindrical. The A is very large. The PC is longer than the V (PC/V range 2.1–2.2). The PR is long and robust. The transition P-E does not show any ET. The E is almost as long as the P (E/P range 0.8–0.9), gradually shrinking and turning into the VD. The transition area between the E and the VD is not clearly visible outside.

###### Internal morphology of the genital organs

**(Fig. 18.2).** The A shows a set of irregular fleshy folds. The P presents four to five large longitudinal fleshy segmented pleats. These pleats distally become weak and irregular entering in the A. The fine structure of the penial wall is smooth. The PP is big, rhombus-shaped and smooth. The P-E transition presents one ER with the PP originating from it. The ELP are not connected with the ER. The epiphallar formula is: 1ER(PP)+ELP. The E shows a pattern of 3 to 4 irregular longitudinal extremely fringed pleats. These pleats abruptly fade and most of the epiphallus is smooth. The V is smooth with a finely granulated surface.

###### Comparative and taxonomical remarks.

*Siciliariacalcaraeborgettensis* ssp. nov. was reported as “Sagana form” in Nordsieck (2013b: 8; personal communication), which differs from the nominate subspecies by a higher inferior lamella, a lower anterior upper palatal plica more or less developed and the palatal edge of the clausilium less strongly bent upwards. The type locality (Anime Sante hill along the Borghetto-Romitello road) is very close to Sagana, which is found 3.5 km eastward. This taxon is similar to the *Siciliariacalcaraecruenta* ssp. nov. (described below) from Monte Gibilmesi, that is also found at short distance from Sagana (1.5 km northeast) but differs from the latter by its more striated shell, lighter colour and the more developed AUPP which is always visible from the frontal view. *Siciliariacalcaraecruenta* ssp. nov. belongs to haplogroup 2, whereas *Siciliariacalcaraeborgettensis* ssp. nov. belongs to haplogroup 1.

###### Distribution.

*Siciliariacalcaraeborgettensis* ssp. nov. is exclusively known from the type locality. It is likely to be present along the whole northern limestone slopes of the Anime Sante hill (Romitello) as far as Sagana, thus further field investigations are needed in order to clearly define its actual distribution range.

###### Ecology.

The subspecies is found on limestone boulders and under scattered rocks.

###### Etymology.

*Siciliariacalcaraeborgettensis* ssp. nov. is named after the nearby Borgetto town (Borgetto-Partinico), where the new subspecies was discovered.

##### ﻿ Siciliaria calcarae jatinensis ssp. nov.

Taxon classificationAnimaliaStylommatophoraClausiliidae

1D21D914-3D83-5959-AF87-5B8EB23B28EC

http://zoobank.org/56D36819-7A84-41DE-8820-966CB683B61E

Figs 1.F, 18.3–18.7, 19.6–19.8, 24.7–24.8

###### Type locality.

Italy, Sicily, San Giuseppe Jato, quarry E of the town, 630 m asl, 37°58'15.07"N, 13°12'2.23"E.

###### Type material.

1 ***Holotype*** (NHMW 113615) [Lab ID 34_2, COI: MW758879, ITS2: MW757118, MW757119, MW757120, MW757121] and 5 ***Paratypes*** (NHMW 113616): Italy, Sicily, San Giuseppe Jato, quarry E of the town, 630 m asl, 37°58'15.07"N, 13°12'2.23"E, [Lab ID 34_1, COI: MW758878; Lab ID 34_3, COI: MW758926; Lab ID 34_4, COI: MW758927],W. De Mattia and J. Macor leg., 22.iv.2017. 3 dissected spm. 8 ***Paratypes*** (CWDM 18224): same locality.

###### Shell diagnosis.

Shell decollate, rarely not decollate; whorls ribbed; dorsal keel weak but distinguishable; inferior lamella very high; anterior upper palatal plicae present and detached from the lunella; parietalis long; palatal edge of clausilium plate distally receding, plate gutter-like, narrowed, palatal edge against distal end bent upwards and more or less pointed.

###### Shell description

**(Figs 19.6–19.8, 24.7, 24.8).** The shell is elongated, fusiform, sinistral, decollate but rarely not decollate. It is reddish-brown in colour. The external surface is regularly ribbed. The spire is slowly and regularly growing with (decollate) 8 to 9 ¼ slightly convex whorls. The sutures are moderately deep with whitish papillae present all along the teleoconch, moderately denser along the last whorls. The basal and the cervical keels are distinguishable. The umbilicus is closed. The aperture is ~ ^1^⁄_5_ of shell height and roundish to subovoid in shape. The PRI is short and ends at the level of the L. It is not fused with the L. The PRI is not visible from a frontal view of the aperture. The L is dorso-lateral, thick and somehow irregular. There is a very reduced, knob-like PUPP connected to it. The AUPP is strong to moderately weak, detached from the L and barely visible from the aperture. The BAS starts directly from the L and it is long and strong, well visible from the aperture. The SCL is absent. The IL is high to very high. The SUL is tooth-like, long and partially overlaps with the SPL. The SCOL is not emergent. The peristome is continuous, markedly thickened and reflected. It is not superiorly fused to the wall of the first whorl. The palatal edge of the clausilium is distally receding and bent upwards. The plate is narrow and gutter-like. The palatal edge against distal end is bent upwards and more or less blunt.

###### Measurements.

***Holotype***: not decollate shell height 20.3, whorl width 4.3, aperture height 3.8, aperture width 2.4. ***Paratypes*** (n = 25, decollate): shell height 20.9 ± 1.0, whorl width 4.4 ± 0.1, aperture height 3.9 ± 0.2, aperture width 2.4 ± 0.2.

###### External morphology of the genital organs

**(Figs 18.3, 18.6).** The FO is longer than the V with a FO/V ratio ranging from 2.0 to 2.1. The VD is thin along its whole course. The ratio of the FDBC with the BC+SDBC (FDBC/BC+SDBC) ranges from 1.1 to 1.3. The BC+SDBC is club-like and longer than the V, with a blunt apex. It is slightly longer than the V, with a ratio (BC+SDBC/V) that ranges from 1.6 to 1.8. There is a clear distinction between the SDBC and the BC. The D is longer than the V (with a D/V ranging from 2.1 to 2.3) and slightly longer than BC+SDBC (D/BC+SDBC ratio 1.2–1.3). It is thinner than the BC+SDBC and with a small and round apex. The V is big and cylindrical. The A is large. The PC is longer than the V (P+E/V ratio 2.1–2.4). The PR is very short and robust. The ET is weak. The E is longer than the P (E/P ratio 1.1–1.2) and almost abruptly shrinking and turning into the VD.

###### Internal morphology of the genital organs

**(Figs 18.4, 18.7).** The A shows few irregular pleats. The P presents 4 to 6 heavily segmented longitudinal pleats that begin directly from the PP and run as far as the A, becoming irregular as approaching their distal sections. The penial wall is smooth. The PP is big, rounded to rhomboid, smooth with a round to blunt apex. The P-E transition presents a first distal ER, while both the PP and ELP originate from the second proximal ER. The epiphallar formula is: 1ER+2ER(PP+ELP). The E shows a pattern of 4 to 6 smooth longitudinal pleats. These pleats merge one into another forming two main smooth pleats that run as far as the vas deferens. The V shows a weak irregular pattern of smooth pleats.

###### Comparative and taxonomical remarks.

The shell of *Siciliariacalcaraejatinensis* ssp. nov. is very similar to the shells of *Siciliariacalcaraebelliemii* and *Siciliariacalcaraeparajatinensis* spp. nov. The three taxa have markedly ribbed shells. The density of the ribs along the body whorl is similar, with overlapping ranges: 32 ± 5 for *Siciliariacalcaraebelliemii* (n = 25), 35 ± 6 for *Siciliariacalcaraejatinensis* ssp. nov. (n = 21) and 34 ± 5 for *Siciliariacalcaraeparajatinensis* spp. nov. (n = 15). The PRI are short and stop at the level of the L, whereas the AUPP is strong to moderately weak and detached from the L. Slight differences are observed concerning the dimensions and the colour of the shell: *Siciliariacalcaraejatinensis* ssp. nov. is dark reddish to dark brown with an average height of 20.9 ± 1.0 whereas *Siciliariacalcaraeparajatinensis* ssp. nov. (average height: 21.7 ± 1.4) and *Siciliariacalcaraebelliemii* (average height: 17.6 ± 1.2, type locality) are light yellowish to light brown. The three subspecies show just slight differences in their anatomy of the genital organs, which fit into the intraspecific variability of *S.calcarae* and do not allow a certain separation. The different subspecific status of these populations is mainly supported by the phylogenetic results in connection to their patchy and isolated distribution. *Siciliariacalcaraejatinensis* ssp. nov. is included in the haplogroup 2 whereas *Siciliariacalcaraeparajatinensis* n. spp. and *Siciliariacalcaraebelliemii* both belong to haplogroup 1.

Nordsieck (2013a) recently supplemented his *Siciliaria* web article (May 2021) after receiving samples we collected from Monte Jato (*S.calcaraejatinensis* ssp. nov.) and the W side of Monte Kumeta (*S.calcaraeparajatinensis* ssp. nov.), considering these populations as belonging to the type form of *Siciliariatiberii* and, more precisely, “to the southern and eastern form with less strong dorsal keel”. Nordsieck (2013b, supplemented 2021) justifies his identification stating that “There is no doubt that *Clausiliatiberii* A. Schmidt (1868: 42–43) belongs to the southern form” despite not justifying or explaining why “there is no doubt”, considering also that no locality is provided in Schmidt’s description.

Nordsieck (2013b, supplemented 2021) stated that “Originals from Benoit (SMF 67544, 96458, see also Benoit 1859: pl. 6, fig. 7) do not differ from the form from Monte Kumeta”. Following the caption of fig. 10 “M. della Scala” was wrongly identified by Nordsieck as Monte Kumeta. Information provided by locals during field collections allowed us to ascertain that Monte della Scala is an old, almost forgotten toponym for Monte Maganoce. Monte Maganoce was once named “Monte della Scala” because of the church called “Madonna della Scala” was located along its slopes.

Moreover, in the text Nordsieck cited samples SMF 67544, 96458, while in fig. 10 the caption indicated the sample as “N 10008". Thus, it is not clear if the depicted specimen originated from Benoit or from a different source and kept in Nordsieck´s collection.

Monte Kumeta was also deemed by Nordsieck as a spelling mistake for Monte della Fiera (pers. comm., April 2021) and stating that “form from Monte della Fiera belongs to the northern subspecies (of *S.tiberii*), based on SMF samples”. Monte della Fiera (37°59'47.90"N, 13°8'48.22"E) is 10 km distant from Monte Kumeta and represents the eastern continuation of Monte Belliemi. It is not clear what the “northern subspecies” may be, but probably it is referred as the northern form of the nominate subspecies.

In the COI and combined tree (Fig. 4) *S.calcaraejatinensis* ssp. nov. is well embedded into *Siciliariacalcarae* subclade A2. The morphologically similar *S.calcaraeparajatinensis* ssp. nov. is also well embedded into *S.calcarae* clade in the COI tree.

We agree that the sample depicted in Fig. 10 is similar to *S.calcaraejatinensis* ssp. nov., but it is not clearly explained by Nordsieck (2013b; supplemented 2021) why it is supposed to belong to *S.tiberii*. To check this hypothesis, we included in our molecular genetic analysis two specimens of *S.tiberiitiberii* from Capo Rama (Terrasini) representing the “northern” form in both COI and concatenated trees, they fall well embedded within the subclade that comprises all (and exclusively) the remaining *S.tiberii* subspecific taxa.

Regarding shell morphology, we carefully browsed Schmidt’s description (1868: 41–43). Few important details better fit with the shell morphology of the “northern” type form of *Siciliariatiberii* (Figs 26.1, 26.3) rather than with the specimen depicted by Nordsieck (2013b, supplemented 2021, fig. 10) and the specimen we depicted in Figs 19.9–19.11. Schmidt refers to the more expanded peristome (“mehr umgeschlagene Mündung”), the deep dorsal keel (“sendet nach unten eine stark entwickelte Gaumenfalte ab, welche auf der, durch die tiefe, breite Nackenfurche gebildeten, inneren Wölbung steht”) and the deep palatal hollow. Other shell differences are detectable comparing Figs 19.9–19.11 and Figs 26.1, 26.3, the number and shape of the ribs of the neck and along the whorls, strength and visibility of the AUPP, the length of the PRI, the presence of a second lower AUPP in *S.calcaraeparajatinensis* ssp. nov., the strength and visibility of the sutural papillae, the depth of the sutures and the more convex whorls of *S.tiberiitiberii*. Thus, phylogenetic and morphological evidence prevent us from considering the Monte Jato and Monte Kumeta populations as belonging to *S.tiberii*.

###### Distribution.

*Siciliariacalcaraejatinensis* ssp. nov. is known only from the type locality: the abandoned quarry north of the town of San Giuseppe Jato. Its actual distribution range must be further investigated as it is probably present along the northwestern cliffs of the Monte Jato, E of the town of San Giuseppe Jato.

###### Ecology.

The new subspecies is an obliged limestone rock-dweller, exclusively collected on walls and cliffs, both natural or resulting from the previous quarrying activity.

###### Etymology.

*Siciliariacalcaraejatinensis* ssp. nov. is named after Monte Jato (San Giuseppe Jato), where the new subspecies was discovered.

##### ﻿ Siciliaria calcarae parajatinensis ssp. nov.

Taxon classificationAnimaliaStylommatophoraClausiliidae

1286D997-518E-557A-9D1D-46F95081F7BB

http://zoobank.org/BF415C49-9548-4ECB-8E6E-6236F8789AD4

Figs 1.F, 18.8–18.9, 19.9–19.12, 24.9–24.10

###### Type locality.

Italy, Sicily, Monreale, W part of Monte Kumeta toward Jato Antica, 630 m asl, 37°57'8.18"N, 13°13'14.57"E.

###### Type material.

1 ***Holotype*** (NHMW 113617) and 4 ***Paratypes*** (NHMW 113618): Italy, Sicily, Monreale, W part of Monte Kumeta toward Jato Antica, 630 m asl, 37°57'8.18"N, 13°13'14.57"E, W. De Mattia and J. Macor leg., 22.iv.2017. 3 dissected spm. (CWDM 18225): same locality.

###### Shell diagnosis.

Shell not decollate; whorls ribbed; dorsal keel weak but distinguishable; inferior lamella high or very high; anterior upper palatal plicae present and detached from the lunella; parietalis long; palatal edge of clausilium plate distally receding, plate gutter-like narrowed, palatal edge against distal end bent upwards and more or less pointed.

###### Shell description

**(Figs 19.9–19.12, 24.9, 24.10).** The shell is elongated, fusiform, sinistral, not decollate but sometimes decollate. It is light brown in colour. The external surface is regularly ribbed. The spire is slowly and regularly growing with (decollate) 9 ^1^/_2_–10 ^3^/_4_ slightly convex whorls. The sutures are shallow with whitish papillae present all along the teleoconch, moderately denser along the last whorls. The basal and the cervical keels are distinguishable. The umbilicus is closed. The aperture is ~ ^1^⁄_5_ of shell height and roundish to subovoid in shape. The PRI is short and its length slightly exceeds the L. It is not fused with the L. The PRI is not visible from a frontal view of the aperture. The L is dorsal. The PUPP is absent or vestigial. The AUPP is thin, detached from the L and barely visible from the aperture. The BAS starts directly from the L and it is long and strong, well visible from the aperture. The SCL is absent. The IL is high to very high. The SUL is tooth-like, very long and remarkably overlapping with the SPL. The SCOL is sometimes visible. The peristome is continuous, markedly thickened and reflected. It is not superiorly fused to the wall of the first whorl. The palatal edge of the clausilium is distally receding and bent upwards. The plate is narrow and gutter-like. The palatal edge against distal end is bent upwards and more or less blunt.

**Figure 18.**
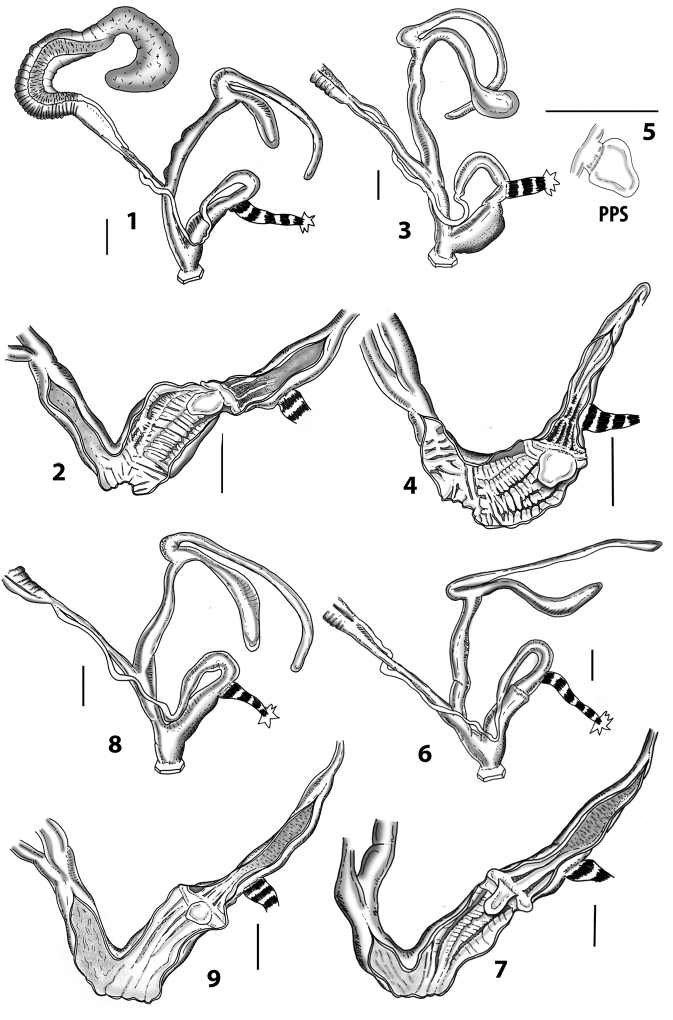
*Siciliariacalcaraeborgettensis* ssp. nov., road to Romitello, Borghetto **18.1** whole distal genital organs **18.2** internal distal part of genital organs. *Siciliariacalcaraejatinensis* ssp. nov. San Giuseppe Jato **18.3** whole distal genital organs **18.4** internal distal part of genital organs **18.5** detail of the pseusopapilla **18.6** whole distal genital organs **18.7** internal distal part of genital organs. *Siciliariacalcaraeparajatinensis* ssp. nov., west side Monte Kumeta **18.8** whole distal genital organs **18.9** internal distal part of genital organs.

###### Measurements.

***Holotype***: not decollate shell height 21.7, whorl width 4.2, aperture height 4.1, aperture width 3.3. ***Paratypes*** (n = 30, not decollate): shell height 21.7 ± 1.4, whorl width 4.4 ± 0.2, aperture height 4.2 ± 0.2, aperture width 3.1 ± 0.4.

###### External morphology of the genital organs

**(Fig. 18.8).** The FO is longer than the V (FO/V ratio 2.0–2.2). The VD is thin along its whole course. The FDBC is slightly longer than the BC+SDBC (FDBC/BC+SDBC ratio 1.0–1.2). The BC+SDBC is club-like and longer than the V (BC+SDBC/V ratio 1.4–1.6), with a clear distinction between the SDBC and the BC. The apex is very big and rounded. The D is slightly longer than the V (D/V ratio 2.1–2.2) and slightly longer that the BC+SDBC (D/BC+SDBC ratio 1.0–1.2), thinner than the BC+SDBC and with a small and round apex. The V is big and cylindrical. The A is large. The PC is longer than the V (P+E/V ratio 2.7–3.0). The PR is very short and robust. The ET is well visible. The E is longer than the P (E/P ratio 1.2–1.4) and gradually shrinking and turning into the VD.

###### Internal morphology of the genital organs

**(Fig. 18.9).** The A shows weak longitudinal pleats entering directly from the P. The P presents 4 to 6 smooth and weak longitudinal pleats. These pleats become irregular and weaker as approaching their distal sections. The PP is big, rounded to rhomboid, smooth with a round apex. The P-E transition presents a first distal ER, while the PP and ELP originate from the second proximal ER. The epiphallar formula is: 1ER+2ER(PP+ELP). The E distally presents a variable set of irregular smooth small pleats the abruptly vanish. The remaining E shows a coarse pattern of finely granulated wall. The V shows a smooth and fine granulation, with no pleats or folds.

###### Comparative and taxonomical remarks.

*Siciliariacalcaraeparajatinensis* ssp. nov. in the COI tree is well embedded within the *calcarae* subclade and closely related to the nominate subspecies (Fig. 4). For a further differential comparative analysis see the comparative and taxonomical remarks of *Siciliariacalcaraejatinensis* ssp. nov.

###### Distribution.

*Siciliariacalcaraeparajatinensis* ssp. nov. is exclusively known from the type locality, the western slopes of the Monte Kumeta. Further field research could extend its distributional range to the whole western side of the Monte Kumeta (Monreale) and surrounding mountains. Following Nordsieck (2013b, supplemented 2021, Fig. 10) the range of *Siciliariacalcaraeparajatinensis* ssp. nov. can be extended also for Monte Maganoce (also known as Monte della Scala), W of Piana degli Albanesi. In the central-eastern part of Monte Kumeta massif the type form of *S.calcaraecalcarae* is known to occur.

###### Ecology.

*Siciliariacalcaraeparajatinensis* ssp. nov. is an obliged rock-dweller and inhabits limestone walls and boulders. The type locality, that is not included in any protected area, comprises a rich population.

###### Etymology.

*Para* = next to, considering strong morphological, genetic and distribution affinities with the newly described *Siciliariacalcaraejatinensis* ssp. nov.

**Figure 19.**
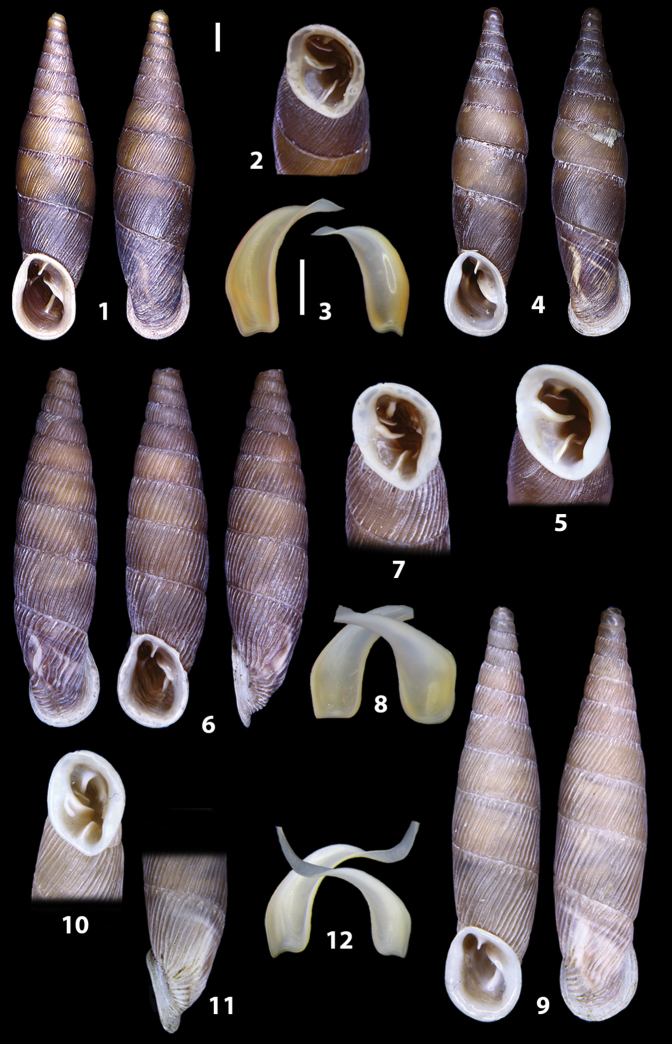
*Siciliariacalcaraeborgettensis* ssp. nov., road to Romitello, Borghetto **19.1** shell **19.2** detail of the aperture **19.3** clausiliar plate double side **19.4** shell **19.5** detail of the aperture. *Siciliariacalcaraejatinensis* ssp. nov. San Giuseppe Jato **19.6** shell **19.7** detail of the aperture **19.8** clausiliar plate double side. *Siciliariacalcaraeparajatinensis* ssp. nov., west side Monte Kumeta **19.9** shell **19.10** detail of the aperture **19.11** detail of the columellar side of last whorl **19.12** clausiliar plate double side.

##### ﻿ Siciliaria calcarae orlandoi

Taxon classificationAnimaliaStylommatophoraClausiliidae

Liberto, Reitano, Giglio, Colomba & Sparacio, 2016

60D81626-4E5B-59F6-AB03-8B759966EA79

Figs 1.F, 20.1–20.4, 21.1–21.5, 24.11

Siciliaria (Siciliaria) calcarae orlandoi Liberto, Reitano, Giglio Colomba and Sparacio 2016: 371.

###### Specimens examined.

Italy, Sicily, Monreale, Bosco Ficuzza, Ponte Arcere, 470 m asl, 37°55'46.42"N, 13°23'5.22"E, [Lab ID 36_1, COI: MW758921, ITS2: MW757132MW757133], W. De Mattia and J. Macor leg., 25.iv.2017. 4 dissected spm.

###### Shell

**(Figs 21.1–21.5, 24.11).** Shell not decollate; whorls striated, with sutural papillae; dorsal keel little distinct; lateral lunella; principal plica not fused with lunella; inferior lamella moderately high; anterior upper palatal plica reduced, separated from a very short upper palatal plica; palatal edge of clausilium plate distally not receding, palatal edge distally more or less strongly bent upwards.

###### Measurements

**(n = 12, not decollate).** shell height 18.0 ± 0.7, whorl width 4.1 ± 0.1, aperture height 3.8 ± 0.2, aperture width 2.7 ± 0.1.

###### External morphology of the genital organs

**(Fig. 20.1).** The FO is longer than the V (FO/V range 1.9–2.3). The VD is thin along its whole course. The FDBC is slightly shorter or as long as the BC+SDBC (FDBC/BC+SDBC range 0.9–1.0). The BC+SDBC is cylindrical to club-like and longer than the V (BC+SDBC/V range 3.3–3.6), with no distinction between the SDBC and the BC. The apex is big and round. The D is much longer than the V (D/V range 3.8–4.2) and longer that the BC+SDBC (D/BC+SDBC range 1.3–1.5), slightly thinner than the BC+SDBC and with a small rounded apex. The V is very short and cylindrical. The A is very large. The PC is longer than the V (PC/V range 3.0–3.3). The PR is long and robust. The ET is well visible. The E is slightly longer than the P (E/P range 1.2–1.3), almost abruptly shrinking and turning into the VD.

###### Internal morphology of the genital organs

**(Fig. 20.2).** The A shows a set of transverse fleshy folds as they represent the continuation of the penial sculpture. The P presents a set of transverse, interrupted fleshy smooth pleats. These pleats are laterally surrounded by two longitudinal small smooth pleats. The fine structure of the wall is smooth. The PP is oval, smooth and with a round apex. The P-E transition presents a first distal ER, while both PP and ELP originate from the second proximal ER. The epiphallar formula is: 1ER+2ER(PP+ELP). The E shows two main longitudinal fringed pleats that fade before the VD. The wall of the E is finely granulated. The V is almost totally smooth but the wall is also finely granulated.

###### Ecology.

Following Liberto et al. (2016: 372): ”*Siciliariacalcaraeorlandoi* lives under the bark of dead trees and in the leaf litter of woods vegetating both in sandstone (Bosco del Cappelliere, Diga Scanzano) and calcareous (Alpe Cucco, Rocca Busambra) soils; in these two last localities *S.calcaraeorlandoi* is found also on calcareous rocks into the woods”. We collected this subspecies on *Quercus* sp. barks close to a small creek.

###### Distribution.

*Siciliariacalcaraeorlandoi* in known to occur in the Nature Reserve of the Bosco della Ficuzza, Rocca della Busambra, Bosco del Cappelliere e Gorgo del Drago that is included in the wider Sicani Mountains Regional Nature Park (Liberto et al. 2016: 372).

##### ﻿ Siciliaria calcarae cruenta ssp. nov.

Taxon classificationAnimaliaStylommatophoraClausiliidae

9D283503-9AF8-52B4-A483-50C798867810

http://zoobank.org/BA270C4D-6DCE-46C0-BB8F-2CC3C55DE703

Figs 1.F, 20.3–20.4, 21.6–21.10, 24.12–24.14

###### Type locality.

Italy, Sicily, Monreale, N side of Monte Gibilmesi, 890 m asl, 38°03'37.03"N, 13°12'38.53"E.

###### Type material.

1 ***Holotype*** (NHMW 113619) [Lab ID 47_1, COI: MW758933, ITS2: MW757078, MW757079] and two ***Paratypes*** (NHMW 113620): Italy, Sicily, Monreale, N side of Monte Gibilmesi, 890 m asl, 38°03'37.03"N, 13°12'38.53"E, [Lab ID 47_2, COI: MW758934, ITS2: MW757077, MW757087, MW757088; Lab ID 47_3, MW758935; Lab ID 47_4, MW758936], W. De Mattia and J. Macor leg., 22.vi.2015. 4 dissected spm. 4 ***Paratypes*** (CWDM 18226): same locality.

###### Shell diagnosis.

Shell not decollate; whorls finely striated; dorsal keel absent or barely distinguishable; inferior lamella high or very high; anterior upper palatal plicae present but weak, detached from the lunella; parietalis very long; palatal edge of clausilium plate distally receding, plate somehow cylindrical, palatal edge against distal end bent upwards and more or less pointed.

###### Shell description

**(Figs 21.6–21.10, 24.12–24.14).** The shell is elongated but somehow compressed, markedly fusiform to slightly pyriform, sinistral and not decollate. It is very dark reddish-brown in colour. The external surface is irregularly finely striated to almost completely smooth in some part of the teleoconch. The spire is slowly and regularly growing, with (decollate) 9 ^1^/_4_ to 10 ^3^/_4_ slightly convex whorls. The sutures are moderately deep with white papillae all along the teleoconch, definitely denser along the last whorls. The basal and the cervical keels are only barely distinguishable. The umbilicus is closed. The aperture is ~ ^1^⁄_5_ of shell height and roundish to subovoid in shape. The PRI is short and it ends at the level of the L. It is not fused with the L but presents a thickening along its posterior part in correspondence with the L. The PRI is not visible from a frontal view of the aperture. The L is dorsal to dorso-lateral. The PUPP is very short or knob-like and connected to the L. The AUPP is thin or rarely absent, detached from the L and not visible from the aperture. The BAS starts directly from the L and it is long and strong, well visible from the aperture. The SCL is absent or very short, connected with the L and resembling an irregular thickening. The IL is high to very high. The SUL is tooth-like, extremely long and remarkably overlapping with the SPL. The SPL is short and it ends at half of the last whorl. The SCOL is not visible. The peristome is continuous, markedly thickened and reflected. It is not superiorly fused to the wall of the first whorl. The palatal edge of the clausilium is distally receding and bent upwards. The plate is somehow cylindrical and gutter-like. The palatal edge against distal end bent upwards and more or less blunt.

**Figure 20.**
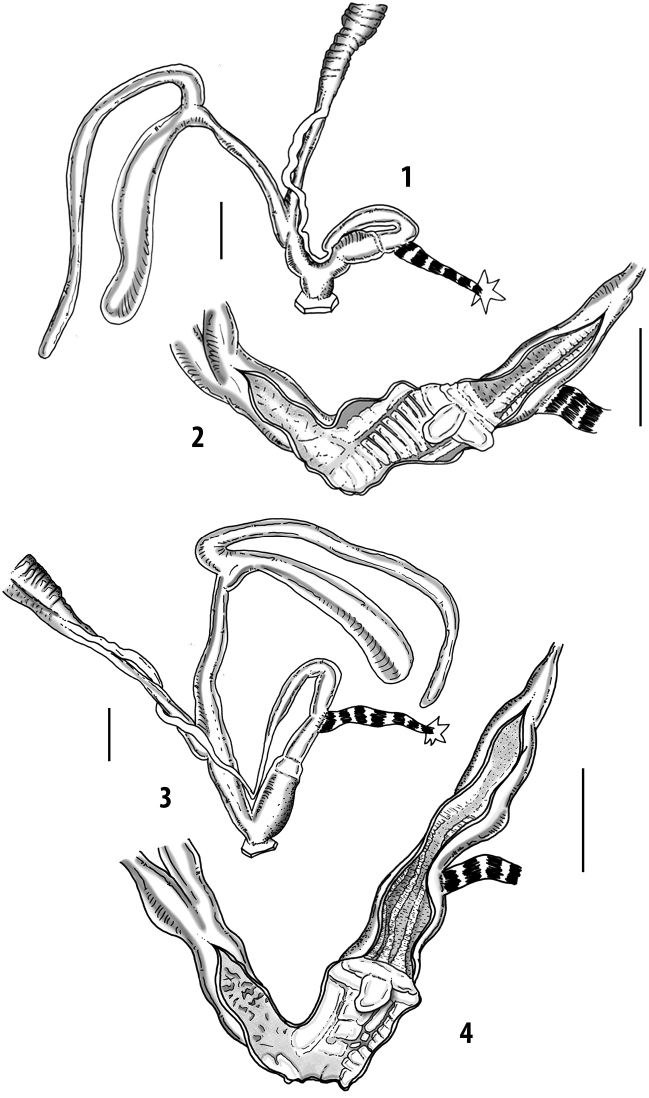
*Siciliariacalcaraeorlandoi* Liberto, Reitano, Giglio, Colomba & Sparacio, 2016, Bosco Ficuzza **20.1** whole distal genital organs **20.2** internal distal part of genital organs. *Siciliariacalcaraecruenta* ssp. nov., Monte Gibilmesi **20.3** whole distal genital organs **20.4** internal distal part of genital organs.

**Figure 21.**
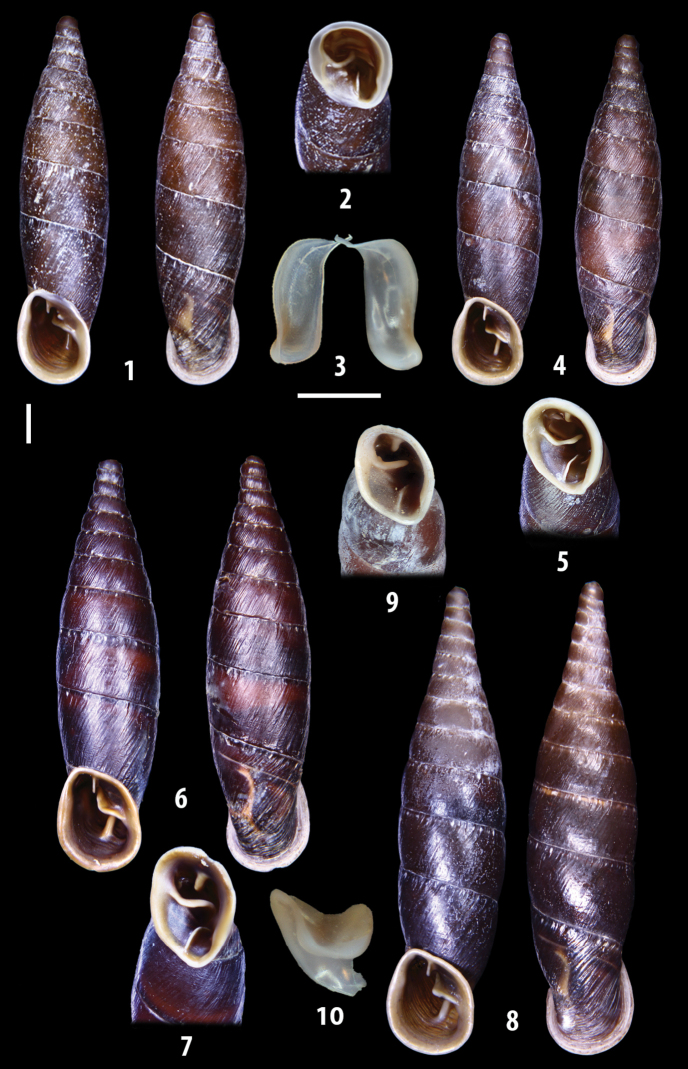
*Siciliariacalcaraeorlandoi* Liberto, Reitano, Giglio, Colomba & Sparacio, 2016, Bosco Ficuzza **21.1** shell **21.2** detail of the aperture **21.3** clausiliar plate double side **21.4** shell **21.5** detail of the aperture. *Siciliariacalcaraecruenta* ssp. nov., Monte Gibilmesi **21.6** shell **21.7** detail of the aperture **21.8** shell **21.9** detail of the aperture **21.10** view of the clausiliar plate.

###### Measurements.

***Holotype***: not decollate shell height 19.2, whorl width 4.4, aperture height 4.3, aperture width 3.2. ***Paratypes*** (n = 11, not decollate): shell height 19.4 ± 1.0, whorl width 4.5 ± 0.3, aperture height 4.3 ± 0.3, aperture width 3.1 ± 0.2.

###### External morphology of the genital organs

**(Fig. 20.3).** The FO is longer than the V (FO/V range 1.8–2.1). The VD is thin along its whole course. The FDBC is slightly shorter than the BC+SDBC (FDBC/BC+SDBC range 0.8–0.9). The BC+SDBC is club-like to cylindrical and longer than the V (BC+SDBC/V range 2.1–2.3), with no clear distinction between the SDBC and the BC. The apex is big and rounded. The D is longer than the V (D/V range 3.5–3.8) and longer that the BC+SDBC (D/BC+SDBC range 1.3–1.4), slightly thinner than the BC+SDBC and with a small and round apex. The V is cylindrical. The A is large. The PC is longer than the V (P+E/V range 2.9–3.1). The PR is long and robust. The E is longer than the P (E/P range 2.1–2.3) and gradually shrinking and turning into the VD.

###### Internal morphology of the genital organs

**(Fig. 20.4).** The A is smooth or with few weak fleshy folds. The P presents a large longitudinal fleshy scarcely segmented pleat. Accessory pleats are also present, heavily segmented or smooth, running from the proximal part of the P as far as the A. The fine structure of the penial wall is smooth. The penial pseudopapilla is big, rhombus-shaped and smooth. The P-E transition presents a first distal ER, the PP originates from the second proximal ER. The ELP are not connected to the second proximal ER. The epiphallar formula is: 1ER+2ER(PP)+ELP. The E shows a pattern of 3 to 4 irregular longitudinal extremely fringed pleats. These pleats merge one into another forming two main fringed pleats that run as far as the VD. The V shows a weak irregular pattern of smooth pleats.

###### Comparative and taxonomical remarks.

The morphologically most similar taxon to *Siciliariacalcaraecruenta* ssp. nov. is *Siciliariacalcaraeborgettensis* ssp. nov., but it differs from the latter by its very dark and remarkably smoother shell and the AUPP is not sharp but always suffused and almost not visible from the aperture. As regards the genital organs, contrary to *Siciliariacalcaraeborgettensis* ssp. nov., *Siciliariacalcaraecruenta* ssp. nov. presents a very long SDBC+BC and FO. The sculpturing of the internal penis differs by the less fringed longitudinal pleats and the EPLs that reach proximally back as far as the beginning of the VD. *Siciliariacalcaraecruenta* ssp. nov. is similar to *Siciliariacalcaraeorlandoi*, anyhow the latter presents a more finely striated shell, its AUPP is stronger and usually closer to the lunella. It was found in haplogroup 1, while *Siciliariacalcaraecruenta* ssp. nov. is in the haplogroup 2.

###### Distribution.

*Siciliariacalcaraecruenta* ssp. nov. in known only from the type locality: northern slopes of Monte Gibilmesi near Sagana (Montelepre). Further field investigation is needed in order to determine the actual distribution of the taxon.

###### Ecology.

This subspecies was found climbing on limestone cliffs hiding among moss and rocks crevices.

###### Etymology.

*Siciliariacalcaraecruenta* ssp. nov. was named after its intense dark red colour of the shell (*cruentus = bloody*).

##### ﻿ Siciliaria ferrox

Taxon classificationAnimaliaStylommatophoraClausiliidae

Brandt, 1961

852EA40D-D7A2-5CFD-8D8D-71C89DAD2AA7

Figs 1.F, 22.1–22.6, 23.1–23.4, 24.15–24.16

(?) Clausiliaconfinatacommeata – Westerlund 1892: 47.
Siciliaria ferrox Brandt 1961: 6.
Siciliaria (Siciliaria) ferrox – Manganelli et al. 1995: 25.
Siciliaria ferrox – Reitano et al. 2007: 328.
Siciliaria ferrox – Welter-Schultes 2012: 339.
Siciliaria ferrox – Liberto et al. 2015: 2015: 489.
Charpentieria ferrox – De Mattia 2017e.

###### Remarks.

In the COI tree (Fig. 4) *Siciliariaferrox* forms a separate subclade, which is separated by ~17% p distance from the other *Siciliaria* species. This species is restricted to the easternmost area of *Siciliaria* distribution, in the environs of Trabia, Altavilla Milicia and Termini Imerese. It is allotopic, isolated from all other *Siciliaria* species, except for the area of Montagna Grande, where also *S.calcarae* was reported (Nordsieck 2013b: 8). The species presents a distinctive shell as stated by Brandt (1961: 6): “(...) die sich durch den doppelten Nackenkiel und die zwei falschen oberen Gaumenfalten unterscheidet.”. Following our genital anatomical investigations, the internal structure of the genital organs revealed to be unique among the *Siciliaria* species, namely an extremely modified ER that becomes finely longitudinally fringed (Fig. 22.2). Moreover, the penial papilla is uncommonly long and irregular (Fig. 22.2–22.3) and the ELP are extremely fringed as well.

The status of *Clausiliaconfinatacommeata* Westerlund, 1892 (Westerlund 1892: 47) is not clear as its original description is poor, lacking the description of essential shell characters (AUPP) and unavailability of the type material (Reitano et al. 2007: 328).

###### Specimens examined.

Italy, Sicily, Trabia, Contrada Sant’Onofrio, 170 m asl, 37°59'17.62"N, 13°36'51.11"E, [Lab ID Sf_1, COI: MW758922, ITS2: MW757115; Lab ID Sf_2, COI: MW758917], W. De Mattia and J. Macor leg., 21.xii.2007. 3 dissected spm.

###### Shell

**(Figs 23.1–23.4, 24.15, 24.16).** Shell mostly not decollate; whorls ribbed, sutural papillae recognisable; dorsal keel distinct or indistinct; inferior lamella moderately high or high; two anterior upper palatal plicae present, upper one mostly separated from upper palatal plica; palatal edge of clausilium plate distally less or not receding, palatal edge distally not bent upwards (as in Nordsieck 2013b).

###### Measurements

**(n = 32, decollate).** shell height 17.7 ± 0.7, whorl width 3.8 ± 0.1, aperture height 3.6 ± 0.2, aperture width 2.4 ± 0.2.

###### External morphology of the genital organs

**(Fig. 21.1).** The FO is longer than the V (FO/V range 1.4–1.9). The VD is thin along its whole course except for its proximal section where it is wider. The FDBC is longer than the BC+SDBC (FDBC/BC+SDBC range 1.6–2.1). The BC+SDBC is club-like and slightly shorter than the V (BC+SDBC/V range 0.8–0.9), with no clear distinction between the SDBC and the BC. The apex is wide and round. The D is longer than the V (D/V range 1.8–2.0) and longer that the BC+SDBC (D/BC+SDBC range 1.9–2.2), thinner than the BC+SDBC and with a pointed apex. The V is short, wide and cylindrical. The A is small. The PC is longer than the V (P+E/V range 2.0.7–2.2). The PR is long and thin. The E is slightly thinner and shorter than the P (E/P range 0.8–0.9), almost abruptly shrinking and turning into the VD.

###### Internal morphology of the genital organs

**(Figs 22.3–22.6).** The A shows small irregular pleats. These pleats are the direct continuation of the irregular sculpture of both the distal P and V. Proximally, the P presents three or four fleshy pads. These pleats turn into and irregular-wrinkly pattern towards the distal part. The PP is always big, irregular and wrinkly. It is tapered towards the apex that is elongated and irregular. In cross section a clear folding is visible. It originates almost directly from the epiphallar transition wall. The transition wall shows an extremely irregular surface with a set of longitudinal papillated “pleats”. The transition area presents a particular structure, namely an extremely longitudinally fringed ring. It is not clear if the characteristic epiphallar ring becomes this fringed ring or it is missing and replaced by this unique structure. The epiphallus shows two main longitudinal extremely fringed pleats. The V shows a smooth irregular pattern of fine longitudinal pleats.

**Figure 22:**
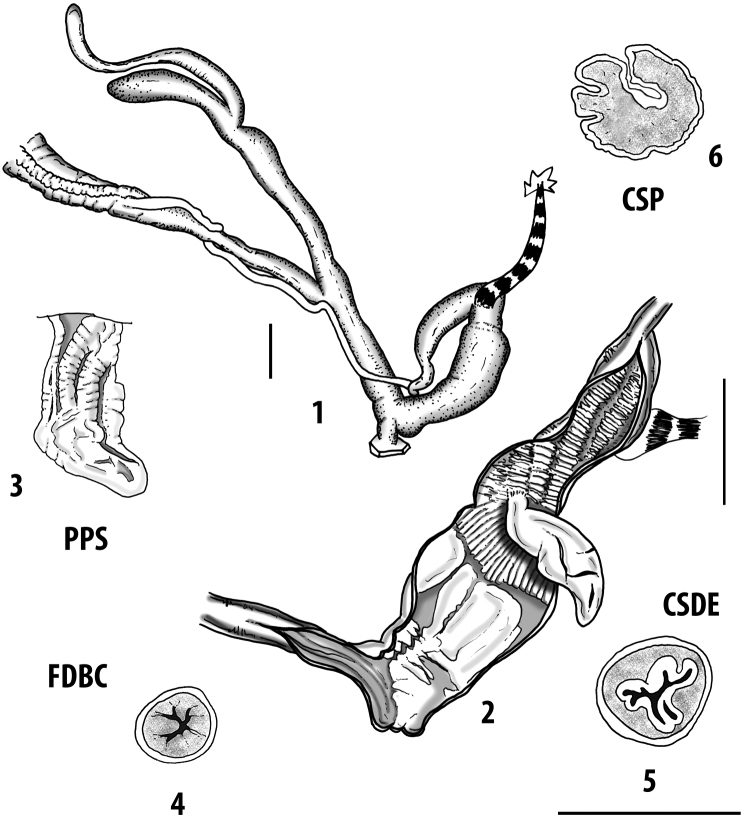
*Siciliariaferrox* (Brandt, 1961). Trabia Sant’Onofrio **22.1** whole distal genital organs **22.2** internal distal part of genital organs **22.3** detail of the pseusopapilla **22.4** cross section of the FDBC**22.5** cross section of the distal epiphallus **22.6** cross section of the pseudopapilla.

###### Ecology.

*Siciliariaferrox* inhabits limestone walls, hiding in rocks cracks and crevices. According to De Mattia (2017e)*Siciliariaferrox* is considered as Critically Endangered following the IUCN criteria B1ab(iii)+2ab(iii).

###### Distribution.

*Siciliariaferrox* occupies the easternmost part of the distribution range of the northwestern Sicilian *Siciliaria*. It is found from Altavilla Milicia, Sant’Onofrio to Termini Imerese.

##### ﻿ Siciliaria tiberii

Taxon classificationAnimaliaStylommatophoraClausiliidae

(A. Schmidt, 1868), s. l.

BB833D97-4732-5B84-B816-E946FC829B89

###### Remarks.

*Siciliariatiberii* s. l. forms an independent subclade in the COI tree and is separated from the other *Siciliaria* species by 14.3–17.8% (Fig. 4, Table 3).

**Figure 23.**
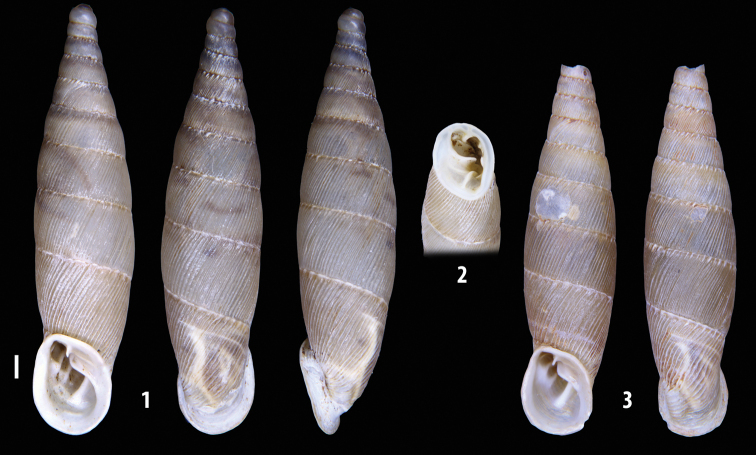
*Siciliariaferrox* (Brandt, 1961). Trabia Sant’Onofrio **23.1** shell **23.2** detail of the aperture **23.3** shell **23.4** detail of the aperture.

##### ﻿ Siciliaria tiberii tiberii

Taxon classificationAnimaliaStylommatophoraClausiliidae

(A. Schmidt, 1868)

1DD47A1A-356B-5385-A4D3-DEAA8AA05FA4

Figs 1.F, 25.1–25.2, 26.1–26.5, 29.1–29.3

Clausilia tiberii Schmidt 1868: 40.
Clausilia tiberiana Benoit 1876: 152.
Siciliaria tiberii – Boettger 1877: 33.
Siciliaria (Siciliaria) tiberii – Nordsieck 1979: 259.
Charpentieria tiberii – Beckmann 2004: 188.
Charpentieria (Siciliaria) tiberii tiberii – Nordsieck 2007: 54.
Siciliaria tiberii – Welter-Schultes 2012: 343.
Siciliaria tiberii – Nordsieck 2013b: 8.
Charpentieria tiberii – De Mattia 2017f.

###### Specimens examined.

Italy, Sicily, Terrasini, Capo Rama, 30 m asl, 38°08'19.06"N, 13°03'14.01"E, [Lab ID 19_1, COI: MW758919, ITS2: MW757116, MW757117; Lab ID 19_2, COI: MW758920], W. De Mattia and J. Macor leg., 16.xii.2007. 3 dissected spm.

###### Shell

**(Figs 26.1–26.5, 29.1–29.3).** Shell decollate; whorls ribbed; dorsal keel prominent; inferior lamella high to very high; anterior upper palatal plica strong and long, separated from the lunella; sometimes the anterior upper palatal plica presents a distal knob, sometimes another lower weak upper palatal plica is present close to the ALPP; anterior lower upper palatal plica strong and long; palatal edge of clausilium plate distally not receding, palatal edge distally more or less strongly bent upwards.

###### Measurements

(n = 40, decollate): shell height 16.3 ± 1.2, whorl width 4.0 ± 0.1, aperture height 3.1 ± 0.2, aperture width 2.3 ± 0.2.

###### External morphology of the genital organs

**(Fig. 25.1).** The FO is longer than the V (FO/V range 2.0–2.3). The VD is thin along its whole course except for the proximal part where it is larger in diameter. The FDBC is longer than the BC+SDBC (FDBC/BC+SDBC range 1.5–1.7). The BC+SDBC is club-like to cylindrical and slightly longer than the V (BC+SDBC/V range 1.2–1.5), with no clear distinction between the SDBC and the BC. The apex is big and rounded. The D is slightly longer than the V (D/V range 1.7–1.9) and slightly longer that the BC+SDBC (D/BC+SDBC range 1.1–1.5), thinner than the BC+SDBC and with a small and round apex. The V is big and cylindrical. The A is large and long. The PC is longer than the V (P+E/V range 2.9–3.4). The PR is very short and robust. The ET is clearly visible. The E is longer than the P (E/P range 1.1–1.7) and gradually shrinking and turning into the VD.

###### Internal morphology of the genital organs

**(Fig. 25.2).** The A shows few fleshy weak irregular folds. The P presents a set of transverse smooth pleats. The pleats are slightly irregular and tend to fade towards the A. The penial wall is smooth. The PP is big, elongated and markedly wrinkled with a pointed apex. It originates from one transverse epiphallar ring. The E shows two main fringed pleats that run as far as the VD. The V shows a weak irregular pattern of smooth pleats.

**Figure 24.**
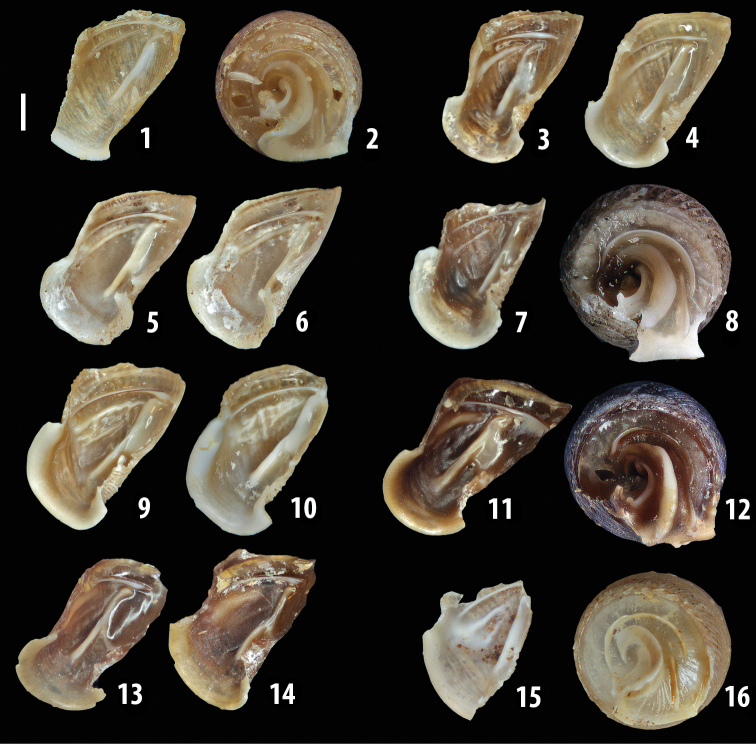
*Siciliariacalcaraecalcarae* (Philippi, 1844), Visicari, Castellammare del Golfo **24.1** palatal plicae **24.2** parietal lamellae. *Siciliariacalcaraebelliemii* (Brandt, 1961), Monte Belliemi, Partinico **24.3** palatal plicae. *Siciliariacalcaraebelliemii* (Brandt, 1961), Castello di Calatubo, Alcamo **24.4** palatal plicae. *Siciliariacalcaraeborgettensis* ssp. nov., road to Romitello, Borghetto **24.5–24.6** palatal plicae. *Siciliariacalcaraejatinensis* ssp. nov. San Giuseppe Jato **24.7** palatal plicae **24.8** parietal lamellae. *Siciliariacalcaraeparajatinensis* ssp. nov., west side Monte Kumeta **24.9–24.10** palatal plicae. *Siciliariacalcaraeorlandoi* Liberto, Reitano, Giglio, Colomba & Sparacio, 2016, Bosco Ficuzza **24.11** palatal plicae **24.12** parietal lamellae. *Siciliariacalcaraecruenta* ssp. nov., Monte Gibilmesi **24.13–24.14** palatal plicae. *Siciliariaferrox* (Brandt, 1961). Trabia Sant’Onofrio **24.15** palatal plicae **24.16** parietal lamellae.

###### Ecology.

*Siciliariatiberiitiberii* is found under stones, among scree, rocky debris and on dry stonewalls along the coastline, on a limestone plateau. According to De Mattia (2017f), the taxon is Near Threatened.

###### Distribution.

Beckmann (2004: 188), following Nordsieck’s personal communications cites *Siciliariatiberiitiberii* from a southern area surrounding San Cipirello (Madonna dei Furi, Monte Palmeto, Monte della Fiera, San Giuseppe Jato and Monte della Scala). As previously stated, this identification revealed to be wrong.

As a result, the actual distributional range of *Siciliariatiberiitiberii* seems to be limited to the area from San Cataldo (Partinico) in the south to Monte Palmeto and Capo Rama (Terrasini) in the north to the northern slopes of Monte Pecoraro. Further field research is needed in order to define its actual distribution. Recent findings of Diego Viola (Trieste, Italy) from a locality between San Giuseppe Jato and Piana degli Albanesi revealed the presence of a population of *Siciliaria* cfr. *tiberii* that clearly differ, as a shell, from *S.calcaraejatinensis* ssp. nov. and *S.calcaraeparajatinensis* ssp. nov.(personal communication). The poor samples available (few damaged shells) did not allow us a thorough investigation.

###### Remarks.

Monte San Calogero in the east (cited by Nordsieck as also included in the *S.tiberii* distribution), is an isolate limestone massif 10 km SE of Termini Imerese, 50 km east of San Cipirello and 65 km SSE of Capo Rama. The presence of *S.tiberiitiberii* in this locality, due to its remarkable distance, should be confirmed by further field research and not only inferred from museum samples. Mislabelling or wrong locality assignments could have played a role in this regard (as for *Siciliariaseptemplicataalcamoensis*, see below). The diagnosis provided by Nordsieck (2013b: 8) “Shell not or only in part decollate; dorsal keel less strong” imply that it might represent another population of *S.calcarae* s. l., yet more field and taxonomical research is needed. *Clausiliatiberiana*Paulucci 1878 is deemed as junior synonym of *Siciliariatiberiitiberii* by Nordsieck (2013b: 8). The authorship is probably wrong as Paulucci (1878: 13) cites *Clausiliatiberiana* Benoit: Benoit (1875: 152) introduced the name *Clausiliatiberiana* as a ”Elegante e bella specie de’ dintorni di S. Giuseppe delle Mortelle contrada non lungi da Palermo” and depicted this taxon in 1876 (Table VI, fig. 7). Benoit (1881: 107) stated that again that ”Questa bellissima e grande conchiglia, non conosciuta dagli autori siciliani vive nella contrada detta S. Giuseppe delle Mortelle, non lungi da Palermo”. Despite intensive collecting in the surrounding of Palermo by one of the authors (WDM), we were unable to identify the position of this old toponym (also by asking locals).

##### ﻿ Siciliaria tiberii alcamoensis

Taxon classificationAnimaliaStylommatophoraClausiliidae

Brandt, 1961 comb. nov.

3B4D1DDD-1672-500C-8969-6130F7AE9AEB

Figs 1.F, 25.3–25.5, 26.6–26.9, 29.4, 29.5

Siciliaria (Siciliaria) alcamoensis Brandt 1961: 9.
Siciliaria (Siciliaria) septemplicata alcamoensis – Manganelli et al. 1995: 25.
Siciliaria septemplicata hemmeni Beckmann 2004: 190.
Charpentieria (Siciliaria) septemplicata alcamoensis – Nordsieck 2007: 54.
Siciliaria septemplicata alcamoensis – Nordsieck 2013b: 10.

###### Examined specimens.

Italy, Sicily, Cinisi, Piano Margi, 670 m asl, 38°08'58.56"N, 13°09'25.83"E, [Lab ID 37_1, COI: MW758928, ITS2: MW757102, MW757101], W. De Mattia and J. Macor leg., 14.iv.2017. 2 dissected spm.

###### Shell

**(Figs 26.6–26.9, 29.4–29.5).** Shell decollate; whorls striated to rib-striated; dorsal keel very prominent; inferior lamella high; anterior upper palatal plica present, mostly separated from upper palatal plica, sometimes a second or also third upper palatal plica present; anterior lower palatal plica strong; palatal edge of clausilium plate distally not receding, palatal edge distally more or less strongly bent upwards.

###### Measurements

(n = 18, decollate): shell height 21.5 ± 1.3, whorl width 5.2 ± 0.3, aperture height 4.6 ± 0.2, aperture width 3.4 ± 0.4.

###### External morphology of the genital organs

**(Fig. 25.3).** The FO is longer than the V (FO/V range 1.4–1.6). The VD is thin along its whole course. The FDBC is as long as the BC+SDBC (DBC/BC+SDBC = 1.0). The BC+SDBC is spindle-like to cylindrical and longer than the V (BC+SDBC/V range 1.4–1.5), with no distinction between the SDBC and the BC. The apex is pointed. The D is longer than the V (D/V range 2.5–2.6) and longer that the BC+SDBC (D/BC+SDBC range 1.6–1.8), thinner than the BC+SDBC and with a pointed apex. The V is long, cylindrical and small in diameter. The A is large. The PC is much longer than the V (P+E/V range 2.4–2.6). The PR is long and thin. The ET is clearly visible. There is a clear distinction between P and E, there is a visible ER, and a proximal narrowing. The E is longer than the P (E/P range 2.0–2.1), gradually shrinking and turning into the VD.

###### Internal morphology of the genital organs

**(Figs 25.4–25.5).** The A shows a set of irregular fleshy folds. The P presents two or three main large longitudinal pleats. These pleats are heavily transversely segmented, getting a comb-like shape. The fine structure of the wall is smooth.

The PP is large, elongated, and heavily wrinkled. The P-E transition presents one ER with both the PP and ELP originating from the ER. The epiphallar formula is: 1ER(PP+ELP). The E shows a set of three or four smooth longitudinal pleats. The V is almost completely smooth. The wall shows a fine granulation.

###### Ecology.

*Siciliariatiberiialcamoensis* comb. nov. inhabits scattered isolated limestone boulders, and is hiding in rock cracks and crevices. After careful field research in a wide area around Piano Margi, the taxon revealed to be remarkably rare and scattered.

###### Distribution.

*Siciliariatiberiialcamoensis* comb. nov. is known only from a small area of less than 1 km^2^ in the surrounding of the type locality: Piano Margi south of Cinisi on the Montagna Longa. Further field research is needed to define its actual distribution range.

###### Remarks.

Brandt (1961: 9) described Siciliaria (Siciliaria) alcamoensis based on material from the Senckenberg Museum (SMF163949), provided by Monterosato and labelled as *Clausiliadifficilis* from “Alcamo in NW-Sizilien, ohne nähere Fundortangabe”. Brandt (1961: 9) introduced the name *alcamoensis* as *Clausiliadifficilis* sensu Monterosato [non *Clausiliadifficilis* Retowski, 1889, currently *Rosenielladifficilis* (Retowski, 1889)] is a nomen nudum. Brandt (1961: 10) stated that he was not able to find this species again during field collecting nearby Alcamo, but he also lists in the type material: ”Paratypoide Coll. Brandt Cl. 2365/2 leg. Belluci”. He did not provide the collecting site of the paratype material so its origin is unknown, but it is conceivable that their shell morphology matches with what he described as Siciliaria (Siciliaria) alcamoensis indeed. Undoubtedly, the locality indication provided by Monterosato on the original SMF label is wrong. Despite intensive field research (WDM) all over the Alcamo area and Bosco d’Alcamo/Monte Bonifato, no specimens/population was found that matches Brandt’s description of *Siciliariaalcamoensis*. Beckmann (2004: 190) described *Siciliariaseptemplicatahemmeni* from Piano Margi (Cinisi, Palermo). Its description and the pictures (Beckmann 2004: 189 and 190) match with Brandt’s Siciliaria (Siciliaria) alcamoensis, as stated by Nordsieck (2013b: 7) and therefore, it is reasonable to assume that the Monterosato material (SMF163949) was collected from the same area of Beckmann’s species.

*Siciliariatiberiialcamoensis* comb. nov. (Figs 26.6–26.9) was treated as a subspecies of *S.septemplicata* (MolluscaBase 2021). This is due to the fact that the typical form of *S.septemplicata* (e.g., Monte Gallo populations, Figs 10.1–10.7), is quite similar regarding the morphology of the clausiliar apparatus, (Nordsieck 2013b), despite they greatly differ in the shell surface, sutures ornamentation, colouration, thickness and dimensions. The genital morphology (Figs 25.3–25.4) of *S.tiberiialcamoensis* gretaly differs from that of *S.s.septemplicata* (Figs 9.1–9.2). In the COI and concatenated trees, *S.t.alcamoensis*is is well embedded within the *Siciliariatiberii* clade. Since it is found well within the distribution range of *S.tiberii* s. l., introgression due to hybridisation appears as an unlikely explanation. It might be better explained by incomplete lineage sorting. Possible interspecies hybridisation should be tested on a larger sample size with additional nuclear markers. There is no argument for considering *S.s.alcamoensis* as a subspecies of *S.septemplicata*, we here consider it a subspecies *S.tiberii*.

**Figure 25.**
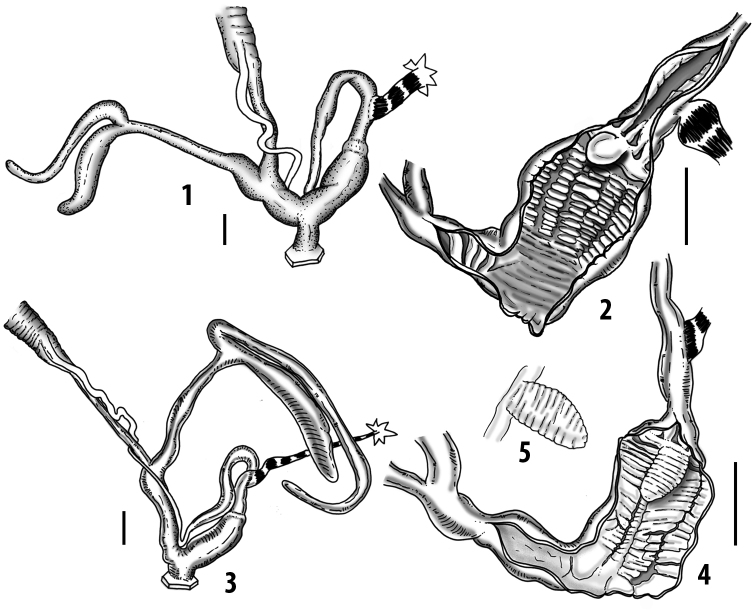
*Siciliariatiberiitiberii* (A. Schmidt, 1868), Capo Rama, Terrasini **25.1** whole distal genital organs **25.2** internal distal part of genital organs. *Siciliariatiberiialcamoensis* Brandt, 1961 comb. nov., Piano Margi, Carini **25.3** whole distal genital organs **25.4** internal distal part of genital organs **25.5** detail of the pseudopapilla.

**Figure 26.**
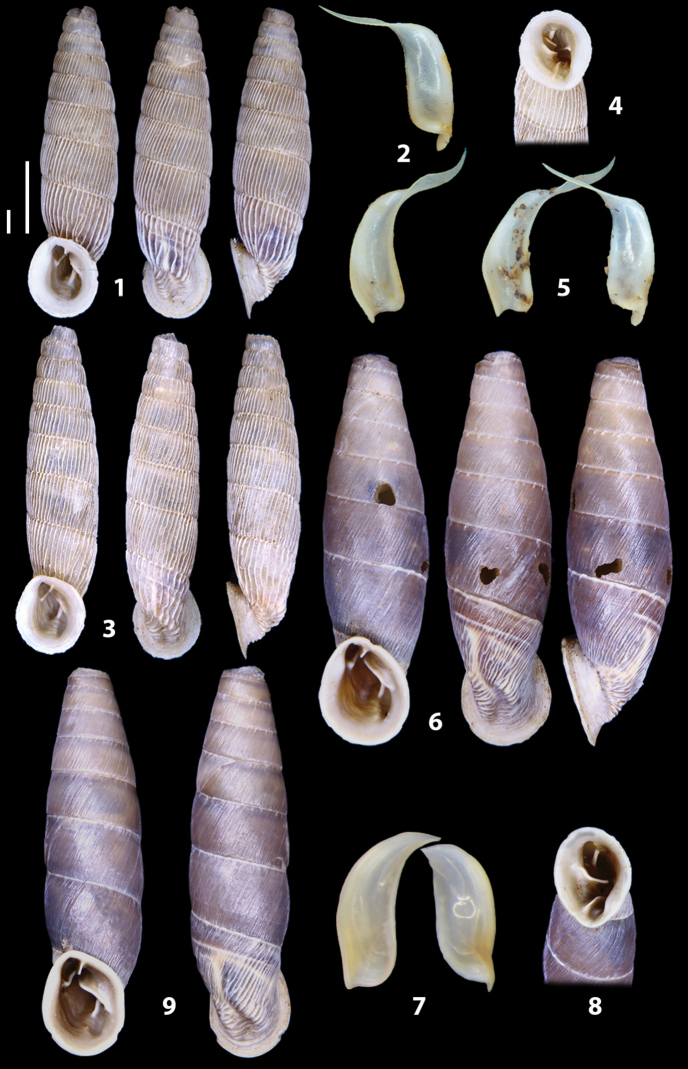
*Siciliariatiberiitiberii* (A. Schmidt, 1868), Capo Rama, Terrasini **26.1** shell **26.2** clausiliar plate double side **26.3** shell **26.4** detail of the aperture **26.5** clausiliar plate double side. *Siciliariatiberiialcamoensis* Brandt, 1961 comb. nov., Piano Margi, Carini **26.6** shell **26.7** clausiliar plate double side **26.8** detail of the aperture **26.9** shell.

##### ﻿ Siciliaria tiberii armettensis ssp. nov.

Taxon classificationAnimaliaStylommatophoraClausiliidae

E3DDE510-75F5-5ADA-A0EA-09297D66A35E

http://zoobank.org/BF64153C-F65F-4EA2-8794-269213F5A345

Figs 1.F, 27.1–27.3, 28.1–28.3, 29.6–29.8

###### Type locality.

Italy, Sicily, Carini, Grotta dei Puntali o dell’Armetta, 90 m asl, 38°8'58.56"N, 13°9'25.83"E.

###### Type material.

1 ***Holotype*** (NHMW 113621) [Lab ID 38_1, COI: MW758880, ITS2: MW757098, MW757099, MW757100] and 4 ***Paratypes*** (NHMW 113622): Italy, Sicily, Carini, Grotta dei Puntali o dell’Armetta, 90 m asl, 38°08'58.56"N, 13°09'25.83"E, [Lab ID 38_2, COI: MW758881], W. De Mattia and J. Macor leg., 16.iv.2017. 3 dissected spm. 8 ***Paratypes*** (CMWDM 18227): same locality.

###### Shell diagnosis.

Shell decollate; whorls moderately ribbed; dorsal keel prominent; inferior lamella high; anterior upper palatal plica present, knob present at distal end of the upper palatal plica, detached from the lunella; lower anterior palatal plica (ALPP) present; palatal edge of clausilium plate distally not receding, palatal edge distally strongly bent upwards.

###### Shell description

**(Figs 28.1–28.3, 29.6–29.8).** The shell is elongated, fusiform, sinistral, decollate and robust. It is brown to dark reddish in colour. The shell surface has small ribs, more densely packed towards the decollate tip and gradually spreading towards the aperture. There are whitish papillae along the moderately deep sutures throughout the whole teleoconch. The spire is slowly and regularly growing, with 7 ½ (after decollation) slightly convex whorls. The basal and cervical keels are well distinct. The umbilicus is closed. The aperture is roundish to subovoid and its height is ~ ^1^/_4_ to ^1^/^5^ of shell height. The PRI is short and it ends at the level of the L. It is not fused with the L and presents a slight thickening along its posterior end. The PRI is not visible from a frontal view of the aperture. The L is dorso-lateral to lateral. The PUPP is short or obsolete, knob-like and connected to the L. The AUPP is strong and long, detached from the L and clearly visible from the aperture. The distal end of the AUPP has a well-developed knob. The BAS starts directly from the L and it is long and strong, well visible from the aperture. The SCL is absent or resembling an irregular small thickening, connected with the lunella. The IL is very high to extremely high. The SUL is tooth-like, long and overlapping with the SPL. The SPL is long and it ends at ^4^/_5_ of the last whorl. The SCOL is not visible. The peristome is continuous, markedly thickened and reflected. It is not superiorly fused to the wall of the first whorl. The palatal edge of the clausilium is distally receding and bent upwards. The plate is cylindrical in shape. The palatal edge against the distal end is bent upwards and more or less blunt.

###### Measurements.

***Holotype***: decollate shell height 20.1, whorl width 4.8, aperture height 4.6, aperture width 3.1. ***Paratypes*** (n = 35, decollate): shell height 19.7 ± 1.6, whorl width 4.6 ± 0.2, aperture height 4.2 ± 0.3, aperture width 2.8 ± 0.3.

###### External morphology of the genital organs

**(Fig. 27.1).** The FO is longer than the V (FO/V range 1.5–1.8). The VD is thin along its whole course. The FDBC is slightly longer than the BC+SDBC (FDBC/BC+SDBC range 1.0–1.3). The BC+SDBC is irregularly spindle-like and longer than the V (BC+SDBC/V range 1.6–1.9), with no distinction between the SDBC and the BC. The apex is pointed. The D is longer than the V (D/V range 3.0–3.2) and longer that the BC+SDBC (D/BC+SDBC range 2.1–2.3), much thinner than the BC+SDBC and with a small but round apex. The V is short, cylindrical and large in diameter. The A is large. The PC is much longer than the V (P+E/V range 2.6–2.8). The PR is short and robust. The ET is not clearly visible. The E is longer than the P (E/P range 1.7–1.9), gradually shrinking and turning into the VD.

###### Internal morphology of the genital organs

**(Figs 27.2, 27.3).** The A shows a set of irregular fleshy folds. The P presents a main longitudinal fleshy fold that goes from the PP as far as the A. This fold is heavily segmented in an oblique way. Additionally, the P also shows a set of small longitudinal and irregular small pleats that reach the A. The fine structure of the wall is smooth. The PP is small, elongated and smooth. The P-E transition presents a first distal and a second median ER while both the PP and ELP originates from a third proximal ER. The epiphallar formula is: 1ER+2ER+3ER(PP+ELP). The E shows five to seven smooth longitudinal pleats that proximally turn into a pattern of coarse small weak pleats that gradually fade away towards the VD. The V shows a set of heavy and robust transverse smooth pleats that proximally begin somehow irregularly. The wall is smooth.

###### Comparative analysis.

*Siciliariatiberiiarmettensis* ssp. nov., like the nominate subspecies, has a ribbed shell and it is clearly distinguishable from all the remaining subspecies that present a more or less striated but ribless shell surface. Nonetheless, the nominate subspecies shows a stronger ribbing, with elevated and robust ribs. The distal end of the AUPP always presents a knob that is well visible from the frontal view (Figs 28.1–28.2). This feature is shared also by the nominate subspecies (Fig. 26.1) but only by the ≈25% of the investigated specimens (n = 40).

###### Ecology.

*Siciliariatiberiiarmettensis* ssp. nov. inhabits east-exposed limestone walls and cliffs, hiding among tuft of vegetation, rocks cracks and crevices.

###### Distribution.

*Siciliariatiberiiarmettensis* ssp. nov. is exclusively known from the type locality, the surroundings of the Grotta dei Puntali o dell’Armetta. Further field investigation along the Monte Pecoraro eastern slopes is needed in order to determine the actual distribution of the taxon. Grotta dei Puntali o dell’Armetta are included in the protected area Riserva Naturale Grotta dei Puntali.

###### Etymology.

The taxon is named after the Grotte dell’Armetta, a complex of natural caves where wall engravings and stone handcrafted tools, dated from the Paleolithic to the Bronze Age, were discovered (Mannino 1978).

**Figure 27.**
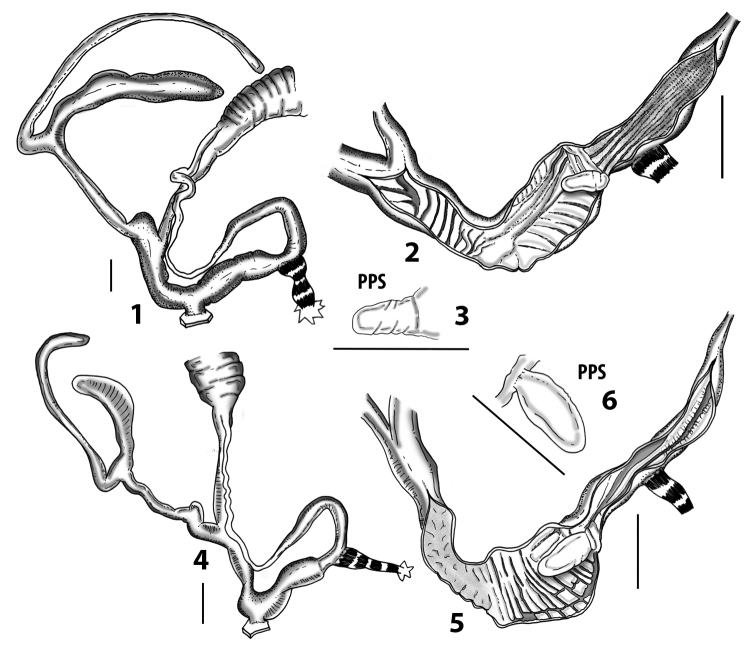
*Siciliariatiberiiarmettensis* ssp. nov., Grotta dei Puntali, Carini **27.1** whole distal genital organs **27.2** internal distal part of genital organs **27.3** detail of the pseudopapilla. *Siciliariatiberiiscalettensis* Beckmann, 2004 Portella Scaletta, Villagrazia **27.4** whole distal genital organs **27.5** internal distal part of genital organs **27.6** detail of the pseudopapilla.

##### ﻿ Siciliaria tiberii scalettensis

Taxon classificationAnimaliaStylommatophoraClausiliidae

Beckmann, 2004

E0761FBC-191B-5C97-AE54-3926465B2E5B

Figs 1.F, 27.4–27.6, 28.5–28.9, 29.9–29.11

Charpentieria (Siciliaria) tiberii scalettensis Beckmann 2004: 188.
Siciliaria tiberii scalettensis – Nordsieck 2007: 54.
Siciliaria tiberii scalettensis – Nordsieck 2013b: 10.

###### Specimens examined.

Italy, Sicily, Cinisi, Portella Scaletta, 90 m asl, 38°10'35.53"N, 13°07'27.22"E, [Lab ID 17_2, COI: MW758923, ITS2: MW757108, MW757109, MW757110], W. De Mattia and J. Macor leg., 18.iv.2017. 3 dissected spm. Italy, Sicily, Cinisi, along the SS113 road from Villagrazia di Carini to Cinisi, 70 m asl, 38°10'42.45"N, 13°07'34.50"E, [Lab ID 39_1, COI: MW758882, ITS2: MW757095, MW757096, MW757097], W. De Mattia and J. Macor leg., 18.iv.2017. 2 dissected spm.

###### Shell

**(Figs 28.5–28.9, 29.9–29.11).** Shell mostly decollate; whorls striated; dorsal keel mostly prominent; inferior lamella high to very high; anterior upper palatal plica present, separated or connected with upper palatal plica; a second lower separate upper palatal present, anterior lower palatal plica strong; palatal edge of clausilium plate distally not receding, palatal edge distally more or less strongly bent upwards.

**Figure 28.**
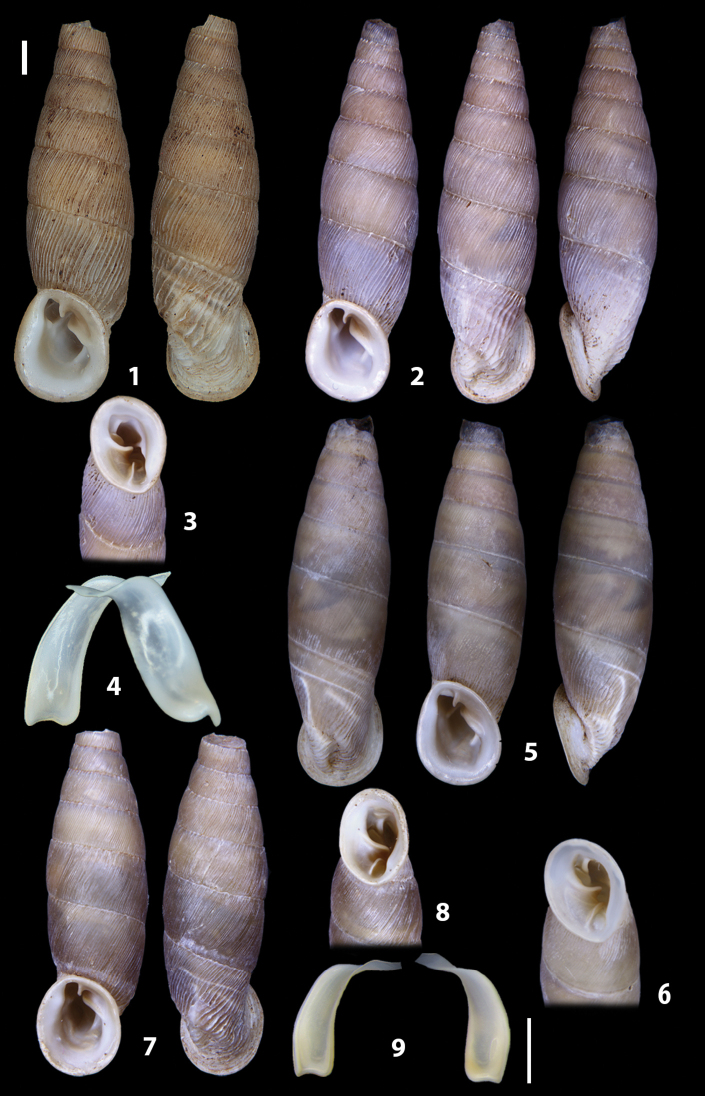
*Siciliariatiberiiarmettensis* ssp. nov., Grotta dei Puntali, Carini **28.1** shell **28.2** shell clausiliar plate double side **28.3** detail of the aperture **28.4** clausiliar plate double side. *Siciliariatiberiiscalettensis* Beckmann, 2004 Portella Scaletta, Villagrazia **28.5** shell **28.6** detail of the aperture **28.7** shell **28.8** detail of the aperture **28.9** clausiliar plate double side.

###### Measurements

**(n = 45, decollate).** shell height 18.5 ± 1.3, whorl width 4.6 ± 0.2, aperture height 4.0 ± 0.2, aperture width 2.9 ± 0.2.

###### External morphology of the genital organs

**(Fig. 27.4).** The FO is as long as the V (FO/V = 1.0). The VD is thin along its whole course. The FDBC is slightly longer than the BC+SDBC (FDBC/BC+SDBC range 1.2–1.3). The BC+SDBC is club-like to cylindrical and slightly shorter than the V (BC+SDBC/V range 0.8–0.9), with no clear distinction between the SDBC and the BC. The apex is big and rounded. The D is longer than the V (D/V range 1.3–1.7) and longer that the BC+SDBC (D/BC+SDBC range 1.6–1.8), thinner than the BC+SDBC and with a small and round apex. The V is cylindrical. The A is large. The PC is longer than the V (P+E/V range 1.5–1.9). The PR is short and robust. The ET is visible. The E is longer than the P (E/P range 2.0–2.2) and gradually shrinking and turning into the VD.

**Figure 29.**
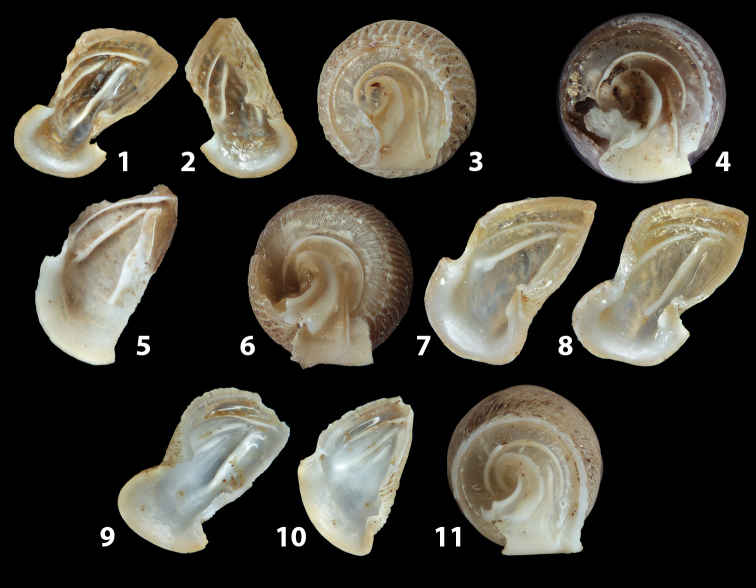
*Siciliariatiberiitiberii* (A. Schmidt, 1868), Capo Rama, Terrasini **29.1–29.2** palatal plicae **29.3** parietal lamellae. *Siciliariatiberiialcamoensis* Brandt, 1961 comb. nov., Piano Margi, Carini **29.4** parietal lamellae **29.5** palatal plicae. *Siciliariatiberiiarmettensis* ssp. nov., Grotta dei Puntali, Carini **29.6** parietal lamellae **29.7–29.8** palatal plicae. *Siciliariatiberiiscalettensis* Beckmann, 2004 Portella Scaletta, Villagrazia **29.9–29.10** palatal plicae **29.11** parietal lamellae.

###### Internal morphology of the genital organs

**(Figs 27.5, 27.6).** The A is smooth or with few fleshy weak, oblique folds. The P presents a set of transverse, often interrupted smooth pleats. The penial wall is smooth. The PP is big, elongated and smooth. The P-E transition presents one ER. The PP doesn’t originate from an ER and is connected to ELP. The epiphallar formula is: 1ER+PP(ELP).

The P of the population from the road to Cinisi (300 from NNE of the type locality), proximally shows pleats that are longitudinal and progressively turn into a transverse pattern as proceeding towards the A. The PP is also markedly different, being heavily wrinkled but the epiphallar formula is the same as the LT. The E shows a pattern of three or four smooth longitudinal pleats. These pleats merge one into another forming two main fringed pleats that run as far as the VD. The V is almost smooth with a very fine granulation.

###### Ecology.

*Siciliariatiberiiscalettensis* inhabits limestone walls and cliffs, hiding among tuft of vegetation, rocks cracks and crevices. The taxon is common at the type locality (Villa Paradiso) with a rich population and few more scattered, smaller populations among the limestone spur.

###### Distribution.

*Siciliariatiberiiscalettensis* is known only from a small area of less than 0.5 km^2^ around the of the type locality: Portella Scaletta along the SS113 road, from Villagrazia di Carini to Cinisi (Palermo). It was collected along a limestone spur heading NNE.

###### Remarks.

90% of the specimens (n = 45) of *Siciliariatiberiiscalettensis* present a second, lower AUPP, shorter than the superior AUPP and detached both from the AUPP and the L. This feature is shared only with the nominate subspecies.

####### ﻿*Sicania* taxonomic part

In Table 7 the main differences among the *Sicania* taxa regarding both shell and genital main taxonomical characters are summarised.

**Table 7.** Main morphological differences (shell and genital organs) among the *Sicania* taxa.

	***Sicaniacrassicostata* comb. nov.**	***Sicaniaeminens* comb. nov.**	***Sicanianobilisnobilis* comb. nov.**	***Sicanianobilisspezialensis* comb. nov., stat. nov.**
Whorls	ribbed, percostate	ribbed	weakly rib-striated	densely ribbed
Surface	opaque	opaque	white surface layer	white surface layer
Dorsal keel	weak	weak or missing	missing	missing
AUPP	missing	present, visible from outside	present, mostly not visible from outside	mostly missing
ALPP	very short or mostly missing	present, visible from outside	present, visible from outside	very short to knob-like, not visible from outside
Spiralis	not overlapping parietalis	overlapping parietalis	slightly overlapping parietalis	mostly not overlapping parietalis
Sculpturing of atrium	fleshy pleats	large fleshy pleats	irregular folds	irregular fleshy folds.
Sculpturing of vagina	smooth to an irregular	smooth to an irregular	smooth to an irregular	distally irregular pleats, proximally oblique pleats
Sculpturing of penis	longitudinal fleshy smooth pleats	longitudinal fleshy pleats	smooth, tubercles, longitudinal, segmented pleats	longitudinal fleshy smooth pleats
Pseudopapilla	short and conical	small and rounded	big and elongate to small and round	little, rhombus-shaped and smooth
Epiphallar ring	single	single	two or three	two
Sculpturing of epiphallus	two main longitudinal pleats	two main longitudinal pleats	two main longitudinal pleats	three or four irregular longitudinal fringed pleats

### Order Stylommatophora

**Infraordo Clausilioidei Gray, 1855**

**Superfamilia Clausilioidea Gray, 1855**

**Familia Clausiliidae Gray, 1855**

**Subfamilia Alopiinae A.J. Wagner, 1913**

**Tribus Delimini R.A. Brandt, 1956**

#### Genus *Sicania* Tomlin, 1929

##### ﻿ Sicania crassicostata

Taxon classificationAnimaliaStylommatophoraClausiliidae

(L. Pfeiffer, 1856) comb. nov.

DCDE1BB4-8549-5F61-AAB6-D64BC33F088B

Figs 1.F, 30.1–30.4, 31.1–31.7, 39.1–39.2

Clausilia crassicostata L. Pfeiffer 1856: 184.
Clausilia crassicostata – Schmidt 1868: 40.
Siciliaria crassicostata – Boettger 1877: 33.
Clausilia crassicostata – Westerlund 1884: 45.
Siciliaria (Siciliaria) crassicostata – Manganelli et al. 1995: 25.
Charpentieria crassicostata – Beckmann 2004: 188.
Charpentieria crassicostata – Nordsieck 2007: 54.
Siciliaria crassicostata – Welter-Schultes 2012: 339.
Charpentieria crassicostata – De Mattia 2017g.

###### Remarks

. The morphology of the genital organs of *Sicaniacrassicostata* comb. nov. revealed to be very stable in both of the investigated populations (Fig. 30.1–30.4). The internal sculpturing of P is made of large longitudinal pleats that it is a common genital trait, with very few exceptions, for all the taxa belonging to *Sicania.*

The shell is remarkably ribbed to percostated. It shares few characters with *Sicanianobilisspezialensis* comb. nov. stat. nov., e.g., the (mostly) missing AUPP, both the very short (or missing) ALPP and the spiralis (mostly) not overlapping the parietalis (Fig. 39.1–39.2).

Although *Sicaniaeminens* comb. nov. was initially introduced by Schmidt (1868: 40) as a variety of *Sicaniacrassicostata* comb. nov. due to shell similarities (and distribution proximity), the mt tree based on COI sequences reveals *Sicaniacrassicostata* comb. nov. as a distinct clade which is the sister group of *Sicanianobilis* s. l. (Figs 4–6). Considering the ITS2 tree and the combined tree (COI plus ITS2), the relationships among the three taxa are not well resolved. Notably, the distances between the three taxa are in the same range (~ 13%; see Table 2). The form of *Sicaniacrassicostata* comb. nov. from the surroundings of Tonnara di Monte Cofano is remarkably percostate, whereas the population we investigated from Mangiapane is ribbed (as stated also in Nordsieck 2013b: 9).

###### Specimens examined.

Italy, Sicily, Castelluzzo, cliffs W of the Tonnara di Monte Cofano, 50 m asl, 38°06'44.89"N, 12°40'34.27"E, [Lab ID 33_1, COI: MW758876; Lab ID 33_2, COI: MW758877, ITS2: MW757103, MW757104, MW757105MW757131], W. De Mattia and J. Macor leg., 21.xii.2007. 3 dissected spm. Italy, Sicily, Custonaci, cliffs N of the Mangiapane cave, 63 m asl, 38°05'40.91"N, 12°40'10.98"E, [Lab ID 1_1, COI: MW758912, ITS2: MW757114, MW757113; Lab ID 1_4, COI: MW758924] W. De Mattia and J. Macor leg., 21.xii.2007. 2 dissected spm.

###### Shell

(**Figs 31.1–31.8, 39.1–39.2).**: Shell decollate; whorls markedly ribbed, in part percostate; dorsal keel weak; inferior lamella moderately high or high; anterior upper palatal plica missing, ALPP very short or mostly missing; palatal edge of clausilium plate distally not receding, palatal edge distally somewhat or not bent upwards (Nordsieck 2013b).

###### Measurements

**(n = 24, decollate).** shell height 24.6 ± 1.2, whorl width 4.9 ± 0.1, aperture height 4.8 ± 0.3, aperture width 3.0 ± 0.1.

###### External morphology of the genital organs

**(Figs 30.1, 30.3).** The FO is much longer than the V (FO/V range 2.8–3.0). The VD is thin along its whole course except for its proximal section that becomes wider. The FDBC is longer than the BC+SDBC (FDBC/BC+SDBC range 1.7–1.9). The BC+SDBC is club-like and longer than the V (BC+SDBC/V = 2.0), with clear distinction between the SDBC and the BC. The apex is wide and rounded. The D is much longer than the V (D/V range 3.0–3.1) and longer that the BC+SDBC (D/BC+SDBC range 1.6–1.9), thinner than the BC+SDBC, slim and with a pointed apex. The V is wide and cylindrical. The A is large. The PC is much longer than the V (P+E/V range 3.2–3.4). The PR is short and robust. The ET is missing. The E is thinner and slightly longer than the P (E/P range 1.0–1.3), gradually shrinking and turning into the VD.

###### Internal morphology of the genital organs

**(Figs 30.2, 30.4).** The A shows few large fleshy pleats that are the direct continuation of the penial pleats. The P presents 3–6 longitudinal heavy fleshy smooth pleats that can sometimes become wrinkly and segmented. The fine structure of the wall is smooth. The V shows an irregular pattern of fine pleats. Distally, the V shows a large fleshy continuous cushion that abruptly turns proximally into a dense and irregular net-like pattern of small smooth pleats. The PP is big, short and conical with a pointed apex. The PP is big, elongated and smooth. The P-E transition presents two slightly different structures in the two dissected population. The population from Tonnara Cofano presents one ER. The PP does not originate from an ER and is connected to ELP. The epiphallar formula is: 1ER+PP(ELP). The Mangiapane population presents one ER with the PP originating from it. The ELP are not connected with the ER. The epiphallar formula is: 1ER(PP)+ELP. The E has two main longitudinal pleats, heavily segmented towards the VD, that proximally fade or can also show three smooth longitudinal pleats that almost abruptly turn into an irregular pattern of dense, small, transversely-oriented papillae.

###### Ecology.

*Sicaniacrassicostata* comb. nov. was collected on limestone cliffs or under rocks and boulders. According to De Mattia (2017g)*Sicaniacrassicostata* comb. nov. is considered Critically Endangered, following the IUCN criteria B1ab(i,ii,iv,v); C2a(ii) mainly due its very restricted geographic range and the severe population fragmentation.

###### Distribution.

This species has a very limited distribution range, restricted to the western part of Monte Cofano north of Custonaci (Tonnara di Cofano and Cornino). A (probably) introduced population is known to occur on the Island of Favignana (Trapani) (Nordsieck 2013b), but we did not have the opportunity to check this record in the field, in order to assess whether or not the population is still extant.

**Figure 30.**
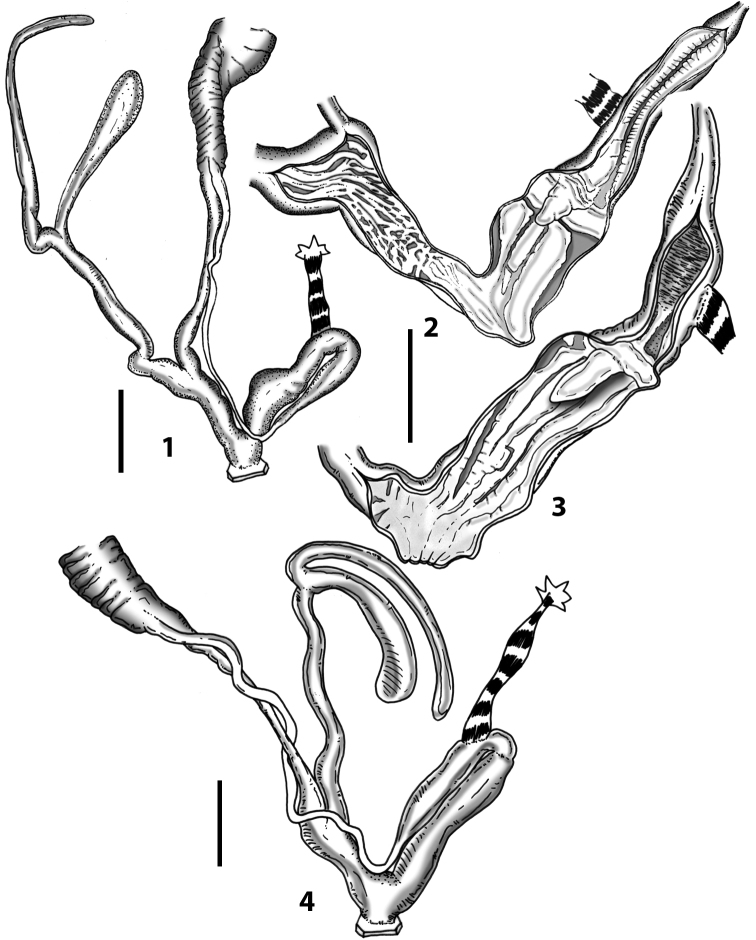
*Sicaniacrassicostata* (L. Pfeiffer, 1856), comb. nov., Monte Cofano **30.1** whole distal genital organs **30.2** internal distal part of genital organs **30.3** whole distal genital organs **30.4** internal distal part of genital organs.

**Figure 31.**
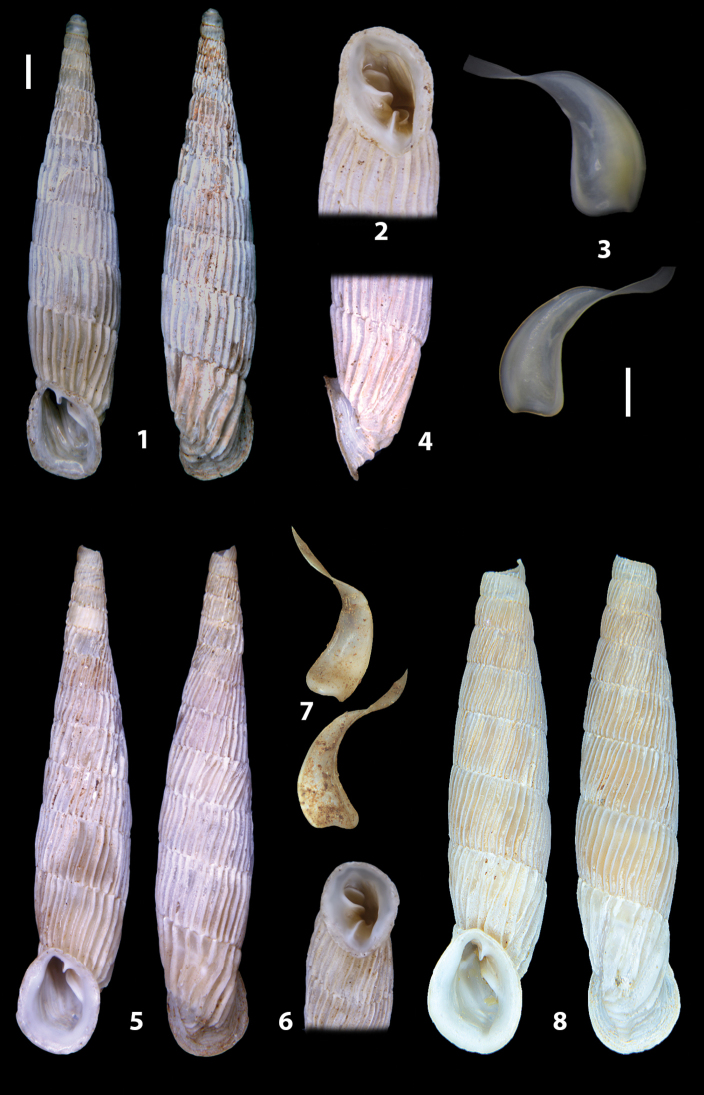
*Sicaniacrassicostata* (L. Pfeiffer, 1856), comb. nov., Monte Cofano **31.1** shell **31.2** detail of the aperture **31.3** clausiliar plate double side **31.4** detail of the columellar side of last whorl **31.5** shell **31.6** detail of the aperture **31.7** clausiliar plate double side. Custonaci **31.8** shell.

##### ﻿ Sicania eminens

Taxon classificationAnimaliaStylommatophoraClausiliidae

(A. Schmidt, 1868) comb. nov.

3C69BB36-2423-5471-A443-83052EE45099

Figs 1.F, 32.1–32.9, 33.1–33.12, 39.3, 39.4

Clausilia crassicostata var. eminens Schmidt 1868: 40.
Clausilia crassicostata var. eminens – Westerlund 1884: 45.
Siciliaria crassicostata eminens – Alzona 1971: 92.
Charpentieria eminens – Beckmann 2004: 188.
Charpentieria eminens – Nordsieck 2007: 53.
Siciliaria crassicostata var. eminens – Welter-Schultes 2012: 339.
Siciliaria eminens – Nordsieck 2013b: 9.
Charpentieria eminens – De Mattia 2017h.

###### Remarks.

*Sicaniaeminens* comb. nov. inhabits a restricted range that approximately includes the surroundings of Custonaci and the S slopes of Monte Cofano in the east as far as Castellammare del Golfo in the west.

The general genital outline is stable for all the dissected populations. The internal sculpturing of the penis reveals a basic pattern of one to four longitudinal pleats. The remaining main genital internal parts (penial pseudopapilla, epiphallus and atrium) show good stability despite slight variations, which will be described below. Only the internal wall of the vagina could be either smooth or with a strong sculpturing.

The shell is remarkably ribbed although the number of the ribs (density) progressively decreases along a geographical S-N axis, whereby simultaneously the thickness of the ribs increases. Southern populations, e.g., from Dolina Buffara and Baglio Messina, present a dense ribbing (with an average of 70 ribs along the first whorl) whereby the northernmost populations from the environs of Mangiapane Caves and Scurati show much less rib density (with an average of 32 ribs along the first whorl). The northern populations are found a few hundred meters from the southernmost population of *Sicaniacrassicostata* comb. nov. and could be, by inexperienced eyes, easily confused with it. Probably this is the reason why the taxon was for long time considered as a subspecies of *Sicaniacrassicostata* comb. nov. (Alzona, 1971: 92). Nevertheless, *Sicaniaeminens* comb. nov. can be distinguished by the markedly pyriform shape of its shell and the presence of a strong anterior upper palatal plica and the ALPP, both of them clearly visible from outside of the shell’s aperture and missing in *Sicaniacrassicostata* comb. nov. (Figs 31.1–31.7, 39.1, 39.2).

*Sicaniaeminens* comb. nov. is monophyletic in both the COI and in the ITS2 tree (both well supported). The ITS2 sequences were almost identical (Fig. 5). The distribution of the samples within the subclade, in spite of their low number, approximately reflects the S-N gradient in the rib density of the shells. The upper part includes the southern high-rib-density populations of Dolina Buffara and Baglio Messina, whereas the lower part includes the low-rib-density populations of Custonaci and Contrada Scurati. The results of the molecular genetic analysis, the morphology of the genital organs, conchological and biogeographic investigations confirm the status of *Sicaniaeminens* comb. nov. as a valid species, clearly distinguished from *Sicaniacrassicostata* comb. nov.

###### Specimens examined.

Italy, Sicily, Custonaci, contrada Scurati, crossing with Strada Procinciale 19, 60 m asl, 38°4'55.97"N, 12°40'9.91"E, [Lab ID 51_1, COI: MW758896, ITS2MW757072; Lab ID 51_2, COI: MW758897], W. De Mattia and J. Macor leg., 12.iv.2017. 2 dissected specimens. Italy, Sicily, Custonaci, contrada Scurati, 60 m asl, 38°05'18.10"N, 12°40'17.18"E, [Lab ID 52_1, COI: MW758898; Lab ID 52_2, COI: MW758899, ITS2MW757071, MW757085, MW757086], W. De Mattia and J. Macor leg., 12.iv.2017. 2 dissected spm. Italy, Sicily, Custonaci, contrada Scurati, crossing with Strada Provinciale 18, 65 m asl, 38°4'56.04"N, 12°40'9.86"E, W. De Mattia and J. Macor leg., 12.iv.2017. 2 dissected spm. Italy, Sicily, Custonaci, contrada Scurati, limestone cliffs S of the village, 60 m asl, 38°06'44.89"N, 12°40'34.27"E, W. De Mattia and J. Macor leg., 12.iv.2017. 2 dissected spm. Italy, Sicily, Custonaci, Buffara, NE side of the hollow, 150 m asl, 38°04'0.52"N, 12°41'1.34"E, [Lab ID 49_1, COI: MW758892; Lab ID 49_2, COI: MW758893], W. De Mattia and J. Macor leg., 12.iv.2017. 2 dissected spm. Italy, Sicily, Custonaci, Baglio Messina, 340 m asl, 38°04'6.38"N, 12°41'48.68"E, [Lab ID 50_1, COI: MW758894, ITS2MW757073, MW757074, MW757075, MW757076; Lab ID 50_2, COI: MW758895], W. De Mattia and J. Macor leg., 12.iv.2017. 2 dissected spm.

###### Shell

**(Figs 33.1–33.12, 39.3, 39.4).** shell decollate; whorls ribbed; dorsal keel weak or missing; inferior lamella mostly high; anterior upper palatal plica strong, mostly separated from upper palatal plica, ALPP tending to be shortened but visible; palatal edge of clausilium plate distally not receding, palatal edge distally somewhat or not bent upwards (Nordsieck 2013b).

###### Measurements

(n = 45, decollate): shell height 20.1 ± 0.7, whorl width 4.7 ± 0.3, aperture height 4.7 ± 0.4, aperture width 3.1 ± 0.1.

###### External morphology of the genital organs

**(Figs 32.1, 32.4, 32.6, 32.8).** The FO is remarkably longer than the V (FO/V range 4.1–4.7). The VD is thin along its whole course. The FDBC is longer than the BC+SDBC (FDBC/BC+SDBC range 1.6–1.9). The BC+SDBC is club-like and longer than the V (BC+SDBC/V range 2.1–2.5), with distinction between the SDBC and the BC. The apex is wide and round. The D is much longer than the V (D/V range 4.4–4.9) and longer that the BC+SDBC (D/BC+SDBC range 2.4–2.7), thinner than the BC+SDBC, slim and with a pointed apex. The V is short, wide and cylindrical. The A is very large. The PC is much longer than the V (P+E/V range 3.7–4.2). The PR is short and robust. The ET is not present in all the dissected samples. The E is thinner and shorter than the P (E/P range 0.8–0.9), almost abruptly shrinking and turning into the VD.

###### Internal morphology of the genital organs

**(Figs 32.2, 32.3, 32.5, 32.7, 32.9).** The A shows few large fleshy pleats that are the direct continuation of the penial pleats. The P presents 1–4 longitudinal fleshy pleats. These pleats can be totally smooth (Contrada Scurati), only one or few of them are segmented (metameric) (Contrada Scurati, Dolina Buffara) or the only pleat is proximally segmented and distally irregular (Baglio Messina). The fine structure of the wall is smooth. The PP is always very small and rounded. It is smooth but in Baglio Messina where it is remarkably wrinkly. The P-E transition presents three slightly different structures in the dissected populations. The samples from Dolina Buffara present one ER with the PP and ELP originating from the ER. Its epiphallar formula is: 1ER(PP+ELP). The samples from Scurati show a common structure, namely a first distal ER where the PP originates and the ELP originate from the second proximal ER. The epiphallar formula is: 1ER(PP)+2ER(ELP). The population from Baglio Messina present a slight difference with a first distal ER, whereas the PP and the ELP originate from the second proximal ER. The epiphallar formula is: 1ER+2ER(PP+ELP). The E shows two main longitudinal pleats that can be either smooth or fringed. These two pleats distally originate from a set of smaller irregular pleats. The V can be either completely smooth or with a set of irregular smooth pleats.

**Figure 32.**
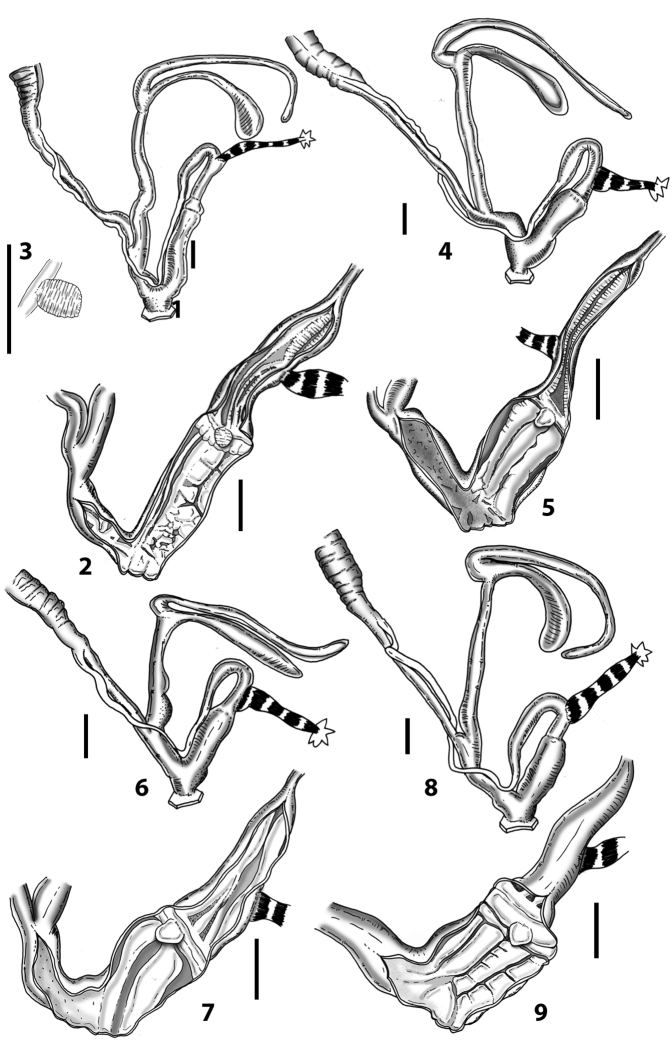
*Sicaniaeminens* (A. Schmidt, 1868), comb. nov., Custonaci, Baglio Messina **32.1** whole distal genital organs **32.2** internal distal part of genital organs **32.3** detail of the pseudopapilla. Dolina Buffara **32.4** whole distal genital organs **32.5** internal distal part of genital organs. Contrada Scurati, Custonaci **32.6** whole distal genital organs **32.7** internal distal part of genital organs. Contrada Scurati toward Mangiapane **32.8** whole distal genital organs **32.9** internal distal part of genital organs.

###### Ecology.

*Sicaniaeminens* comb. nov. inhabits limestone cliffs and boulders, hiding in rock cracks and crevices. It is also found climbing on tree barks and decaying woods (Custonaci, Baglio Messina). This taxon has never been found syntopic with other *Sicania* or *Siciliaria* taxa. Along its eastern distributional range, it is sympatric with *Siciliariacalcaraecalcarae*. According to De Mattia (2017h)*Sicaniaeminens* comb. nov. is considered Endangered following the IUCN criteria B1ab(iv,v)+2ab(iv,v). Approximately 20% of its distribution range (mainly in the southern part) is currently under a heavy quarrying activity. This results in a complete habitat destruction. Only a very small part of its distributional range falls under a protected area: Riserva Naturale Orientata di Monte Cofano, between Monte Cofano and Monte Palatimone. The subpopulations are fragmented and localised over very small areas. This species is assessed as Endangered since it has a very restricted geographic range, it occurs at less than five locations and there is a continuing decline in the quality of the habitat and in the number of mature individuals due to extended periods of drought in the summer. In addition, this species is an extremely sought-after item for shell collectors and traders (De Mattia 2017h).

###### Distribution.

The distribution range of *Sicaniaeminens* comb. nov. includes the southern slopes of Monte Cofano and Monte Palatimone in the north and, in the south, the line from Monte Sparagio-Castellammare del Golfo (east) and Monte Buffara (west). Nordsieck (2013b) includes the surroundings of Calatafimi and Monte Bonifato near Alcamo but recent field research failed to find this taxon in these localities.

##### ﻿ Sicania nobilis

Taxon classificationAnimaliaStylommatophoraClausiliidae

(L. Pfeiffer, 1848), s. l. comb. nov.

2C4C3C95-036E-5AB8-BBC8-7F8317F3B9C1

###### Remarks.

*Sicanianobilis* comb. nov. s. l. has a big, weakly striated shell, mostly decollate and whitish in colour. *Sicanianobilis* comb. nov. forms a well-supported clade in the COI tree. Even in the ITS2 tree almost all sequences cluster, except those of population 14 (SanVito Lo Capo, Torazza, 3 clones), which are found within the *S.eminens* + *S.nobilis* cluster. This might point towards hybridisation between those taxa. Such an assumption would deserve further investigation.

**Figure 33.**
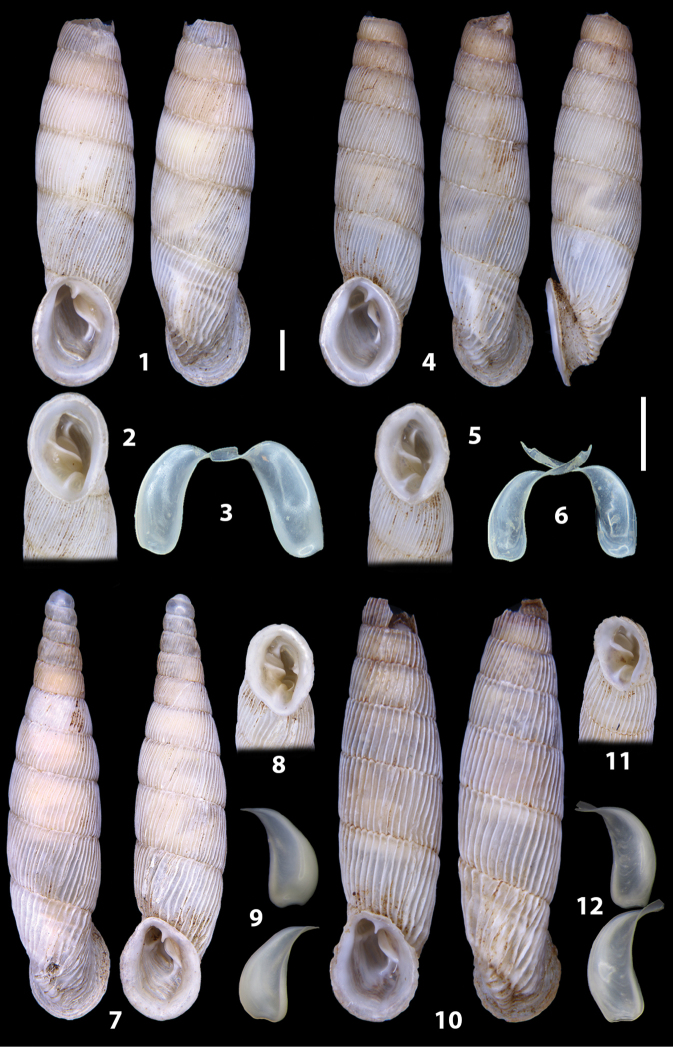
*Sicaniaeminens* (A. Schmidt, 1868), comb. nov., Custonaci, Baglio Messina **33.1** shell. **33.2** detail of the aperture **33.3** clausiliar plate double side. Dolina Buffara **33.4** shell **33.5** detail of the aperture **33.6** clausiliar plate double side. Contrada Scurati, Custonaci **33.7** shell **33.8** detail of the aperture **33.9** clausiliar plate double side. Contrada Scurati toward Mangiapane **33.10** shell **33.11** detail of the aperture **33.12** clausiliar plate double side.

*Sicanianobilis* comb. nov. cannot be framed within a precise genital morphological outline. The anatomy of the genital organs of *Sicanianobilis* comb. nov. exhibits significant differences among the populations (albeit stable within a population), especially concerning the features of the internal sculpturing of V (smooth to transversal pleats), penis (smooth, with tubercles of fringed longitudinal pleats) and the shape of the penial pseudopapilla (small and roundish to big and elongate). As seen in the genital descriptions, also ratios among principal genital parts show great variability. *Sicanianobilisspezialensis* stat. nov. comb. nov. shows a further different genital outline (Fig. 35.1–35.7) (e.g., the internal penis with robust smooth longitudinal pleats) that differs from all the *Sicanianobilis* s. l. dissected populations. Therefore, the morphology of the genital organs cannot serve as a specific diagnostic set of characters, except for *Sicaniaspezialensis* comb. nov., yet, from this taxon only one population was available.

*Sicanianobilis* comb. nov. s. l. is clearly differentiated from the remaining *Sicania/Siciliaria* taxa by shell morphology, especially the waxy whitish surface of the whorls and the arrangement of the plicae and lamellae, as repeatedly reported in literature with detailed descriptions and differential diagnoses (Nordsieck 1984, 2002, 2013b; Beckmann 2004).

##### ﻿ Sicania nobilis nobilis

Taxon classificationAnimaliaStylommatophoraClausiliidae

(L. Pfeiffer, 1848) comb. nov.

17FCECD3-D08C-5498-8418-AC9AA0F90189

Figs 1.F, 34.1–34.8, 36.1–36.13, 39.5–39.7

Clausilia nobilis L. Pfeiffer 1848: 434.
Clausilia sicula Benoit 1876: 151.
Siciliaria (Siciliaria) nobilis episoma Brandt 1961: 7.
Siciliaria (Siciliaria) nobilis – Manganelli et al. 1995: 25.
Siciliaria (Siciliaria) nobilis (= episoma) – Manganelli et al. 1995: 25.
Charpentieria nobilis – Beckmann 2004: 188.
Siciliaria nobilis – Welter-Schultes 2012: 341.
Siciliaria nobilis – Nordsieck 2013b: 8.
Charpentieria nobilis – De Mattia 2017i.

###### Specimens examined.

Italy, Sicily, Castelluzzo, Monte Cofano E of Tonnara di Monte Cofano, 80 m asl, 38°06'22.04"N, 12°40'59.77"E, W. De Mattia and J. Macor leg., 19.xii.2007. 2 dissected spm. I.D. 7. Italy, Sicily, San Vito lo Capo, Castelluzzo, west cliffs E of town, 120 m asl, 38°06'25.71"N, 12°44'33.37"E, [Lab ID 60_1, COI: MW758906, ITS2: MW757065, MW757066, MW757067, MW757139; Lab ID 60_2, COI: MW758907; Lab ID 60_4, COI: MW758938; Lab ID 60_5, COI: MW758939], W. De Mattia and J. Macor leg., 14.iv.2017. 2 dissected spm. Italy, Sicily, San Vito lo Capo, cliffs S of “El-Bahira” camping, 60 m asl, 38°08'48.81"N, 12°44'6.61"E, [Lab ID 62_2, COI: MW758914; Lab ID 62_3, COIMW758940], W. De Mattia and J. Macor leg., 14.iv.2017. 2 dissected spm. Italy, Sicily, San Vito lo Capo, Torre delle Usciere, 5 m asl, 38°10'30.07"N, 12°46'14.09"E, [Lab ID 18_1, COI: MW758918, ITS2: MW757106, MW757107], W. De Mattia and J. Macor leg., 19.xii.2007. 2 dissected spm. “*episoma*” Italy, Sicily, San Vito lo Capo, boulders W of the town, 50 m asl, 38°10'51.85"N, 12°43'37.70"E, [Lab ID 14_1, COI: MW758875, ITS2: MW757128, MW757129, MW757130], W. De Mattia and J. Macor leg., 19.xii.2007. 2 dissected spm.

###### Shell

(**Figs 36.1–36.13, 39.5–39.7).** Shell decollate; with well-developed whitish surface layer; whorls mostly weakly rib-striated, with sutural papillae; dorsal keel missing; inferior lamella moderately high or high; anterior upper palatal plica present, mostly separated from the lunella; ALPP tending to be shortened, posterior lower palatal plica more or less reduced; palatal edge of clausilium plate distally not receding, palatal edge distally somewhat or not bent upwards.

###### Measurements

**(n = 45, decollate).** shell height 22.9 ± 1.6, whorl width 5.0 ± 0.2, aperture height 4.8 ± 0.3, aperture width 3.6 ± 0.4.

###### External morphology of the genital organs

**(Figs 34.1, 34.3, 34.5, 34.7).** The FO is much longer than the V (FO/V range 1.9–3.4). The VD is thin along its whole course. The FDBC is slightly shorter or longer than the BC+SDBC (FDBC/BC+SDBC range 0.9–2.1). The BC+SDBC is club-like to cylindrical and longer than the V (BC+SDBC/V range 1.4–3.1), with no distinction between the SDBC and the BC. The apex is big and round. The D is longer than the V (D/V range 2.1–3.3) and longer that the BC+SDBC (D/BC+SDBC range 1.3–2.0), thinner than the BC+SDBC and with a small but round apex. The V is short, cylindrical and large in diameter. The A is very large. The PC is much longer than the V (P+E/V range 1.8–3.7). The PR is very short and robust. The ET is always present. The E is slightly longer than the P (E/P range 1.2–1.6), very gradually shrinking and turning into the VD.

###### Internal morphology of the genital organs

**(Figs 34.2, 34.4, 34.6, 34.8).** The A is smooth or shows weak large longitudinal pleats. The V shows a variable pattern of sculpturing: from smooth to an irregular pattern of small pleats. The P also presents a variable pattern of sculpturing: totally smooth or with coarse and irregular big tubercles, or one or two longitudinal remarkably segmented pleats. The PP can be either big, elongated with a rounded or pointed apex or small, short, round and totally smooth. The P-E transition presents two different structures. The samples from Cofano (7), San Vito (14) and Torre Usciere (18) present a first distal and a second median ER while both the PP and ELP originates from a third proximal ER. The epiphallar formula is: 1ER+2ER+3ER(PP+ELP). The samples from Castelluzzo (60) and San Vito cliffs (62) present a simpler ephiphallar thickening structure, namely a first distal ER and the PP originates from the second proximal ER. The ELP are not connected to the second proximal ER. The epiphallar formula is: 1ER+2ER(PP)+ELP. The E shows two main longitudinal smooth or fringed pleats that distally fades out or abruptly ends before the VD. These pleats originate from the merging of 3–7 minor distal pleats. The V is smooth.

###### Ecology.

*Sicanianobilisnobilis* comb. nov. is a strictly limestone dweller and inhabits cliffs and big isolated boulders, hiding among rock cracks and crevices. It is found both in dry, sun exposed cliffs (i.e., San Vito lo Capo, cliffs S of ”El-Bahira“ camping) or shady walls under dense tree cover (San Vito lo Capo, Castelluzzo, west cliffs E of town). According to De Mattia (2017i)*Sicanianobilisnobilis* comb. nov. is considered Endangered following the IUCN criteria B1ab(iii,v)+2ab(iii,v).

###### Distribution.

*Sicanianobilisnobilis* comb. nov. is known to occur in two limestone massifs: Monte Cofano and in San Vito lo Capo Peninsula, from Monte Acci to Monte Monaco.

###### Remarks.

*Siciliarianobilisepisoma* hwas described by Brandt (1961: 7) from ”Barbello bei San Vito” by its smaller dimensions, opaque (glanzlose) and markedly striated (scharf rippenstreifigen) shell. Although we were unable to find the locality named Barbello, the description of this taxon corresponds to a population known to inhabit the area east of San Vito lo Capo at Torre dell’Usciere, on limestone cliffs few meters from the shoreline. In the mt tree, this population is positioned within the *Sicanianobilis* clade (Figs 4–6) its COI sequence almost identical to that of an individual from San Vito (ID 14_1). The distinctive shell characters provided by Brandt (1961) reveal to be weak and not reliable to justify the introduction of a new subspecific name. The “opaque” and the “markedly striated” shell surface seems to be a common character of *Sicanianobilis* comb. nov. (see Figs 36.1. 36.4, 36.7 and 36.10, 36.13), as well as the shell’s dimensions that fall inside the variability of *Sicanianobilisnobilis* comb. nov. Moreover, also regarding the morphology of its genital organs, *Sicanianobilis “episoma*” does not show any substantial difference from the nominate subspecies. We therefore support the opinion of Nordsieck (2007: 54) that the name “*episoma*” should be deemed as a junior synonym of *S.nobilisnobilis* comb. nov.

##### ﻿ Sicania nobilis spezialensis

Taxon classificationAnimaliaStylommatophoraClausiliidae

(Nordsieck, 1984), stat. nov. comb. nov.

BD9C4051-F7C0-5EBA-890A-A01BD7B8CEBC

Figs 1.F, 35.1–35.7, 36.14–36.16

Siciliaria (Siciliaria) spezialensis Nordsieck 1984: 202.
Siciliaria (Siciliaria) spezialensis – Manganelli et al. 1995: 25.
Charpentieria spezialensis – Beckmann 2004: 188.
Siciliaria spezialensis – Welter-Schultes 2012: 343.
Siciliaria spezialensis – Nordsieck 2013b: 9.
Charpentieria spezialensis – De Mattia 2017j.

###### Specimens examined.

Italy, Sicily, San Vito Lo Capo, Macari, E side of Monte Speziale, 75 m asl, 38°07'41.78"N, 12°44'9.24"E, [Lab ID 63_1, COI: MW758910, ITS2: MW757039, MW757040; Lab ID 63_2, COI: MW758911], W. De Mattia and J. Macor leg., 14.iv.2017. 4 dissected spm.

###### Shell

**(Figs 36.14–36.16).** Shell decollate; whorls densely ribbed, without or with very weak sutural papillae; dorsal keel absent; inferior lamella moderately high; anterior upper palatal plica absent, posterior upper palatal plica very short; ALPP very short to knob-like, posterior lower reduced; palatal edge of clausilium plate distally not receding, palatal edge distally not bent upwards (see Nordsieck, 1984: 202).

**Figure 34.**
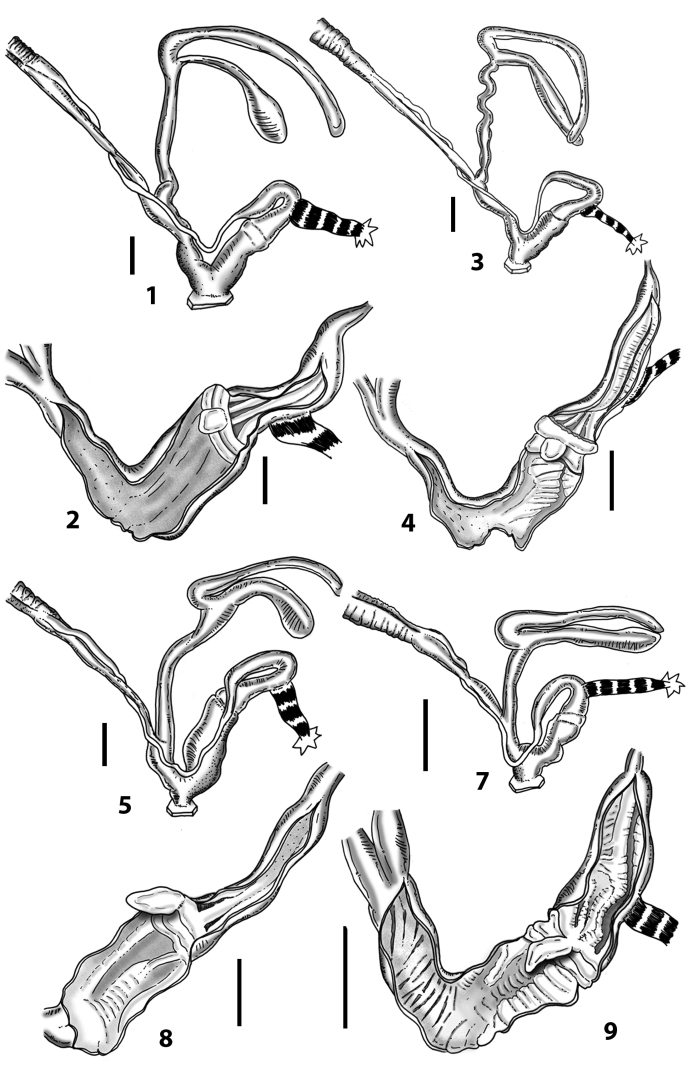
*Sicanianobilisnobilis* (A. Schmidt, 1868), comb. nov., Castelluzzo **34.1** whole distal genital organs **34.2** internal distal part of genital organs. San Vito Lo capo, camping **34.3** whole distal genital organs **34.4** internal distal part of genital organs. San Vito Lo Capo, boulders **34.5** whole distal genital organs **34.6** internal distal part of genital organs. Torre delle Usciere (ex. forma episoma) **34.7** whole distal genital organs **34.8** internal distal part of genital organs.

###### Measurements

**(n = 24, decollate).** shell height 22.2 ± 1.3, whorl width 5.1 ± 0.2, aperture height 4.1 ± 0.3, aperture width 2.8 ± 0.3.

###### External morphology of the genital organs

**(Figs 35.1, 35.3).** The FO is longer than the V (FO/V range 1.9–2.2). The VD is thin along its whole course. The FDBC is slightly longer than the BC+SDBC (FDBC/BC+SDBC range 1.1–1.2). The BC+SDBC is club-like and longer than the V (BC+SDBC/V range 1.7–1.8), with no clear distinction between the SDBC and the BC. The apex is big and rounded. The D is longer than the V (D/V range 2.1–2.3) and longer that the BC+SDBC (D/BC+SDBC range 1.3–1.7), thinner than the BC+SDBC and with a small and round apex. The V is cylindrical. The A is very large. The PC is longer than the V (P+E/V range 1.8–2.0). The PR is short and robust. The E is almost as long as the P (E/P range 0.8–0.9), gradually shrinking and turning into the VD.

###### Internal morphology of the genital organs

**(Figs 35.2, 35.4–35.7).** The V distally presents a set of irregular, weak, and fleshy folds that proximally become a set of oblique smooth pleats. The A shows a set of irregular fleshy folds. The P presents 4–to 6 longitudinal fleshy smooth pleats, slightly depressed in the middle forming a sort of longitudinal hollow. These pleats distally become weak and irregular entering in the A. The fine structure of the wall is smooth. The PP is little, rhombus-shaped and smooth. The P-E transition presents a first distal ER, whereas the PP and the ELP originate from the second proximal ER. The epiphallar formula is: 1ER+2ER(PP+ELP). The E shows a pattern of three or four irregular longitudinal fringed pleats. The wall of the E is smooth.

###### Ecology.

*Sicanianobilisspezialensis* stat. nov., comb. nov. is a strictly limestone dweller and inhabits cliffs, hiding among rock cracks and crevices. As previously deemed as a species, according to De Mattia (2017j) the taxon is Critically Endangered.

###### Distribution.

*Sicanianobilisspezialensis* stat. nov., comb. nov. is known only from the type locality: along the western cliffs of Monte Speziale in the surroundings of Macari (San Vito lo Capo). No overlapping zone with *Sicanianobilisnobilis* stat. nov., comb. nov. is known until now. Although, in its original description, the taxon was reported for one locality only (Nordsieck 1984: 202 “Monte Speziale bei San Vito”), the same author reported later and extended area of distribution (Nordsieck 2013b: “from Macari and Castelluzzo to Monte Speziale and Monte Acci”).

###### Remarks.

*Sicanianobilisspezialensis* stat. nov., comb. nov. is now considered as a subspecies of *S.nobilis*. It is well embedded within the clade of *Sicanianobilis* in both COI and combined trees. *Sicanianobilisspezialensis* stat. nov., comb. nov. is a local isolated, spatially restricted ribbed form of the more widespread *Sicanianobilis* comb. nov. with few different features of the shell, viz. the lack of the anterior upper palatal plica and a clausilium plate that fits into a frame of the palatal plicae.

####### ﻿*Mauritanica* taxonomic part

The genus *Mauritanica* comprises four species that are distributed over northeastern Algeria and central-northern Tunisia (Nordsieck 2002; Sparacio et al. 2020). It is currently considered as a subgenus of *Charpentieria* (MolluscaBase 2021). Only the Tunisian taxa were recently reviewed (Sparacio et al. 2020), mainly by means of shell morphology and a brief analysis of the anatomy of the genital organs, discussing the position of the genus and separating it from *Charpentieria* without involving additional morphological and molecular genetic data.

**Figure:**
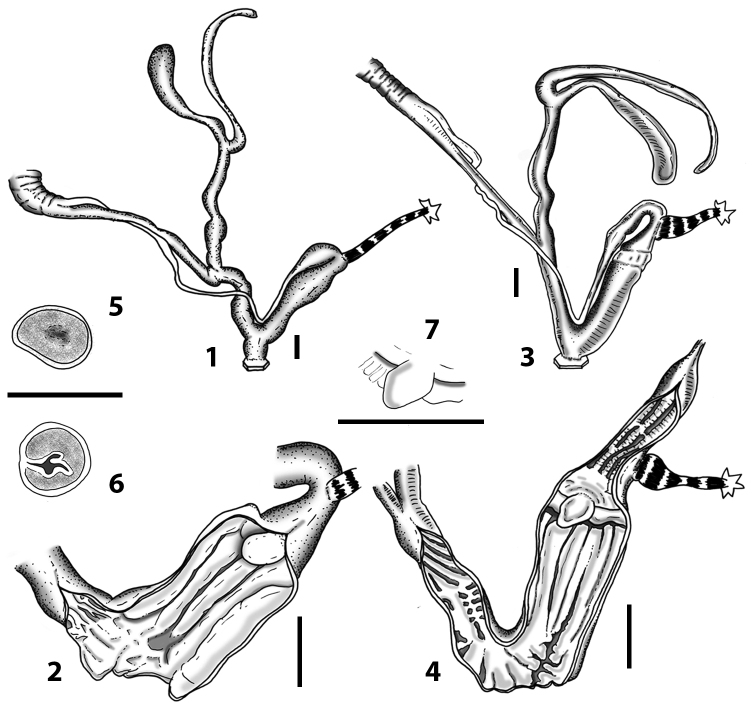
**35.***Sicanianobilisspezialensis* (Nordsieck 1984), stat. nov., comb. nov. Macari **35.1** whole distal genital organs **35.2** internal distal part of genital organs **35.3** whole distal genital organs. **35.4** internal distal part of genital organs **35.5** cross section of the pseudopapilla **35.6** cross section of the epiphallus **35.7** detail of the pseudopapilla.

In our molecular genetic analysis (Figs 4–6) we only had *Mauritanicaperinnipolygyra* available. In the COI, ITS2 and combined trees, this species falls outside the *Siciliaria*/*Sicania* clades, forming a separate independent clade together with *Siciliariascarificata*, endemic to Marettimo. In the COI tree, this clade represents the “sister” clade of *Sicania* (average p distance 17%) and the same is found in the ITS2 tree (Fig. 5), yet with lower support. Despite low sample number, no association with any of the other *Charpentieria* lineages was observed.

**Figure 36.**
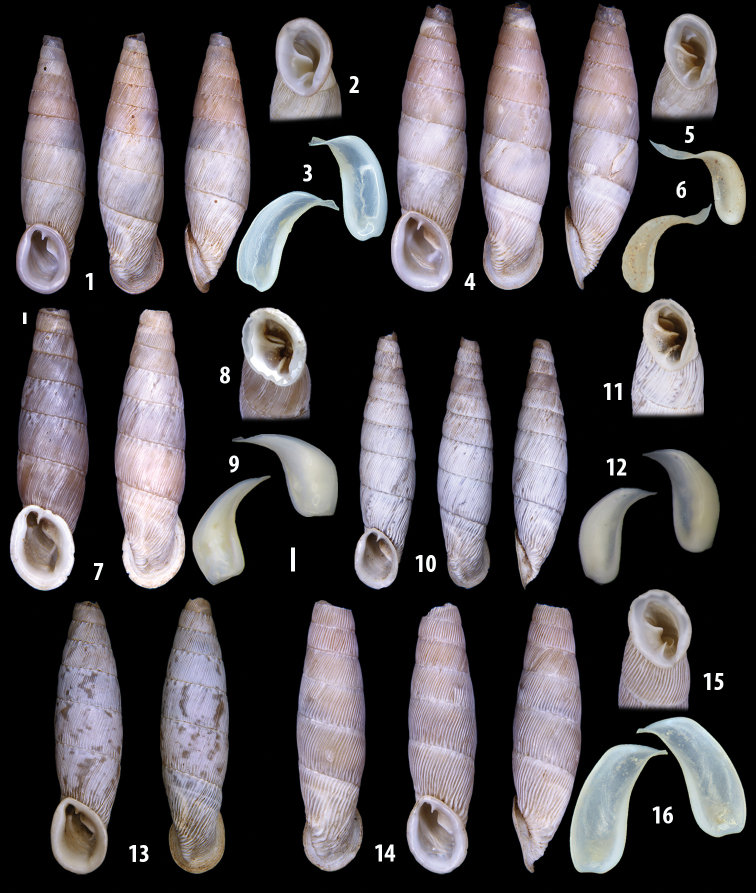
*Sicanianobilisnobilis* (A. Schmidt, 1868), comb. nov., Castelluzzo **36.1** shell **36.2** detail of the aperture **36.3** clausiliar plate double side. San Vito Lo capo, camping **36.4** shell **36.5** detail of the aperture **36.6** clausiliar plate double side. San Vito Lo Capo, boulders **36.7** shell **36.8** detail of the aperture **36.9** clausiliar plate double side. Torre delle Usciere (ex. forma episoma) **36.10** shell **36.11** detail of the aperture **36.12** clausiliar plate double side. Monte Cofano, Tonnara Cofano **36.13** shell. *Sicanianobilisspezialensis* (Nordsieck 1984), stat. nov., comb. nov. Macari **36.14** shell **36.15** detail of the aperture **36.16** clausiliar plate double side.

The separation of *Mauritanica* from *Siciliaria/Sicania* is consistent through all the trees, thus we provisionally keep *Mauritanica* as a distinct genus, although the type species *Mauritanicatristrami* (L. Pfeiffer,1861) was not available to us. In order to fully understand the phylogenetic relationships of *Mauritanica* with *Siciliaria*/*Sicania* more genetic markers and specimens are essential.

Both *M.perinnipolygyra* and *M.scarificata* underwent anatomical investigation. A similar external genital morphology was detected. The only noticeable difference is the relative length of the FO, with a V/FO ratio of 1.1 for *M.scarificata* and 0.6 for *M.perinnipolygyra*. Remarkable differences were detected regarding the internal details of the penial complex. *Mauritanicascarificata* presents 3 ERs with a very short and globose PP that makes it fall into the general *Siciliaria*/*Sicania* genital anatomical arrangement. *Mauritanicaperinnipolygyra* lacks any ER and the PP is irregular with a convolute surface pattern. It is directly connected to the OELP. Unfortunately, the genital descriptions and the pictures provided by Sparacio et al. (2020) do not include fine details of the internal genital organs (ER, PP structure and origin, ELP etc) so that any comparison to our results is impossible.

**Figure 37.**
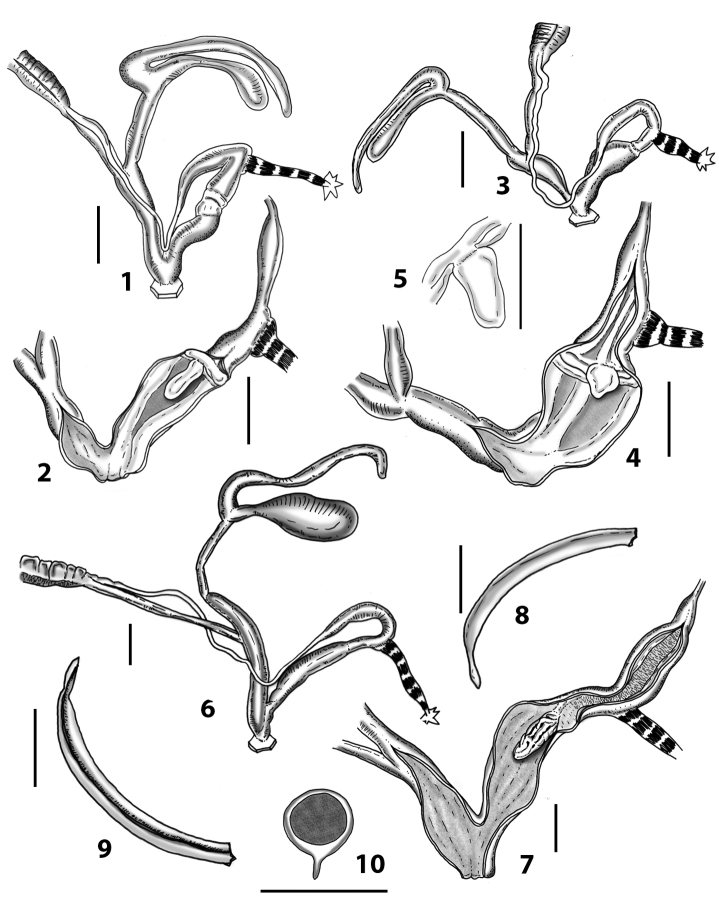
*Mauritanicascarificata* (L. Pfeiffer, 1856), comb. nov., Marettimo **37.1** whole distal genital organs **37.2** internal distal part of genital organs **37.3** whole distal genital organs **37.4** internal distal part of genital organs **37.5** detail of the pseudopapilla. *Mauritanicaperinnipolygyra* (O. Boettger, 1879), Tunisia, Zaghouan **37.6** whole distal genital organs **37.7** internal distal part of genital organs **37.8–37.9** spermatophore **37.10** cross section of the spermatophore.

Following our genital anatomical investigations, the common genital anatomical traits deemed by Nordsieck (2013a, 2013b) to justify his proposal to consider *Mauritanica* as a subgenus of *Siciliaria*, namely, ”the presence of a thickening in the passage from the penis to the epiphallus and the indistinct delimitation between proximal and distal penis“ revealed that it is probably not a reliable feature. The thickening was not found in *Mauritanicaperinnipolygyra* (Fig. 37). Moreover, the delimitation between proximal and distal penis revealed to be an extremely subtle and variable character also in *Siciliaria*, *Charpentieria* and *Stigmatica*. Only few taxa of *Charpentieria* show a detectable, constant and distinct delimitation between proximal and distal penis [e.g., *Charpentieriaitalalorinae* (Fig. 46.7) and *Charpentieriaitalatrepida* (Fig. 49.1)].

##### ﻿ Mauritanica scarificata

Taxon classificationAnimaliaStylommatophoraClausiliidae

(L. Pfeiffer, 1856) comb. nov.

9C6E9DED-1516-512C-9904-114FE0294560

Figs 1.F, 37.1–37.5, 38.1–38.4, 39.8, 39.9

Clausilia scarificata L. Pfeiffer 1856: 185.
Clausilia scarificata – L. Pfeiffer 1859: 765.
Clausilia sacrificata – Benoit 1876: 152.
Clausilia confinata – Benoit 1881: 105.
Clausilia (Siciliaria) confinata Monterosato 1892: 28.
Siciliaria scarificata – Manganelli et al. 1995: 25.
Charpentieria scarificata – Beckmann 2004: 188.
Siciliaria scarificata – Welter-Schultes 2012: 342.
Siciliaria scarificata – Nordsieck 2013b: 7.
Siciliaria scarificata – Liberto et al. 2015: 482.
Charpentieria scarificata – De Mattia 2017k.
Charpentieria scarificata – MolluscaBase eds 2021.

###### Specimens examined.

Italy, Sicily, Marettimo, W of the town, path to Pizzo Falcone. 80 m asl, 37°58'11.08"N, 12°03'59.36"E, [Lab ID 2_1, COI: MW758915, ITS2: MW757112, MW757111; Lab ID 2_2, COI: MW758916], leg. WDM, JM, 12.vi.2015. 29 live spm, 4 dissected spm.

###### Shell

**(Figs 38.1–38.4, 39.8–39.9).** Shell mostly decollate; whorls ribbed, sutural papillae visible; dorsal keel indistinct ore missing; inferior lamella high; anterior upper palatal plica present, separated from or connected with upper palatal plica; palatal edge of clausilium plate distally receding, palatal edge distally somewhat or not bent upwards (as in Nordsieck, 2013b).

###### Measurements

(n = 35, decollate): shell height 18.5 ± 1.0, whorl width 4.6 ± 0.2, aperture height 4.0 ± 0.3, aperture width 2.9 ± 0.2.

###### External morphology of the genital organs

**(Fig. 37.1, 37.3).** The FO is longer than the V (FO/V range 1.8–2.1). The VD is thin along its whole course. The FDBC is slightly longer than the BC+SDBC (FDBC/BC+SDBC range 1.1–1.2). The BC+SDBC is cylindrical to club-like and longer than the V (BC+SDBC/V range 1.3–1.4), with no clear distinction between the SDBC and the BC. The apex is big and round. The D is much longer than the V (D/V range 4.1–4.5) and longer that the BC+SDBC (D/BC+SDBC range 1.4–1.5), thinner than the BC+SDBC and with a small and round apex. The V is V-shaped with a narrow distal part next to the A. The A is large. The PC is longer than the V (P+E/V range 3.0–3.3). The PR is short and robust. The E is longer than the P (E/P range 1.2–1.3), gradually shrinking and turning into the VD.

###### Internal morphology of the genital organs

**(Figs 37.2, 37.4–37.5).** The A is smooth. The P presents two or three big smooth longitudinal pleats that originate directly from the rings of the pseudopapilla. The fine structure of the wall is smooth. The PP is small, smooth and with a pointed apex. It originates from the first proximal ER. The E shows two to four main but smooth ELP that fade before the VD. The wall of the E is smooth. The V is from almost smooth to very finely granulated. The epiphallar formula is: 1ER+ 2ER+3ER(PP)+ELP.

###### Ecology.

*Mauritanicascarificata* comb. nov. is found in a variety of habitats: limestone walls and cliffs, isolated boulders, under rocks and among scree and rocky debris. The species is common and abundant throughout the Island of Marettimo. According to De Mattia (2017k)*Mauritanicascarificata* comb. nov. is considered Least Concern.

###### Distribution.

*Mauritanicascarificata* comb. nov. is endemic of the island of Marettimo.

##### ﻿ Mauritanica perinni polygyra

Taxon classificationAnimaliaStylommatophoraClausiliidae

(O. Boettger, 1879)

464D0C8C-9C0B-5995-90E5-0AE106263C30

Figs 37.6–37.10, 38.5, 38.6

Clausilia (Mauritanica) polygyra Boettger in Kobelt 1879: 153.
Charpenteria (Mauritanica) perinni polygyra – Nordsieck 2002: 27, 29, 35, 36.
Charpenteria (Mauritanica) perinni polygyra – Nordsieck 2007: 54.
Mauritanica perinni polygyra – Sparacio et al. 2020: 318.

###### Remarks.

*Mauritanicaperinnipolygyra* was recently revised by Sparacio et al. (2020: 318). Based on differences of the shell morphology alone, Sparacio et al. (2020: 319) proposed species status for *Mauritanicaperinnipolygyra*, independent from *Mauritanicaperinniperinni* (Bourguignat, 1876). A genital comparison with the nominate subspecies was not possible since the genital organs of this taxon were not provided by Sparacio et al. (2020). Moreover, a comparison between our anatomical results and those provided by Sparacio et al. (2020: 337, figs 60–61) for *Mauritanicaperinnipolygyra* was rather difficult: Essential details, as the internal structure of the epiphallus and the presence/absence of the ER and ELP are not detectable. The fine structure of the internal penis is not clear and, contrary to our results that show a smooth internal wall, the vagina seems to have a smooth longitudinal pleat (2020: 337, fig. 61). The presence of the ET is not clear as well (2020: 337, fig. 60).

###### Specimens examined.

Tunisia, above Zaghouan (road to summit), 789 m asl, 36°22'2.45"N, 10°07'11.81"E, [Lab ID SMF335011_1, COI: MW758966, ITS2: MW757044, MW757045], ex Nordsieck coll. (11639), currently SMF 335011/1, 1 dissected spm.

###### Shell

(**Figs 38.5, 38.6).** Shell decollate or not decollate; whorls finely and irregularly striated, flat, with dense papillae along the sutures; dorsal keel indistinct; inferior lamella high; anterior upper palatal plicae absent; posterior upper palatal plica present; lunella dorso-lateral; little subclaustralis, clausilium plough-like with subrectangular plate (Sparacio et al. 2020: 318).

###### Measurements

**(n = 1, decollate).** shell height 22.4, whorl width 4.1, aperture height 3.3, aperture width 2.4.

###### External morphology of the genital organs

**(Fig. 37.6).** The FO is longer than the V (FO/V = 2.1). The VD is thin along its whole course. The FDBC is slightly shorter than the BC+SDBC (FDBC/BC+SDBC = 0.9). The BC+SDBC is club-like and longer than the V (BC+SDBC/V = 1.4), with clear distinction between the SDBC and the BC. The SDBC is very short. The apex is big and round. The D is much longer than the V (D/V = 1.9) and longer that the BC+SDBC (D/BC+SDBC = 2.2), thinner than the BC+SDBC and with a small and round apex. The V is cylindrical but the presence of the spermatophore may modify its normal shape. The A is small. The PC is longer than the V (P+E/V = 2.8). The PR is long and robust. The ET is not clearly visible. The E is shorter than the P (E/P = 0.9), gradually shrinking and turning into the VD.

###### Internal morphology of the genital organs

**(Fig. 37.7).** The A, V and the P show a very finely granulated surface. The PP is small, elongated. Its surface is very irregular, folded and its internal texture is spongy. The P-E transition is simple, with no ER. The origin of the PP is connected with one of the two ELP. The epiphallar formula is: PP(ELP). The E shows two smooth longitudinal pleats that fade before the VD. Between the ELP an irregular transverse small, interrupted pattern gives an overall coarse appearance.

###### Spermatophore

**(Figs 37.8–37.10).** The spermatophore is thin, elongated. It is 4.7 mm long and 0.8 mm wide at its widest point. The tail is pointed and sharp. The head is missing, probably already digested. The upper keel is simple and goes from the tip of the tail as far as the broken head part. The lower keel is missing.

###### Ecology.

*Mauritanicaperinnipolygyra* is a limestone dweller, on cliffs, crevices and under stones (Sparacio et al. 2020: 319).

###### Distribution.

*Mauritanicaperinnipolygyra* is known only from Djebel Zaghouan area (Sparacio et al. 2020: 319).

**Figure 38.**
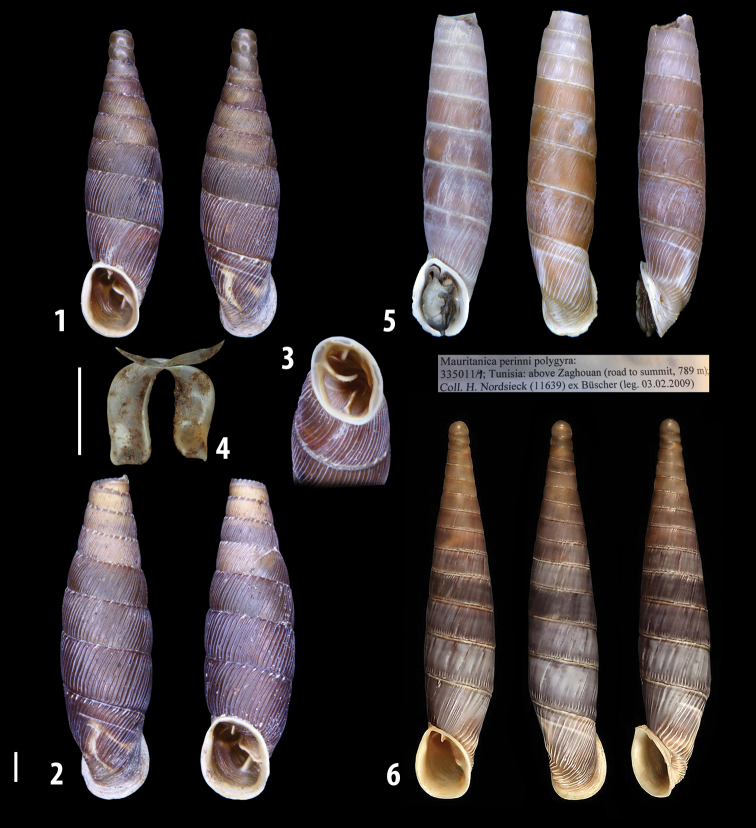
*Mauritanicascarificata* (L. Pfeiffer, 1856), comb. nov., Marettimo **38.1** shell **38.2** shell **38.3** detail of the aperture **38.4** clausiliar plate double side. *Mauritanicaperinnipolygyra* (O. Boettger, 1879), Tunisia, Zaghouan **38.5** shell **38.6** shell (picture courtesy of Claude and Amandine Evanno).

####### An overview of *Siciliaria* related genera *Charpentieria*, *Gibbularia*, and *Stigmatica*

The separation of *Siciliaria* and *Sicania* (and provisionally *Mauritanica*) from the other genera is well supported, but the relationships among *Charpentieria*, *Stigmatica* and *Gibbularia* remain unresolved and appear as polytomy in the trees. *Charpentieria* (four species included) forms one (poorly supported) clade but, the relationships within this clade received no considerable support. The genera *Stigmatica* and *Gibbularia* are not monophyletic and populations from distant areas show high genetic distances (Table 2). *Stigmatica* is based on *Clausiliastigmatica* by tautonomy and, in our trees (Figs 4–6), only this clade with the type species *Stigmaticastigmatica* can be deemed as a representative of this genus. Despite emerging from a polytomy, additional taxa included in our trees and currently considered as belonging to *Stigmatica* (namely *Stigmaticavulcanica* and *Stigmaticapantocratoris*) are clearly separated from the type species. We thus only conventionally keep them as *Stigmatica* species. A focused approach with more genetic markers and specimens is needed to identify the phylogenetic relationships among the species currently included into *Stigmatica* (following MolluscaBase 2021). A similar approach is used for *Gibbularia*, which needs to be excluded from *Siciliaria/Sicania* (Figs 4–6). Its phylogenetic relationship with the other genera is far from being resolved.

**Figure 39.**
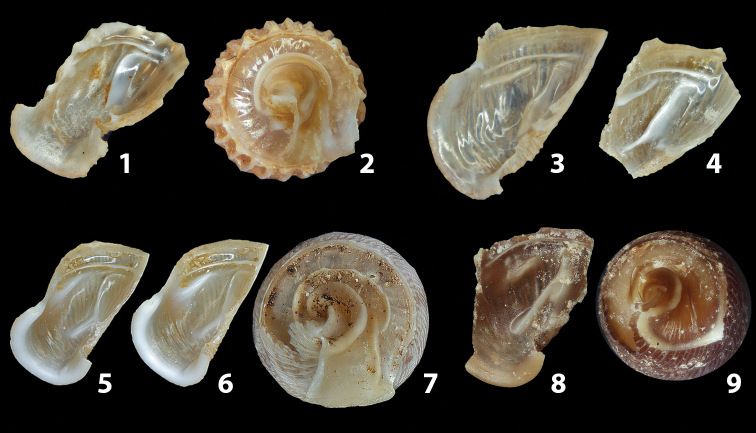
*Sicaniacrassicostata* (L. Pfeiffer, 1856), comb. nov., Monte Cofano **39.1** palatal plicae **39.2** parietal lamellae. *Sicaniaeminens* (A. Schmidt, 1868), comb. nov., Custonaci, Baglio Messina **39.3–39.4** palatal plicae. *Sicanianobilisnobilis* (L. Pfeiffer, 1848), comb. nov., Castelluzzo **39.5–39.6** palatal plicae **39.7** parietal lamellae. *Mauritanicascarificata* (L. Pfeiffer, 1856), comb. nov., Marettimo **39.8** palatal plicae **39.9** parietal lamellae.

*Papillifera*, which was used as outgroup, appeared monophyletic in our trees, even though the distances within this genus are in the same range as among different genera.

####### ﻿Southern alpine *Charpentieria*

The taxa included in the alpine *Charpentieriaitala*–*ornata*–*dyodon* clade show a pan-Southern Alpine distribution, with *C.dyodon* isolated in Piemonte and Ticino, *C.itala* along central and eastern Southern Alps whereas *C.ornata* is present along the easternmost Southern Alps and northwestern Balkans. The taxa included in the *C.stenzii* subclade occupy the eastern Southern Alps, from eastern Trentino-Alto Adige, Friuli-Venezia Giulia, W Slovenia and SE Austria.

Despite the low support values, the COI and the ITS2 phylogenetic trees (Figs 4, 5) show a general picture where *Charpentieriadyodondyodon* (S. Studer, 1820) appears as the sister species of *Charpentieriaitala* (with *C.italaitala*, *C.italabaldensis* and *C.italalorinae*). *Charpentieriastenzii* (with *C.stenziicincta* and *C.stenziiletochana*) represents a separate clade.

The results presented by Scheel and Hausdorf (2012: 3799, 3801) and Xu and Hausdorf (2021: 8) showed that *C.dyodon* is the sister species of the *C.itala* complex whereas *C.stenzii* is sister to *C.ornata*. These results are difficult to compare with our trees, but at least not in contrast.

The stenzioid subspecies of *C.itala* included in our genital anatomical investigations (*clavata*, *variscoi*, *balsamoi*, and *lorinae*) were first assigned to *C.stenzii* (Käufel, 1928) but later transferred to *C.itala* by Nordsieck (1963a, b). In 2007, Nordsieck (2007) transferred these taxa to *C.clavata*. Scheel and Hausdorf (2012), following their molecular genetic results considered the stenzioid *C.clavata* as subspecies of *C.itala*, and thus, its subspecies obtained a new combination with *C.itala*. Scheel and Hausdorf (2012: fig. 3) and Xu and Hausdorf (2021: 6, fig. 5, supplementary table S1) revealed gene flow between *C.stenzii* and the stenzioid subspecies of *C.itala* across the contact zone and also highlighted several mixture events within the *C.itala* complex.

The list of the dissected material (at least two specimens per population) is found in the Table 8.

**Table 8.** Examined taxa of *Charpentieria* with information on shell type (stenzioid/nonstenzioid), availability of genetic data (DNA) and epiphallar formula.

Taxon	Specimens examined	Shell stenzioid/ nonstenzioid	DNA	Epiphallar formula
*Charpentieriadyodondyodon* (S. Studer, 1820)	Trasquera di Iselle, Verbano, Piedmont, Italy. 750 m asl. 46°12'29.53"N, 08°12'46.18"E, I. Niero leg. and det.	-	Y	PP(ELP)
*Charpentieriadyodonalpina* (Stabile, 1859)	Margone, Val di Lanzo, Usseglio, Torino, Piedmont, Italy. 1500 m asl, 45°14'04.89"N, 07°12'18.90"E, leg. and det.	-	N	PP(ELP)
*Charpentieriadyodonthomasiana* (Küster, 1850)	Sentiero per Prà del Vecia, Piedicavallo, Biella, Piedmont, Italy. 1200 m asl. 45°41'32.37"N, 07°58'02.37"E, leg. and det.	-	N	PP(ELP)
*Charpentieriadyodonthomasiana* (Küster, 1850)	Santuario di Oropa, Biella, Piedmont, Italy. 1100 m asl 45°37'49.01"N, 07°58'42.07"E, W. De Mattia and J. Macor leg. and det.	-	N	PP(ELP)
*Charpentieriaornata* (Rossmässler, 1836)	Ogulin city centre, Croatia. 320 m asl. 45°16'2.55"N, 15°13'33.34"E, W. De Mattia and J. Macor leg. and det.	-	N	PP+ELP
*Charpentieriaitalaitala* (G. von Martens, 1824)	San Donato, Barbarano Vicentino, Vicenza, Veneto, Italy. 300 m asl. 45°24'22.30"N, 11°31'11.93"E, W. De Mattia and J. Macor leg. and det.	nonstenzioid	Y	PP(ELP)
*Charpentieriaitalaalbopustolata* (De Cristofori & Jan, 1832)	Brescia, city castle walls, Lombardia, Italy. 200 m asl. 45°32'29.87"N, 10°13'28.37"E, W. De Mattia and J. Macor leg. and det.	nonstenzioid	N	PP(ER+ELP)
*Charpentieriaitalaallatollae* (Käufel, 1928)	Albergo alla Tolla, Val Ampola, Storo, Trentino-Alto Adige, Italy. 720 m asl. 45°51'7.62"N, 10°38'04.51"E, W. De Mattia and J. Macor leg. and det.	nonstenzioid	N	PP(ELP)
*Charpentieriaitalabaldensis* (Strobel, 1851)	Naole, San Zeno di Montagna, Verona, Veneto, Italy. 900 m asl. 45°38'43.67"N, 10°44'15.86"E, I. Niero leg. and det.	nonstenzioid	Y	ER+PP+ELP
*Charpentieriaitalabalsamoi* (Strobel, 1850)	Loc. Galleria, Bracca, Val Serina, Bergamo, Lombardia, Italy. 45°49'24.49"N, 09°43'06.73"E, W. De Mattia and J. Macor leg. and det.	stenzioid	N	PP(ER(ELP))
*Charpentieriaitalaclavata* (Rossmässler, 1836)	Ballabio, Lecco, Lombardia, Italy. 710 m asl. 45°54'25.96"N, 09°25'55.16"E, W. De Mattia and J. Macor leg. and det.	stenzioid	N	PP(ELP)
*Charpentieriaitalalatestriata* (Küster, 1850)	Bedulita, Bergamo, Lombardia, Italy. 625 m asl. 45°47'16.15"N, 09°32'42.26"E, W. De Mattia and J. Macor leg. and det.	nonstenzioid	N	PP(ER+ELP)
*Charpentieriaitalalorinae* (Gredler, 1869)	Val di Lorina, Storo, Brescia, Lombardia, Italy. 600 m asl. 45°50'35.28"N, 10°37'12.47"E, W. De Mattia and J. Macor leg. and det.	stenzioid	Y	ER+PP(ELP)
*Charpentieriaitalazalloti* nom. nov.	Udine, Friuli-Venezia Giulia, Italy. 140 m asl. 46°03'53.37"N, 13°14'12.35"E, L. Anzil leg., W. De Mattia det.	nonstenzioid	N	PP(ER(ELP))
*Charpentieriaitalazalloti* nom. nov.	Udine, Friuli-Venezia Giulia, Italy. 140 m asl. 46°03'53.37"N, 13°14'12.35"E, L. Anzil leg., W. De Mattia det.	nonstenzioid	N	PP(ER(ELP))
*Charpentieriaitalaserravalensis* (Nordsieck, 1963)	Serravalle near Vittorio Veneto, Veneto, Italy. 500 m asl. 46°00'04.46"N, 12°17'11.13"E, W. De Mattia and J. Macor leg. and det.	nonstenzioid	nonstenzioid	PP(ER(ELP))
*Charpentieriaitalatrepida* (Käufel, 1928)	Forte Cima Ora, Anfo, Brescia, Lombardia, Italy. 1500 m asl. 45°47'50.57"N, 10°28'03.60"E, W. De Mattia and J. Macor leg. and det.	stenzioid	N	PP(ELP)
*Charpentieriaitalatriumplinae* Nardi, 2011	Cima Caldoline, Lavenone, Brescia, Lombardia, Italy. 1700 m asl. 45°48'03.54"N, 10°24'20.12"E, G. Nardi leg. and det.	stenzioid	N	PP(ER(ELP))
*Charpentieriaitalavariscoi* (Pini, 1883)	Lavina, Val Taleggio, Bergamo, Lombardia, Italy. 650 m asl. 45°53'11.73"N, 09°33'55.46"E, W. De Mattia and J. Macor leg. and det.	stenzioid	N	PP(ER+ELP)
*Charpentieriastenziistenzii* (Rossmässler, 1836)	San Romedio, Trento, Italy. 730 m asl. 46°22'03.83"N, 11°06'31.84"E, W. De Mattia and J. Macor leg. and det.	stenzioid	N	EP(ELP)
*Charpentieriastenziibutoti* (Bank, 1987)	Fai della Paganella, Trento, Trentino-Alto Adige, Italy. 600 m asl. 46°12'57.04"N, 11°04'22.88"E, W. De Mattia and J. Macor leg. and det.	stenzioid	N	EP(ELP)
*Charpentieriastenziicincta* (Brumati, 1838)	Bosplans, Pordenone, Friuli-Venezia Giulia, Italy. 500 m asl. 46°11'58.11"N, 12°38'5.24"E, W. De Mattia and J. Macor leg. and det.	stenzioid	Y	EP(ELP)
*Charpentieriastenziicincta* (Brumati, 1838)	Val Resia, sella di Carnizza, Tarvisio, Friuli-Venezia Giulia, Italy. 400 m asl. 46°20'15.55"N, 13°19'17.11"E, W. De Mattia and J. Macor leg. and det. Mattia leg. and det.	stenzioid	N	EP(ELP)
*Charpentieriastenziifaueri* (Bank, 1987)	Grotte di Oliero Valstagna, Vicenza, Veneto, Italy. 800 m asl. 45°50'47.79"N, 11°40'04.24"E, W. De Mattia and J. Macor leg. and det.	stenzioid	N	EP(ELP)
*Charpentieriastenziiletochana* (Gredler, 1874)	Val Fonda, Misurina, Belluno, Veneto, Italy. 1500 m asl. 46°36'27.42"N, 12°12'20.01"E, W. De Mattia and J. Macor leg. and det.	stenzioid	Y	EP(ELP)
*Charpentieriastenziinordsiecki* Fauer, 1991	Cison di Val Marino, Treviso, Veneto, Italy. 250 m asl. 45°58'05.53"N, 12°08'52.60"E, W. De Mattia and J. Macor leg. and det.	stenzioid	N	no pseudopapilla
*Charpentieriastenziiparoliniana* (De Betta & Martinati, 1855)	Loc. La Goccia, Foza, Valstagna, Vicenza, Veneto, Italy. 800 m asl. 45°52'29.20"N, 11°38'59.73"E, W. De Mattia and J. Macor leg. and det.	stenzioid	N	EP(ELP)
*Charpentieriastenziiwesterlundi* (Nordsieck, 1993)	Val Fiscalina, Croda Rossa, Sesto, Bolzano, Trentino-Alto Adige, Italy. 1500 m asl. 46°38'02.83"N, 12°21'42.79"E, I. Niero leg. and det.	stenzioid	N	EP(ELP)

The present anatomical investigation indeed generated consistent results that are congruent with the molecular genetic outcomes.

**Figure 40.**
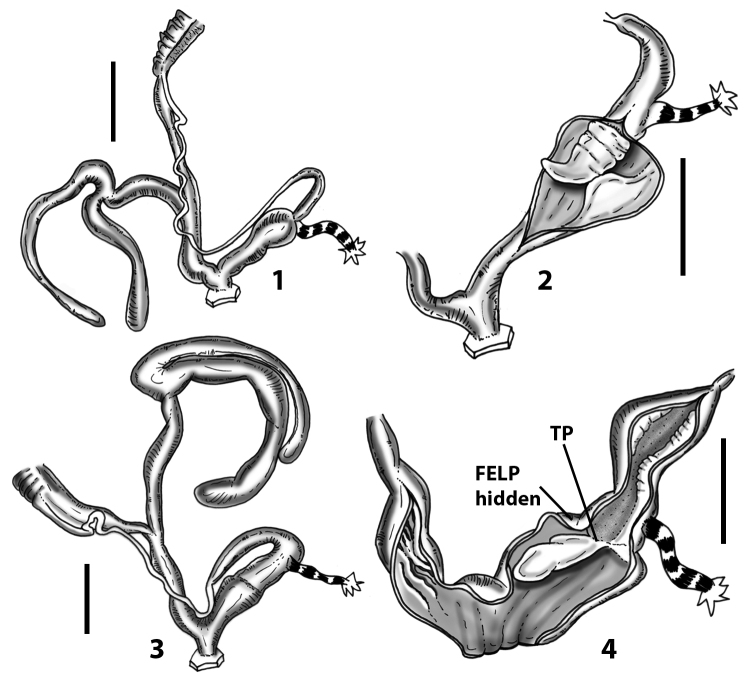
*Charpentieriadyodondyodon* (S. Studer, 1820), Verbano **40.1** whole distal genital organs **40.2** internal distal part of genital organs. *Charpentieriadyodonalpina* (Stabile, 1859). Val di Lanzo, Usseglio **40.3** whole distal genital organs **40.4** internal distal part of genital organs.

**Figure 41.**
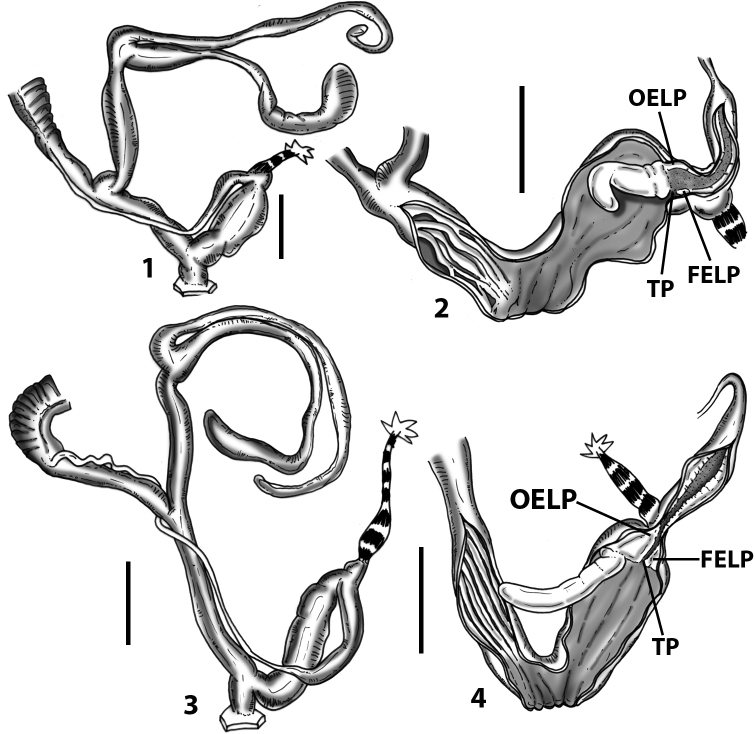
*Charpentieriadyodonthomasiana* (Küster, 1850), Piedicavallo **41.1** whole distal genital organs **41.2** internal distal part of genital organs. Oropa, Biella **41.3** whole distal genital organs **41.4** internal distal part of genital organs.

*Charpentieriaitala*–*ornata*–*dyodon* and *C.stenzii* groups of taxa show the same external overall shape of the distal genital organs (Figs 40, 41, 43–46, 48, 49, 51–55). No major difference can be observed regarding the main genital parts as, e.g., the shape and relative dimensions of the OSD, FO, A and penial complex (P+E). Only the V is shorter in *C.stenzii*, with an average P/V ratio of 1.4 for *C.itala* and 2.3 for *C.stenzii*. Moreover, this general external genital arrangement of *Charpentieria* is not distinguishable by any means from *Siciliaria*, *Sicania*, and *Mauritanica*.

The genus *Charpentieria* was introduced by Stabile (1864: 80) [type species: *Charpentieriadyodon* (S. Studer, 1820)]. The type species was subsequently designated by Kennard and Woodward (1923: 306). Boettger (1877: 53) listed only *C.dyodon* and its subspecies for the genus *Charpentieria*.

Boettger (1877: 35) introduced the name *Tirolica* as ”Gruppe der *Stentzi*“ (type species: *Delimastenzii* = Charpentieria*stenzii*), that included most of the stenzioid southern alpine taxa known in 1877. This genus included taxa currently belonging to both *Charpentieriaitala* (*C.i.balsamoi* and *C.i.lorinae*) and *C.stenzii* (*C.s.stenzii* and its synonym *Clausiliasaccata* Küster, 1848, *C.stenziicincta* and its synonyms: *Clausiliarossmaessleri* L. Pfeiffer, 1841, Clausiliacinctavar.maior Rossmässler, 1842 and Clausiliacinctavar.minor Westerlund, 1878 and finally *C.s.letochana* and its synonym *Clausiliafunki* Gredler, 1874). Inexpicably, Boettger (1877:35) did not include the stenzioid *C.stenziiparoliniana* (De Betta & Martinati, 1855) and *C.italaclavata*. Xu and Hausdorf (2021: 6), after sequencing 843 loci, found that *C.stenzii* is well embedded in *Charpentieria*. Although our anatomical investigations revealed a peculiar internal structure of the P–E transition and the penial pseudopapilla is found exclusively in *C.stenzii* (we named it hemipapilla, see point 6.6 for a detailed decription), we follow Xu and Hausdorf (2021) in not separating this group on generic level. The hemipapilla was not found in the stenzioid subspecies despite the notable gene flow with *C.stenzii*, as proven by Xu and Hausdorf (2021: 7).

Boettger, 1877, introduced the genus *Itala* as “Gruppe der *itala*” (type species: *Delimaitala* = Charpentieria*itala*), including all the alpine non-stenzioid taxa, excluding *C.dyodon* but including two stenzioid taxa: *C.stenziiparoliniana* and *C.italaclavata*. He also included two taxa from the Balkans: *Delimaconspersa* [= *Strigilodelimaconspersa* (L. Pfeiffer, 1848)] and its synonym *Delimaplatystoma* Küster, 1850. As for *Tirolica*, the recent results by Xu and Hausdorf (2021: 6–8) indicated that the *Charpentieriaitala* complex should not be separated on a (sub)generic level (i.e., *Itala*).

Wagner (1925: 17) introduced the genus subgenus Alpidelima [type species *Clausiliaitala* Martens, 1824, by subsequent designation by Schileyko (2000: 664)] that must be deemed as a junior synonym of *Charpentieria*.

Nordsieck (1963a: 95, figs 9–10; 97, fig. 11; 1963b: 185, 189, 193, 197) depicted the genital organs of some taxa currently belonging to the genus *Charpentieria* from the Alps. The sketchy drawings lacked important details, both concerning internal and external features. Except for fig. 11a of Nordsieck (1963a), the exact position of the insertion of the proximal vas deferens into the beginning of the free oviduct was not clear. The internal structure of the penis-epiphallus transition was not investigated. The pseudopapilla was shown, but its origin was missing as well as its connection to (and the presence/absence of) the epiphallar ring(s) and the epiphallar longitudinal pleats. Only in Nordsieck (1963a: fig. 11b) the origin of the pseudopapilla and the epiphallar ring seem to be roughly depicted. Nordsieck (1963a) reported the presence of the penial flagellum for all the four taxa, which is in contrast to our results, based on 63 specimens, where this particular anatomical structure was never detected despite careful check.

He introduced a new subspecific taxon, *C.italaserravalensis* (1963a: 175), but depicted its external anatomy of the genital organs [Nordsieck, 1963b: 184–185, Abb. 47 *italaserravalensis*, Serravalle (präp. 109)] with the same genital drawing provided for *C.italaitala* in Nordsieck, (1963a: 97, fig. 11a, b). The shell description, genital organs and measures provided for *C.italaserravalensis* (Nordsieck 1963b: 175) match with the morphology of the specimens from Colli Euganei (San Donato, Barbarano Vicentino, Padova, Italy) (Fig. 44.1–44.4) studied in the present paper.

Nordsieck cites *C.italaitala* (1963a) but unexpectedly, Nordsieck (1963b) neither cited the nominate subspecies, nor did he provide information about the type locality of the species

*Charpentieriaitala* was described by G. von Martens (1824: 442), but he did not provide any indication about the collecting site. L. Pfeiffer (1853: 606) reports the description of the species given by Martens. In his ”Reisebemerkungen über einige Binnenschnecken Italiens“, E. von Martens (1857: 129), the son of G. von Martens, finally gives precise indications where his father collected the first series of specimens of *C.itala* (between Mira and Dolo): ”Zwischen Mira und Dolo hatte mein Vater auch früher seine *Clausiliaitala*.”. Dolo and Mira are located in the province of Venice, between Venice and Padova, along the river Brenta in the Pianura Padana. Being it a quaternary alluvial plain area, the specimens were probably collected on walls and houses. Eduard von Martens (1857: 129) collected more samples of *C.itala* from the walls of the Botanic Garden of Padova. These alluvial plain populations probably originate from downstream transport by creeks, deriving from the Colli Euganei and Colli Berici, which flow into the Brenta River.

####### ﻿Brief genital anatomical description of *Charpentieriadyodon* subspecific taxa: the parapseudopapilla

*Charpentieriadyodon* is the name-bearing species of the genus *Charpentieria*. It comprises five subspecies [*C.dyodondyodon* (Studer, 1820; *C.dyodonalpina* (Stabile, 1859); *C.dyodonpaulucciana* (Pollonera, 1885); *C.dyodonstuderi* (Pini, 1885) and *C.dyodonthomasiana* (Küster, 1850)]. These taxa are distributed over an area that includes the western Southern Alps of Piemonte, Italy (from Dora Riparia River to Lago Maggiore) and the South Switzerland (Ticino and Valais). This species is not sympatric with any other *Charpentieria* species.

In our COI tree only the nominate subspecies *Charpentieriad.dyodon* was included (Figs 4–6). It emerged as the sister species of *C.itala*, yet with only low node support.

Three out of five subspecies of *C.dyodon* were anatomically investigated (Table 8). They revealed a common overall genital arrangement (Figs 40, 41), including a stable presence of the penial pseudopapilla. The pseudopapilla can be either simple or rooted. The internal wall of the penis is smooth, the epiphallus shows two fringed longitudinal pleats and the internal wall of V presents many smooth longitudinal pleats. *Charpentieriadyodon* has a distribution detached from *C.itala* with no overlapping areas. *Charpentieriadyodon* shows substantial differences of the shell characters (smooth or weakly striated surface, lunella mostly absent and no palatal folds, sutural papillae very scarce or absent) and epiphallar structure (see the following chapter).

The four populations of *Charpentieriadyodon* included for dissection, revealed a very stable morphology of the genital organs (Figs 40, 41). The internal surface of the P is smooth and the V presents many smooth longitudinal pleats that stop before entering the atrium. The external transition between P and E is always visible. *Charpentieriadyodon* revealed a peculiar morphology of the pseudopapilla and the internal transition area between penis and epiphallus (Figs 40, 41). The parapseudopapilla involves the pseudopapilla and the epiphallar longitudinal pleats: the pseudopapilla has its origin at the distal end of one epiphallar longitudinal pleat (“occupied“ epiphallar longitudinal pleat) and the opposite wall is delimited and narrowed by the other epiphallar longitudinal pleat (“free“ epiphallar longitudinal pleat) and connected by a ER, creating a funnel-like structure. The PPP, at a first glance, resembles a true penial papilla, but it remarkably lacks the seminal channel. The extremely large ER of the PPP completely obstructs the lumen of the transition area. The connection between the penis and epiphallus is represented by a short, narrow passage, named “epiphallar funnel” (EF), that is found at the base of the PPP and it is quite difficult to detect.

This parapseudopapilla represents an intermediate form between the simple structure of *Charpentieriaitala* and the hemipapilla (HP) of *Charpentieriastenzii*. In the normal PP the transition stretch E is relatively wide and the counter wall opposite of the PP is represented by the simple P-E transition wall.

##### ﻿ Charpentieria dyodon dyodon

Taxon classificationAnimaliaStylommatophoraClausiliidae

(S. Studer, 1820)

A951C547-BBFD-5059-BF0B-6A56E2CC7BFA

Figs 40.1, 40.2, 42.1

###### Distribution.

*Charpentieriadyodon* s. l. has a limited distribution, restricted to northern Piedmont and adjacent Swiss territories. The nominate subspecies is restricted to the surroundings of Iselle (Piedmont) and adjacent Swiss localities.

###### Specimens examined.

Italy, Piedmont, Trasquera di Iselle, Verbano. 750 m asl, 46°12'29.53"N, 8°12'46.18"E, [Lab ID 44_1, COI: MW758956. ITS2: MW757051, MW757052; Lab ID C44_3, COI: MW758951], I. Niero leg. and det., 2 dissected spm.

###### External morphology of the genital organs (Fig. 40.1).

The FO is slightly longer than the V. The FDBC is shorter the BC+SDBC (SDBC+BC). The BC+SDBC is club-like and longer than the V with no distinction between the SDBC and the BC. The apex is round. The D is slightly longer than the BC+SDBC, thinner with a small but round apex. The V is short, cylindrical and large in diameter. The PC is much longer than the V. The PR is short and robust. The E is shorter than the P and very thin.

###### Internal morphology of the genital organs (Fig. 40.2).

The A and the P are smooth, with a very fine granulated sculpturing. The V shows many smooth longitudinal pleats. The smooth PP is simple, big, conical with a pointed apex. Its base is partially connected to the ELP and transversally extends along the transition wall, narrowing the transition passage. The epiphallar formula is: PP(ELP). The E shows two main longitudinal moderately fringed pleats that proximally fade before the VD.

##### ﻿ Charpentieria dyodon alpina

Taxon classificationAnimaliaStylommatophoraClausiliidae

(Stabile, 1859)

E5F0EB81-453B-5652-BEA2-CFB73B2FDB53

Figs 40.3–40.4, 42.2

###### Distribution.

*Charpentieriadyodonalpina* is found in scattered populations along the Val di Lanzo, NW of Torino (Piedmont).

###### Specimens examined.

Italy, Piedmont, Torino, Margone, Val di Lanzo, Usseglio. 1500 m asl, 45°14'4.89"N, 07°12'18.90"E, leg. and det., 2 dissected spm.

###### External morphology of the genital organs

**(Fig. 40.3).** The FO is almost as long as the V. The FDBC is slightly shorter than the BC+SDBC (SDBC+BC). The BC+SDBC is cylindrical and longer than the V with no clear distinction between the SDBC and the BC. The apex is round. The D is shorter than the BC+SDBC, thinner with a round apex. The V is as long as the first duct of the BC+SDBC. The PC is 2 × as long as the V. The PR is long and robust. The E is slightly longer than the P and thin.

###### Internal morphology of the genital organs

**(Fig. 40.4).** The A and the P are smooth, with a very fine granulated sculpturing. The V shows many smooth longitudinal pleats. The smooth simple PP is simple, big, club-like with a round apex. Its base is partially connected to the ELP and transversally extends along the transition wall, narrowing the transition passage. The epiphallar formula is: PP(ELP). The E shows two main longitudinal moderately fringed pleats that proximally fade before the VD.

##### ﻿ Charpentieria dyodon thomasiana

Taxon classificationAnimaliaStylommatophoraClausiliidae

(Küster, 1850)

418C1406-9E19-5FBC-B8E7-E2B00CAC0B07

Figs 41.1–41.4, 42.3, 42.4

###### Specimens examined.

Italy, Piedmont, Biella, Santuario di Oropa. 1100 m asl 45°37'49.01"N, 07°58'42.07"E, W. De Mattia and J. Macor leg. and det., 3 dissected spm. Italy, Piedmont, Biella, Sentiero per Prà del Vecia, Piedicavallo. 1200 m asl, 45°41'32.37"N, 07°58'2.37"E, leg. and det., 2 dissected spm.

###### Distribution.

*Charpentieriadyodonthomasiana* is found in the surroundings of Oropa and Piedicavallo, surroundings of Biella (Piedmont).

###### External morphology of the genital organs

**(Fig. 41.1, 41.3).** The FO is half as long as the V. The FDBC is shorter than the BC+SDBC (SDBC+BC). The BC+SDBC is club-like and longer than the V with slight distinction between the SDBC and the BC. The apex is pointed. The D is slightly longer than the BC+SDBC, thinner with a round apex. The V is long and cylindrical. The PC is longer than the V. The PR is long and robust. The E is slightly shorter than the P and thin.

###### Internal morphology of the genital organs

**(Fig. 41.2, 41.4).** The A and the P are smooth, with a very fine granulated sculpturing. The V shows many smooth longitudinal pleats. The smooth simple PP is simple, elongated with a pointed apex. Its base is partially connected to the ELP and transversally extends along the transition wall, narrowing the transition passage. The epiphallar formula is: PP(ELP). The E shows two main longitudinal moderately fringed pleats that proximally fade before the VD.

####### ﻿Brief genital anatomical description of the subspecific taxa of *Charpentieriaornata* and *Charpentieriaitala*

The Southern Alpine *C.itala* is distributed from Como Lake to the Garda Lake, Trentino, Veneto (including Colli Berici and Colli Euganei) and the Pre-Alps of Friuli. Nordsieck (1963b) provided, for the eastern border of its distribution, the axis Pordenone-Ampezzo. Recent collecting extended its eastern distribution as far Udine and Cividale in Friuli (WDM, personal unpublished data). Its eastern distribution range overlaps with *C.stenzii* and in few localities they are found syntopic (e.g., San Romedio, Trento, where *C.stenziistenzii* and *C.italaserravalensis* are found syntopic). Concerning the nomenclature of *C.itala*, we adopted the system by Scheel and Hausdorf (2012) that considered *C.clavata* as a subspecies of *C.itala*.

**Figure 42.**
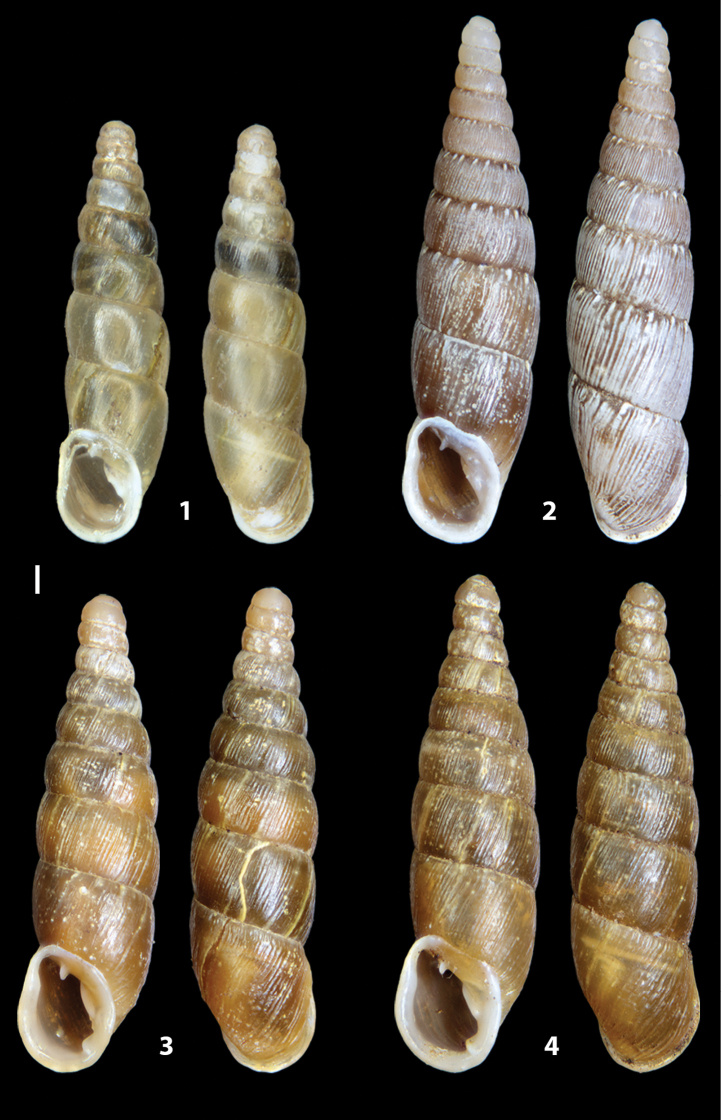
*Charpentieriadyodondyodon* (S. Studer, 1820), Verbano **42.1** shell. *Charpentieriadyodonalpina* (Stabile, 1859). Val di Lanzo, Usseglio **42.2** shell. *Charpentieriadyodonthomasiana* (Küster, 1850), Piedicavallo **42.3** shell. Oropa, Biella **42.4** shell.

Its shell is weakly striated to frankly ribbed, with evident sutural papillae and a strong lunella, which is remarkably curved. Twelve out of the seventeen currently valid subspecies of *C.itala* were anatomically investigated, revealing a common overall arrangement of the genital organs. The pseudopapilla can be either smooth or markedly wrinkled and occupies almost the whole penial volume.

##### ﻿ Charpentieria ornata

Taxon classificationAnimaliaStylommatophoraClausiliidae

(Rossmässler, 1836)

68D3F251-358C-571B-AC66-875150A79565

Figs 43.1, 43.2, 47.1

###### Distribution and remarks.

*Charpentieriaornata* has a wide distribution that includes Sudetes, southeastern Alps (Slovenia) and the western Dinarids (Welter-Schultes 2012). This species has a detached distribution which is not overlapping with *C.itala* and it is also frequently found (as the latter species) in disturbed habitats (walls, artificial limestone cliffs etc), revealing a good degree of anthropophily. *Charpentieriaornata* presents, for the subgenus, an unusually short pseudopapilla. Moreover, contrary to *C.itala*, the peristome is never detached from the body whorl.

###### Specimens examined.

Croatia, Ogulin city centre, 320 m asl, 45°16'2.55"N, 15°13'33.34"E, W. De Mattia and J. Macor leg. and det. 3 dissected spm.

###### External morphology of the genital organs

**(Fig. 43.1).** The FO is almost as long as the V. The FDBC is as long as the BC+SDBC. The BC+SDBC is club-like to cylindric in shape, with slight distinction between the SDBC and the BC. The apex is wide and rounded. The D is slightly longer than the BC+SDBC with a round apex. The V is long and slim. The PC is only 1.5 × longer than the V. The P is swollen. The PR is short and robust. The P-E transition is clearly visible. The E is as long as the P and much thinner in diameter.

###### Internal morphology of the genital organs

**(Fig. 43.2).** The V is smooth. The P presents big irregular longitudinal pleats that reach the A. The pleats are smooth and discontinuous, irregularly splitting and merging one into another. The PP is smooth and short, almost triangular and doesn’t reach the A. It is detached from the ELP. The epiphallar formula is: PP+ELP. The ELP are small and smooth. They proximally fade before the VD.

**Figure 43.**
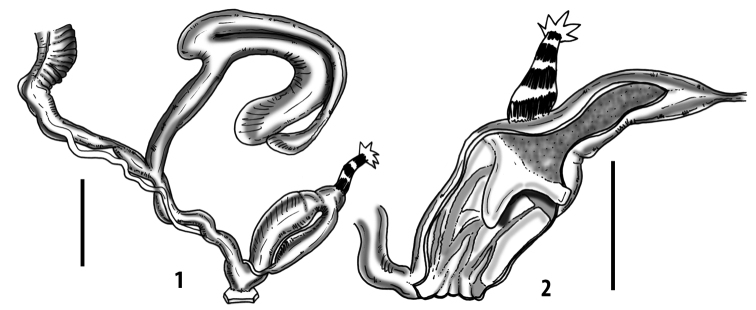
*Charpentieriaornata* (Rossmässler, 1836), Ogulin, HR **43.1** whole distal genital organs **43.2** internal distal part of genital organs.

##### ﻿ Charpentieria itala itala

Taxon classificationAnimaliaStylommatophoraClausiliidae

(G. von Martens, 1824)

65540387-DF0A-5C9D-A3E4-E86EB55C6BB9

Figs 44.1–44.4, 47.2–47.4

###### Distribution and remarks.

The actual distribution of *C.italaitala* depends on the status and validity of other subspecific taxa with similar shell morphology, especially *C.italaserravalensis* or other available names of uncertain validity as *Clausiliaitalavicentina* Schmidt, 1868 or *Clausiliaalboguttulata* var. a, *major* Pirona, 1865. Following the original descriptions (Pirona 1865: 693; Schmidt 1868: 47), both taxa probably have “overlapping“ distribution with the nominate subspecies, being cited for the surroundings of Vicenza and the western Friuli plain. According to the current systematics, *C.italaitala* is distributed in the Pianura Veneta (Venetian Plain), Colli Euganei and Berici, including the Prealpine hills of Vicenza.

###### Specimens examined.

Italy, Veneto, Vicenza, San Donato, Barbarano Vicentino. 300 m asl, 45°24'22.30"N, 11°31'11.93"E, [Lab ID C1_1, COI: MW758952], W. De Mattia and J. Macor leg. and det., 2 dissected spm.

###### External morphology of the genital organs

**(Fig. 44.1).** The FO is almost as long as the V. The FDBC is longer than the BC+SDBC. The BC+SDBC is club-like, with visible distinction between the SDBC and the BC. The apex is rounded. The D is slightly longer than the BC+SDBC with a round apex. The V is long and partially swollen. The PC is only slightly longer than the V. The P is short and remarkably swollen. The PR is long and robust. The E is slightly longer than the P and thinner in diameter.

###### Internal morphology of the genital organs

**(Fig. 44.2).** The A and the P present big transversal pleats. The pleats are smooth and discontinuous along the distal part of the P and in the A. The V is smooth. The PP is smooth, elongated with a round apex and directly connected to the ELP. The epiphallar formula is: PP(ELP). The E shows two ELP, fringed at their distal ends. They proximally fade before the VD.

##### ﻿ Charpentieria itala albopustolata

Taxon classificationAnimaliaStylommatophoraClausiliidae

(De Cristofori & Jan, 1832)

EF1E1095-BB4C-5F9E-ABA2-2CC742733BE7

Figs 44.5, 44.6, 47.5

###### Distribution.

*Charpentieriaitalaalbopustolata* is widely distributed throughout the Central Alps of Lombardia (Nardi 2011: 101) and a small area of southwestern Veneto, next to the southern part of Garda Lake (F. Scarlassara pers. comm.) and Verona.

###### Specimens examined.

Italy, Lombardia, Brescia, city castle walls. 200 m asl, 45°32'29.87"N, 10°13'28.37"E, W. De Mattia and J. Macor leg. and det., 2 dissected spm.

###### External morphology of the genital organs

**(Fig. 44.5).** The FO is almost 2 × as long as the V. The FDBC is much longer than the BC+SDBC. The BC+SDBC is club-like, with visible distinction between the SDBC and the BC. The apex is rounded. The D is slightly longer than the BC+SDBC, thin and with a round apex. The V is long and thin. The PC is double as long as the V. The P is long and swollen at its proximal part. The PR is long and thin. The E is very short and thin in diameter. The overall appearance of the external morphology of the genital organs is slim and elongated.

###### Internal morphology of the genital organs

**(Fig. 44.6).** The A and the P are smooth, with very loose and barely visible longitudinal elevations. The V is smooth. The PP is folded, elongated with a pointed apex. The ER is present and it is connected with the ELP by means of 5 or 6 small longitudinal pleats. The PP originates from the ER. The epiphallar formula is: PP(ER+ELP). The E shows two fringed ELP. They proximally fade before the VD.

##### ﻿ Charpentieria itala allatollae

Taxon classificationAnimaliaStylommatophoraClausiliidae

(Käufel, 1928)

10A494DA-7561-572A-84A9-2876FE3A4393

Figs 44.7, 44.8, 47.6

###### Distribution.

This taxon is known to occur in Val Ampola and Val Lorina (Province of Trento, Trentino-Alto Adige, Italy). It is also reported by Gredler (1891) for Monte Tombea as *Clausiliatombeana* Gredler, 1891 and from other localities in eastern Lombardia by Nardi (2011: 101).

###### Specimens examined.

Italy, Trentino-Alto Adige, Trento, Storo, Albergo alla Tolla, Val Ampola. 720 m asl, 45°51'7.62"N, 10°38'4.51"E, W. De Mattia and J. Macor leg. and det., 2 dissected spm.

###### External morphology of the genital organs

**(Fig. 44.7).** The FO is 1.5 × longer than the V. The FDBC is longer than the BC+SDBC. The BC+SDBC is club-like, with visible distinction between the SDBC and the BC. The apex is rounded. The D is slightly longer than the BC+SDBC, thin and with a round apex. The V is short but wide in diameter. The PC is double as long as the V. The P is swollen along its whole length. The PR is short and robust. The E is longer than the P but thinner in diameter.

###### Internal morphology of the genital organs

**(Fig. 44.8).** The A, the V and the P are smooth, with few, very loose and barely visible longitudinal elevations. The PP is almost smooth, elongated with a pointed apex. The PP originates from the distal end of the ELP. The ER is not present. The epiphallar formula is: PP(ELP). The E shows two ELP that proximally fade before the VD.

###### Remarks.

This taxon, previously considered as a junior synonym of *C.italalorinae* (Nordsieck 1963b: 184) was recently elevated (with no explanation) as a valid subspecies by the same author (Nordsieck 2007: 54). It was long considered as a transitional hybrid form between *C.clavata* and *C.itala* but this assumption was based exclusively upon shell characters (Nardi 2011: 101). Scheel and Hausdorf (2012: 3799) proved this is not correct as the taxa previously belonging to one or the other species appear deeply intermingled in the phylograms they provided.

##### ﻿ Charpentieria itala baldensis

Taxon classificationAnimaliaStylommatophoraClausiliidae

(Strobel, 1851)

82D8ADE1-E77B-5849-B2A9-2CFFF0D3FD03

Figs 45.1–45.3, 47.7

###### Distribution.

*Charpentieriaitalabaldensis* is exclusively known from Monte Baldo and Monte Altissimo (Verona, Veneto, Italy), restricted to the peak areas.

**Figure 44.**
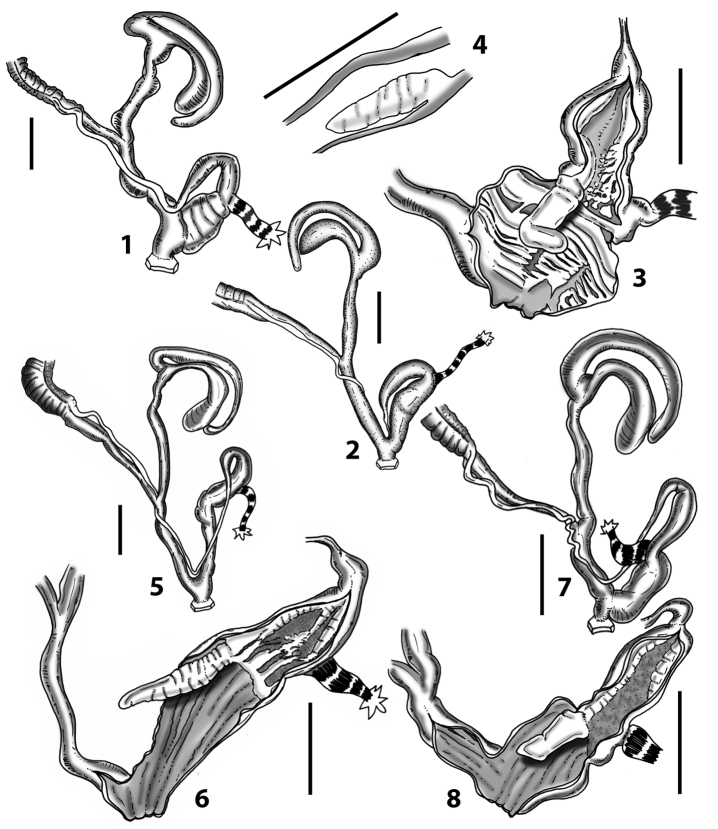
*Charpentieriaitalaitala* (G. von Martens, 1824), Barbarano Vicentino **44.1, 44.2** whole distal genital organs **44.3** internal distal part of genital organs **44.4** detail of the pseudopapilla. *Charpentieriaitalaalbopustolata* (De Cristofori & Jan, 1832), Brescia **44.5** whole distal genital organs **44.6** internal distal part of genital organs. *Charpentieriaitalaallatollae* (Käufel, 1928), Val Ampola, Storo **44.7** whole distal genital organs **44.8** internal distal part of genital organs.

###### Specimens examined.

Italy, Veneto, Verona, Naole, San Zeno di Montagna. 900 m asl, 45°44'25.95"N, 10°51'13.69"E, [Lab ID C2_2, COI: MW758955], I. Niero leg. and det., 2 dissected spm.

###### External morphology of the genital organs

**(Fig. 45.1).** The FO is as long as the V. The FDBC is longer than the BC+SDBC (SDBC+BC). The BC+SDBC presents a very distinguishable second duct as the BC abruptly inflates. The apex is rounded. The D is slightly longer than the BC+SDBC, with a thin proximal part and a swollen distal part. The V is long and thin in diameter. The PC is double as long as the V. The P is slightly swollen at its proximal part. The transition area P-E is clearly detectable from outside. The PR is short and robust. The E is slightly longer than the P but thinner in diameter.

###### Internal morphology of the genital organs

**(Figs 45.2, 45.3).** The A and the V show smooth few, very loose and barely visible longitudinal elevations. The P shows five to six smooth longitudinal plates that proximally directly start from the ER. The PP is smooth, very elongated with a pointed apex and it occupies almost the whole penial internal volume. Its basis is slightly swollen. The PP originates independently, not connecting neither with the ER or the ELP. The epiphallar formula is: ER+PP+ELP. The E shows two remarkably fringed ELP. They proximally fade before the VD.

**Figure 45.**
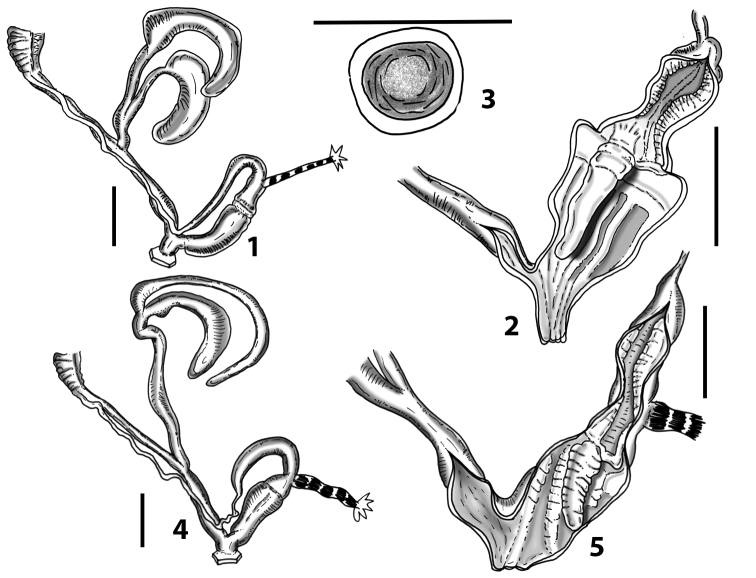
*Charpentieriaitalabaldensis* (Strobel, 1851), San Zeno, Verona **45.1** whole distal genital organs **45.2** internal distal part of genital organs **45.3** cross section of the pseudopapilla. *Charpentieriaitalabalsamoi* (Strobel, 1850), Val Serina, Bracca **45.4** whole distal genital organs **45.5** internal distal part of genital organs.

###### Remarks.

This small subspecies, found exclusively at higher altitudes, was previously considered as a transitional form between *C.italaalbopustolata* and *C.italarubiginea* (Nordsieck 1963b: 176–177). The same author (Nordsieck 2007: 54) recently elevated it (with no taxonomic explanation) as a valid subspecies. The taxon was described by Strobel (1851: 25) and not by Charpentier (1852: 384) as stated in MolluscaBase (2021).

##### ﻿ Charpentieria itala balsamoi

Taxon classificationAnimaliaStylommatophoraClausiliidae

(Strobel, 1850)

CAA2DDEC-40CE-5D85-AA3D-18CEF5C7068A

Figs 45.4, 45.5, 47.8

###### Distribution.

This subspecies is known from Val Serina (province of Bergamo, Lombardia), Italy (Nardi 2011: 101).

###### Specimens examined.

Italy, Lombardia, Bergamo, Val Serina, Loc. Galleria, Bracca. 45°49'24.49"N, 09°43'6.73"E, W. De Mattia and J. Macor leg. and det., 2 dissected spm.

###### External morphology of the genital organs

**(Fig. 45.4).** The FO is 1.5 × longer than the V. The FDBC is extremely short. The BC+SDBC is club-like, with no distinction between the SDBC and the BC. The D is longer than the BC+SDBC. The V is cylindrical. The PC is double as long as the V. The P is slightly swollen at its proximal part with a clearly visible transition between P and EP. The PR is long and robust. The E is slightly longer than the P but thinner in diameter.

###### Internal morphology of the genital organs

**(Fig. 45.5).** The A and the V are almost smooth, with fine and barely visible longitudinal elevations. The P shows 3 oblique fringed pleats that proximally are not connected to the ER. The PP is fringed and occupies almost the whole penial internal volume. The PP originates from the ER that is connected with the ELP. The epiphallar formula is: PP(ER(ELP)). The E shows two remarkably fringed ELP. They proximally fade before the VD.

##### ﻿ Charpentieria itala clavata

Taxon classificationAnimaliaStylommatophoraClausiliidae

(Rossmässler, 1836)

1A4D90C6-D6CA-56E0-B402-D807647AADBF

Figs 46.1–46.3, 47.9

###### Distribution.

This subspecies is known to occur in central-western Lombardia, in the provinces of Bergamo and Lecco (Nordsieck 1963b; Nardi 2011).

###### Specimens examined.

Italy, Lombardia, Lecco, Ballabio. 710 m asl, 45°54'25.96"N, 09°25'55.16"E, W. De Mattia and J. Macor leg. and det., 2 dissected spm.

###### External morphology of the genital organs

**(Fig. 46.1).** The V is slightly longer than the FO. The FDBC is longer than the SDBC+BC. The BC+SDBC is cylindrical, with no clear distinction between the SDBC and the BC. The D is longer than the BC+SDBC. The V is cylindrical and long. The PC is ~ 1.5 × longer than the V. The P is conical and the transition between P and EP is not clearly visible. The PR is short and robust. The E is shorter than the P but shows almost the same diameter.

###### Internal morphology of the genital organs

**(Figs 46.2, 46.3).** The A and the V show irregular smooth folds that continue proximally inside the P. The P, as the V and A, does not present a regular pattern of pleats but a combination of irregular smooth folds. The PP is very big, folded with a wide base. It occupies the whole penial volume and reaches the A. The PP originates from the epiphallar wall and it is connected with the ELP. The epiphallar formula is: PP(ELP). The E shows two moderately fringed ELP. They proximally fade before the VD.

**Figure 46.**
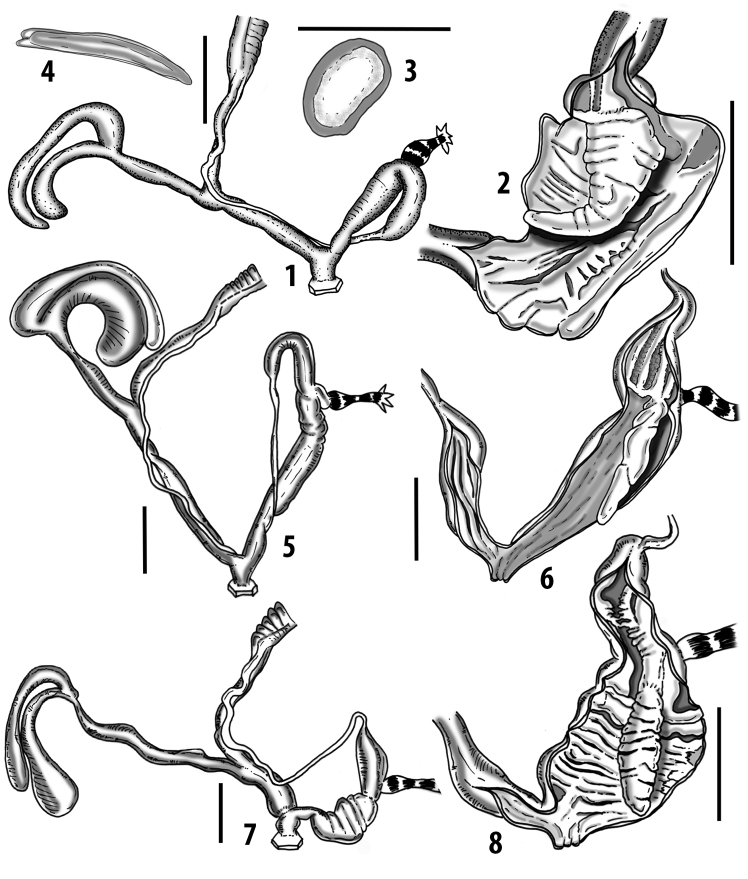
*Charpentieriaitalaclavata* (Rossmässler, 1836), Ballabio, Lecco **46.1** whole distal genital organs **46.2** internal distal part of genital organs **46.3** cross section of the pseudopapilla **46.3** spermatophore. *Charpentieriaitalalatestriata* (Küster, 1850), Bedulita, Bergamo **46.5** whole distal genital organs **46.6** internal distal part of genital organs. *Charpentieriaitalalorinae* (Gredler, 1869), Val Lorina, Storo **46.7** whole distal genital organs **46.8** internal distal part of genital organs.

##### ﻿ Charpentieria itala latestriata

Taxon classificationAnimaliaStylommatophoraClausiliidae

(Küster, 1850)

812CF2C5-F539-5FCA-8714-02DC8181EF55

Figs 46.4, 46.5, 47.10

###### Distribution.

Known to occur in central Lombardia, Italy (Nardi 2011: 101).

###### Specimens examined.

Italy, Lombardia, Bergamo, Bedulita. 625 m asl, 45°47'16.15"N, 09°32'42.26"E, W. De Mattia and J. Macor leg. and det., 2 dissected spm.

###### External morphology of the genital organs

**(Fig. 46.5).** The V is almost 2 × longer than the FO. The FDBC is as long as the SDBC+BC. The BC+SDBC is club-like, with a more or less clear distinction between the SDBC and the BC. The D is longer than the BC+SDBC and slender. The V is cylindrical and long, slightly tapered along its proximal part. The PC is ~ 1.5 × longer than the V. The P is conical and the transition between P and EP is not clearly visible. The PR is short and robust. The E is shorter than the P but shows almost the same diameter. The whole genital organs show a slender appearance [recalling the ectomorph genital organs as in De Mattia et al. (2020)].

###### Internal morphology of the genital organs

**(Fig. 46.6).** The V presents irregular longitudinal pleats that stop just before the A. The A the P are mainly smooth with barely visible longitudinal elevations. The PP is smooth, slender, elongated and slightly irregular in shape. It occupies half of the internal penial volume. The PP originates from the ER that is connected with the ELP by means of four minor smooth longitudinal pleats. The epiphallar formula is: PP(ER+ELP). The E shows two moderately fringed ELP. They proximally fade before the VD.

##### ﻿ Charpentieria itala lorinae

Taxon classificationAnimaliaStylommatophoraClausiliidae

(Gredler, 1869)

57F974DB-7647-5754-8CE4-A8717D514AA3

Figs 46.6, 46.7, 47.11

###### Distribution.

The distribution of this subspecies is limited to Val Lorina and surrounding peaks, across the border of Trento and Brescia provinces (Lombardia and Trentino-Alto Adige), Italy (Nardi 2011:101).

###### Specimens examined.

Italy, Lombardia, Brescia, Val di Lorina, Storo. 600 m asl, 45°50'23.18"N, 10°36'49.06"E, [Lab ID C3_2, COI: MW758954], W. De Mattia and J. Macor leg. and det., 3 dissected spm.

###### External morphology of the genital organs

**(Fig. 46.7).** The V is half as long as the FO. The FDBC is very long, at least 2 × the SDBC+BC. The BC+SDBC is club-like, with a more or less clear distinction between the SDBC and the BC. The D is slightly shorter than the BC+SDBC. The V is cylindrical and very short, wide in diameter. The PC is ~ 2 × longer than the V. The P is short, folded and wide in diameter and the transition between P and EP is clearly visible. The PR is short and robust. The E is almost as long as P and wide in diameter. The genital organs show a slender appearance [recalling the ectomorph genital organs as in De Mattia et al. (2020)].

###### Internal morphology of the genital organs

**(Fig. 46.8).** The A the V are mainly smooth with weak longitudinal elevations. The P presents strong irregular transversal smooth pleats. The PP is folded, irregular and elongated. It occupies almost the whole internal penial volume. The ER is found distally of the PP and not connected with it. The PP is directly connected with the ELP. The epiphallar formula is: ER+PP(ELP). The E shows two irregularly fringed ELP. They proximally fade before the VD.

**Figure 47.**
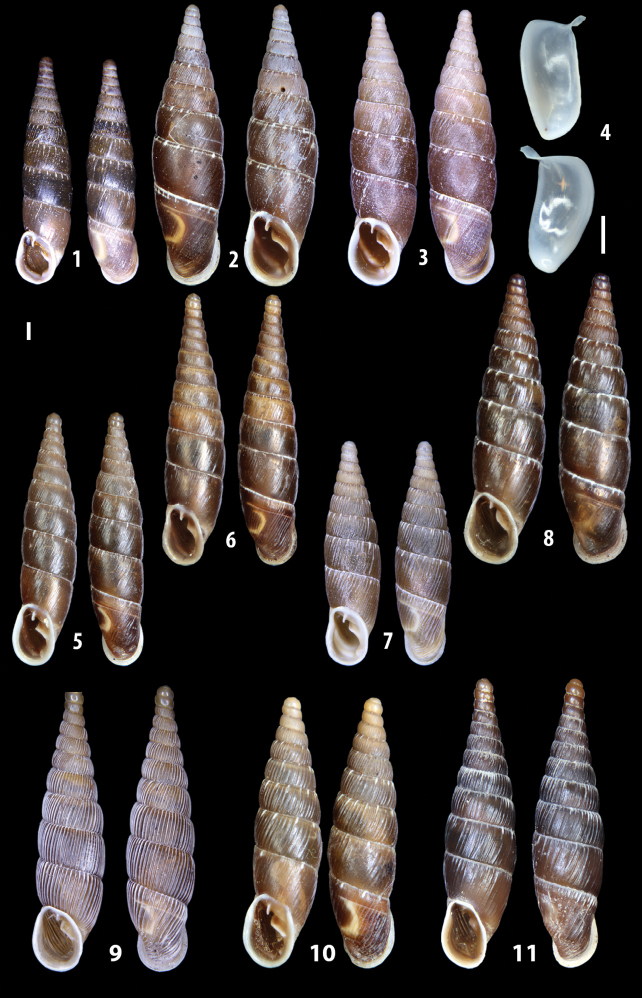
*Charpentieriaornata* (Rossmässler, 1836), Ogulin, HR **47.1** shell. *Charpentieriaitalaitala* (G. von Martens, 1824), Barbarano Vicentino **47.2–47.3** shell **47.4** clausiliar plate double side. *Charpentieriaitalaalbopustolata* (De Cristofori & Jan, 1832), Brescia **47.5** shell. *Charpentieriaitalaallatollae* (Käufel, 1928), Val Ampola, Storo **47.6** shell. *Charpentieriaitalabaldensis* (Strobel, 1851), San Zeno, Verona **47.7** shell. *Charpentieriaitalabalsamoi* (Strobel, 1850), Val Serina, Bracca **47.8** shell. *Charpentieriaitalaclavata* (Rossmässler, 1836), Ballabio, Lecco **47.9** shell. *Charpentieriaitalalatestriata* (Küster, 1850), Bedulita, Bergamo **47.10** shell. *Charpentieriaitalalorinae* (Gredler, 1869), Val Lorina, Storo **47.11** shell.

##### ﻿ Charpentieria itala zalloti nom. nov.

Taxon classificationAnimaliaStylommatophoraClausiliidae

47A137B8-54F7-5944-B6EC-CCB9896B43AB

Figs 48.1–48.2, 50.1–50.2

###### Remarks.

**Figure 48.**
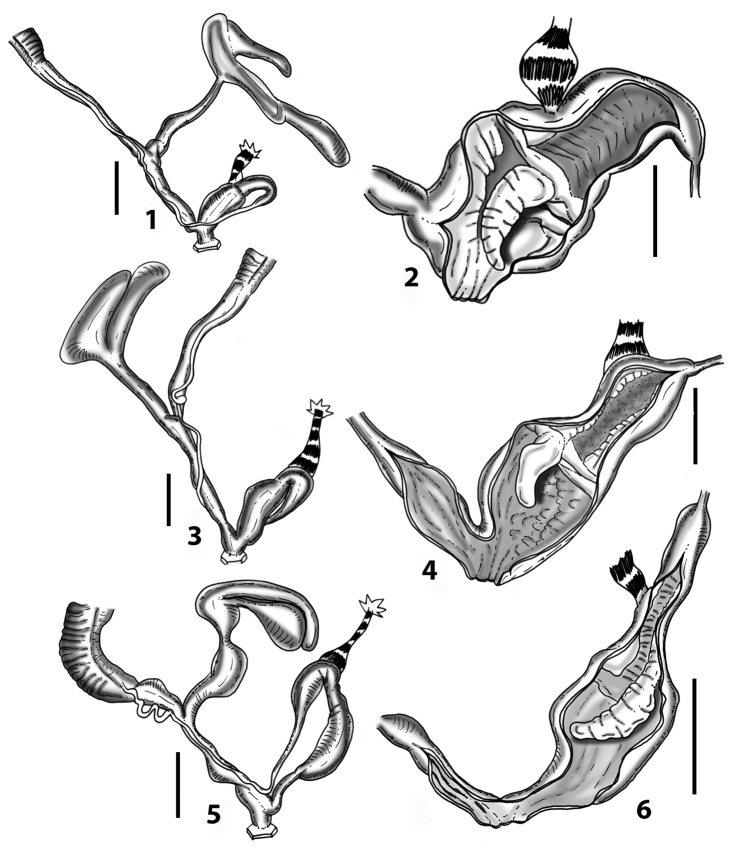
*Charpentieriaitalazalloti* nom. nov., Udine **48.1** whole distal genital organs **48.2** internal distal part of genital organs. *Charpentieriaitalaserravalensis* (Nordsieck, 1963), Serravalle, Vittorio Veneto **48.3** whole distal genital organs **48.4** internal distal part of genital organs. *Charpentieriaitalatrepida* (Käufel, 1928), Cima Ora, Anfo **48.5** whole distal genital organs **48.6** internal distal part of genital organs.

During field collecting (WDM), five populations belonging to *C.itala* ssp. were found in the surrounding of Udine and Cividale (Friuli, Italy). These populations show a remarkably reduced size of the shell comparing to the nominate subspecies or *C.italaserravalensis*, that are the closest *C.itala* subspecies in the west (Figs 44, 48). Moreover, the sutures are almost colourless, the shell is translucent and the peristome is continuous (Figs 50.1, 50.2). Nordsieck (1963b) stated that the eastern distributional limit for *C.itala* is represented by the axis Pordenone-Ampezzo. Thus, these Friulian populations were neglected or simply not known by him. Nevertheless, these populations of *C.itala* were not unknown to past authors. Pirona (1865: 692) cited ”*Clausiliaalboguttata* Wagn. [= *C.itala*]. Nei muri vecchi muscosi (On old mossy walls); Udine, Tricesimo, Dolegna, Sacile, Portogruaro”. He listed two morphotypes: var. a, *major* for the western part (Caneva and Portogruaro) and var. b, *minor* for Udine and Dolegna. In his description, for var. b, *minor*Pirona (1865) pointed out the translucent shell (*cornea*) and the continuous peristome (*peristomate continuo*) and these details, as previously stated, are regularly found also in the easternmost populations recently collected. Concerning the anatomy of the genital organs, these populations present the absence of the (2) longitudinal epiphallar pleats (Figs 48.1–48.2). As regards the identity of the var. a, *major*, following Pirona’s description (*saturate rubiginea*, *peristomate soluto*) it can be postulated that he dealt with the nominate subspecies.

The type locality of the nominate subspecies is less than 50 km west of Portogruaro. Thus, following Pirona (1865) and recent research, the distributional area of *C.itala* is further extended eastward as far as the Friuli plain and Cividale/Dolegna in the east.

Despite Pirona’s (1865) thorough description, *Clausiliaminor* Pirona, 1865 is several times preoccupied by: *Clausiliaminor* Küster, 1850, currently a synonym of *Medoracontractacontracta* (Rossmässler, 1842); *Clausiliaminor* Küster, 1850, currently a synonym of *Medoraalmissanaalmissana* (Küster, 1847); *Clausiliaminor* Charpentier, 1852, currently a synonym of *Albinariaprofuga* (Charpentier, 1852); *Clausiliaminor* A. Schmidt, 1856, currently a synonym of *Clausiliarugosaprovincialis* Coutagne, 1886; *Clausiliaminor* Walderdorff, 1864, currently a synonym of *Delimamontenegrinasemilabiata* (Walderdorff, 1864).

The Friulian populations with translucent shell and reduced shell dimensions are here named *C.italazalloti* nom. nov.

Nordsieck (2011, supplemented May 2021) in chapter 4 clearly referred to our data and manuscript when he felt the need to supplement his web article. He cited Pirona (1865) stating that “The form from Udine, Friuli (…) is strikingly similar to that from Verona. (…) Because of this similarity it is deemed to have been introduced.” We disagree with his rushed conclusion, as the presence of this taxon also in many natural spots along the low Prealps makes an introduction quite unlikely or, at least, deserving additional research.

###### Distribution and habitat.

*Charpentieriaitalazalloti* nom. nov. is found on limestone stonewalls (Cividale) or natural limestone small cliffs (Udine castle hill, Tricesimo and Julian Prealps), usually in shady spots. The taxon is found in the Friuli plain and in natural habitats along the low Prealps. Pirona (1865: 692) provides a wider distribution including territories in the west, as far as Sacile, and in the east as far as Dolegna.

###### Specimens examined.

Italy, Friuli-Venezia Giulia, Udine, castle hill. 140 m asl, 46°03'53.37"N, 13°14'12.35"E, L. Anzil leg., W. De Mattia det., 3 dissected spm. Italy, Friuli-Venezia Giulia, Udine, Tribil Superiore. 600 m asl, 46°08'26.55"N, 13°37'3.22"E, W. De Mattia and J. Macor leg. and det., 2 dissected spm. Italy, Friuli-Venezia Giulia, Udine, Lesizza. 340 m asl, 46°7'55.33"N, 13°33'57.00"E, W. De Mattia and J. Macor leg. and det. Italy, Friuli-Venezia Giulia, Udine, Stermizza. 730 m asl, 46°11'15.09"N, 13°31'31.28"E, W. De Mattia and J. Macor leg. and det. Italy, Friuli-Venezia Giulia, Udine, Rucchin. 670 m asl, 46°9'12.62"N, 13°38'12.23"E, W. De Mattia and J. Macor leg. and det.

###### External morphology of the genital organs

**(Fig. 48.1).** The V is slightly shorter than the FO. The FDBC is as long as the SDBC+BC. The BC+SDBC is club-like, with a more or less clear distinction between the SDBC and the BC. The D is slightly shorter than the BC+SDBC. The V is cylindrical and wide in diameter. The PC is only slightly longer than the V. The P is short but wide in diameter and the transition between P and EP is clearly visible. The PR is short and robust. The E is almost as long as the P but small in diameter.

###### Internal morphology of the genital organs

**(Fig. 48.2).** The V is smooth. The P presents two strong irregular longitudinal folded pleats that reach the A. The PP is folded and elongated with a pointed tip. It occupies almost the whole penial volume. The PP originates from the ER which is connected to the ELP. The two ELP are weak. The epiphallar formula is: PP(ER(ELP)).

###### Etymology.

*Charpentieriaitalazalloti* nom. nov. was named after Enrico Zallot (Feltre, Italy-Monster, The Netherlands), skilled malacologist, as a token of friendship and esteem.

##### ﻿ Charpentieria itala serravalensis

Taxon classificationAnimaliaStylommatophoraClausiliidae

(Nordsieck, 1963)

18C9B3C4-5485-51DF-BB84-2A9F8702907E

Figs 48.3, 48.4, 50.3

###### Distribution.

This taxon is widespread throughout the southeastern Alps, from Val Sugana in the west, Alpi Venete, Alps of Belluno and the axis Ampezzo-Pordenone in the east (Italy) (Nordsieck 1963b: 175).

###### Specimens examined.

Italy, Veneto, Serravalle (L.T.) near Vittorio Veneto. 500 m asl, 46°00'4.46"N, 12°17'11.13"E, W. De Mattia and J. Macor leg. and det., 3 dissected spm.; Italy, Trentino-Alto Adige, Trento, San Romedio. 730 m asl, 46°22'3.83"N, 11°06'31.84"E, W. De Mattia and J. Macor leg. and det.

###### External morphology of the genital organs

**(Fig. 48.3).** The V is as long as the FO. The FDBC is as long as the SDBC+BC. The BC+SDBC is cylindrical, short, with no distinction between the SDBC and the BC. The D is slightly shorter than the SDBC+BC. The V is cylindrical and long, thin in diameter. The PC is approximately as long as the V. The P is swollen and wide in diameter and the transition between P and EP is clearly visible. The PR is short and robust. The E is almost as long as the P but thinner in diameter.

###### Internal morphology of the genital organs

**(Fig. 48.4).** The A and the V are smooth, with of almost undetectable longitudinal elevations. The P presents weak tubercles longitudinally arranged along irregular lines. The PP is smooth and elongated. It is small comparing to the whole penial volume. The PP originates from the ER, which is connected with the ELP. The epiphallar formula is: PP(ER(ELP)). The E shows two fringed ELP. They proximally fade before the VD.

###### Remarks.

The actual status and validity of *C.italaserravalensis*, especially in relation to the nominate subspecies, must be further assessed with more field research and morphological/genetic molecular studies.

##### ﻿ Charpentieria itala trepida

Taxon classificationAnimaliaStylommatophoraClausiliidae

(Käufel, 1928)

D857BFBD-1998-5976-9A0E-2BEC88838A72

Figs 48.5, 48.6, 50.4

###### Distribution:

The taxon is known for several stations around the Idro Lake, at the boundary between the provinces of Brescia and Trento (Nardi 2011: 98).

###### Examined material:

Forte Cima Ora, Anfo, Brescia, Lombardia, Italy. 1500 m asl, 45°47'50.57"N, 10°28'3.60"E, W. De Mattia and J. Macor leg. and det., 2 dissected spm.

###### External morphology of the genital organs (Fig. 48.5):

The V is 3 × longer than the FO. The FDBC is 2 × longer than the SDBC+BC. The BC+SDBC is markedly club-like, short, with clear distinction between the SDBC and the BC. The D is slightly longer than the SDBC+BC and thinner in diameter. The V is cylindrical and long, slightly swollen in its mid part. The PC is ~ 2 × as long as the V. The P is distally pedunculate and abruptly swollen and widens in diameter along its proximal part. The transition between P and EP is not clearly visible. The PR is long and robust. The E is much shorter than the P but wide in diameter.

###### Internal morphology of the genital organs (Fig. 48.6):

The A is smooth. The V presents smooth longitudinal pleats. The P is completely smooth. The PP is folded and elongated. It occupies half of the internal penial volume. The ER is missing. The PP is directly connected with the ELP. The epiphallar formula is: PP(ELP). The E shows two moderately fringed ELP. They proximally fade before the VD.

###### Remarks:

In the surroundings of Forte Cima Ora (Anfo) we collected this costate form that was wrongly identified as *Charpentieriaitalatiesenhauseni* (Gredler, 1855) by Nardi (2011: 98, Fig. 6). *Charpentieriaitalatiesenhauseni* seems to be restricted to the type locality (Bocca di Valle) and few additional localities E of the Lago d’Idro (Nordsieck pers. comm.). The population from Forte Cima Ora is here considered as a costate form of *C.italatrepida* (see Nordsieck 2007: 54).

##### ﻿ Charpentieria itala triumplinae

Taxon classificationAnimaliaStylommatophoraClausiliidae

Nardi, 2011

96BDD215-87DE-5ED4-B115-41B07AACC572

Figs 48.5–48.6, 49.1–49.2, 50.4, 50.5

###### Distribution.

This subspecific taxon of *C.itala* is endemic to the Central Alps. It is known from the type locality and few scattered localities in the Val Trompia area: Corna Blacca, Passo delle Portole, Cima Caldoline, Passo della Berga and other localities in the municipalities of Lavenone and Collio (Brescia, Lombardia, Italy), from 1500 m asl up (Nardi 2011: 99–100).

###### Specimens examined.

Italy, Lombardia, Brescia, Lavenone, Cima Caldoline. 1700 m asl, 45°48'3.54"N, 10°24'20.12"E, G. Nardi leg. and det., 2 dissected spm.

###### External morphology of the genital organs

**(Fig. 49.1).** The V is half as long as the FO. The FDBC is 2 × longer than the SDBC+BC. The BC+SDBC is club-like, short, with no clear distinction between the SDBC and the BC. The D is slightly longer than the SDBC+BC. The V is short and cylindrical. The PC is ~ 2 × as long as the V. The P is swollen. The transition between P and EP is clearly visible. The PR is short and robust. The E is much shorter than the P but wide in diameter.

###### Internal morphology of the genital organs

**(Fig. 49.2).** The A is smooth. The V presents irregular pleats. The P is completely smooth. The PP is folded and elongated. It occupies ^3^/_4_ of the internal penial volume. The PP originates from the ER that is directly connected with the ELP. The epiphallar formula is: PP(ER(ELP)). The E shows two moderately fringed ELP. They proximally fade before the VD.

###### Remarks.

Nardi (2011: 99) roughly described the anatomy of the genital organs providing a sketch of a specimen from the same locality we had available as well. His description and depiction differ from our anatomical results by a remarkably shorter FO and a longer V, whereas the remaining genital external details seem to be similar (e.g., the penial complex). Nardi (2011: 99) did not provide any detail of the internal sculpturing of the genital organs, depicting only the pseudopapilla (named ”papilla penis“). Moreover, the pseudopapilla appears slightly different according to our results, being it smooth instead of folded. However, its general conical shape and dimensions (in comparison with the volume of the penis) match with our results. The folding of its surface could be due to a reproductive/seasonal/physiological moment of the specimen at the time of collecting/fixation.

##### ﻿ Charpentieria itala variscoi

Taxon classificationAnimaliaStylommatophoraClausiliidae

(Pini, 1883)

AECD2866-6FB5-5CDB-88D7-0397FDB30F41

Figs 49.3, 49.4, 50.6

###### Distribution.

This taxon is known only from the Val Brembana and Val Taleggio (Bergamo, Lombardia, Italy) (Nordsieck 1963b).

###### Specimens examined.

Italy, Lombardia, Bergamo, Lavina, Val Taleggio. 650 m asl, 45°53'11.73"N, 09°33'55.46"E, W. De Mattia and J. Macor leg. and det., 2 dissected spm.

###### External morphology of the genital organs

**(Fig. 49.3).** The V is slightly shorter than the FO. The FDBC is as long as the SDBC+BC. The BC+SDBC is club-like in shape with clear distinction between the SDBC and the BC. The D is longer than the SDBC+BC. The V is short and slightly swollen. The PC is ~ 3 × as long as the V. The P is fusiform. The transition between P and EP is not clearly visible. The PR is long and thin. The E is slightly shorter than the P and thinner in diameter.

###### Internal morphology of the genital organs

**(Fig. 49.4).** The A and the V are smooth. The P is distally smooth, with transversal irregular weak pleats along its proximal part. The PP is folded and very elongated. It occupies the whole penial volume. The PP originates from the ER that is connected with the ELP by means of few minor longitudinal pleats. The epiphallar formula is: PP(ER+ELP). The E shows two moderately fringed ELP. They proximally fade before the VD.

**Figure 49.**
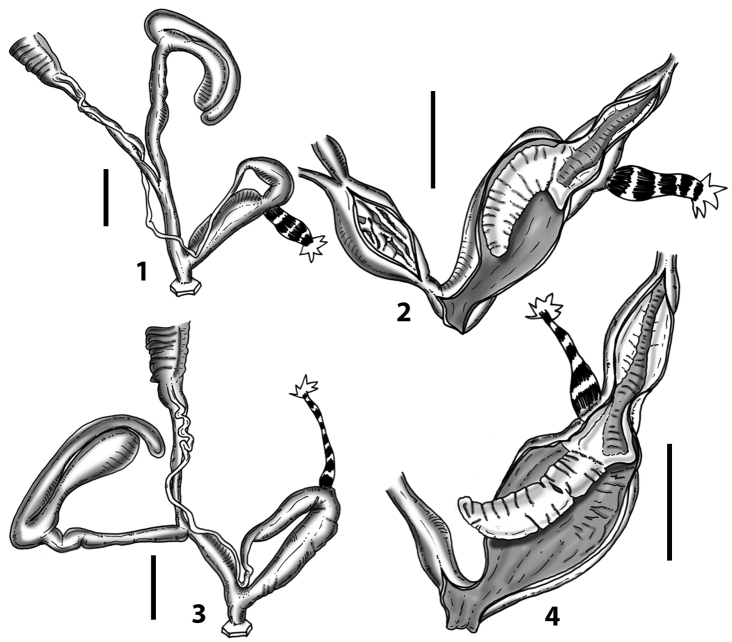
*Charpentieriaitalatriumplinae* Nardi, 2011, Lavenone, Brescia **49.1** whole distal genital organs **49.2** internal distal part of genital organs. *Charpentieriaitalavariscoi* (Pini, 1883), Val Taleggio, Bergamo **49.3** whole distal genital organs **49.4** internal distal part of genital organs.

####### ﻿Brief genital anatomical description of *Charpentieriastenzii* subspecific taxa

*Charpentieriastenzii* is present along the Eastern Alps, from the Val di Non in Trentino and Pre-Alps of Veneto in the West to the Alpi Giulie in the East (Zallot 2001; Welter-Schultes 2012). Currently nine subspecific taxa are considered valid (Zallot 2001: 137–138; MolluscaBase 2021). *Charpentieriastenzii* shows a remarkable variability as regards the shell features. This is well depicted and described in Zallot (2001: 143–155). As for *C.itala* the subspecies were traditionally based on shell morphological characters, e.g., the sculpturing of the shell surface (smooth, striated to ribbed) or the development and variability of the internal plicae and lamellae. The shell is almost smooth or weakly striated to striated or frankly ribbed (as in *C.stenziiletochana*). The suture is always remarkably whitish. Compared to *C.dyodon*, *C.itala* and *C.ornata*, the species, presents a reduction of the buccal armature, with both, columellaris and parietalis generally small to almost absent. The lunella is usually weak to almost undetectable (Zallot 2001).

**Figure 50.**
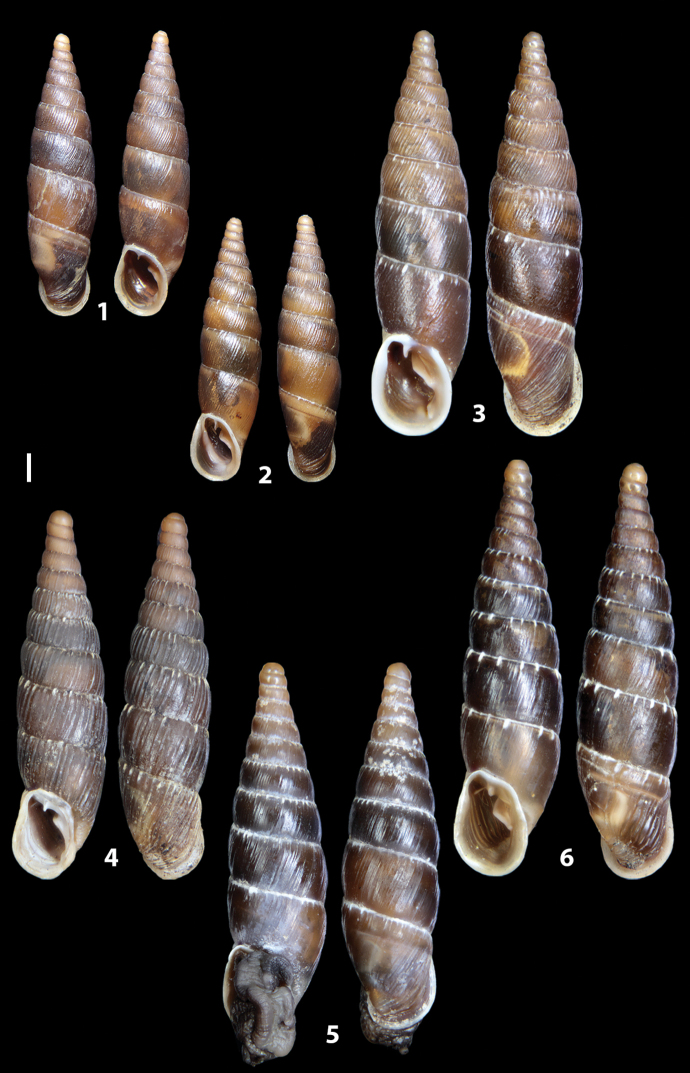
*Charpentieriaitalazalloti* nom. nov., Udine **50.1–50.2** shell. *Charpentieriaitalaserravalensis* (Nordsieck, 1963), Serravalle, Vittorio Veneto **50.3** shell. *Charpentieriaitalatrepida* (Käufel, 1928), Cima Ora, Anfo **50.4** shell. *Charpentieriaitalatriumplinae* Nardi, 2011, Lavenone, Brescia **50.5** shell. *Charpentieriaitalavariscoi* (Pini, 1883), Val Taleggio, Bergamo **50.6** shell.

**Figure 51.**
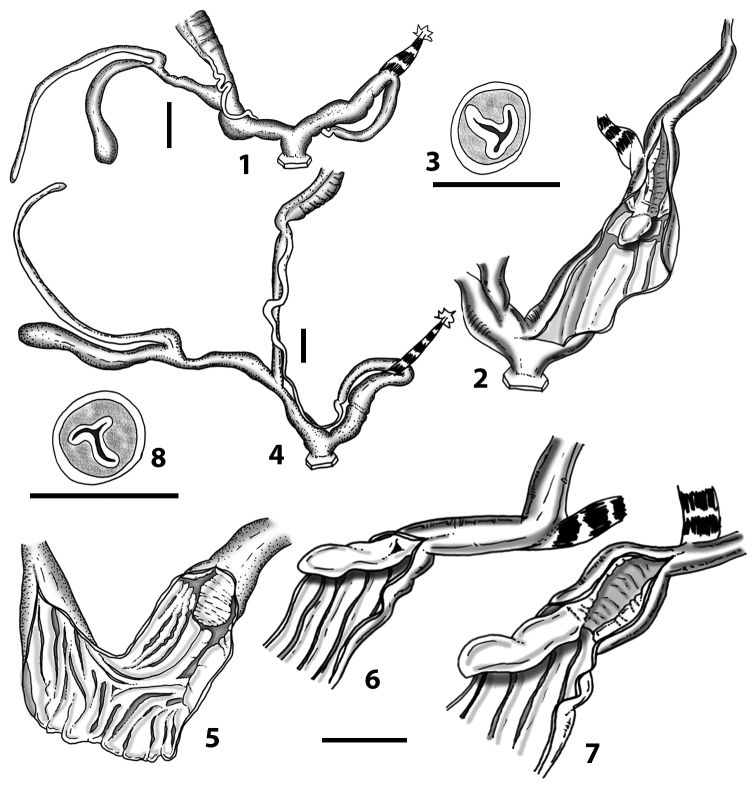
*Charpentieriastenziistenzii* (Rossmässler, 1836), San Romedio, Trento **51.1** whole distal genital organs **51.2** internal distal part of genital organs **51.3** cross section of the epiphallus. *Charpentieriastenziibutoti* (Bank, 1987), Fai della Paganella **51.4** whole distal genital organs **51.5.** internal distal part of genital organs **51.6** detail of the hemipapilla: uncut **51.7** detail of the hemipapilla: cut funnel **51.8** cross section of the epiphallus.

**Figure 52.**
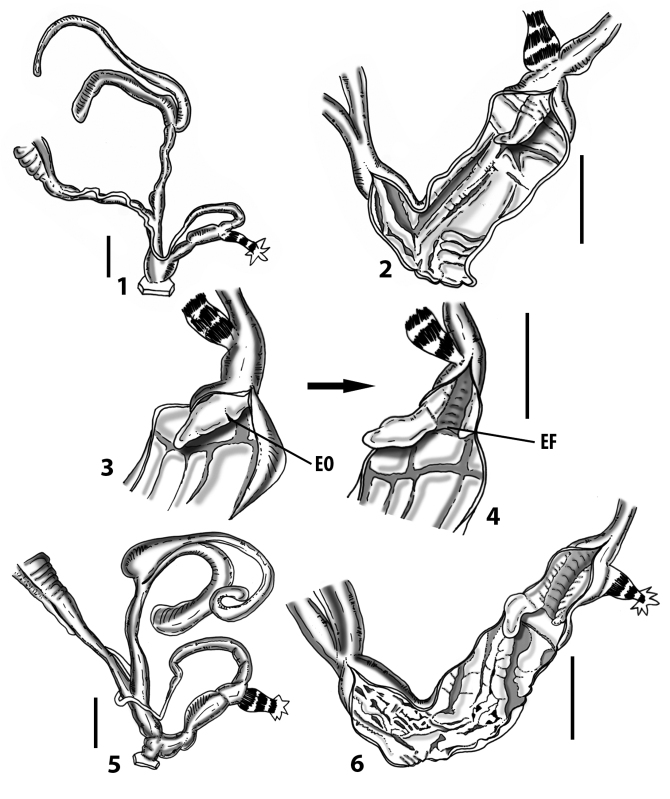
*Charpentieriastenziicincta* (Brumati, 1842), Bosplans, Pordenone **52.1** whole distal genital organs **52.2** internal distal part of genital organs **52.3** detail of the hemipapilla: uncut **52.4** detail of the hemipapilla: cut funnel. *Charpentieriastenziicincta* (Brumati, 1842), Val Resia, Udine **52.5** whole distal genital organs **52.6** internal distal part of genital organs with cut funnel.

**Figure 53.**
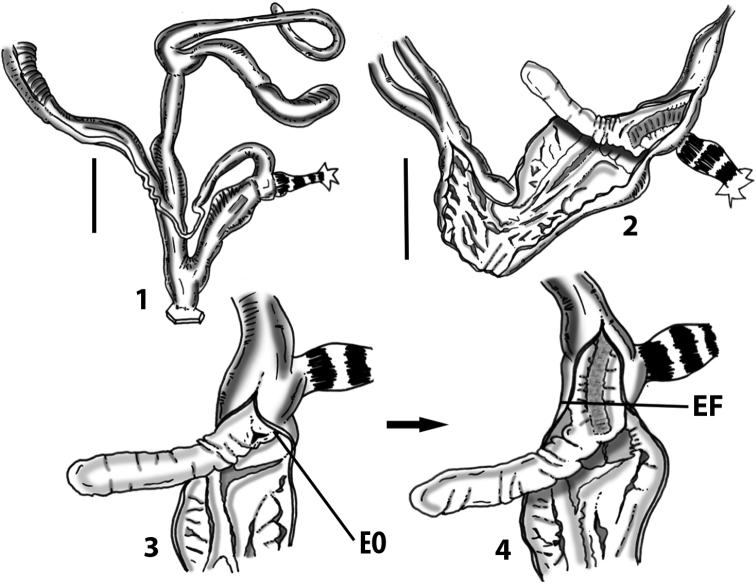
*Charpentieriastenziifaueri* (Bank, 1987), Oliero, Vicenza **53.1** whole distal genital organs. **53.2** internal distal part of genital organs with cut funnel **53.3** detail of the hemipapilla: uncut **53.4** detail of the hemipapilla: cut funnel.

**Figure 54.**
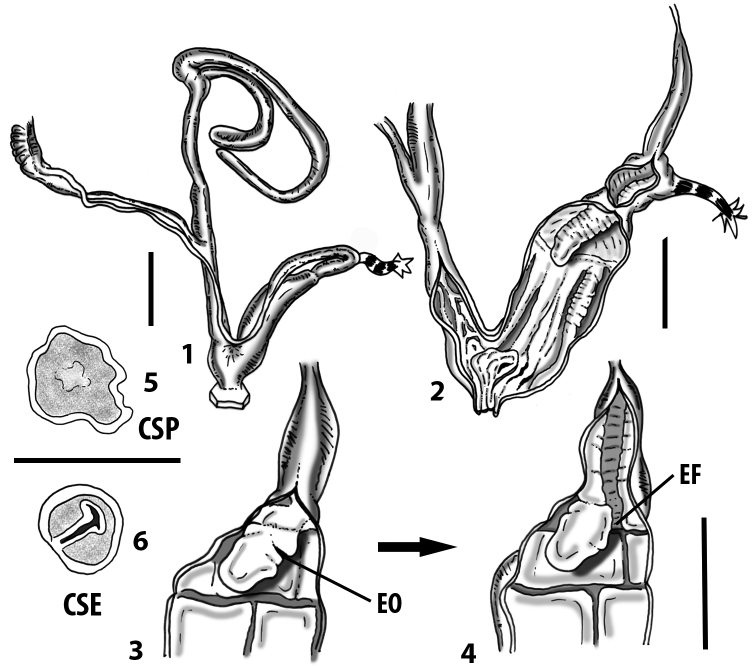
*Charpentieriastenziiletochana* (Gredler, 1874), Val Fonda, Belluno **54.1** whole distal genital organs **54.2** internal distal part of genital organs **54.3** detail of the hemipapilla: uncut **54.4** detail of the hemipapilla: cut funnel **54.5** cross section of the pseudopapilla **54.6** cross section of the epiphallus.

Eight of nine subspecies of *C.stenzii* were anatomically investigated (Table 8). A new anatomical feature, namely the hemipapilla, was discovered and revealed to be typical and exclusive of this species. The hemipapilla consists of an uninterrupted, circular and fleshy elevated ring that almost entirely obliterates the lumen along the penis and epiphallus transition area, slightly distally to the PR. The base of the pseudopapilla is fused with almost the entire ring, making it difficult to distinguish it from a real penial papilla. The sperm channel is anyway present, it is not just a narrow section as in the epiphallar ring of *Sicania/Siciliaria* or the rooted pseudopapilla. Its outlet is found at the base of the pseudopapilla (Figs 51–54). The pseudopapilla is channel-less and filled-up of spongy tissue. The inner atrial pad depicted and deemed by Nordsieck (1963b) as a characteristic of the species, in our genital anatomical investigations was found only in *C.stenziiletochana.* The other subspecies of *C.stenzii* showed a variable inner sculpturing of the atrium, from irregular smooth pleats (as *C.stenziifaueri*) to almost smooth (as *C.stenziiwesterlundi*) with different degrees of complexity in between.

##### ﻿ Charpentieria stenzii stenzii

Taxon classificationAnimaliaStylommatophoraClausiliidae

(Rossmässler, 1836)

48396783-0779-51D2-8CAE-D9E3C84A39DA

Figs 51.1–51.3, 56.1

###### Distribution.

Italy, Trentino-Alto Adige, including westernmost Dolomites in the surroundings of Schlern mountain, Val di Non and Passo della Mendola (Nordsieck 1963b: 191; Zallot 2001: 154).

###### Specimens examined.

Italy, Trentino-Alto Adige, Trento, San Romedio. 730 m asl, 46°22'3.83"N, 11°06'31.84"E, W. De Mattia and J. Macor leg. and det., 2 dissected spm.

###### External morphology of the genital organs

**(Fig. 51.1).** The V is slightly longer than the FO. The FDBC is as long as the SDBC+BC. The BC+SDBC is cylindrical in shape without clear distinction between the SDBC and the BC. The D is longer than the SDBC+BC and thinner. The V is short and wide in diameter. The PC is ~ 3 × longer than the V. The P is cylindrical. The transition between P and EP is not clearly visible. The PR is short and thin. The E is slightly shorter than the P and thinner in diameter.

###### Internal morphology of the genital organs

**(Figs 51.2–51.3).** The A and the V are smooth or with weak irregular convolute pleats. The P presents irregular and wide longitudinal pleats. The hemipapilla is smooth and very short. The outlet of the hemipapilla is roundish. The E shows two moderately fringed ELP. They proximally fade before the VD.

##### ﻿ Charpentieria stenzii butoti

Taxon classificationAnimaliaStylommatophoraClausiliidae

(Bank, 1987)

930BE5A0-9C8C-56C3-94FD-0D10617A057F

Figs 51.4–51.8, 56.2

###### Specimens examined.

Italy, Trentino-Alto Adige, Trento, Fai della Paganella. 600 m asl, 46°12'57.04"N, 11°04'22.88"E, W. De Mattia and J. Macor leg. and det., 3 dissected spm.

###### Distribution.

Massif of Mount Paganella, Trentino-Alto Adige, Italy (Zallot 2001: 154).

###### External morphology of the genital organs

**(Fig. 51.4).** The V is shorter than the FO. The FDBC is slightly shorter than the SDBC+BC. The BC+SDBC is cylindrical in shape without clear distinction between the SDBC and the BC. The D is much longer than the SDBC+BC and thinner. The V is short and wider in diameter along its distal part. The PC is ~ 3 × longer than the V. The P is cylindrical. The transition between P and EP is clearly visible. The PR is long and thin. The E is longer than the P but thinner in diameter along its proximal part.

###### Internal morphology of the genital organs

**(Figs 51.5–51.8).** The A, the V and the P show many more or less irregular, mostly smooth longitudinal pleats. The hemipapilla is smooth and short. The outlet of the hemipapilla is triangular. The E shows two fringed ELP. They proximally fade before the VD.

##### ﻿ Charpentieria stenzii cincta

Taxon classificationAnimaliaStylommatophoraClausiliidae

(Brumati, 1838)

FD29D2C4-399A-5F9F-A452-4BB30DB9E0D7

Figs 52.1–52.6, 56.3

###### Specimens examined.

Italy, Friuli-Venezia Giulia, Pordenone, Bosplans. 500 m asl, 46°11'40.51"N, 12°38'14.26"E, [Lab ID C2_2, COI: MW758953, ITS2: MW757049, MW757050], W. De Mattia and J. Macor leg. and det., 2 dissected spm; Italy, Friuli-Venezia Giulia, Tarvisio, Val Resia, sella di Carnizza. 400 m asl, 46°20'15.55"N, 13°19'17.11"E, W. De Mattia and J. Macor leg. and det., 2 dissected spm.

###### Distribution.

*Charpentieriastenziicincta* presents a wide distribution, approximately including in Italy the Alpi and Prealpi Carniche and Giulie, Dolomiti, Cansiglio and Monti Lessini (Zallot 2001: 154), Stubai Alps (Tribulaun) and farther to the East: Alps of Carinthia and westernmost Slovenia.

###### External morphology of the genital organs (Fig. 52.1).

The V is much shorter than the FO. The FDBC is slightly shorter than the SDBC+BC. The BC+SDBC is cylindrical to club-like in shape, without clear distinction between the SDBC and the BC. The D is much longer than the SDBC+BC and thinner. The V is very short and wide in diameter. The PC is ~ 3 × longer than the V. The P is cylindrical and swollen along its proximal part. The transition between P and EP is clearly visible. The PR is short and thin. The E is longer than the P but thinner in diameter.

###### Internal morphology of the genital organs (Figs 52.2–52.6).

The A, the V and the P show many more or less irregular (longitudinal to oblique) smooth pleats. The hemipapilla is smooth to moderately folded and usually short. The outlet of the hemipapilla is slit-like. The E shows two fringed ELP. They proximally fade before the VD.

##### ﻿ Charpentieria stenzii faueri

Taxon classificationAnimaliaStylommatophoraClausiliidae

(Bank, 1987)

BC460A52-6BD7-58D9-82E0-128EAA207B35

Figs 53.1–53.4, 56.4

###### Specimens examined.

Italy, Veneto, Vicenza, Grotte di Oliero near Valstagna. 800 m asl, 45°50'47.79"N, 11°40'4.24"E, W. De Mattia and J. Macor leg. and det., 3 dissected spm.

###### Distribution.

*Charpentieriastenziifaueri* is exclusively known from Col del Vento and Grigno (Trento, Trentino-Alto Adige) and Oliero (Vicenza, Veneto), Italy (Bank 1987: 139).

###### External morphology of the genital organs

**(Fig. 53.1).** The V is much shorter than the FO. The FDBC is as long as the SDBC+BC. The BC+SDBC is cylindrical to club-like in shape, without clear distinction between the SDBC and the BC. The D is much longer than the SDBC+BC and thinner. The V is very short and wide in diameter. The PC is ~ 3 × longer than the V. The P is cylindrical and swollen along its proximal part. The transition between P and EP is clearly visible. The PR is short and robust. The E is as long as the P but much thinner in diameter.

###### Internal morphology of the genital organs

**(Figs 53.2–53.4).** The A, the V and the P show many more or less irregular smooth pleats. The hemipapilla is long, smooth to moderately folded. The outlet of the hemipapilla is triangular. The E shows two fringed ELP. They proximally fade before the VD.

##### ﻿ Charpentieria stenzii letochana

Taxon classificationAnimaliaStylommatophoraClausiliidae

(Gredler, 1874)

BA389149-8081-5E60-8676-622E40244051

Figs 54.1–54.6, 56.5

###### Specimens examined.

Italy, Veneto, Belluno, Misurina, Val Fonda. 1500 m asl, 46°36'27.42"N, 12°12'20.01"E, [Lab ID C19_1, COI: MW758957], W. De Mattia and J. Macor leg. and det., 2 dissected spm.

###### Distribution.

*Charpentieriastenziiletochana* is exclusively known from Val Fonda (Belluno, Veneto) and Val Pusteria (Schönleitental) (Bolzano, Trentino-Alto Adige), Italy (Nordsieck 1963b: 198).

###### External morphology of the genital organs

**(Fig. 54.1).** The V is shorter than the FO. The FDBC is as long as the SDBC+BC. The BC+SDBC is slim, club-like in shape, with a clear distinction between the SDBC and the BC. The D is much longer than the SDBC+BC and thinner. The V is short and thin in diameter. The PC is ~ 2 × longer than the V. The P is cylindrical and slightly swollen along its proximal part. The transition between P and EP is clearly visible. The PR is long and robust. The E is as long as the P but much thinner in diameter.

###### Internal morphology of the genital organs

**(Figs 54.2–54.6).** The V presents irregular smooth pleats. The A has a fleshy, mushroom-like fold. The P shows many irregular smooth to fringed pleats. The hemipapilla is short, smooth to moderately folded. The outlet of the hemipapilla is slit-like. The E shows two moderately fringed ELP. They proximally fade before the VD.

##### ﻿ Charpentieria stenzii nordsiecki

Taxon classificationAnimaliaStylommatophoraClausiliidae

(Fauer, 1991)

EA2232B1-0AFD-56D3-BE62-034D34B7CA1D

Figs 55.1, 55.2, 56.6

###### Specimens examined.

Italy, Veneto, Treviso, Cison di Val Marino. 250 m asl, 45°58'5.53"N, 12°08'52.60"E, W. De Mattia and J. Macor leg. and det., 4 dissected spm.

###### Distribution.

*Charpentieriastenziinordsiecki* is exclusively known from the pre-alpine area of Cison di Valmarino, Valmareno and Passo San Boldo (Treviso, Veneto), Italy (Fauer 1991: 164).

###### External morphology of the genital organs

**(Fig. 55.1).** The V is shorter than the FO. The FDBC is longer than the SDBC+BC. The BC+SDBC is slim, fusiform in shape, without a clear distinction between the SDBC and the BC. The D is longer than the SDBC+BC and thinner. The V is moderately long and thin in diameter. The PC is ~ 3.5 × longer than the V. The P is slim and cylindrical. The transition between P and EP is clearly visible. The PR is long and robust. The E is longer than the P and slightly thinner in diameter. The genital organs show an extremely slender appearance [recalling the ectomorph genital organs as described in De Mattia et al. (2020)].

###### Internal morphology of the genital organs

**(Figs 55.1–55.2).** The V and the A are smooth. The P shows 3 to 4 smooth pleats. The pleats proximally fade toward the transition with the E. The hemipapilla is missing. The E shows two moderately fringed ELP. They proximally fade before the VD.

###### Remarks.

*Charpentieriastenziinordsiecki* lacks the pseudopapilla and presents an extremely ectomorphic genital organs type. This morphotype of genital organs was already found in the genus *Montenegrina* [e.g., *Montenegrina lillae* Fehér & Szekeres, 2016 and *Montenegrina skipetarica* (So**ós**, 1924)] (De Mattia et al. 2020). It seems that there is not direct correlation between ectomorphic state of genital organs and lack of the papilla/pseudopapilla as other *Montenegrina* taxa [e.g., *Montenegrina janinensis* (Mousson, 1859) or *Montenegrina okolensis okolensis* Szekeres, 2006] or also in *Stigmatica* [e.g., *Stigmaticasplendens* (Nordsieck, 1996), see below)] that despite showing the lack of the papilla/pseudopapilla, present a mesomorphic state of genital organs (De Mattia et al. 2020). The complete absence of the papilla/pseudopapilla could probably represent an apomorphic character state due to isolation and lack of gene flow among populations.

##### ﻿ Charpentieria stenzii paroliniana

Taxon classificationAnimaliaStylommatophoraClausiliidae

(De Betta & Martinati, 1855)

90ADD6F6-937B-5F3D-A5DB-920F93FF64B8

Figs 55.3–55.5, 56.7

###### Distribution.

*Charpentieriastenziiparoliniana* is known from the lower Valle del Brenta from Carpanè, Cismon del Grappa to Primolano, and surrounding mountains on the right bank of the Brenta River (Foza, Vicenza, Veneto), Italy (Nordsieck 1963b: 196).

**Figure 55.**
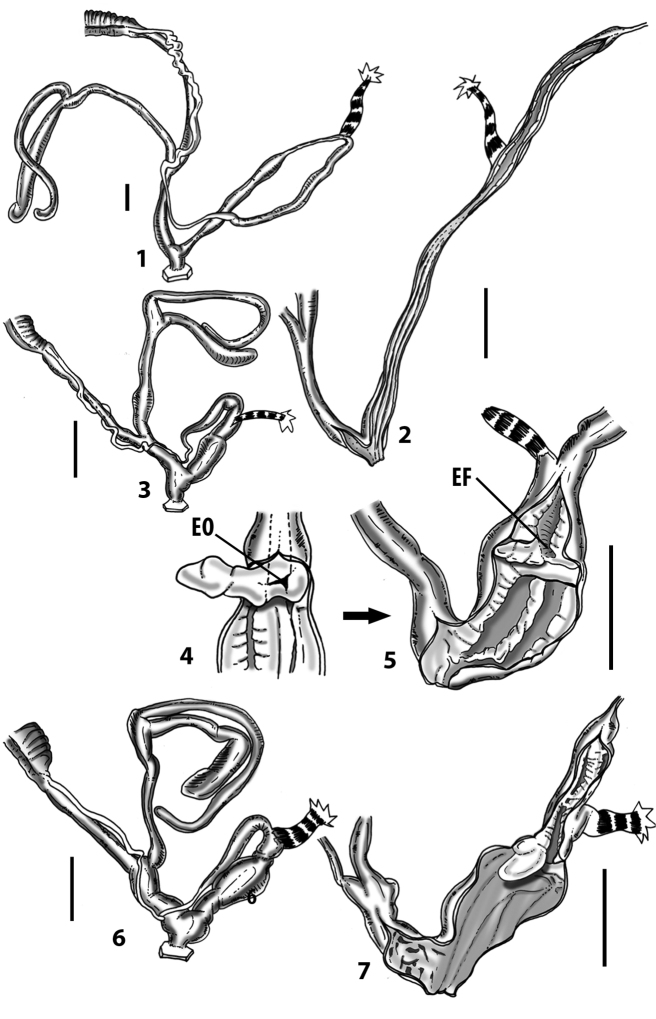
*Charpentieriastenziinordsiecki* Fauer, 1991, Cison di Valmarino, Treviso **55.1** whole distal genital organs **55.2** internal distal part of genital organs. *Charpentieriastenziiparoliniana* (De Betta & Martinati, 1855), Valstagna, Vicenza **55.3** whole distal genital organs **55.4** detail of the hemipapilla: uncut **55.5** internal distal part of genital organs with cut funnel. *Charpentieriastenziiwesterlundi* Nordsieck, 1993, Val Fiscalina, Bolzano **55.6** whole distal genital organs **55.7** internal distal part of genital organs with cut funnel.

**Figure 56.**
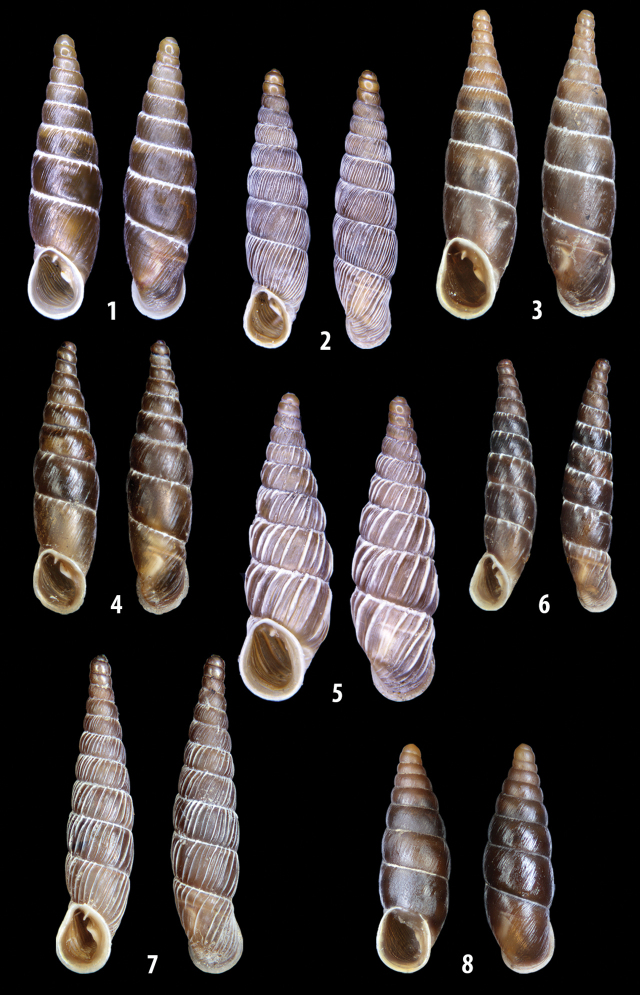
*Charpentieriastenziistenzii* (Rossmässler, 1836), San Romedio, Trento **56.1** shell. *Charpentieriastenziibutoti* (Bank, 1987), Fai della Paganella **56.2** shell. *Charpentieriastenziicincta* (Brumati, 1842), Bosplans, Pordenone **56.3** shell. *Charpentieriastenziifaueri* (Bank, 1987), Oliero, Vicenza **56.4** shell. *Charpentieriastenziiletochana* (Gredler, 1874), Val Fonda, Belluno **56.5** shell. *Charpentieriastenziinordsiecki* Fauer, 1991, Cison di Valmarino, Treviso **56.6** shell. *Charpentieriastenziiparoliniana* (De Betta & Martinati, 1855), Valstagna, Vicenza **56.7** shell. *Charpentieriastenziiwesterlundi* Nordsieck, 1993, Val Fiscalina, Bolzano **56.8** shell.

###### Specimens examined.

Italy, Veneto, Vicenza, Valstagna, Foza, Loc. La Goccia. 800 m asl, 45°52'29.20"N, 11°38'59.73"E, W. De Mattia and J. Macor leg. and det., 3 dissected spm.

###### External morphology of the genital organs

**(Fig. 55.3).** The V is much shorter than the FO. The FDBC is as long as the SDBC+BC. The BC+SDBC is club-like in shape, with a more or less clear distinction between the SDBC and the BC. The D is much longer than the SDBC+BC and slightly thinner. The V is short but wide in diameter. The PC is ~ 2.5 × longer than the V. The P is cylindrical and slightly swollen. The transition between P and EP is clearly visible. The PR is long and robust. The E is longer than the P but thinner in diameter.

###### Internal morphology of the genital organs

**(Figs 55.4–55.5).** The V is smooth. The P shows three to four irregularly fringed pleats that continue as far as the A. The hemipapilla is very short, conical and moderately folded. The outlet of the hemipapilla is triangular. The E shows two fringed ELP. They proximally fade out before the VD.

##### ﻿ Charpentieria stenzii westerlundi

Taxon classificationAnimaliaStylommatophoraClausiliidae

Nordsieck, 1993

3D84B5D0-84DC-56FE-A1D6-E693539275BB

Figs 55.6, 55.7, 56.8

###### Distribution.

*Charpentieriastenziiwesterlundi* is known only from Val Fiscalina (Fischleintal) in the surrounding of Sesto (Bolzano, Trentino-Alto Adige), Italy (Nordsieck 1963b: 196).

###### Specimens examined.

Italy, Trentino-Alto Adige, Bolzano, Sesto, Val Fiscalina (Fischleintal), Croda Rossa. 1500 m asl, 46°38'2.83"N, 12°021'42.79"E, I. Niero leg. and det., 2 dissected spm.

###### External morphology of the genital organs

**(Fig. 55.6).** The V is much shorter than the FO. The FDBC is as long as the SDBC+BC. The BC+SDBC is club-like in shape, with a more or less clear distinction between the SDBC and the BC. The D is much longer than the SDBC+BC and slightly thinner. The V is short but wide in diameter. The PC is ~ 2.5 × longer than the V. The P is cylindrical and slightly swollen. The transition between P and EP is clearly visible. The PR is long and robust. The E is longer than the P but thinner in diameter.

###### Internal morphology of the genital organs (Fig. 55.7).

The V is smooth. The P shows three to four irregularly fringed pleats that continue as far as the A. The hemipapilla is very short, conical and moderately folded. The outlet of the hemipapilla is triangular. The E shows two fringed ELP. They proximally fade before the VD.

###### Remarks.

The original name of this “Form vom Fischleintal in Südtirol” (Nordsieck 1993: 36) namely Clausiliacinctavar.disjuncta Westerlund, 1878, is pre-occupied by *Clausiliadisjuncta* Mortillet, 1854 [= *Armenicadisjuncta* (Mortillet, 1854)]. The new name *Charpentieriastenziiwesterlundi* nom. nov. was provided by Nordsieck (1993: 36).

####### ﻿Brief genital anatomical description of three *Gibbulariagibbula* subspecific taxa

*Gibbularia* is a monotypic genus, currently considered both as a subgenus of *Siciliaria* (Nordsieck 2007; Nordsieck 2013a) or a subgenus of *Charpentieria* (MolluscaBase 2021).

MolluscaBase eds. (2021) citation is based on Nordsieck (2002: 29) and the information provided by Nordsieck’s papers of 2007 and 2013a have never been subsequently adopted. The (sub)genus was briefly reviewed by Nordsieck (2013a: 6). The diagnosis is shell-based and the genus is subdivided into two main groups of species as seen in Table 9. Charpentieria (Gibbularia) gibbula honii (O. Boettger, 1879) differs from the Charpentieria (Gibbularia) gibbula gibbula (Rossmässler, 1836) by virtue of a few details of the buccal armature and shape of the clausilium (Nordsieck 2013a: 6). Although considered as a subgenus of *Siciliaria* by Nordsieck (2013a: 4) highlighted the lack of the epiphallar thickening in *Gibbularia*.

**Table 9.** Siciliaria (Gibbularia) gibbula taxa as grouped by Nordsieck (2013a: 6).

Siciliaria (Gibbularia) gibbula (Rossmässler, 1836)	
Siciliaria (Gibbularia) gibbula gibbula (Rossmässler, 1836) **GROUP**	Siciliaria (Gibbularia) gibbula honii (O. Boettger, 1879) **GROUP**
Siciliaria (Gibbularia) gibbula gibbula (Rossmässler, 1836)	Siciliaria (Gibbularia) gibbula honii (O. Boettger, 1879)
Siciliaria (Gibbularia) gibbula multiplex (Westerlund, 1884)	
Siciliaria (Gibbularia) gibbula niethammeri (Rensch, 1934)	
Siciliaria (Gibbularia) gibbula pelagosana (O. Boettger, 1877)	
Siciliaria (Gibbularia) gibbula cf. sanctangeli (A. J. Wagner, 1925)	
Siciliaria (Gibbularia) gibbula selecta (Monterosato in Cecconi, 1908)	

In our study, two populations of *Gibbulariagibbulagibbula* underwent molecular genetic analysis and genital anatomical investigations, one from northern Italy: Muggia (Trieste) and one from Southern Italy: Apricena (Foggia) (Table 10). Their genital morphology is similar, with only slight differences such as the sculpturing of the internal penis (finely granulated vs. finely granulated + 1 weak smooth longitudinal pleats) and the presence of a weak epiphallar ring in the Apricena’s population. Both populations show a pseudopapilla directly originating from the epiphallar wall. Gibbulariagibbulacf.sanctangeli (not in the phylogenetic trees) from Gargano was also dissected and shows a remarkable different anatomy of the genital organs compared to the previous taxon. It shows an epiphallar ring that is connected to the ELP and also represents the origin of the pseudopapilla. In the mt tree, phylogenetic relationships among *Gibbularia* and the other genera, appear as a polytomy and low support values prevent us to provide any reliable statement. In the ITS2 tree, its distance from *Charpentieria* appears clear, preventing us to consider *Gibbularia* as a subgenus of *Charpentieria* as once listed by Nordsieck (2007).

**Table 10.** Examined taxa of *Gibbularia* and *Stigmatica* with information on availability of genetic data (DNA; Yes/No) and epiphallar formula.

Taxon	Examined specimens	Epiphallar formula	DNA
*Gibbulariagibbulagibbula* (Rossmässler, 1836)	Muggia, Europa Gardens, Trieste, Friuli-Venezia Giulia, Italy. 30 m asl. 45°36'17.64"N, 13°45'59.24"E. W. De Mattia and J. Macor leg. and det.	PP	Y
*Gibbulariagibbulagibbula* (Rossmässler, 1836)	Apricena, Foggia, Puglia, Italy. 75 m asl. 41°47'01.22"N, 15°26'39.92"E, H. Nordsieck leg and det.	ER(ELP)+PP	Y
Gibbulariagibbulacf.sanctangeli (A.J. Wagner, 1925)	Mattinata close to Vieste, Puglia, Italy. 500 m asl. 41°45'15.61"N, 16°04'56.31"E, W. De Mattia and J. Macor leg. and det.	ER(PP+ELP)	N
*Stigmaticastigmaticastigmatica* (Rossmässler, 1836)	Reka Dubrovačka, Sustjepan, Dubrovnik, Croatia. 5 m asl. 42°40'12.26"N, 18°05'38.24"E, W. De Mattia and J. Macor leg. and det.	ER(PP+ELP)	N
Stigmaticastigmaticacf.stigmatica (Rossmässler, 1836)	Rijeka Reževići, on the Budva–Petrovac road, 100 m asl. 42°13'33.84"N, 18°54'40.70"E, Deli, Erőss, Fehér leg. W. De Mattia det.	ER(PP+ELP)	N
*Stigmaticastigmatica* cf. *stigmatica* (Rossmässler, 1836)	Orjen Mts, Podi and Kameno, Herceg Novi-Trebinije Road, Montenegro. 325 m asl. 42°28'08.05"N, 18°32'29.17"E. Deli, Erőss, Fehér leg. W. De Mattia det.	ER(PP+ELP)	N
*Stigmaticastigmaticasturmii* (L. Pfeiffer, 1848)	Punta Penne next to the Airport, Brindisi, Puglia, Italy. 5 m asl. 40°40'31.60"N, 17°56'40.44"E, H. Nordsieck leg. and det.	PP	Y
*Stigmaticaernae* Fauer, 1978	San Rufo, Passo della Sentinella, Salerno, Campania, Italy. 850 m asl. 40°25'27.06"N, 15°26'17.44"E. W. De Mattia and J. Macor leg. and det.	PP+ELP	N
*Stigmaticaincerta* (Küster, 1861)	Serra San Bruno, Vibo Valentia, Calabria, Italy. 945 m asl. 38°32'42.36"N, 16°19'09.42"E, W. De Mattia and J. Macor leg. and det.	PP(ER)+ELP	N
*Stigmaticakobeltiana* (Küster, 1876)	Altilia, Cosenza, Calabria, Italy. 460 m asl. 39°07'58.01"N, 16°15'01.92" E, W. De Mattia and J. Macor leg. and det.	PP(ER)+ELP	N
*Stigmaticakobeltiana* (Küster, 1876)	Gerace, Strada Provinciale 1, 3.5 km NW of the town, Reggio Calabria, Calabria, Italy. 400 m asl. 38°16'53.42"N, 16°11'49.3"E, W.De Mattia and J. Macor leg. and det.	PP(ER)+ELP	N
*Stigmaticalamellata* (Rossmässler, 1836)	Gardiki fortress, Corfu, Greece. 40 m asl. 39°28'37.39"N, 19°53'05.74"E, W. De Mattia and J. Macor leg. and det.	PP+ELP	N
*Stigmaticapaestanapaestana* (Philippi, 1836) (= *S.p.semisculpta* Paulucci, 1878)	Monte Telegrafo, Argentario, Toscana, Italy. 600 m asl. 42°23'13.90"N, 11°10'03.98"E, A. Margelli leg. and det.	PP(ER)+ELP	N
*Stigmaticapaestanaintustructa* (Westerlund, 1883)	Balvano, Potenza, Basilicata; Italy. 450 m asl. 40°38'40.44"N, 15°30'39.09"E, I. Niero leg. and det.	PP(ELP)	N
*Stigmaticapaestanaintustructa* (Westerlund, 1883)	Balvano, near the castle, Potenza, Basilicata; Italy. 450 m asl. 40°39'01.23"N, 15°30'38.91"E, W. De Mattia and J. Macor leg. and det.	PP(ELP)	N
*Stigmaticapantocratorispantocratoris* (O. Boettger, 1889)	Lafki, Corfu, Greece. 420 m asl. 39°46'18.23"N, 19°50'47.23"E, W. De Mattia and J. Macor leg. and det.	PP+ELP	N
*Stigmaticasplendens* (Nordsieck, 1996)	Vlorë district, Qeparo, macchia with tree spurges W of the village, 20 m, 50 m asl.40°03'25.07"N, 19°49'47.06"E, Juhász, Kovács, Murányi, Puskás leg. W. De Mattia det.	ELP	N
*Stigmaticapantocratorismargaritifera* (Nordsieck, 1996)	Delvinë District, Krongj, rocky grassland at the peak region of Mt Koqinolithar, Albania. 890 m asl. 39°54'12.34"N, 20°10'11.39"E, Barina, Pifkó, Puskás leg. W. De Mattia det.	PP(ER)+ELP	N
*Stigmaticavulcanicavulcanica* (Benoit, 1860)	Cosenza, Via Porta Piana, Calabria, Italy. 300 m asl. 39°17'02.08"N, 16°15'30.22"E, H. Nordsieck leg. and det.	PP+ER(ELP)	Y
*Stigmaticavulcanicavulcanica* (Benoit, 1860)	Castello di San Lucido, Paola, Calabria, Italy. 120 m asl. 39°21'33.06"N, 16°02'39.97"E, I. Niero leg. and det.	PP+ER(ELP)	Y
*Stigmaticavulcanicavulcanica* (Benoit, 1860)	Amantea, Cosenza, Calabria, Italy. 30 m asl. 39°08'24.65"N, 16°04'18.13"E, I. Niero leg. and W. De Mattia det.	PP(ER)+ELP	N
*Stigmaticavulcanicavulcanica* (Benoit, 1860)	Contrada Piscitello, Belpasso, Catania, Sicily, Italy. 650 m asl. 37°36'13.96"N, 14°59'25.42"E, W. De Mattia and J. Macor leg. and det.	PP+ELP	N
*Stigmaticavulcanicasigridae* (Nordsieck, 2013)	Contrada Rizzuto, Cosenza, Calabria, Italy. 770 m asl. 39°14' 51.24"N, 16°10'30.51"E, W. De Mattia and J. Macor leg. and det.	PP(ER)+ELP	N
*Stigmaticavulcanicasigridae* (Nordsieck, 2013)	Contrada Rizzuto road to San Bartolo, Cosenza, Calabria, Italy. 740 m asl. 39°15'20.65"N, 16°11'09.16"E, W. De Mattia and J. Macor leg. and det.	PP(ER)+ELP	N

Gibbularia until now was considered as a subgenus of Siciliaria (MolluscaBase 2021; Nordsieck, 2013a). Our results indicate that no *Gibbularia* sample falls inside *Siciliaria*/*Sicania* subclades, suggesting a meaningful phylogenetic distance. Its position inside *Siciliaria* cannot be maintained, albeit its relationships with the other genera (mainly with *Stigmatica*), are still not clear. For this reason, we currently consider *Gibbularia* a valid genus until further results will be available.

##### ﻿ Gibbularia gibbula gibbula

Taxon classificationAnimaliaStylommatophoraClausiliidae

(Rossmässler, 1836)

EDBEFE71-4482-5B2B-AA66-D01FBD13B37E

Figs 57.1–57.4, 58.1–58.4

###### Distribution.

The actual distribution of the genus *Gibbularia* is far from being completely understood. This is mainly due to the passive anthropocorous dispersion and the absence of clear data about its centre of dispersion. Only a wide range was provided, that includes eastern peninsular and northeastern Italy as far as Istria to central Dalmatia (Alzona 1971: 89; Welter-Schultes 2012: 340; Nordsieck 2013a: 3). The subspecific taxa, except for the nominotypic one, present restricted to very restricted distribution ranges, e.g., *Gibbulariagibbulapelagosana* from Pelagosa or *Gibbulariagibbulaselecta* from Tremiti Islands (Alzona 1971: 89–90).

###### Specimens examined.

Italy, Friuli-Venezia Giulia, Muggia, Trieste, Europa Gardens. 20 m asl, 45°36'8.91"N, 13°46'0.72"E, [Lab ID G64_1, COI: MW758958, ITS2: MW757048, MW757082, MW757083; Lab ID G64_2, COI: MW758959], W. De Mattia and J. Macor leg. and det., 3 dissected spm.

###### External morphology of the genital organs

**(Fig. 57.3).** The V is slightly longer than the FO. The FDBC is slightly longer than the SDBC+BC. The BC+SDBC is club-like in shape, with a clear distinction between the SDBC and the BC. The D is much longer than the SDBC+BC and slightly thinner. The V is short but wide in diameter. The PC is ~ 2 × longer than the V. The P is cylindrical. The transition between P and EP is visible. The PR is very short and robust. The E is much shorter than the P and thin in diameter.

###### Internal morphology of the genital organs

**(Fig. 57.4).** The V, the A and the P show a smooth with a fine granulation that becomes coarser along the proximal P. The pseudopapilla is short, roundish and originates directly from the P-E transition wall. The epiphallar formula is: PP. The E shows a particular sculpturing made of extremely tiny transverse, interrupted micro-pleats.

###### Additional specimens examined.

Italy, Puglia, Foggia, Apricena. 75 m asl, 41°47'1.22"N, 15°26'39.92"E, [Lab ID 12167_1, COI: MW758947; Lab ID 12167_2, COI: MW758948, ITS2: MW757055, MW757056, MW757057], H. Nordsieck leg and det., 2 dissected spm.

###### External morphology of the genital organs

**(Fig. 57.1).** The V is half as long as the FO. The FDBC is 3 × longer than the SDBC+BC. The BC+SDBC is club-like in shape, with a clear distinction between the SDBC and the BC. The D is much longer than the SDBC+BC and slightly thinner. The V is moderately long but small in diameter. The PC is ~ 2 × longer than the V. The P is cylindrical. The transition between P and EP is not clearly visible. The PR is long and robust. The E is much shorter than the P and thin in diameter.

###### Internal morphology of the genital organs

**(Fig. 57.2).** The V and the A are smooth. The P presents four to five smooth longitudinal, little elevated pleats. The PP is short, roundish and originates directly from the P-E transition wall. The epiphallar formula is: ER(ELP)+PP. The weak distal ELP soon disappear revealing a smooth internal E.

##### ﻿ Gibbularia gibbula cf. sanctangeli

Taxon classificationAnimaliaStylommatophoraClausiliidae

(A. J. Wagner, 1925)

D80E120F-0385-51DD-8334-4FDF3C838305

Figs 57.5, 57.6, 58.6

###### Distribution.

The taxon is exclusively known from the southern slopes of Monte Sant’Angelo, Gargano Peninsula (Puglia, Italy) (Wagner 1925: 57).

###### Specimens examined.

Italy, Puglia, Mattinata close to Vieste, southern slopes of the Gargano Peninsula. 500 m asl, 41°45'15.61"N, 16°04'56.31"E, W. De Mattia and J. Macor leg. and det., 3 dissected spm.

###### External morphology of the genital organs

**(Fig. 57.5).** The V is 4 × shorter than the FO. The FDBC is much longer (2 ×) than the SDBC+BC. The BC+SDBC is club-like in shape, with a more or less clear distinction between the SDBC and the BC. The D is remarkably longer than the SDBC+BC. The V is extremely short but wide in diameter. The PC is ~ 3.5 ×longer than the V. The P is spindle-like and slightly swollen. The transition between P and EP is clearly visible. The PR is short and robust. The E is almost as long as the P but slightly thinner in diameter.

###### Internal morphology of the genital organs

**(Fig. 57.6).** The V is smooth. The P shows four to five main vast smooth pleats. These pleats are mainly longitudinally arranged but tend to become smaller and irregular to transversal along the proximal P. The pseudopapilla originates from the epiphallar thickening. The ER is connected with the ELP. The epiphallar formula is: ER(PP+ELP). The E shows four to five small longitudinal pleats that merge into two smooth ELP. They proximally fade before the VD.

###### Remarks.

As a shell, Gibbulariagibbulacf.sanctangeli shows reliable differences with the nominate subspecies, e.g., the bigger dimensions, the slender shape with flatter whorls, the suture without the darker band and the more prominent cervical keels. The principalis and the anterior upper palatal plicae are usually not visible from frontal view (Fig. 58.5). We cite this population as cf. sanctangeli, since the typical form is known from the type locality only and this one may represent a transitional form.

**Figure 57.**
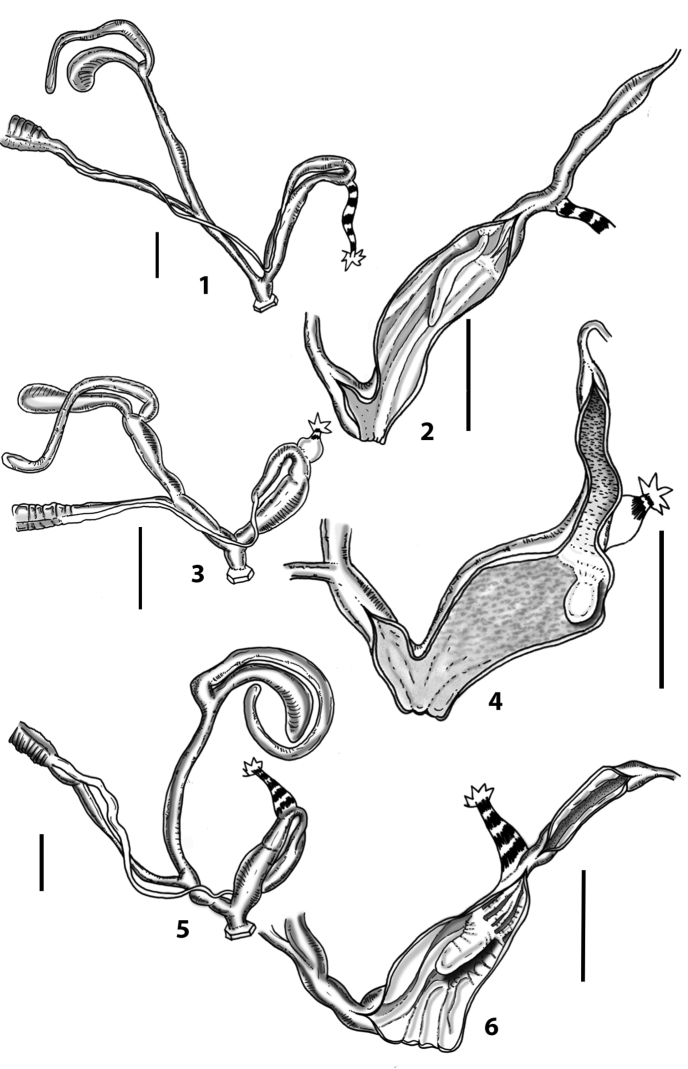
*Gibbulariagibbulagibbula* (Rossmässler, 1836). Apricena, Foggia **57.1** whole distal genital organs **57.2** internal distal part of genital organs. Muggia, Trieste **57.3** whole distal genital organs **57.4** internal distal part of genital organs. Gibbulariagibbulacf.sanctangeli (A. J. Wagner, 1925), Mattinana, Vieste **57.5** whole distal genital organs **57.6** internal distal part of genital organs.

####### ﻿Brief genital anatomical description of ten *Stigmatica* taxa

*Stigmatica* (type species *Clausiliastigmatica* Rossmässler, 1836 = Siciliaria (Stigmatica) stigmatica) is currently considered as a subgenus of *Charpentieria* (MolluscaBase 2021) probably based on Nordsieck (2002: 29). Nordsieck’s decision (2007, 2013a) where Stigmatica is again deemed as a subgenus of Siciliaria wasn’t adopted. *Stigmatica* includes 16 taxa among species and subspecies and presents a wide distribution, including taxa from the SW Balkans (S Dalmatia, NW Greece and Ionian Islands, Montenegro and Albania) and Peninsular Italy from Tuscany southward and E Sicily (Welter-Schultes 2012; MolluscaBase 2021), in a typical pattern of trans-Adriatic distribution as already known for, e.g., *Medora* (Nordsieck 1970; 2012). In order to assess the relationships between *Stigmatica* and *Siciliaria*, four taxa of *Stigmatica* have undergone both molecular and genital anatomical investigations. Three additional populations were exclusively anatomically investigated (Table 10). In both the COI and ITS2 trees, *Stigmatica* forms three independent lineages, distant from *Siciliaria/Sicania* (Figs 4–6). In the ITS2 tree the few available clones confirmed its polyphyly, as *Stigmaticapantocratorispantocratoris* from Corfu and *Stigmaticastigmaticasturmii* from Brindisi fall in different distant subclades. As a genital anatomical peculiarity, *Stigmatica* shows, even though rarely, the presence of a vestigial epiphallar flagellum, which has never been found in *Siciliaria*/*Sicania*. This structure, when present, is very difficult to be identified as extremely small and almost transparent. It was recorded in one specimen of *Stigmaticaincerta* and *Stigmaticavulcanicasigridae*.

**Figure 58.**
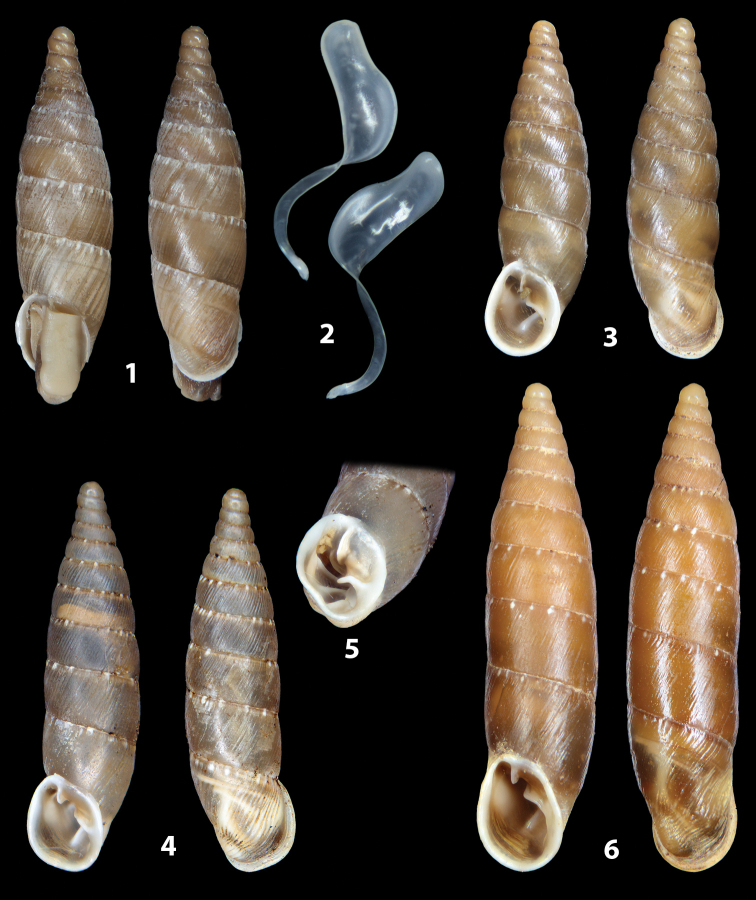
*Gibbulariagibbulagibbula* (Rossmässler, 1836). Apricena, Foggia **58.1** shell **57.2** clausiliar plate double side. Muggia, Trieste **58.3–58.4** shell **58.5** detail of the aperture. Gibbulariagibbulacf.sanctangeli (A. J. Wagner, 1925), Mattinana, Vieste **58.6** shell.

##### ﻿ Stigmatica stigmatica stigmatica

Taxon classificationAnimaliaStylommatophoraClausiliidae

(Rossmässler, 1836)

BE69101F-107D-5E1E-9F24-1E38B6A5D55C

Figs 59.1–59.6, 61.1–61.3

###### Distribution.

The nominate subspecies is distributed from southern Dalmatia (from the Neretva River southward), Herzegovina, Montenegro to northern Albania.

###### Specimens examined.

Croatia, Dubrovnik, Sustjepan, Reka Dubrovačka. 5 m asl, 42°40'12.26"N, 18° 05'38.24"E, W. De Mattia and J. Macor leg. and det., 2 dissected spm; Montenegro, Rijeka Reževići, on the Budva–Petrovac road, 100 m asl, 42°13'33.84"N, 18°54'40.70"E, Deli, Erőss, Fehér leg. W. De Mattia det., 2 dissected spm.; Montenegro, Orjen Mts, Podi and Kameno, Herceg Novi-Trebinije Road. 325 m asl, 42°28'8.05"N, 18°32'29.17"E Deli, Erőss, Fehér leg. W. De Mattia det., 2 dissected spm.

###### External morphology of the genital organs (Figs 59.1, 59.3, 59.5).

The V is much shorter than the FO. The FDBC is longer than the SDBC+BC. The BC+SDBC is club-like in shape, with a more or less clear distinction between the SDBC and the BC. The D is extremely longer than the SDBC+BC but remarkably thinner. The V is very short but wide in diameter. The PC is ~ 4–5 × longer than the V. The P is cylindrical or slightly swollen. The transition between P and EP is clearly visible. The PR is short but very robust. The E is usually as long as the P and thinner in diameter.

###### Internal morphology of the genital organs

**(Figs 59.2, 59.4, 59.6).** The V is smooth. The P shows a pattern of irregularly fringed or smooth pleats, sometimes forming and irregular net-like pattern, usually disappearing toward the A that is smooth. The pseudopapilla small and roundish. It originates from the E wall, which is connected with the ELP. The epiphallar formula is ER(PP+ELP). The E shows two fringed ELP, sometimes remarkably irregular and connected with small fleshy transversal bridges. They proximally fade before the VD.

##### ﻿ Stigmatica stigmatica sturmii

Taxon classificationAnimaliaStylommatophoraClausiliidae

(L. Pfeiffer, 1848)

9D621469-58E8-5042-8F37-DD0444BF68A6

Figs 60.1, 60.2, 61.4

###### Distribution.

This taxon is restricted to the Salento (Puglia, Italy) and central Albania with the type form (Nordsieck 2013a: 6).

###### Specimens examined:

Italy, Puglia, Brindisi, Punta Penne next to the Airport. 5 m asl, 40°40'31.60"N, 17°56'40.44"E, [Lab ID 12194_1, COI: MW758949, ITS2: MW757053, MW757054; Lab ID 12194_2, COI: MW758950], H. Nordsieck leg. and det. 2 dissected spm.

**Figure 59.**
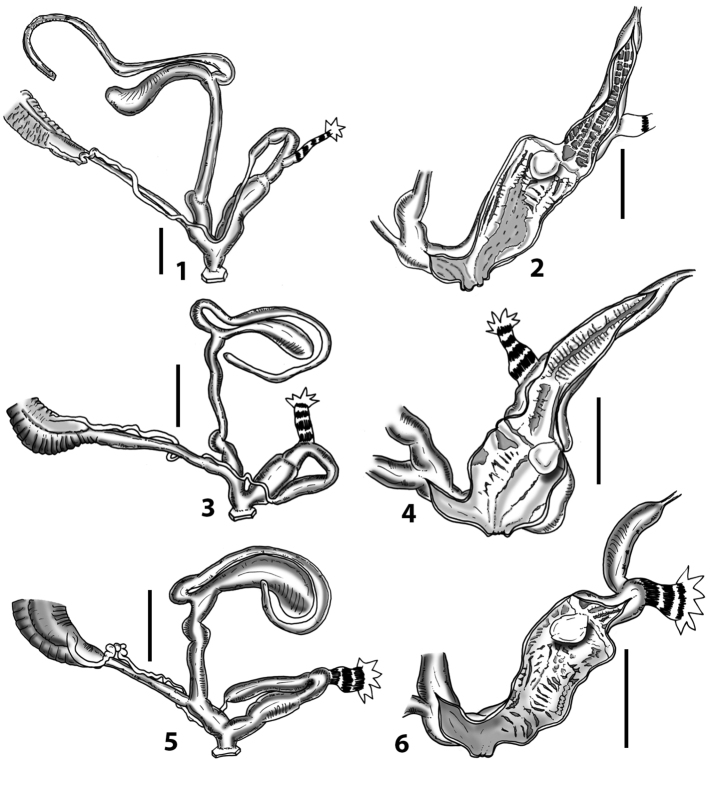
*Stigmaticastigmaticastigmatica* (Rossmässler, 1836), Dubrovnik, HR **59.1** whole distal genital organs **59.2** internal distal part of genital organs. Stigmaticastigmaticacf.stigmatica (Rossmässler, 1836). Budva, Montenegro **59.3** whole distal genital organs **59.4** internal distal part of genital organs. Orjen Mountains, Montenegro **59.5** whole distal genital organs **59.6** internal distal part of genital organs.

###### External morphology of the genital organs

**(Fig. 60.1).** The V is shorter than the FO. The FDBC is longer than the SDBC+BC. The BC+SDBC is club-like in shape, with a more or less clear distinction between the SDBC and the BC. The D is much longer than the SDBC+BC and slightly thinner. The V is moderately long and thin in diameter. The PC is ~ 2 × longer than the V. The P is slim and cylindrical. The transition between P and EP is not visible. The PR is long and robust. The E is shorter than the P and thinner in diameter.

###### Internal morphology of the genital organs

**(Fig. 60.2).** The V is smooth. The P shows a single smooth longitudinal pleat that reaches the atrium. The pseudopapilla is elongated with an irregular surface. It originates from the E wall. The epiphallar formula is PP+ELP. The E shows two smooth ELP. They proximally fade before the VD.

###### Remarks.

The morphology of the genital organs of this population is remarkably different from all other *Stigmatica*, and it shows high affinity with the genus *Gibbularia*. This is also suggested by the ITS2 tree, where *G.gibbulagibbula* from Apricena and this population cluster together implying that this population might were misidentified and belongs to the genus *Gibbularia*. However, in the COI tree, *S.s.sturmii* forms a very distant lineage. Thus, this taxon certainly deserves thorough investigation with a larger sample size.

##### ﻿ Stigmatica ernae

Taxon classificationAnimaliaStylommatophoraClausiliidae

(Fauer, 1978)

5ADAAE83-DACC-5F7A-8AA9-CE91F23BD51C

Figs 60.3, 60.4, 61.5, 61.6

###### Distribution.

This species is restricted to the eastern side of the Monti Alburni, Campania, Italy.

###### Specimens examined.

Italy, Campania, Salerno, San Rufo, Passo della Sentinella. 850 m asl, 40°25'27.06"N, 15°26'17.44"E. W. De Mattia and J. Macor leg. and det., 3 dissected spm.

###### External morphology of the genital organs

**(Fig. 60.3).** The FO is 4–5 ×longer than the V. The FDBC is 2 × as long as the SDBC+BC. The BC+SDBC is slightly club-like in shape, with a more or less clear distinction between the SDBC and the BC. The D is longer than the SDBC+BC. The V is short. The PC is ~ 3 × longer than the V. The P is irregular and folded in shape. The transition between P and E is slightly visible. The PR is very long and slim. The E is half as long as the P and thinner in diameter.

###### Internal morphology of the genital organs

**(Fig. 60.4).** The V is smooth. The P shows five to six irregular transverse smooth pleats that stop before entering in the atrium. The pseudopapilla is moderately short with a smooth surface. It originates from the E wall. The epiphallar formula is PP+ELP. The E shows two smooth ELP. They proximally fade before the VD.

###### Remarks.

*Stigmaticaernae* shows an extremely long FO and, as a whole, the female part of the distal genital organs shows an ectomorphic type (except as regards the vagina) whereas the PC is relatively short.

##### ﻿ Stigmatica incerta

Taxon classificationAnimaliaStylommatophoraClausiliidae

(Küster, 1861)

45F9DC09-EAD3-58D3-A0AC-AC8E4549A732

Figs 60.5, 60.6, 61.7

###### Distribution.

*Stigmaticaincerta* is found in the Madonie and Nebrodi mountains in Sicily and in southern Calabria.

###### Specimens examined.

Italy, Calabria, Vibo Valentia, Serra San Bruno. 945 m asl, 38°32'42.36"N, 16°19'09.42"E, W. De Mattia and J. Macor leg. and det., 3 dissected spm.

###### External morphology of the genital organs

**(Fig. 60.5).** The FO is 2.5 × as long as the V. The FDBC is remarkably longer than the SDBC+BC. The BC+SDBC is short, bulky, club-like in shape, with a more or less clear distinction between the SDBC and the BC. The D is much longer than the SDBC+BC and thinner. The PC is ~ 4 × longer than the V. The P is moderately wide in diameter and swollen. The transition between P and EP is clearly visible. The PR is long and robust. The E is longer than the P but thinner in diameter.

###### Internal morphology of the genital organs

**(Fig. 60.6).** The V presents few smooth longitudinal pleats, distally merging one into another, that stop immediately before entering the A. The P shows three or four slightly fringed pleats that continue almost as far as the A. These penial pleats tend to distally merge one into the other. The pseudopapilla is moderately long and smooth. It originates directly from the E wall, which is connected with the ELP by thin smooth longitudinal pleats. The two ELP are moderately fringed. The epiphallar formula is: PP(ER)+ELP.

###### Remarks.

This species is restricted to higher altitudes from 800 m asl up. It is found in mixed *Fagus* and *Picea* forests on barks and moss in humid habitats. Benoit (1876: 152) reports this taxon also on barks of *Acercampestre* (maple) from the Madonie and *Quercus* sp. (oak) from Gibilmanna (Palermo).

##### ﻿ Stigmatica kobeltiana

Taxon classificationAnimaliaStylommatophoraClausiliidae

(Küster, 1876)

0BE5B2FC-8862-5B78-A555-15931D31EC28

Figs 60.7–60.12, 61.8–61.11

###### Distribution.

Calabria, Italy (Nordsieck 2013a: 7).

###### Specimens examined.

Italy, Calabria, Cosenza, Altilia. 460 m asl, 39°07'58.01"N, 16°15'01.92"E, [Lab ID St11_1, COI: MW758965], W. De Mattia and J. Macor leg. and det., 2 dissected spm; Italy, Calabria, Reggio Calabria, Gerace, Strada Provinciale 1, 3.5 km NW of the town. 400 m asl, 38°16'53.42"N, 16°11'49.3"E, W. De Mattia and J. Macor leg. and det., 2 dissected spm; Italy, Calabria, Cosenza, Amantea. 30 m asl, 39°08'24.65"N, 16°04'18.13"E, W. I. Niero leg., W. De Mattia det., 1 dissected spm.

###### External morphology of the genital organs

**(Figs 60.7, 60.9, 60.11).** The V is as long as the FO. The FDBC is longer than the SDBC+BC. The BC+SDBC is club-like in shape, with a more or less clear distinction between the SDBC and the BC. The D is much longer than the SDBC+BC and slightly thinner. The V is long but thin in diameter. The PC is ~ 2 × longer than the V. The P is wide in diameter and swollen. The transition between P and EP is clearly visible. The PR is long and robust. The E is slightly longer than the P but thinner in diameter.

###### Internal morphology of the genital organs

**(Figs 60.8, 60.10, 60.12).** The V presents many smooth longitudinal pleats that stop immediately before entering the A. The P shows five to seven longitudinal to irregularly fringed pleats that continue as far as the A where they become smooth. These penial pleats are irregular and randomly split and merge again one into the other and irregularly split or merge one into another. The pseudopapilla is smooth, short to moderately long. It originates from the ER which is connected to the ELP by means of three or four smooth longitudinal pleats. The two ELP are slightly fringed and fade before entering the VD. The epiphallar formula is PP(ER)+ELP.

##### ﻿ Stigmatica lamellata

Taxon classificationAnimaliaStylommatophoraClausiliidae

(Rossmässler, 1836)

5B2CEEDD-0EA3-5CB0-BB25-7102C03FFC7C

Figs 60.13–60.15, 61.12

###### Distribution.

The species is distributed in southwestern Albania and Corfu, Greece (Welter-Schultes 2012).

###### Specimens examined.

Greece, Corfu, Gardiki fortress. 40 m asl, 39°28'37.39"N, 19°53'5.74"E, W. De Mattia and J. Macor leg. and det., 3 dissected spm.

###### External morphology of the genital organs

**(Fig. 60.13).** The V is much longer than the FO. The FDBC is shorter than the SDBC+BC. The BC+SDBC is club-like in shape, with a clear distinction between the SDBC and the BC. The SDBC appears very thin. The D is shorter than the SDBC+BC and slightly thinner. The V is very long, cylindrical and thin in diameter. The PC is ~ 2 × longer than the V. The P is cylindrical spindle-like. The transition between P and EP is only slightly visible and an ER is not clearly detectable. The PR is long and robust. The E is longer than the P but thinner in diameter.

###### Internal morphology of the genital organs

**(Figs 60.14–60.15).** The V is smooth. The P shows transversal, huge smooth pleats that stop before the A. The pseudopapilla is elongated, subquadrangular and smooth. The pseudopapilla originated directly from the epiphallar wall and its base is connected to one EPL. The ER is missing. The epiphallar formula is: PP+ELP. The E shows two smooth ELP. They proximally fade before the VD.

##### ﻿ Stigmatica paestana paestana

Taxon classificationAnimaliaStylommatophoraClausiliidae

(Philippi, 1836)

A7960F85-3E22-5C9C-9126-65FDA13F211D

Figs 62.1–62.3, 64.1

###### Distribution.

The nominate subspecies is widespread in central and southern peninsular Italy [from Tuscany (Monte Argentario) and S. Abruzzo to N. Calabria] (Nordsieck 2013a: 7).

###### Specimens examined.

Italy, Toscana, Grosseto, Porto Ercole, Monte Telegrafo. 600 m asl, 42°23'13.90"N, 11°10'3.98"E, A. Margelli leg. and det., 2 dissected spm.

###### External morphology of the genital organs

**(Fig. 62.1).** The V is much shorter than the FO. The FDBC is as long as the SDBC+BC. The BC+SDBC is club-like in shape, with a more or less clear distinction between the SDBC and the BC. The D extremely long, much longer than the SDBC+BC and much thinner. The V is very short but wide in diameter. The PC is ~ 5 × longer than the V. The P is cylindrical. The transition between P and EP is clearly visible with a narrowing and the presence of the ER. The PR is long and robust. The E is slightly longer than the P and similar in diameter.

**Figure 60.**
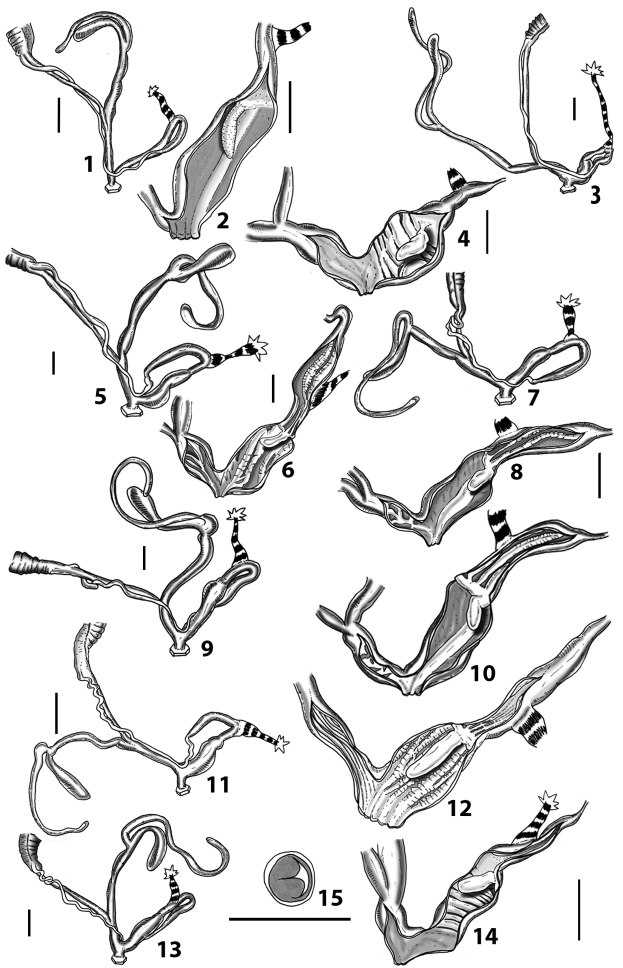
*Stigmaticastigmaticasturmii* (L. Pfeiffer, 1844), Punta Penne, Brindisi **60.1** whole distal genital organs **60.2** internal distal part of genital organs. *Stigmaticaernae* Fauer, 1978, San Rufo, Passo Sentinella **60.3** whole distal genital organs **60.4** internal distal part of genital organs. *Stigmaticaincerta* (Küster, 1861), Serra San Bruno, Vibo Valentia **60.5** whole distal genital organs **60.6** internal distal part of genital organs. *Stigmaticakobeltiana* (Küster, 1876). Altilia, Cosenza **60.7** whole distal genital organs. **60.8** internal distal part of genital organs; Gerace **60.9** whole distal genital organs **60.10** internal distal part of genital organs; Amantea, Cosenza **60.11** whole distal genital organs **60.12** internal distal part of genital organs. *Stigmaticalamellata* (Rossmässler, 1836), Corfu, Greece **60.13** whole distal genital organs. **60.14** internal distal part of genital organs **60.15** cross section of the epiphallus.

**Figure 61.**
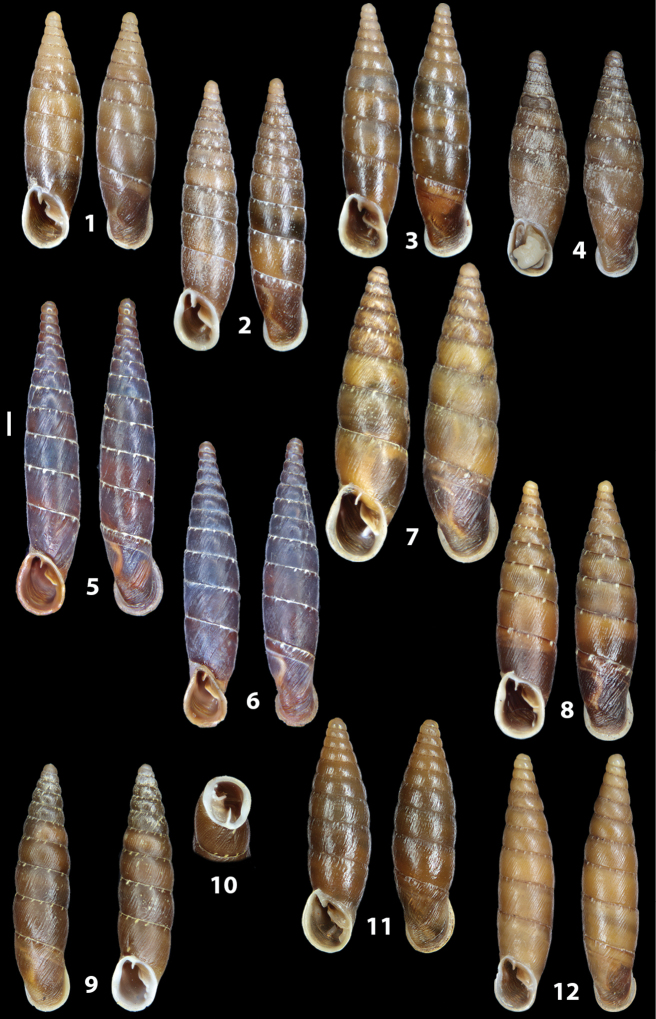
*Stigmaticastigmaticastigmatica* (Rossmässler, 1836), Dubrovnik, HR **61.1** shell. Stigmaticastigmaticacf.stigmatica (Rossmässler, 1836), Budva, MONT **61.2** shell. Stigmaticastigmaticacf.stigmatica (Rossmässler, 1836), Orjen Mountains, MONT **61.3** shell. *Stigmaticastigmaticasturmii* (L. Pfeiffer, 1844), Punta Penne, Brindisi **61.4** shell. *Stigmaticaernae* Fauer, 1978, San Rufo, Passo Sentinella **61.5–61.6** shell. *Stigmaticaincerta* (Küster, 1861), Serra San Bruno, Vibo Valentia **61.7** shell. *Stigmaticakobeltiana* (Küster, 1876). Altilia, Cosenza **61.8** shell. Gerace, Reggio Calabria **61.9** shell **61.10** detail of the aperture; Amantea, Cosenza **61.11** shell. *Stigmaticalamellata* (Rossmässler, 1836), Corfu, Greece. 61.12. shell.

###### Internal morphology of the genital organs

**(Figs 62.2, 62.3).** The V, the A and the P lack any macro-sculpturing. The internal surface shows a coarse fine granulation. The pseudopapilla is cylindrical with a smooth surface. The pseudopapilla originates from a weak ER that is not connected with the ELP. The E shows two moderately fringed ELP. They proximally fade before the VD. The epiphallar formula is PP(ER)+ELP.

##### ﻿ Stigmatica paestana intustructa

Taxon classificationAnimaliaStylommatophoraClausiliidae

(Westerlund, 1883)

C95FB041-88F9-579E-981D-8B6CEAE6798F

Figs 62.4, 62.5, 64.2

###### Distribution.

This taxon is present in Basilicata, known from the area surrounding Balvano (Italy) (Nordsieck 2013b: 7).

**Figure 62.**
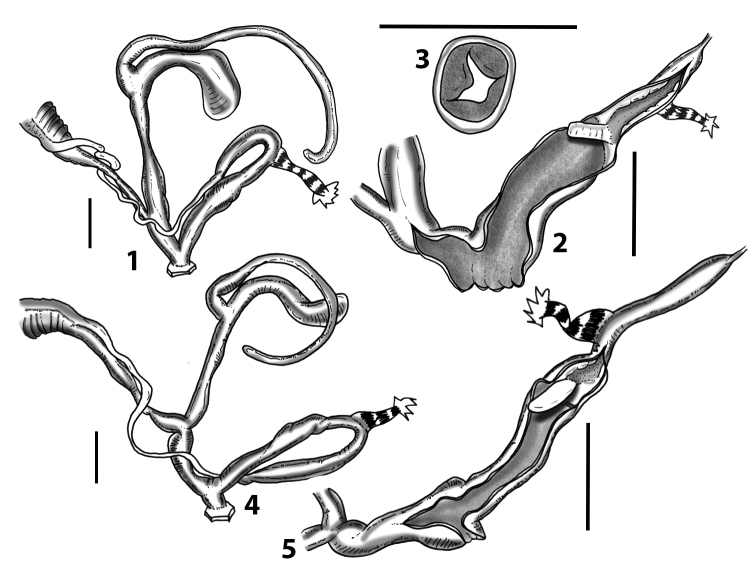
*Stigmaticapaestanapaestana* (Philippi, 1836), Monte Argentario, Grosseto **62.1** whole distal genital organs **62.2** internal distal part of genital organs **62.3** cross section of the epiphallus. *Stigmaticapaestanaintustructa* (Westerlund, 1883), Balvano, Potenza **62.4** whole distal genital organs **62.5** internal distal part of genital organs.

###### Specimens examined.

Italy, Basilicata, Potenza, Balvano, near the castle. 450 m asl, 40°39'01.23"N, 15°30'38.91"E, W. De Mattia and J. Macor leg. and det., 3 dissected spm; Italy, Basilicata, Potenza, Balvano. 450 m asl, 40°38'40.44"N, 15°30'39.09"E, I. Niero leg. and det., 2 dissected spm.

###### External morphology of the genital organs

**(Fig. 62.4).** The V is much shorter than the FO. The FDBC is longer than the SDBC+BC. The BC+SDBC is slightly club-like in shape, with a more or less clear distinction between the SDBC and the BC. The D is much longer than the SDBC+BC and much thinner. The V is short but wide in diameter. The PC is ~ 4 × longer than the V. The P is cylindrical and slightly swollen. The transition between P and EP is clearly visible with a narrowing and the presence of the ER. The PR is short and robust. The E is longer than the P.

###### Internal morphology of the genital organs

**(Fig. 62.5).** The V, the A and the P lack any macro-sculpturing. The internal surface shows a coarse fine granulation. The pseudopapilla is elongated and with a smooth surface. It originates directly from the wall of the E. Its basis is connected with one ELP. The E shows two moderately fringed ELP. They proximally fade before the VD. The epiphallar formula is PP(ELP).

###### Remarks.

*Stigmaticapaestanaintustructa* shows an anatomy of the genital organs very similar to the nominate subspecies.

##### ﻿ Stigmatica pantocratoris pantocratoris

Taxon classificationAnimaliaStylommatophoraClausiliidae

(O. Boettger, 1889)

E83D523C-BB5D-5E1C-A18A-2BAB9F31057E

Figs 63.1, 63.2, 64.3

###### Distribution.

The nominate subspecies is restricted to the Island of Corfu, Greece.

###### Specimens examined.

Greece, Corfu, Lafki. 420 m asl, 39°46'18.23"N, 19°50'47.23"E, [Lab ID St9_2, COI: MW758964, ITS2: MW757041, MW757042, MW757043], W. De Mattia and J. Macor leg. and det., 2 dissected spm.

###### External morphology of the genital organs

**(Fig. 63.1).** The V is much shorter than the FO. The FDBC is longer than the SDBC+BC. The BC+SDBC is spindle-like in shape, with a clear distinction between the SDBC and the BC. The D is much longer than the SDBC+BC and much thinner. The V is short but wide in diameter. The PC is ~ 2.5 × longer than the V. The P is cylindrical and slightly swollen. The transition between P and EP is not clearly visible and ER is difficult to detect. The PR is short and robust. The E is longer than the P but thinner in diameter.

###### Internal morphology of the genital organs

**(Fig. 63.2).** The V presents many smooth longitudinal pleats that stop before entering the A. The A and the P are smooth. The pseudopapilla is smooth, long and occupies almost the whole penial volume. It originates directly from the wall of the E and it is not connected with the ELP. The E shows two moderately fringed ELP. They proximally fade before the VD. The epiphallar formula is PP+ELP.

##### ﻿ Stigmatica pantocratoris margaritifera

Taxon classificationAnimaliaStylommatophoraClausiliidae

(Nordsieck, 1996)

7AA69837-206F-5949-85EE-9CEF864E0BAA

Figs 63.5, 63.6, 64.4

###### Distribution.

*Stigmaticapantocratorismargaritifera* is known from northern Greece up to Kozani and Veria, SW Albania (Gjirokastër, Delvinë and Sarandë districts), and the Greek-Albanian border region of Epirus near Konitsa (Nordsieck 1996: 11).

**Figure 63.**
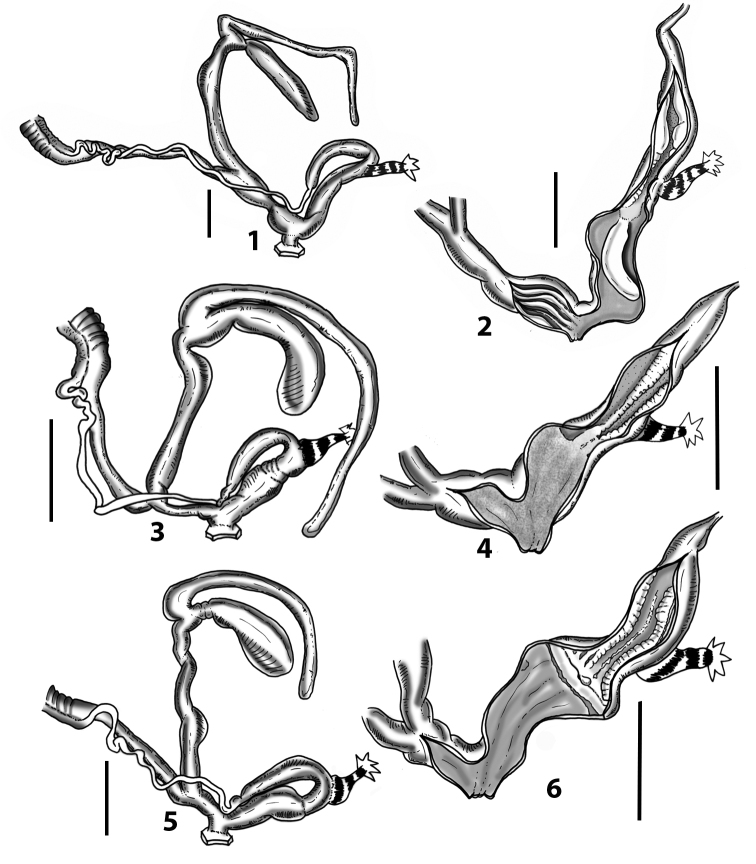
*Stigmaticapantocratorispantocratoris* (O. Boettger, 1889), Pantocrator, Corfu **63.1** whole distal genital organs **63.2** internal distal part of genital organs. *Stigmaticasplendens* (Nordsieck, 1996), Qeparo, AL **63.3** whole distal genital organs **63.4** internal distal part of genital organs. *Stigmaticapantocratorismargaritifera* (Nordsieck, 1996), Krongj, AL **63.5** whole distal genital organs **63.6** internal distal part of genital organs.

###### Specimens examined.

Albania, Delvinë District, Krongj, rocky grassland at the peak region of Mt Koqinolithar. 890 m asl, 39°54'12.34"N, 20°10'11.39"E, Barina, Pifkó, Puskás leg. W. De Mattia det., 2 dissected spm.

###### External morphology of the genital organs

**(Fig. 63.5).** The V is much shorter than the FO. The FDBC is longer than the SDBC+BC. The BC+SDBC is large, club-like in shape, with a more or less clear distinction between the SDBC and the BC. The D is much longer than the SDBC+BC and thinner in diameter. The V is very short but large in diameter. The PC is ~ 5 × longer than the V. The P is large and proximally folded. The transition between P and EP is clearly visible with an evident epiphallar narrowing is not evident. The ER is visible. The PR is short and robust. The E is longer than the P and not much thinner in diameter.

###### Internal morphology of the genital organs

**(Fig. 63.6).** The V, the A and the P lack any macro-sculpturing. The internal surface shows a very fine granulation. The pseudopapilla is extremely reduced, almost vestigial. It originates from a well-developed ER that seems not to be connected with the ELP. The E shows two fringed ELP. They proximally fade before the VD. The epiphallar formula is PP(ER)+ELP.

###### Remarks.

Mainland populations of *Stigmaticapantocratoris* revealed a reduction or the complete lack of the pseudopapilla, whereas it is well developed in the nominate subspecies on the island of Corfu. More anatomical research would be necessary to improve the knowledge about the character states of the pseudopapilla, in order to properly assess, together with genetic molecular data, the status of these taxa.

##### ﻿ Stigmatica splendens

Taxon classificationAnimaliaStylommatophoraClausiliidae

(Nordsieck, 1996)

92D0A952-7112-5112-9351-B35C1D2CD607

Figs 63.3, 63.4, 64.4

###### Distribution.

This taxon is restricted to southern Albania from Tepelenë area near Lum i Bençës (Nordsieck 1996: 12).

###### Specimens examined.

Albania, Vlorë district, Qeparo, macchia with tree spurges W of the village, 20 m, 50 m asl, 40°03'25.07"N, 19°49'47.06"E, Juhász, Kovács, Murányi, Puskás leg. W. De Mattia det., 2 dissected spm.

###### External morphology of the genital organs

**(Fig. 63.3).** The V is much shorter than the FO. The FDBC is shorter than the SDBC+BC. The BC+SDBC is club-like in shape, large, with a more or less clear distinction between the SDBC and the BC. The D is extremely long, much longer than the SDBC+BC and slightly thinner. The V is short and very thin in diameter. The PC is ~ 3 × longer than the V. The P is large and proximally folded. The transition between P and EP is clearly visible even if the epiphallar narrowing is not evident. The ER is visible. The PR is short and robust. The E is longer than the P but thinner in diameter.

###### Internal morphology of the genital organs

**(Fig. 63.4).** The V, the A and the P lack any macro-sculpturing. The internal surface shows a coarse fine granulation. The pseudopapilla is missing. The transition between P and E is visible. The E shows two fringed ELP that start with two smooth longitudinal pleats. They proximally fade before the VD. The epiphallar formula is: ELP.

**Figure 64.**
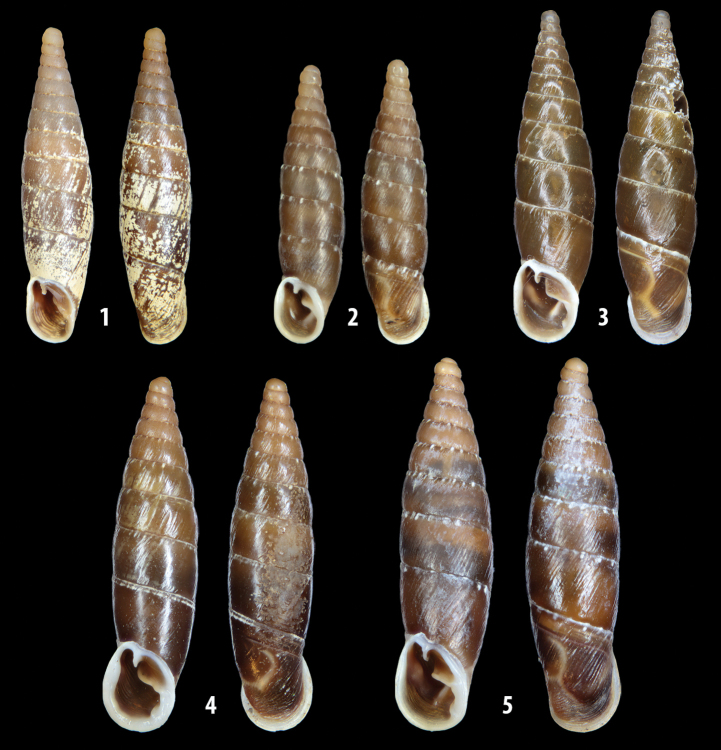
*Stigmaticapaestanapaestana* (Philippi, 1836), Monte Argentario, Grosseto **64.1** shell. *Stigmaticapaestanaintustructa* (Westerlund, 1883), Balvano, Potenza **64.2** shell. *Stigmaticapantocratorispantocratoris* (O. Boettger, 1889), Pantocrator, Corfu **64.3** shell. *Stigmaticasplendens* (Nordsieck, 1996), Qeparo, AL **64.4** shell. *Stigmaticapantocratorismargaritifera* (Nordsieck, 1996), Krongj, AL **64.5** shell.

###### Remarks.

The lack of the pseudopapilla represents a possible apomorphic state of the epiphallus as already seen in other genera (e.g., *Montenegrina*, De Mattia 2020).

##### ﻿ Stigmatica vulcanica vulcanica

Taxon classificationAnimaliaStylommatophoraClausiliidae

(Benoit, 1860)

E094522F-3052-548A-98FE-B54DF49C37DF

Figs 65.1–65.6, 66.1–66.3

###### Distribution.

*Stigmaticavulcanicavulcanica* is found in northeastern Sicily, Eaolian Islands and Calabria (Nordsieck 2013a: 7).

###### Specimens examined.

Italy, Calabria, Cosenza, Via Porta Piana. 300 m asl, 39°17'2.08"N, 16°15'30.22"E, [Lab ID 12068_2, COI: MW758943, ITS2: MW757060, MW757061], H. Nordsieck leg. and det., 2 dissected spm; Italy, Calabria, Paola, Castello di San Lucido, Via Porta Piana. 120 m asl, 39°21'33.06"N, 16°02'39.97"E, I. Niero leg. and det., 1 dissected spm; Italy, Calabria, Cosenza, Amantea. 30 m asl, 39°08'24.65"N, 16°04'18.13"E, W. I. Niero leg., W. De Mattia det., 1 dissected spm; Italy, Sicily, Catania, Belpasso, Contrada Piscitello. 650 m asl, 37°36'13.96"N, 14°59'25.42"E, W. De Mattia and J. Macor leg. and det., 2 dissected spm.

###### External morphology of the genital organs

**(Figs 65.1, 65.3, 65.5, 65.7).** The V can be as long as or shorter or longer than the FO. The FDBC is longer than the SDBC+BC. The BC+SDBC is club-like to slightly elongated in shape, with a more or less clear distinction between the SDBC and the BC. The D is as long as or much longer than the SDBC+BC and thinner. The PC is ~ 2 × longer than the V. The P is cylindrical and slightly swollen. The transition between P and EP is clearly visible and two samples out of nine showed a strong ET. The PR is long and robust. The E is longer than the P but thinner in diameter.

###### Internal morphology of the genital organs

**(Figs 65.2, 65.4, 65.6, 65.8).** Belpasso, Catania, Sicily (Fig. 65.2): The V is smooth. The P shows a chevron-like pattern that gradually disappears as approaching the A. The proximal penis presents an irregular pattern of smooth small pleats. The pseudopapilla is very long and reaches the A. It is smooth and slightly irregularly folded at its base. The ER is not present. The E distally shows many small irregular pleats that proximally merge into two smooth ELP. They proximally fade before the VD. The epiphallar formula is: PP(ELP).

Cosenza and Paola, Calabria (Figs 65.4, 65.6): The V is smooth. The P shows many fine smooth longitudinal pleats that tend to fade towards the A. The pseudopapilla is very long and almost reaches the A. It is smooth and blade-shaped. A strong ER is present. The pseudopapilla originates from the E wall, independent from the ER and it is connected to the ELP by means of 3 to 8 smooth longitudinal pleats. The epiphallar formula is: PP+ER(ELP). The two ELP are smooth and fade before entering the VD.

###### Remarks.

*Stigmaticavulcanicavulcanica* showed a variable morphology of the genital organs. The Sicilian samples present an anatomy of the genital organs that greatly differs from those from Calabria. The internal penis of two populations from Cosenza shows longitudinal pleats vs. the chevron pattern of the Sicilian samples. Moreover, the pseudopapilla and the transition between P and E are different (presence of the ER vs. absence of ER).

Unfortunately, genetic molecular data are not yet available from topotypic populations from the Mongibello-Etna (Sicily) thus, although the anatomical investigations highlight reliable differences, the relationships with and the status of the Calabrian populations still have to be properly assessed.

**Figure 65.**
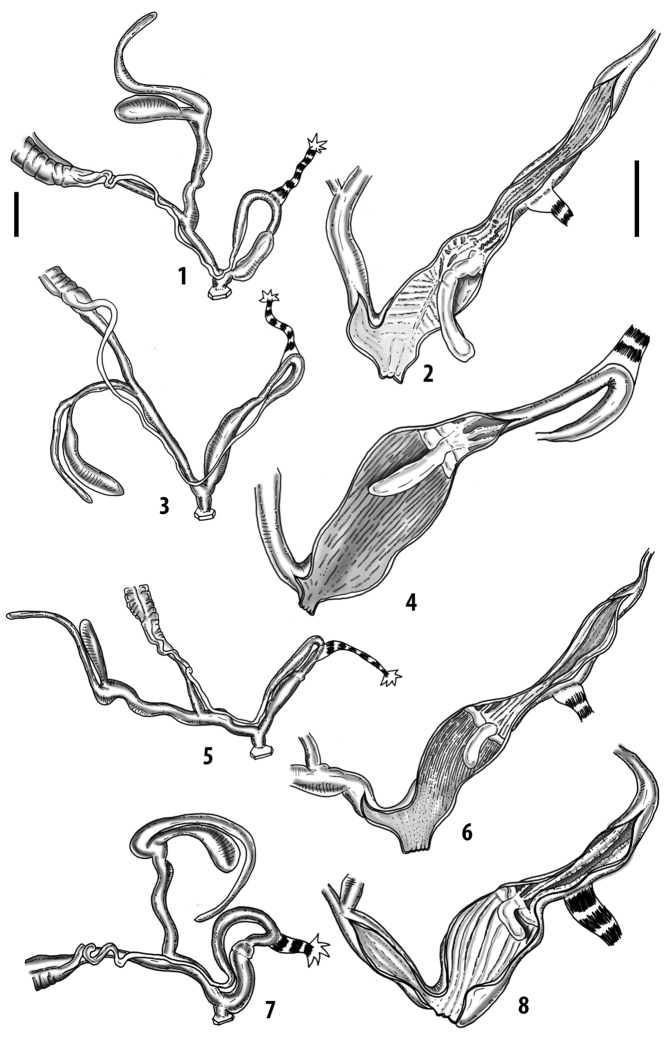
*Stigmaticavulcanicavulcanica* (Benoit, 1860), Belpasso, Catania **65.1** whole distal genital organs **65.2** internal distal part of genital organs. Cosenza **65.3** whole distal genital organs **65.4** internal distal part of genital organs. Paola **65.5** whole distal genital organs **65.6** internal distal part of genital organs. *Stigmaticavulcanicasigridae* Nordsieck, 2013, Mendicino, Cosenza **65.7** whole distal genital organs **65.8** internal distal part of genital organs.

##### ﻿ Stigmatica vulcanica sigridae

Taxon classificationAnimaliaStylommatophoraClausiliidae

Nordsieck, 2013

3769474E-8D23-5501-B481-080CD589FB23

Figs 65.7, 65.8, 66.4, 66.5

###### Distribution.

*Stigmaticavulcanicasigridae* is restricted to a small range east of Cosenza in central Calabria (Nordsieck 2013a: 10).

###### Specimens examined.

Italy, Calabria, Cosenza, Contrada Rizzuto. 770 m asl, 39°14'51.24"N, 16°10'30.51"E, W. De Mattia and J. Macor leg. and det., 2 dissected spm; Italy, Calabria, Cosenza, Contrada Rizzuto road to San Bartolo, Cosenza, Calabria, Italy. 740 m asl, 39°15'20.65"N, 16°11'09.16"E, W. De Mattia and J. Macor leg. and det., 2 dissected spm.

**Figure 66.**
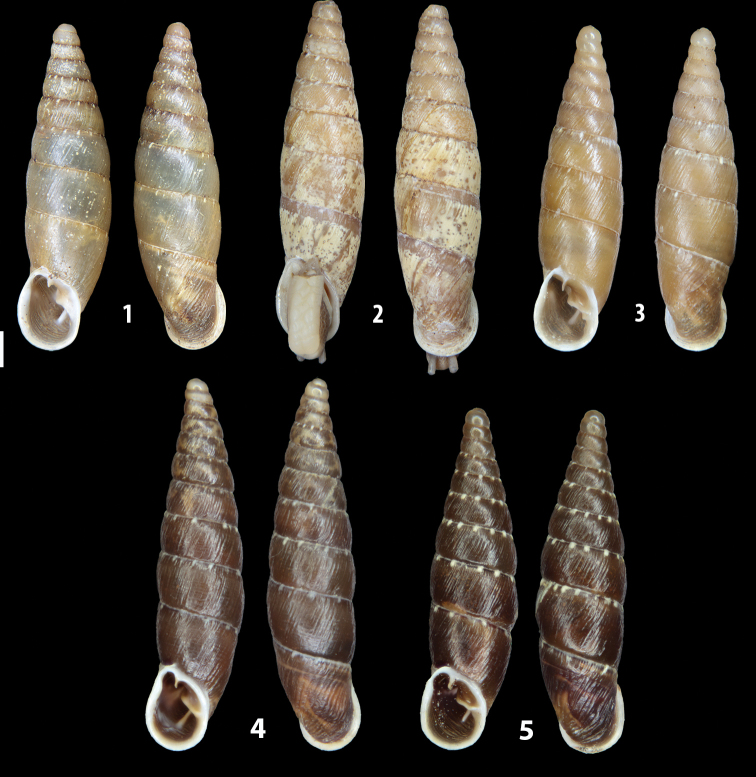
*Stigmaticavulcanicavulcanica* (Benoit, 1860), Belpasso, Catania **66.1** shell. Cosenza **66.2** shell; Paola **66.3** shell. *Stigmaticavulcanicasigridae* Nordsieck, 2013, Mendicino, Cosenza **66.4–66.5** shells.

###### External morphology of the genital organs

**(Fig. 65.9).** The V as long as the FO. The FDBC is 1.5 × longer than the SDBC+BC. The BC+SDBC is club-like in shape, with a clear distinction between the SDBC and the BC. The D is much longer than the SDBC+BC and remarkably thinner. The V is wide in diameter. The PC is ~ 2.5 × longer than the V. The P is cylindrical and slightly swollen. The transition between P and EP is clearly visible with a strong ET. The PR is short and robust. The E is longer than the P but thinner in diameter.

###### Internal morphology of the genital organs

**(Fig. 65.10).** The V shows a weak longitudinal sculpturing. The P shows 6 to 8 smooth longitudinal pleats that fade as entering into the A. The pseudopapilla is smooth and short. It originates from the ER which is connected to the ELP by means of three or four smooth longitudinal pleats. The two ELP are slightly fringed and fade before entering the VD. The epiphallar formula is PP(ER)+ELP.

###### Remarks.

*Stigmaticavulcanicasigridae* was described based on shell characters that slightly differ from the nominate subspecies (Nordsieck 2013a: 10). Its anatomy of the genital organs, except for the smooth (instead of fringed) longitudinal penial pleats, matches with the nominate subspecies populations from Calabria but nevertheless keeps reliable differences with the Sicilian samples.

##### ﻿ Papillifera

Taxon classificationAnimaliaStylommatophoraClausiliidae

Hartmann, 1842

86CEAA0B-13E1-5F7C-B575-05D571BDFC4D

###### Remarks.

The genus *Papillifera* was used as the outgroup genus in the molecular genetic analysis (Figs 4–6). The general features of the genus *Papillifera* were summarised by Nordsieck (2013a: 8) who provided two lists of synonyms without any explanation or justification. *Papillifera* includes two species: *P.papillaris* and *P.solida* (Margelli 2014; MolluscaBase 2021). The current known distribution and status of its (sub)specific taxa is far of being satisfactorily assessed, due to the unknown or inadequately evaluated validity of many old names.

*Papillifera* is clearly distinguishable from *Siciliaria* s. s., *Sicania* and *Stigmatica* by its G-type clausiliar apparatus and the general shell features (e.g., the sutural dark band, see Fig. 68.1–68.7). In our study, we dissected the *Papillifera* samples that underwent molecular genetic investigations, which are shown in Fig. 67.1–67.12. The genital anatomical drawings of *Papilliferapapillarispapillaris* (O. F. Müller, 1774) (Fig. 67.1–67.2), *Papilliferapapillarisaffinis* (Philippi, 1836) (Fig. 67.3–67.8), *Papilliferapapillaristransitans* (Paulucci, 1878) (Figs 67.9–67.10), and *Papilliferasolidasolida* (Draparnaud, 1805) (Fig. 67.11–67.12) show a stable genital structure in dimensions, ratios among the main genital parts and internal features.

Nordsieck (2013a: 8) highlights that the anatomy of the genital organs of *Papillifera* is the same as *Siciliaria* s. l. Following our anatomical investigations, this statement must be rejected, as the general internal architecture of the PC (P+E and transition) revealed to be substantially different from *Siciliaria*/*Sicania* and *Stigmatica*. In detail, *Papillifera* lacks the ET and the connecting longitudinal pleats between PP and the ELP. The epiphallar formula of *Papillifera* is: PP+ELP. The sculpturing of the internal walls of the penis are mainly smooth or with very soft longitudinal pleats. The pseudopapilla is elongated to rounded, smooth and unrooted, directly emerging from the P-E transition walls. The E shows two smooth ELP.

**Table 11.** Examined taxa of *Papillifera* with information on availability of genetic data (DNA) and description of the variability penis-epiphallus transition (epiphallar formula).

Taxon	Examined specimens	Epiphallar formula	DNA
*Papilliferapapillarispapillaris* (O.F. Müller, 1774)	Vicovaro, Latina, Lazio, Italy. 320 m asl. 42°01'04.58"N, 12°54'19.30"E, H. Nordsieck leg. and det.	PP+ELP	Y
*Papilliferapapillarisaffinis* (Philippi, 1836)	Jato Antica, Monreale, Sicily, Italy, 830 m asl, 37°58'2.36"N, 13°11'45.19"E, W. De Mattia and J. Macor leg. and det.	PP+ELP	Y
*Papilliferapapillarisaffinis* (Philippi, 1836)	western part of Monte Kumeta toward Jato Antica, Monreale, Sicily, Italy, 630 m asl, 37°57'8.18"N, 13°13'14.57"E, W. De Mattia and J. Macor leg. and det.	PP+ELP	Y
*Papilliferapapillarisaffinis* (Philippi, 1836)	Monte Bonifato, west side of the mountain, over the quarry, Alcamo, Sicily, Italy. 550 m asl, 37°57'16.92"N, 12°58'09.06"E, W. De Mattia and J. Macor leg. and det.	PP+ELP	Y
*Papilliferapapillaristransitans* (Paulucci, 1878)	road to the Norman Castle, Stilo, Reggio Calabria, Calabria, Italy. 320 m asl. 38°28'45.91"N, 16°27'48.05"E, H. Nordsieck leg. and det.	PP+ELP	Y

**Table 12.** New *Siciliaria*/*Sicania* checklist and comparison with lists provided by Nordsieck (2013b) and MolluscaBase eds. (2021).

Nordsieck 2013b	MolluscaBase 2021	De Mattia, Reier and Haring 2021
*Siciliariagrohmanniana* (Rossmässler, 1836)	*Charpentieriagrohmanniana* (Rossmässler, 1836)	*Siciliariagrohmannianagrohmanniana* (Rossmässler, 1836)
		*Siciliariagrohmannianaaddaurae* ssp. nov. De Mattia, Reier and Haring
*Siciliariaseptemplicata* (Philippi, 1836)	*Charpentieriaseptemplicata* (Philippi, 1836)	*Siciliariaseptemplicata* (Philippi, 1836)
*Siciliarialeucophryna* (L. Pfeiffer, 1862)	*Charpentierialeucophryna* (L. Pfeiffer, 1862)	*Siciliarialeucophryna* (L. Pfeiffer, 1862)
*Siciliariacalcarae* (Philippi 1844)	*Charpentieriacalcarae* (Philippi, 1844)	*Siciliariacalcaraecalcarae* (Philippi 1844)
*Siciliariacalcaraebelliemii* (Brandt, 1961)	*Charpentieriacalcaraebelliemii* (Brandt, 1961)	*Siciliariacalcaraebelliemii* (Brandt, 1961)
		*Siciliariacalcaraeborgettensis* ssp. nov. De Mattia, Reier and Haring
		*Siciliariacalcaraejatinensis* ssp. nov. De Mattia, Reier and Haring
		*Siciliariacalcaraeparajatinensis* ssp. nov. De Mattia, Reier and Haring
	*Charpentieriacalcaraeorlandoi* Liberto, Reitano, Giglio, Colomba & Sparacio, 2016	*Siciliariacalcaraeorlandoi* Liberto, Reitano, Giglio, Colomba & Sparacio, 2016
		*Siciliariacalcaraecruenta* ssp. nov. De Mattia, Reier and Haring
*Siciliariaferrox* (Brandt, 1961)	*Charpentieriaferrox* (Brandt, 1961)	*Siciliariaferrox* (Brandt, 1961)
*Siciliariariberothi* (Brandt, 1961)	*Charpentieriariberothi* (Brandt, 1961)	see Nordsieck (2013b)
*Siciliariatiberiitiberii* (A. Schmidt, 1868)	*Charpentieriatiberii* (A. Schmidt, 1868)	*Siciliariatiberiitiberii* (A. Schmidt, 1868)
		*Siciliariatiberiiarmettensis* ssp. nov. De Mattia, Reier and Haring
*Siciliariaseptemplicataalcamoensis* Brandt, 1961	*Siciliariaseptemplicataalcamoensis* Brandt, 1961	*Siciliariatiberiialcamoensis* Brandt, 1961 comb. nov.
	*Charpentieriaseptemplicatahemmeni* Beckmann, 2004	
*Siciliariatiberiiscalettensis* Beckmann, 2004	*Charpentieriatiberiiscalettensis* Beckmann, 2004	*Siciliariatiberiiscalettensis* Beckmann, 2004
*Siciliariacrassicostata* (L. Pfeiffer, 1856)	*Siciliariacrassicostata* (L. Pfeiffer, 1856)	*Sicaniacrassicostata* (L. Pfeiffer, 1856) comb. nov.
*Siciliariaeminens* (A. Schmidt, 1868)	*Charpentieriaeminens* (A. Schmidt, 1868)	*Sicaniaeminens* (A. Schmidt, 1868) comb. nov.
*Siciliarianobilis* (L. Pfeiffer, 1848)	*Charpentierianobilis* (L. Pfeiffer, 1848)	*Sicanianobilisnobilis* (L. Pfeiffer, 1848) comb. nov.
*Siciliariaspezialensis* (Nordsieck, 1984)	*Charpentieriaspezialensis* (Nordsieck, 1984)	*Sicanianobilisspezialensis* (Nordsieck, 1984) comb. nov.; stat. nov.
*Siciliariascarificata* (L. Pfeiffer 1856)	*Charpentieriascarificata* (L. Pfeiffer 1856)	*Mauritanicascarificata* (L. Pfeiffer 1856) comb. nov.

**Figure 67.**
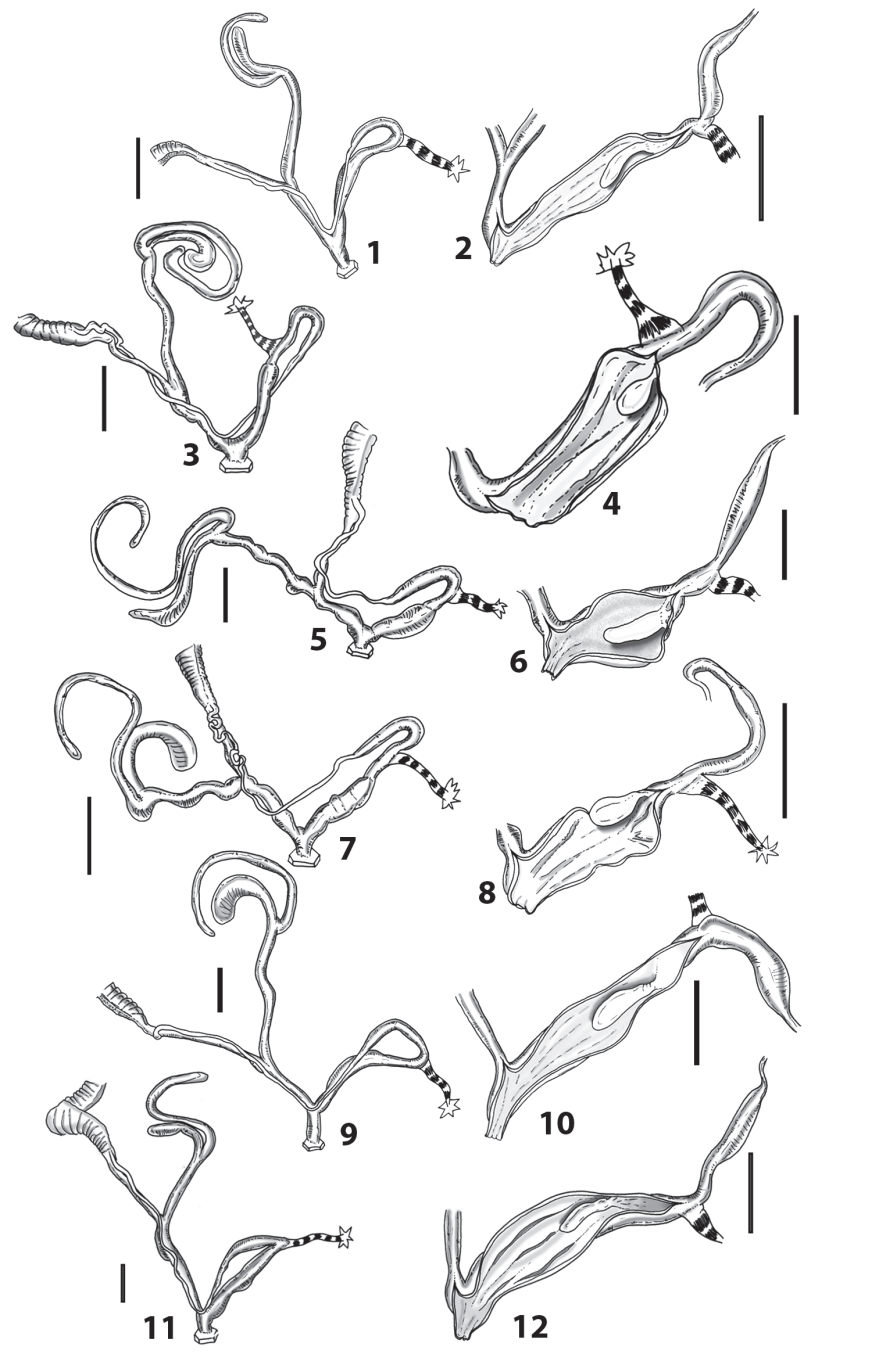
*Papilliferapapillarispapillaris* (O.F. Müller, 1774). Vicovaro, Latina **67.1** whole distal genital organs **67.2** internal distal part of genital organs. *Papilliferapapillarisaffinis* (Philippi, 1836). Jato Antica, San Giuseppe Jato **67.3** whole distal genital organs **67.4** internal distal part of genital organs. Monte Bonifato, Alcamo **67.5** whole distal genital organs **67.6** internal distal part of genital organs. Western slopes Monte Kumeta, Monreale **67.7** whole distal genital organs **67.8** internal distal part of genital organs. *Papilliferapapillaristransitans* (Paulucci, 1878). Road to the Norman Castle, Stilo **67.9** whole distal genital organs **67.10** internal distal part of genital organs. *Papilliferasolidasolida* (Draparnaud, 1805), Matera **67.11** whole distal genital organs **67.12** internal distal part of genital organs.

**Figure 68.**
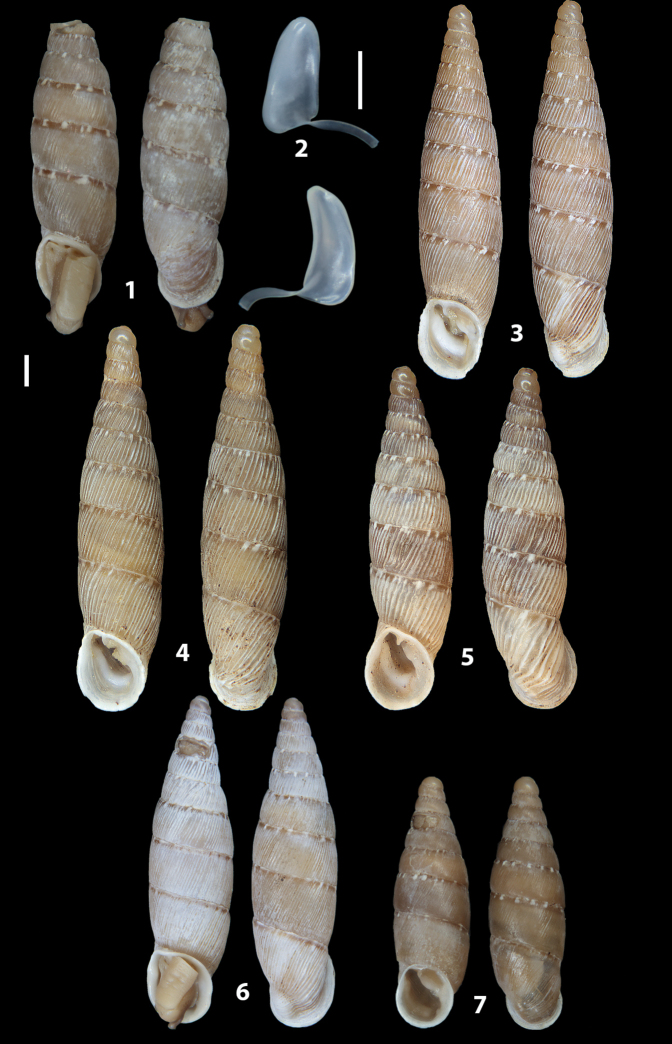
*Papilliferapapillarispapillaris* (O.F. Müller, 1774). Vicovaro, Latina **68.1** shell **68.2** clausiliar plate. *Papilliferapapillarisaffinis* (Philippi, 1836). Jato Antica, San Giuseppe Jato **68.3** shell. Monte Bonifato, Alcamo **68.4** shell. Western slopes Monte Kumeta, Monreale **68.5** shell. *Papilliferapapillaristransitans* (Paulucci, 1878). Road to the Norman Castle, Stilo **68.6** shell. *Papilliferasolidasolida* (Draparnaud, 1805), Matera **68.7** shell.

**Figure 69.**
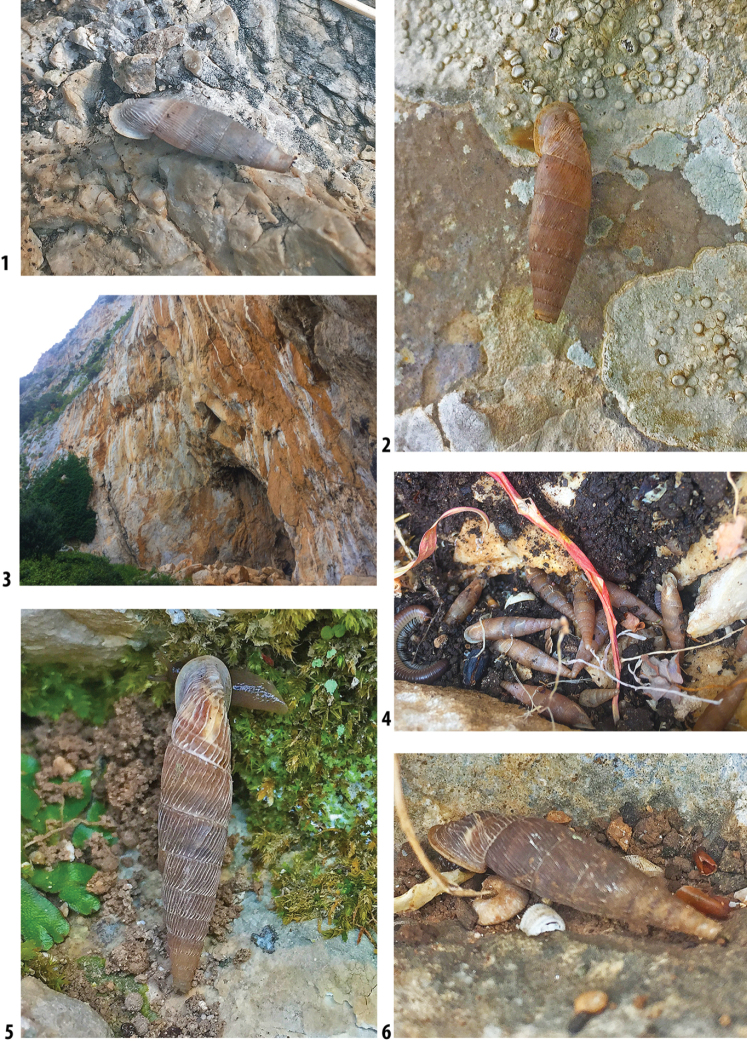
**69.1***Siciliariagrohmannianagrohmanniana* (Rossmässler, 1836), Monte Pellegrino, Palermo **69.2***Siciliariagrohmannianaaddaurae* ssp. nov. Grotta dell’Addaura, Punta Priola **69.3** Limestone walls at Grotta dell’Addaura, Punta Priola **69.4***Siciliariacalcaraebelliemii* (Brandt, 1961), Castello Calatubo, Alcamo **69.5***Siciliariacalcaraeparajatinensis* ssp. nov., west side Monte Kumeta **69**.**6***Siciliariatiberiiarmettensis* ssp. nov., Grotta dei Puntali, Carini.

**Figure 70.**
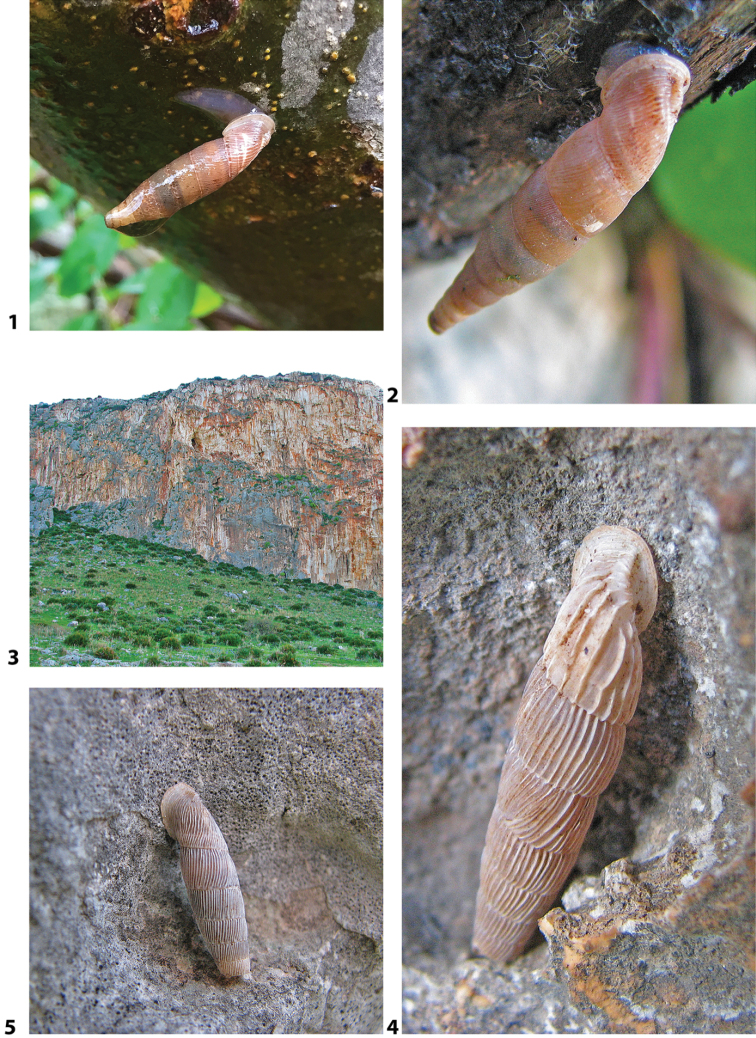
**70.1***Siciliarialeucophryna* (L. Pfeiffer, 1862), Grotta Conza, Palermo **70.2***Siciliariatiberiiscalettensis* Beckmann, 2004 Portella Scaletta, Villagrazia **70.3** Limestone walls at Monte Cofano, Custonaci **70.4***Sicaniacrassicostata* (L. Pfeiffer, 1856), comb. nov., Monte Cofano, Custonaci **70.5***Sicanianobilisspezialensis* (Nordsieck 1984), stat. nov., comb. nov. Macari.

**Figure 71.**
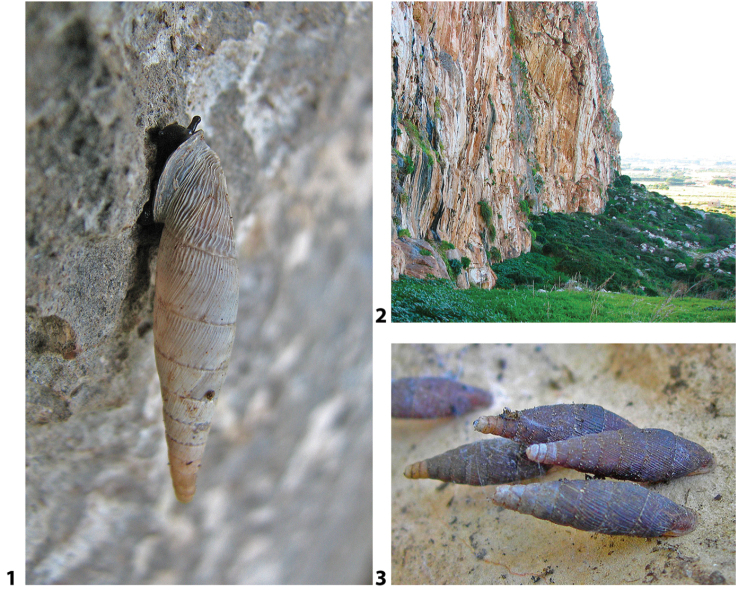
**71.1***Sicanianobilisnobilis* (L. Pfeiffer, 1848), comb. nov., Monte Cofano, Tonnara Cofano **71.2** Limestone walls at Monte Cofano, Tonnara Cofano **71.3***Mauritanicascarificata* (L. Pfeiffer, 1856), comb. nov., Marettimo.

## ﻿Conclusions

The system of *Siciliaria* s. l. that we propose here is based on an integrative approach, as was performed with the recently revised genus *Montenegrina* (De Mattia et al. 2020; Mason et al. 2020). It combines information on genital organs, shell morphology, and DNA sequence data which is a new approach for this genus. A comprehensive and detailed revision of the genus, including related Delimini genera such as *Charpentieria*, *Stigmatica*, *Gibbularia*, and *Papillifera* is still missing in the literature on Clausiliidae. We are aware that the proposed system, particularly of related genera, is based on incomplete sampling and thus has to be considered as preliminary. However, we are aware that a single straightforward, universally applicable rationale on how species should be delimited in Delimini (and Clausiliidae in general) is absent and taxonomic decisions remain arbitrary.

For the genus *Montenegrina* (De Mattia et al. 2020), the morphology of the genital characters of *Siciliaria*/*Sicania* was remarkably helpful for alpha-taxonomy but gave poor contribution to the phylogenetic reconstruction. At an upper-genus level, the epiphallar thickening(s) failed to represent a discriminatory character among *Siciliaria*/*Sicania* and the related genera *Charpentieria*, *Gibbularia*, *Stigmatica*, and *Papillifera*. This study revealed the complexity and variability of the genital characters of a genus thought to display low, if no, morphological variability at all. Finally, the shell-based system (Fig. 2) does not reflect the phylogeny of *Siciliaria*/*Sicania* (and *Mauritanica* as regards the Sicilian taxon).

## Supplementary Material

XML Treatment for Siciliaria grohmanniana

XML Treatment for Siciliaria grohmanniana grohmanniana

XML Treatment for Siciliaria grohmanniana addaurae

XML Treatment for Siciliaria septemplicata

XML Treatment for Siciliaria leucophryna

XML Treatment for Siciliaria calcarae

XML Treatment for Siciliaria calcarae calcarae

XML Treatment for Siciliaria calcarae belliemii

XML Treatment for Siciliaria calcarae borgettensis

XML Treatment for Siciliaria calcarae jatinensis

XML Treatment for Siciliaria calcarae parajatinensis

XML Treatment for Siciliaria calcarae orlandoi

XML Treatment for Siciliaria calcarae cruenta

XML Treatment for Siciliaria ferrox

XML Treatment for Siciliaria tiberii

XML Treatment for Siciliaria tiberii tiberii

XML Treatment for Siciliaria tiberii alcamoensis

XML Treatment for Siciliaria tiberii armettensis

XML Treatment for Siciliaria tiberii scalettensis

XML Treatment for Sicania crassicostata

XML Treatment for Sicania eminens

XML Treatment for Sicania nobilis

XML Treatment for Sicania nobilis nobilis

XML Treatment for Sicania nobilis spezialensis

XML Treatment for Mauritanica scarificata

XML Treatment for Mauritanica perinni polygyra

XML Treatment for Charpentieria dyodon dyodon

XML Treatment for Charpentieria dyodon alpina

XML Treatment for Charpentieria dyodon thomasiana

XML Treatment for Charpentieria ornata

XML Treatment for Charpentieria itala itala

XML Treatment for Charpentieria itala albopustolata

XML Treatment for Charpentieria itala allatollae

XML Treatment for Charpentieria itala baldensis

XML Treatment for Charpentieria itala balsamoi

XML Treatment for Charpentieria itala clavata

XML Treatment for Charpentieria itala latestriata

XML Treatment for Charpentieria itala lorinae

XML Treatment for Charpentieria itala zalloti

XML Treatment for Charpentieria itala serravalensis

XML Treatment for Charpentieria itala trepida

XML Treatment for Charpentieria itala triumplinae

XML Treatment for Charpentieria itala variscoi

XML Treatment for Charpentieria stenzii stenzii

XML Treatment for Charpentieria stenzii butoti

XML Treatment for Charpentieria stenzii cincta

XML Treatment for Charpentieria stenzii faueri

XML Treatment for Charpentieria stenzii letochana

XML Treatment for Charpentieria stenzii nordsiecki

XML Treatment for Charpentieria stenzii paroliniana

XML Treatment for Charpentieria stenzii westerlundi

XML Treatment for Gibbularia gibbula gibbula

XML Treatment for Gibbularia gibbula cf. sanctangeli

XML Treatment for Stigmatica stigmatica stigmatica

XML Treatment for Stigmatica stigmatica sturmii

XML Treatment for Stigmatica ernae

XML Treatment for Stigmatica incerta

XML Treatment for Stigmatica kobeltiana

XML Treatment for Stigmatica lamellata

XML Treatment for Stigmatica paestana paestana

XML Treatment for Stigmatica paestana intustructa

XML Treatment for Stigmatica pantocratoris pantocratoris

XML Treatment for Stigmatica pantocratoris margaritifera

XML Treatment for Stigmatica splendens

XML Treatment for Stigmatica vulcanica vulcanica

XML Treatment for Stigmatica vulcanica sigridae

XML Treatment for Papillifera
